# Speciation and kinetics of fluoride transfer from tetra-*n*-butylammonium difluorotriphenylsilicate (‘TBAT’)†

**DOI:** 10.1039/d3sc05776c

**Published:** 2023-12-07

**Authors:** Maciej M. Kucharski, Allan J. B. Watson, Guy C. Lloyd-Jones

**Affiliations:** a School of Chemistry, University of Edinburgh David Brewster Road Edinburgh EH9 3FJ UK guy.lloyd-jones@ed.ac.uk; b School of Chemistry, University of St Andrews North Haugh, St Andrews KY16 9ST UK

## Abstract

Tetra-*n*-butylammonium difluorotriphenylsilicate (TBAT) is a conveniently handled anhydrous fluoride source, commonly used as a surrogate for tetra-*n*-butylammonium fluoride (TBAF). While prior studies indicate that TBAT reacts rapidly with fluoride acceptors, little is known about the mechanism(s) of fluoride transfer. We report on the interrogation of the kinetics of three processes in which fluoride is transferred from TBAT, in THF and in MeCN, using a variety of NMR methods, including chemical exchange saturation transfer, magnetisation transfer, diffusion analysis, and 1D NOESY. These studies reveal ion-pairing between the tetra-*n*-butylammonium and difluorotriphenylsilicate moieties, and a very low but detectable degree of fluoride dissociation, which then undergoes further equilibria and/or induces decomposition, depending on the conditions. Degenerate exchange between TBAT and fluorotriphenylsilane (FTPS) is very rapid in THF, inherently increases in rate over time, and is profoundly sensitive to the presence of water. Addition of 2,6-di-*tert*-butylpyridine and 3 Å molecular sieves stabilises the exchange rate, and both dissociative and direct fluoride transfer are shown to proceed in parallel under these conditions. Degenerate exchange between TBAT and 2-naphthalenyl fluorosulfate (ARSF) is not detected at the NMR timescale in THF, and is slow in MeCN. For the latter, the exchange is near-fully inhibited by exogenous FTPS, indicating a predominantly dissociative character to this exchange process. Fluorination of benzyl bromide (BzBr) with TBAT in MeCN-*d*_3_ exhibits moderate progressive autoinhibition, and the initial rate of the reaction is supressed by the presence of exogenous FTPS. Overall, TBAT can act as a genuine surrogate for TBAF, as well as a reservoir for rapidly-reversible release of traces of it, with the relative contribution of the pathways depending, *inter alia*, on the identity of the fluoride acceptor, the solvent, and the concentration of endogenous or exogenous FTPS.

## Abstract 2

^19^F NMR analysis of ion-pairing and reaction kinetics shows that TBAT can act as a genuine surrogate for TBAF, as well as a reservoir for rapidly-reversible release of traces of it, to effect both direct (*k*_d_) and dissociative (*k*_F_) fluoride transfer.

## Graphical abstract

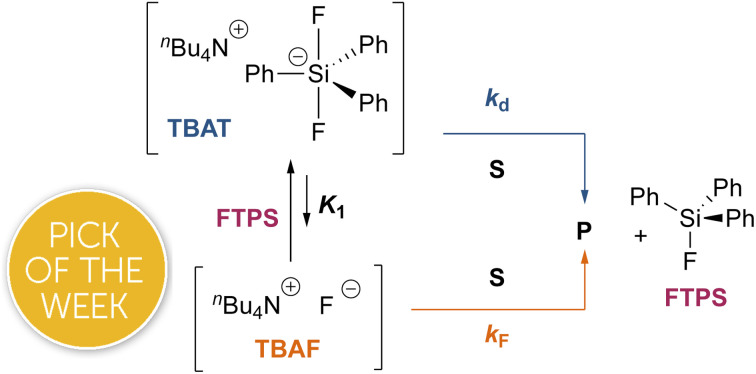

## Introduction

**Fig. 1:**
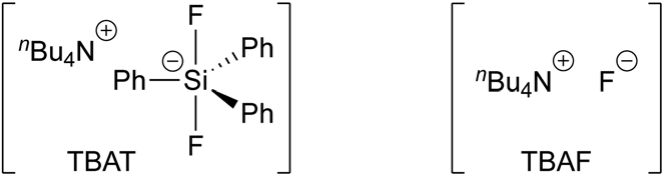
Tetra-*n*-butylammonium difluorotriphenylsilicate (TBAT) and tetra-*n*-butylammonium fluoride (TBAF).

Tetra-*n*-butylammonium difluorotriphenylsilicate (TBAT, Fig. 1) was introduced by DeShong^1^ in 1995 as a convenient alternative to tetra-*n*-butylammonium fluoride (TBAF), and is widely employed, *inter alia*, for C–F generation,^1*a*,2^ deprotection,^3^ benzyne generation,^4^ and anion generation by Si–X cleavage (X = C,^5^ N,^6^ O,^7^ S;^7*d*^).^8,9^

**Scheme 1:**
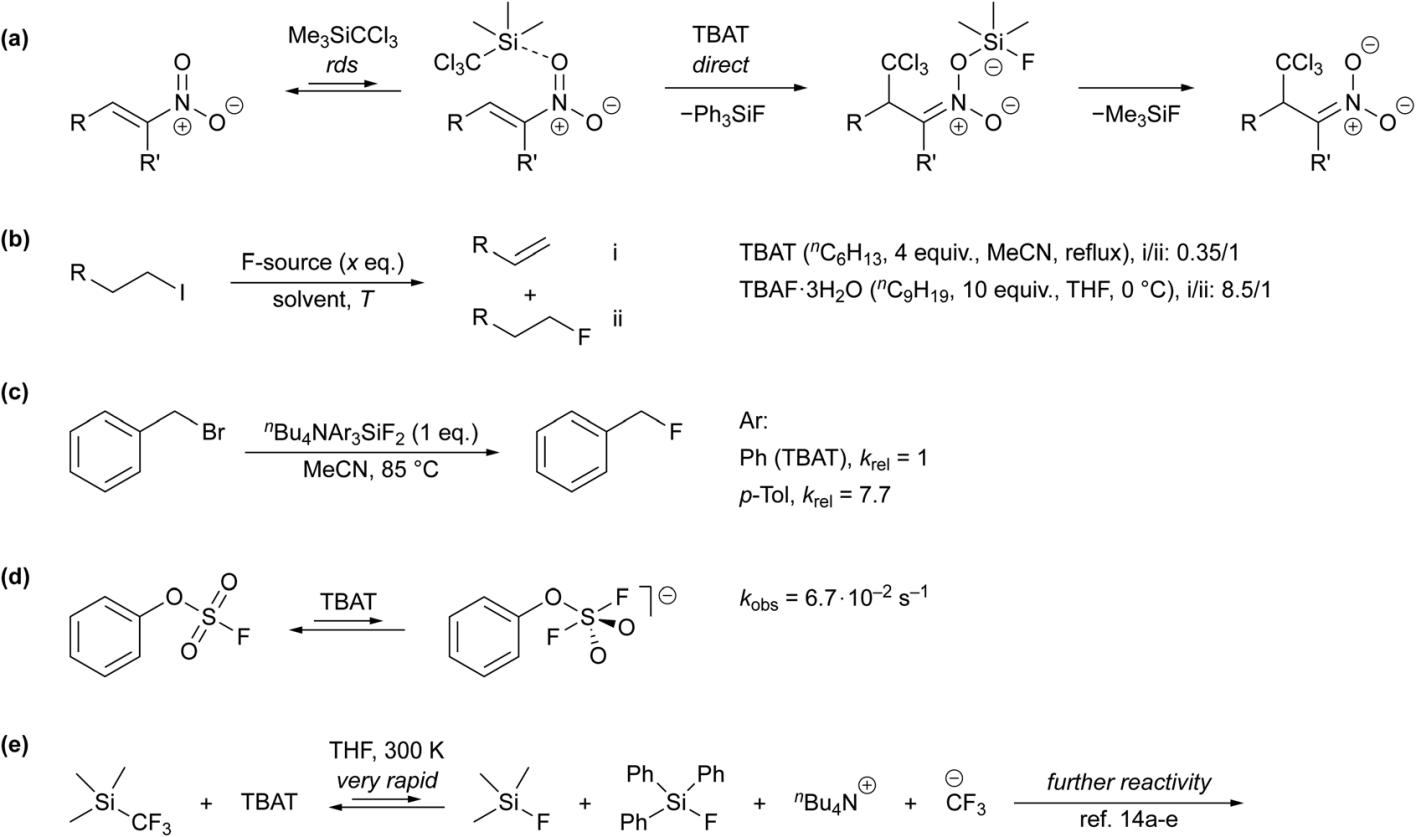
(a) Mechanistic proposal in a study on the addition of TMSCCl_3_ to nitroalkenes,^10^ (b) ratios of β-elimination-to-substitution products in reactions of 1-iodoalkanes with TBAT and TBAF·3H_2_O,^1*a*,11^ (c) relative rate constants of fluorination of benzyl bromide using ^*n*^Bu_4_NAr_3_SiF_2_, where Ar = Ph or *p*-Tol,^12^ (d) rapid fluoride exchange between TBAT and phenyl fluorosulfate studied *via* time-dependent saturation transfer NMR spectroscopy,^13^ (e) prior mechanistic work on TBAT-initiated reactivity of TMSCF_3_, where the fluoride transfer from TBAT is very rapid with respect to NMR spectroscopy. In the presence of excess TMSCF_3_, the CF_3_^−^ anion(oid) is transient, undergoing rapidly-reversible exergonic equilibrium with [TMS(CF_3_)_2_]^−^.^14^

Despite its extensive application, very little has been reported about the kinetics and mechanism by which TBAT transfers fluoride.^7*b*,10–16^ Direct fluoride transfer from TBAT to silicon was proposed for the anion-initiated 1,4-addition of TMSCCl_3_ to nitroalkenes (Scheme 1a).^10^ This conclusion was primarily based on the lack of signals attributable to free fluoride ions in solution-phase ^19^F NMR spectra of the reaction mixture. In the conversion of 1-iodoalkanes to 1-fluoroalkanes, TBAT results in significantly less competing β-elimination than TBAF·3H_2_O (Scheme 1b), albeit under markedly different conditions,^1*a*,11^ again leading to the conclusion that the fluoride transferred from TBAT is much less “naked” than that in TBAF. Mąkosza and Bujok found that the tris-*p*-tolyl analogue of TBAT reacts with benzyl bromide (BzBr) more than seven-fold faster than TBAT itself, Scheme 1c.^12^ However, whether the transfer occurs directly from the silicate was not established. Finally, a recent study by Zheng *et al.* reported a pseudo-first-order rate constant, *k*_obs_ = 6.7 × 10^−2^ s^−1^, for the nominally direct transfer of fluoride from TBAT (180 mm) to the sulfur in phenyl fluorosulfate (PhOSO_2_F, 18 mm) at 298 K in MeCN-*d*_3_ (Scheme 1d). The rate was estimated by time-dependent saturation-transfer NMR spectroscopy.^13^

We have recently studied chain reactions initiated by liberation of a CF_3_^−^ anion(oid) from [TMS(CF_3_)F]^−^. The latter is generated *in situ* by very rapid transfer of fluoride from TBAT to TMSCF_3_, concomitantly producing fluorotriphenylsilane (FTPS), Scheme 1e.^14^ Herein we report on a detailed investigation into the kinetics and mechanism of fluoride transfer from TBAT. We have studied this under three sets of conditions, two involving degenerate fluoride exchange (with FTPS and with 2-naphthalenyl fluorosulfate, ARSF) and one involving fluoride transfer to benzyl bromide (BzBr). The primary focus of the work has been the distinction of whether fluoride is transferred directly between TBAT and the fluoride acceptor, or whether there are pre-dissociation step(s) to liberate TBAF as the transient agent for fluoride delivery.

## Results and discussion

Prior to our NMR-investigation of the kinetics and mechanism of fluoride transfer, we analysed the NMR-spectroscopic features of TBAT, in THF and in MeCN, including its speciation. The latter comprises two aspects: the extent of interaction between the difluorotriphenylsilicate and tetra-*n*-butylammonium ions, Ph_3_SiF_2_^−^ and ^*n*^Bu_4_N^+^, and the extent of fluoride dissociation from the anion, Ph_3_SiF_2_^−^.

### Solution-phase ^1^H,^19^F NMR-spectroscopic parameters of TBAT

The ^1^H and ^19^F nuclei in TBAT were selected for study based on their high abundance and receptivity, 1/2-spin character, and relatively short longitudinal relaxation times. The difluorotriphenylsilicate anion is pseudo-trigonal bipyramidal in both the solid state,^1*a*^ and in solution,^1,17^ with the fluorine atoms axial and the phenyl groups equatorial. ^1^H NMR (400 MHz) spectra of TBAT in THF-*d*_8_ (see Section S2.1 of ESI†) comprise two sets of signals in the aromatic region (*p*-, *m*- and *o*-protons in Ph) and three sets of signals in the aliphatic region (C(1), C(2,3) methylenes and C(4) methyl in ^*n*^Bu). In MeCN-*d*_3_ the three methylene units are all resolved in the aliphatic region, the aromatic region is similar to that in THF. The ^19^F NMR (377 MHz) spectra display a singlet corresponding to the two chemically and magnetically equivalent fluorine atoms in the Ph_3_SiF_2_^−^ anion, with satellites (^1^*J*_SiF_ = (253.8 ± 0.4) Hz and ^2^*J*_FC_ = (41.4 ± 0.3) Hz) that are consistent with the ^29^Si and ^13^C{^1^H} NMR data (both in CDCl_3_) reported by DeShong.^1*b*^ The concentration dependencies of the chemical shift, *δ*^TBAT^, and the longitudinal relaxation time constant, *T*^TBAT^_1_, of the fluorine atoms were correlated empirically, eqn (1)–(4). The chemical shift of TBAT is concentration dependent in THF (varying between −96.8 and −95.3 ppm, in the range studied, eqn (1)), whereas it is essentially invariant in MeCN in the concentration range studied (−95.3 ppm; eqn (3)). This phenomenon may arise from the much lower dielectric constant of THF, compared to MeCN, and thus greater impact of changes in the ionic strength of the medium as the TBAT concentration in THF is raised. The longitudinal relaxation of the ^1^H and ^19^F nuclei in TBAT takes longer in MeCN than in THF, at all concentrations studied, see Sections S3.2 and S3.3 of ESI.†1
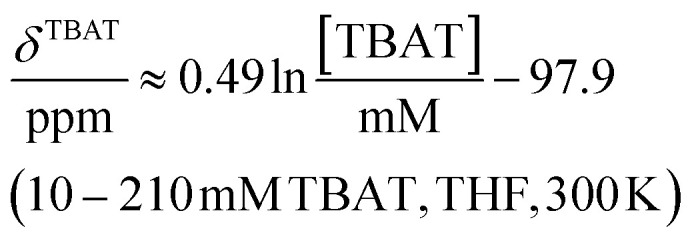
2
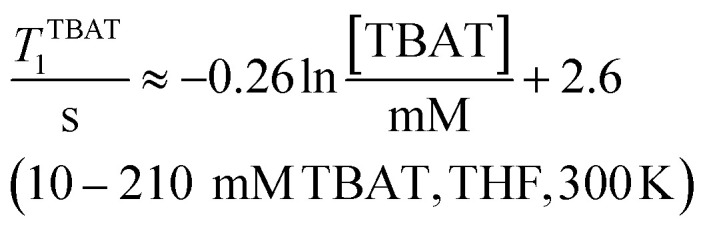
3
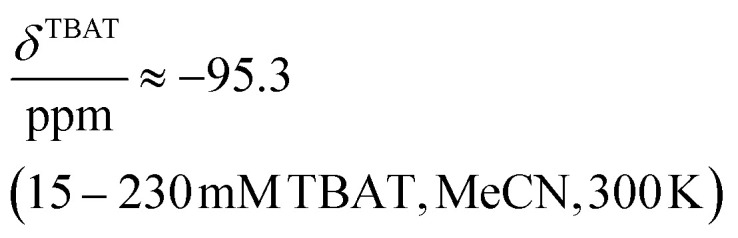
4
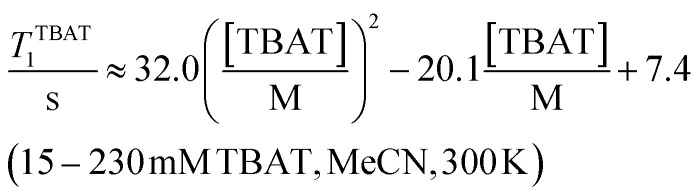


#### Ion pairing

**Fig. 2:**
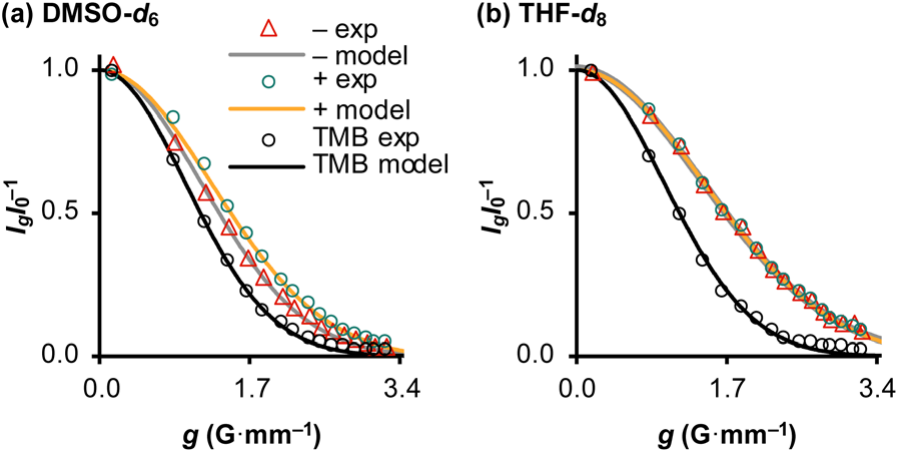
Diffusion profiles of Ph_3_SiF_2_^−^ (“−”), ^*n*^Bu_4_N^+^ (“+”) and the internal diffusion standard (“TMB”) in: (a) 1.5 mm TBAT and 263 mm TMB in DMSO-*d*_6_, and (b) 207 mm TBAT and 252 mm TMB in THF-*d*_8_; both at 300 K.

The translational diffusion (*D*) and mutual proximity (NOESY) of the Ph_3_SiF_2_^−^ and ^*n*^Bu_4_N^+^ ions were used to probe the extent of ion pairing in TBAT, see Section S4.1 of ESI† for details. The relative translational self-diffusion coefficients, *D*^−^/*D*^+^ were determined *via*^1^H pulsed field gradient NMR experiments at three concentrations, in both THF-*d*_8_ and MeCN-*d*_3_, and at three temperatures, Table 1, entries 1–18. The average relative translational self-diffusion coefficient, *D*^−^/*D*^+^, is close to unity in both THF-*d*_8_ (0.987 ± 0.008) and MeCN-*d*_3_, (1.02 ± 0.02). These values indicate either that the ions are strongly paired, or that the separated ions have coincidental diffusive properties. To distinguish these possibilities, we determined translational diffusion coefficients relative to 1,3,5-trimethoxybenzene, TMB, at low concentration in DMSO-*d*_6_, and at high concentration in THF-*d*_8_, Fig. 2a and b. The TMB-normalised coefficients in DMSO-*d*_6_ are not only different to each other, but also significantly larger than in THF-*d*_8_, Table 1, entries 19 and 20. Moreover, the TMB-normalised diffusion coefficient of a reference ^*n*^Bu_4_N^+^ ion liberated from [^*n*^Bu_4_N^+^][B(3,5-(CF_3_)_2_–C_6_H_3_)_4_] in THF-*d*_8_ is very similar to the *D*^+^/*D*^TMB^ value of the TBAT-derived ^*n*^Bu_4_N^+^ ion in DMSO-*d*_6_, Table 1, entries 19 and 21.

**Table 1:** (a) Ratios of translational diffusion coefficients of Ph_3_SiF_2_^−^ and ^*n*^Bu_4_N^+^, *D*^−^/*D*^+^, in solutions of TBAT in THF-*d*_8_ and MeCN-*d*_3_ (entries 1–18). (b) Translational diffusion coefficients of Ph_3_SiF_2_^−^ and ^*n*^Bu_4_N^+^, relative to the internal diffusion standard, TMB, in solutions of TBAT in DMSO-*d*_6_ and THF-*d*_8_ (entries 19 and 20). (c) Translational diffusion coefficient of ^*n*^Bu_4_N^+^, relative to the internal diffusion standard, TMB, in a solution of [^*n*^Bu_4_N^+^][B(3,5-(CF_3_)_2_–C_6_H_3_)_4_] in THF-*d*_8_ (entry 21)

Entry	Solvent	[TBAT] (mm)	*T* (K)	*D* ^−^/*D*^+^
1	THF-*d*_8_	220	300	0.994
2	THF-*d*_8_	220	310	0.984
3	THF-*d*_8_	220	320	0.981
4	THF-*d*_8_	110	300	0.980
5	THF-*d*_8_	110	310	0.973
6	THF-*d*_8_	110	320	1.01
7	THF-*d*_8_	30	300	0.981
8	THF-*d*_8_	30	310	0.980
9	THF-*d*_8_	30	320	1.00
10	MeCN-*d*_3_	220	300	1.04
11	MeCN-*d*_3_	220	320	0.986
12	MeCN-*d*_3_	220	335	0.971
13	MeCN-*d*_3_	120	300	1.04
14	MeCN-*d*_3_	120	320	1.01
15	MeCN-*d*_3_	120	335	0.994
16	MeCN-*d*_3_	40	300	1.03
17	MeCN-*d*_3_	40	320	1.05
18	MeCN-*d*_3_	40	335	1.01
19	DMSO-*d*_6_	1.5	300	1.21
				*D* ^−^/*D*^TMB^ = 0.710
				*D* ^+^/*D*^TMB^ = 0.586
20	THF-*d*_8_	207	300	1.00
				*D* ^−^/*D*^TMB^ = 0.467
				*D* ^+^/*D*^TMB^ = 0.466
21	THF-*d*_8_	201^a^	300	0.931
				*D* ^−^/*D*^TMB^ = 0.536
				*D* ^+^/*D*^TMB^ = 0.576

a [^*n*^Bu_4_N^+^][B(3,5-(CF_3_)_2_–C_6_H_3_)_4_].

^1^H 1D NOESY analysis of 210 mm solutions of TBAT confirmed extensive close-contacts between the ions, in both THF-*d*_8_ and in MeCN-*d*_3_ (see Section S4.2 of ESI† for details). For example, perturbation of selected aromatic protons resulted in extensive inter-ion NOEs at the aliphatic protons, as well as the expected intra-ion NOEs at the residual aromatic protons. The combined analysis of ion-diffusion and ion-proximity in TBAT indicate that Ph_3_SiF_2_^−^ and ^*n*^Bu_4_N^+^ are extensively paired in THF-*d*_8_ and in MeCN-*d*_3_, even at moderately low concentration, and predominantly dissociated in dilute solutions in DMSO-*d*_6_.

#### Exchange of fluoride with TBAT

To the best of our knowledge, the reversible liberation of TBAF from TBAT has not been reported, and TBAF is not detected in standard NMR spectra of solutions of purified TBAT.^1^ However, in such solutions we do detect exchange processes between TBAT and a range of species, including TBAF *vide infra*, using ^19^F chemical exchange saturation transfer (^19^F-CEST) NMR spectroscopy (see Sections S4.3 and S7.1.1 in the ESI†). In one ^19^F-CEST regime, the chemical shift corresponding to a low-concentration undetected spin is selectively pre-saturated to probe for its chemical exchange with a detectable spin present at higher concentration. If the exchange occurs at a rate that is sufficiently large relative to the concentration and longitudinal relaxation time, *T*_1_, of the higher concentration spin, then there is a detectable attenuation in its intensity in the subsequent pulse-acquire 1D NMR spectrum.

The experiments were conducted using concentrated solutions^18^ of TBAT that were prepared and sealed, in J Young valve NMR tubes, in the glove-box. ^19^F-CEST to TBAT was detected when pre-saturating at −170 ppm (FTPS) and at −149/147 ppm in THF/MeCN. The latter species was assigned as tetra-*n*-butylammonium bifluoride, TBABF (*δ*^TBABF^ = −147 ppm, THF and MeCN-*d*_3_).^19,20^ The ^19^F-CEST was greater in MeCN than in THF. When the solutions were stored over 3 Å molecular sieves for 2 months, ^19^F-CEST was supressed to below the detection limit in THF. In MeCN the ^19^F-CEST profile showed exchange with species at −77 ppm, −115 ppm, −128 ppm, −147 ppm (TBABF) and −169 ppm (FTPS). The species at −77 ppm is tentatively assigned as partially-hydrous TBAF (*δ*^TBAF^ = −72 ppm, MeCN-*d*_3_).^19^ The additional signals (at −115 ppm and −128 ppm) possibly arise from co-products of Hofmann elimination, *e.g.*^*n*^Bu_3_N(HF)_*x*_. Lastly, solutions of TBAT containing exogenous FTPS (∼5 mol%)^21^ were studied. The ^19^F-CEST profiles exhibited saturation over a broader frequency range, indicative of rapid exchange between TBAT and FTPS. All other ^19^F-CEST effects were supressed to below the detection limit, indicative that exogenous FTPS drives dissociated fluoride equilibria towards TBAT.

#### Analytical model for the kinetics of TBAT exchange with FTPS

**Scheme 2:**
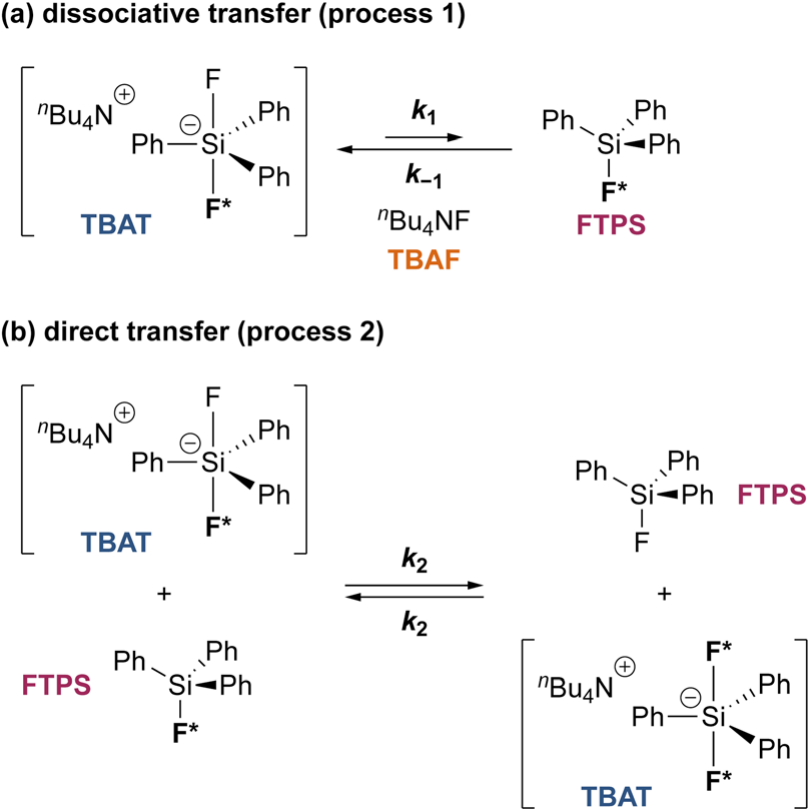
Two limiting pathways of fluoride transfer from TBAT to FTPS. (a) Dissociative transfer *via* TBAF, where *K*_1_ = *k*_1_/*k*_−1_ ≪ 1. (b) Direct bimolecular transfer (the rate constants in each direction, *k*_2_, are identical, as Δ*G*° = 0). In each transfer pathway, examples of exchanging fluorine atoms are marked in bold with an asterisk.

To gain quantitative insight into the exchange processes detected by ^19^F-CEST, we conducted magnetisation transfer NMR-spectroscopic analysis of TBAT solutions, using FTPS as the fluoride acceptor^22^ (see Section S7.1.2 of ESI,† for further discussion). The two limiting pathways for fluoride transfer are outlined in Schemes 2a and 2b. In the dissociative transfer pathway (process 1), endogenous TBAF is the reactive intermediate. The net rate coefficients are *k*_1_ for dissociation of TBAT into FTPS + TBAF, and *k*_−1_ for their recombination to generate TBAT; thus *K*_1_ = *k*_1_/*k*_−1_ ≪ 1. In the direct fluoride transfer pathway (process 2), TBAT undergoes a bimolecular elementary reaction with FTPS. The net rate coefficient for this process is *k*_2_, in both directions (Δ*G*° = 0).^23^

The kinetic models below are derived using a discrete spin formalism,^24^ in which the spin-half nuclei (^19^F) within a large ensemble are opposed (^19^F^∇^) or aligned (^19^F^Δ^) to the +*z*-axis. In this formalism a species ‘*S*’ with *n* equivalent ^19^F nuclei has (*n* + 1) magnetic states: thus, FTPS and TBAF both have two states, while TBAT has three. The populations (*N*) of ^19^F^∇^ and ^19^F^Δ^ in each species (*N*_∇_ and *N*_Δ_) dictate its fractional magnetisation, *m*^*S*^, eqn (5), and this is readily correlated with the integral of the measured NMR signal (*M*_*z*_^*S*^, eqn (6)).5
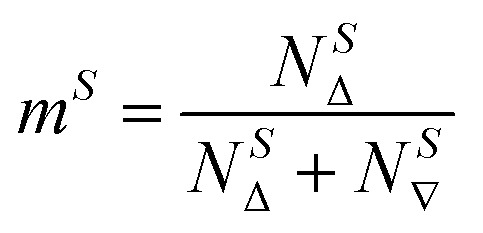

6 *M*_*z*_^*S*^ = *M*_*z*,eq_^*S*^(2*m*^*S*^ − 1)

At equilibrium, the fractional magnetisation is unity (*m*_eq_^*S*^ = 1) and when fully inverted, it is zero (*m*^*S*^ = 0). Rate laws that describe the change in fractional magnetisation (d*m*/d*τ*) can be derived using the spin formalism, with a steady-state approximation applied to the magnetisation of TBAF. The rate laws for *m*^TBAT^ and *m*^FTPS^ can then be solved analytically to give their temporal magnetisation when undergoing dissociative and/or direct transfer (and longitudinal relaxation), eqn (7) and (8), see Section S5.2.1 of ESI† for full derivation.

7 *m*^TBAT^ = *c*_1_e^(*β*+*γ*)*τ*^ + *c*_2_e^(*β*−*γ*)*τ*^ + 1

8 *m*^FTPS^ = *c*_3_e^(*β*+*γ*)*τ*^ + *c*_4_e^(*β*−*γ*)*τ*^ + 1

where:9
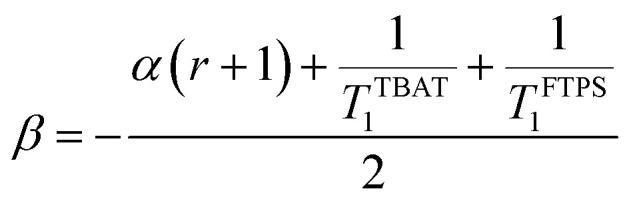
10
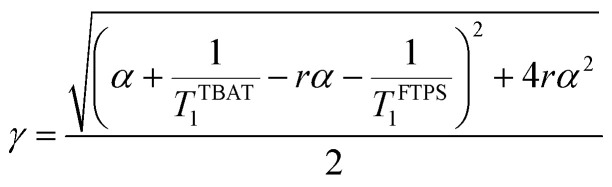
11
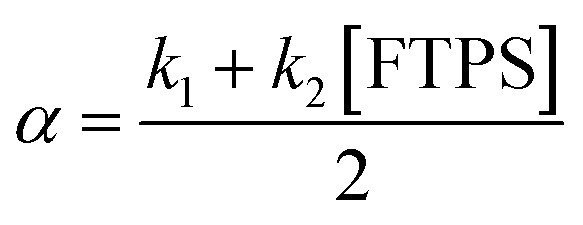
12
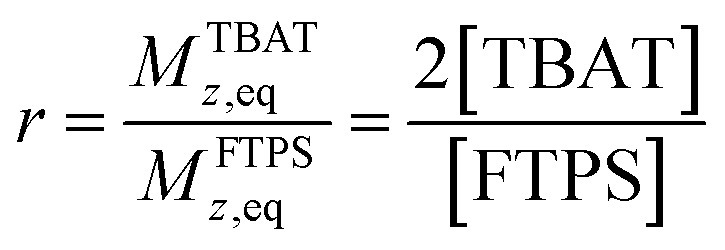


The coefficients for these equations are defined below, in which *m*^TBAT^_0_ and *m*^FTPS^_0_ are fractional magnetisations of the spins at *τ* = 0, the starting point of the relaxation occurring under the spin-formalism.13
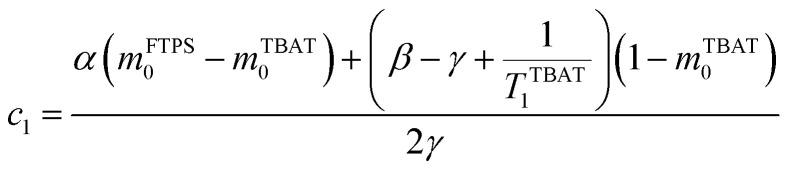
14
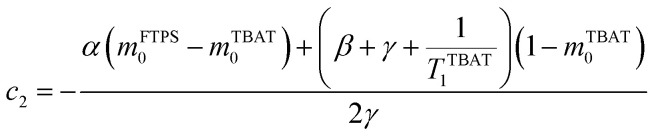
15
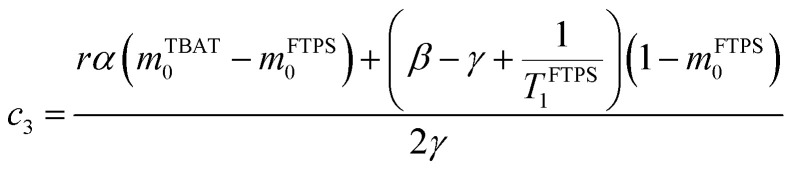
16
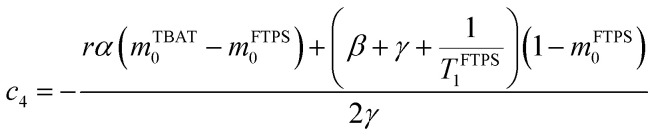


#### Magnetisation transfer in THF and condition-sensitivity

At 300 K in THF the ^19^F longitudinal relaxation of FTPS, *T*^FTPS^_1_ = 12.0 s, is concentration independent and significantly longer than that of TBAT. When the two species were mixed ([TBAT] = 52.5 mm, [FTPS] = 83.9 mm), both ^19^F spins relaxed with equal rates (*T*^obs^_1_ = (2.45 ± 0.02) s), after non-selective inversion. This value is very close to the weighted combination, *T*^calc^_1_ = 2.54 s, of the separated spins (see eqn (S5.51) in Section S5.2.1 of ESI†) and indicative of rapid exchange of ^19^F at the longitudinal relaxation timescale.^25^ This allows use of a simplified kinetic model, eqn (17), to analyse the temporal fractional magnetisation of TBAT after selective inversion of FTPS, and thus extract *α*(*r* + 1).17
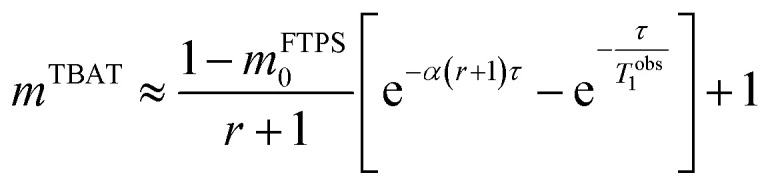


**Fig. 3:**
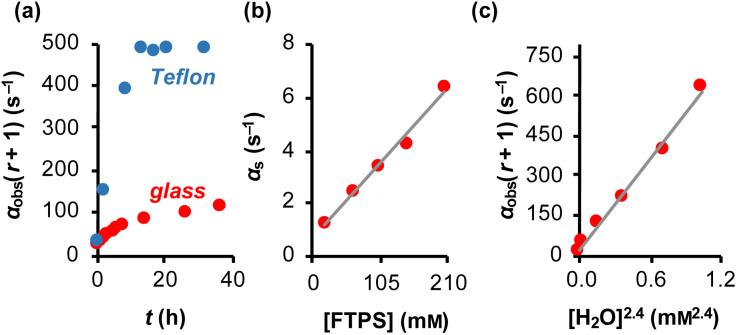
Magnetisation transfer between TBAT and FTPS in THF (at 300 K). (a) An inherent increase of the magnetisation transfer rate with time in reactions conducted inside a dry and sealed glass NMR tube, and inside a dry Teflon insert (located within the NMR tube). (b) Under anhydrous, stabilised, non-acidic conditions, TBAT transfers fluoride to FTPS *via* parallel direct and dissociative mechanisms, with 
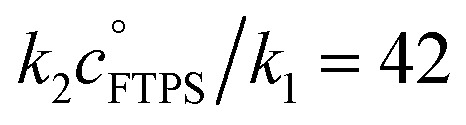
. *α*_s_/s^−1^ = 0.0273[FTPS/mM] + 0.656, *R*^2^ = 0.989. (c) Exchange between the species is catalysed by water, with the order in water estimated as approximately 2. α_obs_(*r* + 1)/s^−1^ = 577[H_2_O/mM]^2.4^ + 17.2, *R*^2^ = 0.989.

The magnitude of *α*(*r* + 1) depends on the rate coefficients of exchange, *k*_1_ and *k*_2_, and the TBAT and FTPS concentrations. However, the experimental values are sensitive to the conditions of sample preparation,^26^ and found to inherently increase with time.^27^ Conducting the reaction inside a dry Teflon insert located within a sealed NMR tube substantially exacerbated the problem, Fig. 3a. A range of tests were conducted to find additives which would afford temporal stability by sequestration of the unidentified acids/ions that were accelerating fluoride exchange between TBAT and FTPS. This eventually led to the use of a combination of 2,6-di-*tert*-butylpyridine (DTBP), as a hindered base, with 3 Å molecular sieves (3 Å-MS), as a passive dehydrating agent. DTBP and 3 Å-MS were not effective in isolation, see Section S5.2.4 of ESI† for further discussion. This allowed us to systematically explore the kinetics of the fluoride transfer from TBAT to FTPS under stabilised, anhydrous and non-acidic conditions,^28^ in THF.

#### Exchange pathways in the temporally-stabilised system

Solutions comprising TBAT (101 mm), FTPS (22.6, 62.9, 101, 144, and 204 mm), DTBP (20 mm) and 1-fluoronaphthalene (internal standard) in THF were sealed in NMR tubes preloaded with 3 Å-MS beads. The exchange rates were then measured periodically by magnetisation transfer over a period of two weeks. The kinetic model, eqn (17), gave excellent fits to all 50 experimental datasets (the average RMSE/*M*^TBAT^_*z*,eq_ = 1%). The resulting plots of *α*_obs_ against *t* were fitted to an empirical exponential decay, eqn (18), to allow evaluation of the underlying temporally-stabilised exchange rate, *α*_s_, see Table S5.10 in Section S5.2.5 of ESI† for fitted parameters and estimated errors.

18 *α*_obs_ = (*α*_0_ − *α*_s_)e^−*k*_s_*t*^ + *α*_s_

Evaluation of *α*_s_ as a linear function of [FTPS], eqn (11), allows the rate coefficients for the two pathways to be determined as *k*_1_ = 1.3 s^−1^ and *k*_2_ = 55 m^−1^ s^−1^, Fig. 3b. Thus, under anhydrous, stabilised, non-acidic conditions in THF, TBAT transfers fluoride to FTPS *via* parallel direct and dissociative mechanisms, with 
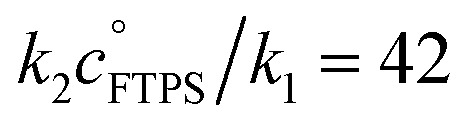
. Analysis of the non-stabilised anhydrous system indicated that the dissociative pathway is again a significant contributor to fluoride transfer from TBAT at low acceptor concentrations, see Sections S5.2.6 and S7.2.3 of ESI† for experimental details and mathematical considerations. The rate of ^19^F transfer from TBAT to FTPS in THF is markedly accelerated by exogenous water which may assist fluoride dissociation from TBAT *via* H-bonding or autoionisation.^29^ Analysis of *α*_obs_(*r* + 1) against [H_2_O], Fig. 3c, indicates that this predominantly involves interaction with a water dimer, or sequential reaction with two water molecules (see Section S5.2.4 of ESI†).

The exchange between non-stabilised TBAT and FTPS at 300 K in MeCN was found to be significantly more rapid than in THF. Indeed, the initial fractional magnetisation of TBAT (*m*^TBAT^_0_) was less than unity due to non-negligible exchange with FTPS during the 1.3 ms selective inversion pulse. In freshly prepared samples, a statistical distribution of ^19^F spins was achieved within 5 ms, and the exchange rate again increased with time. Use of DTBP + 3 Å-MS afforded a four-fold decrease in the initial magnetisation transfer rate in freshly prepared samples, accompanied by reduced line broadening, *e.g.*, the ^29^Si satellites could be detected (see Fig S5.22c and S5.24a in Section S5.2.7 of ESI† for the appearance of the signals in the absence and in the presence of DTBP + 3 Å-MS, respectively). However, the stabilisation was short-lived and unsuitable for detailed kinetic interrogation. We thus switched to the use of less-reactive fluoride acceptors to facilitate study of the kinetics of transfer from TBAT in MeCN, *vide infra*.

### Aryl fluorosulfate ^19^F-exchange with TBAT in MeCN and inhibition by exogenous FTPS

**Fig. 4:**
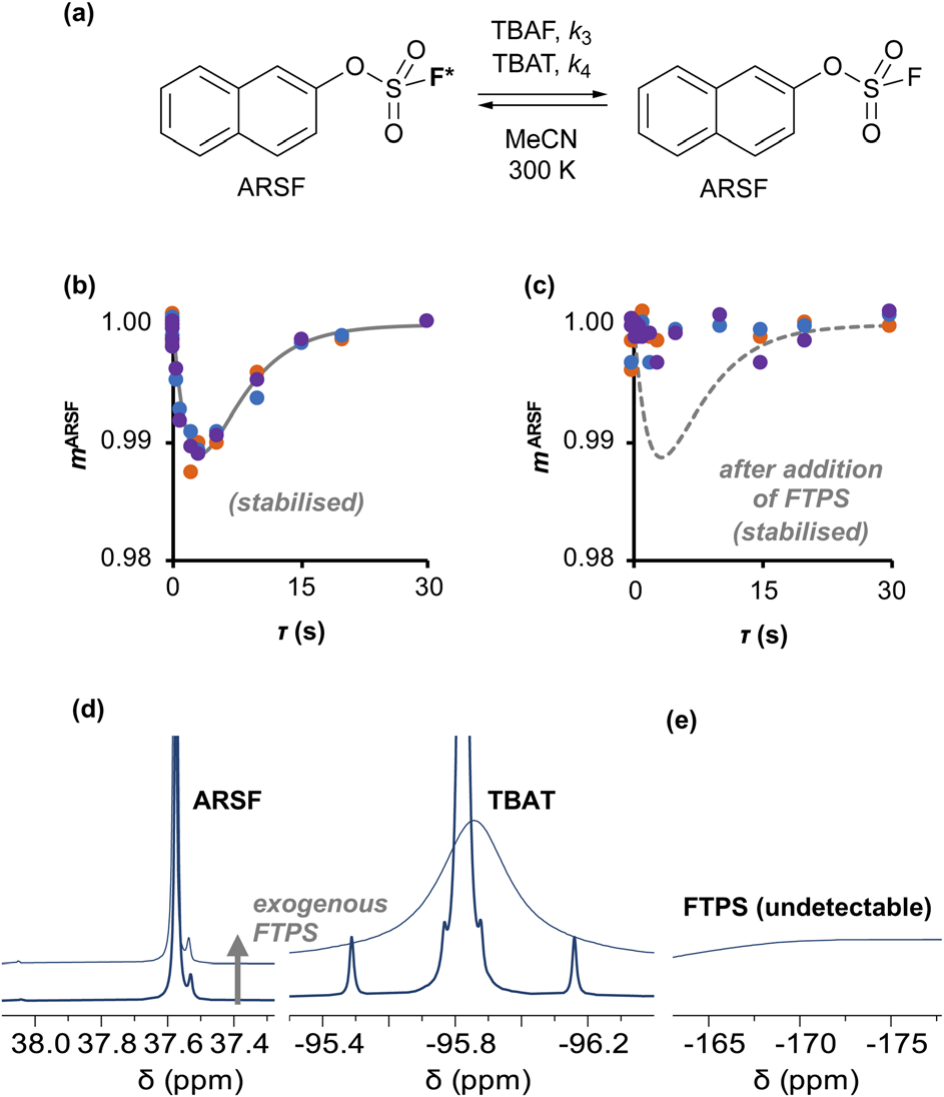
(a) An approximation for concerted fluoride transfer between TBAT and ARSF in MeCN (at 300 K; exchanging fluorine atoms are marked with an asterisk for clarity). (b) Magnetisation transfer between the spins in MeCN; first measurement, prior to stabilisation, not shown. Solid line show fitted model. (c) Exchange inhibited by addition of a small amount of exogenous FTPS to the solution; dashed line shows fitted model from (b) for reference. (d) The ^19^F NMR signal of ARSF does not exhibit line broadening upon addition of exogenous FTPS, unlike that of TBAT. (e) The FTPS signal is too broad to detect.

Zheng *et al.* reported on exchange between aryl fluorosulfates and several fluoride sources, including TBAT in MeCN.^13^ They proposed that the ‘SuFEx’ process proceeds *via* an endergonic equilibrium association of fluoride at sulfur. To test for dissociative *versus* direct transfer pathways (processes 3 and 4, respectively; Fig. 4a), we selected 2-naphthalenyl fluorosulfate (ARSF) and derived a kinetic model for its magnetisation after selective inversion of TBAT, eqn (19); see Section S5.3.1 of ESI† for the derivation.19
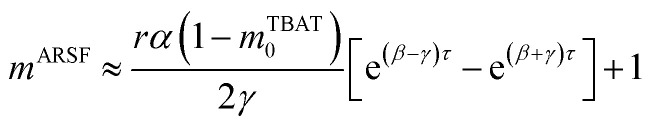
where:20
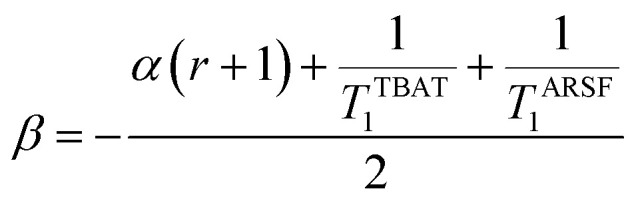
21
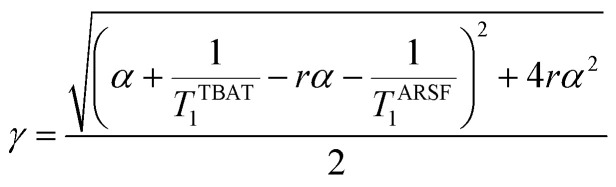
22
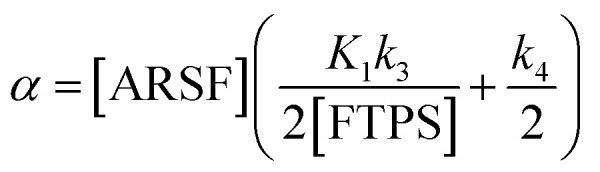
23
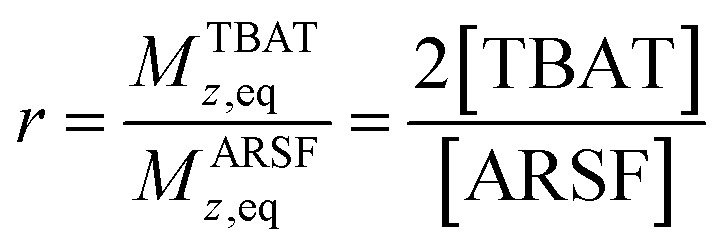


Exchange was detected at 300 K and four magnetisation transfer measurements were performed on the same sample over a period of 3 hours immediately after its preparation (188 mm TBAT; 52 mm ARSF, MeCN; Section S5.3.3 of ESI†), Fig. 4b. The rate of exchange initially decreased then became stable and the kinetic model, eqn (19), correlated well with the experimental datasets, affording an average (stabilised) exchange rate constant *α*_s_ = 1.4 × 10^−3^ s^−1^.

In the above experiment the endogenous FTPS is at very low concentration (*K*_1_ ≪ 1). However, as evident from eqn (22), while FTPS does not affect the direct transfer pathway (*k*_4_; TBAT), *α* is a decreasing function of [FTPS] for the dissociative pathway (*k*_3_; TBAF). Magnetisation transfer measurements were thus repeated on the sample after addition of FTPS, ∼4 mm. The rate of ^19^F-transfer between TBAT and ARSF was inhibited to below the qualitative detection limit, Fig. 4c; individual *m*^ARSF^ profiles before and after FTPS addition are presented in Fig. S5.28 in Section S5.3.3 of ESI.† Fitting the data to eqn (19) gave an average value of *α*_s_ = 8.1 × 10^−5^ s^−1^ across all four runs, *i.e.*, 94% inhibition of the rate of fluoride transfer. Furthermore, comparison of the ^19^F NMR spectra before and after addition of FTPS (Fig. 4d) showed that while the ARSF signal was unaffected, the TBAT signal exhibited very significant line broadening, and the FTPS signal itself was broadened to an extent which rendered it undetectable, Fig. 4e. These features indicate that fluoride exchange between TBAT and aryl fluorosulfates is predominantly *via* the dissociative pathway, with an estimated standard state partitioning 
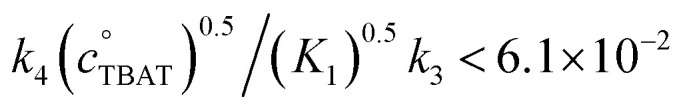
 for 2-naphthalenyl fluorosulfate (ARSF).

#### TBAT-mediated aryl fluorosulfate decomposition

**Fig. 5:**
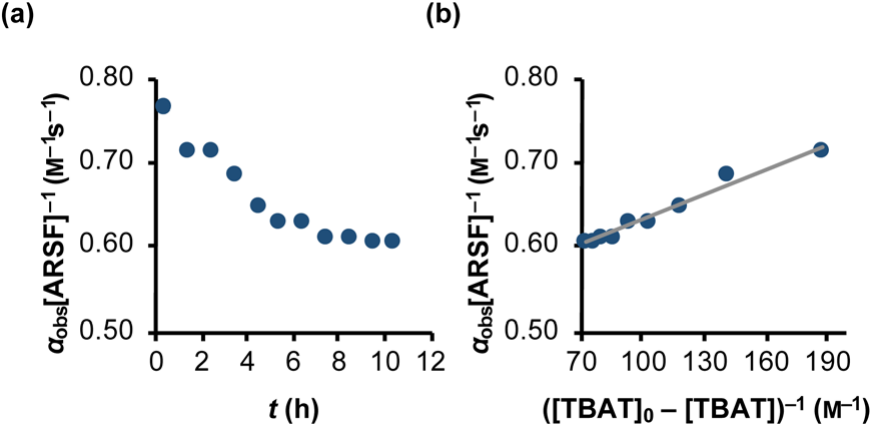
Temporal evolution of the rate of magnetisation transfer between TBAT and ARSF in MeCN (at 335 K). (a) Exchange between the spins is progressively inhibited due to accumulating FTPS, formed from decomposing TBAT, indicating at least partially dissociative character to the exchange. (b) Fitting of the model of eqn (24) showed that fluoride is transferred to ARSF *via* both dissociative and direct transfer pathways, with *k*_3_/*k*_4_ = 43. *α*_obs_[ARSF]^−1^/M^−1^ s^−1^ = 1 × 10^−3^ ([TBAT]_0_-[TBAT])^−1^ /M^−1^ + 0.53; *R*^2^ = 0.974.

Rapid defluorosulfation of phenyl fluorosulfate by nominally anhydrous TBAF was reported by Zheng *et al.*^13^ We detected analogous decomposition of ARSF by TBAT in MeCN at 335 K, as indicated by the growth of two low intensity signals identified as [FSO_2_O^−^][^*n*^Bu_4_N^+^] and SO_2_F_2_.^30^ The third by-product of the decomposition is nominally FTPS, but this is not observed in the spectra, even on cooling to 233 K, possibly due to the rapid exchange line-broadening.^31^ However, a series of magnetisation transfer measurements (see Section S5.3.4 of ESI† for details) showed a progressive decrease in the rate of ^19^F exchange between TBAT and ARSF, arising from the impact of the growing FTPS concentration on the dissociative pathway, eqn (24) and Fig. 5a.24
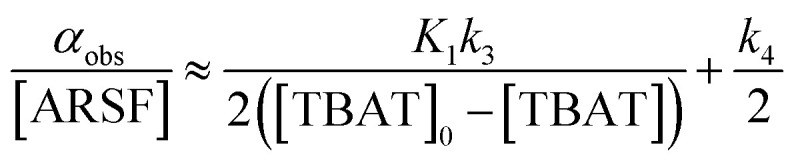


The experimental data corresponding to *α*_obs_, [ARSF], and [TBAT] was fitted against eqn (24) and Fig. 5b, to estimate *k*_3_/*k*_4_ = 43, and thus that TBAF is a significantly more potent direct fluoride donor than TBAT.

### FTPS inhibition of the reaction of benzyl bromide with TBAT

**Fig. 6:**
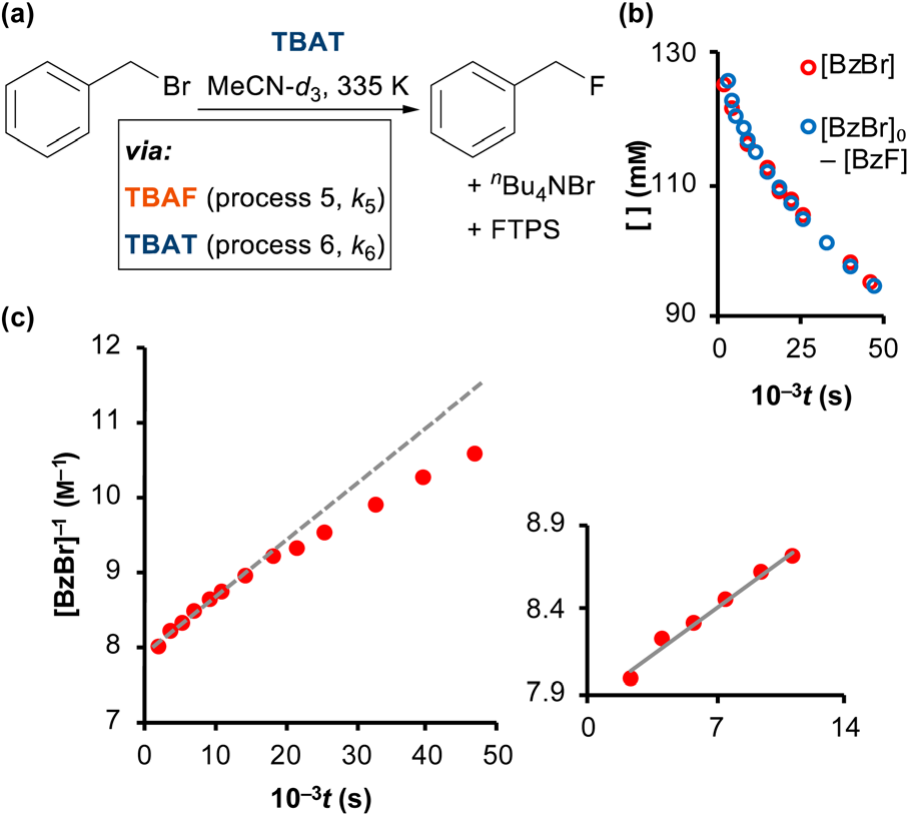
(a) Fluorination of benzyl bromide with TBAT in MeCN-*d*_3_ (at 335 K). (b) The reaction was followed by ^1^H and ^19^F NMR spectroscopy, and the decay of BzBr mirrored the growth of BzF. (c) The reaction exhibits progressive inhibition, see dashed line, due to accumulating FTPS. Inset shows linear second-order correlation over initial 30% conversion: 1/[BzBr]/M^−1^ = 9 × 10^−5^*t*/s + 1/[BzBr]_0_/M^−1^; *R*^2^ = 0.985.

As reported by Mąkosza and Bujok, TBAT reacts with benzyl bromide (BzBr) at elevated temperatures in MeCN, Fig. 6a.^12^ However, unlike the degenerate exchange processes explored thus far, *vide supra*, the process stoichiometrically co-generates FTPS. Thus, fluoride transfer to BzBr that proceeds *via* a dissociative pathway (process 5) will undergo progressive inhibition by FTPS. Conversely, if the fluoride transfer proceeds predominantly, or exclusively, *via* direct transfer from the difluorotriphenylsilicate anion (process 6), then there should be no significant inhibition by accumulating, or exogenous, FTPS. The reaction of [TBAT]_0_ = 129 mm with [BzBr]_0_ = 131 mm was readily analysed *in situ* by ^1^H/^19^F NMR spectroscopy at 335 K in MeCN-*d*_3_, under N_2_; with the decay in BzBr mirroring the growth of BzF, Fig. 6b; see Section S6.1 of ESI† for details.

Standard graphical analysis, Fig. 6c, afforded an initial pseudo second-order rate coefficient of 9 × 10^−5^m^−1^ s^−1^. The curvature in the reciprocal plot is consistent with progressive inhibition of the reaction. Graphical analysis of an identical reaction conducted in the presence of one equivalent of exogenous FTPS (140 mm), afforded a pseudo second-order rate coefficient of 4 × 10^−5^m^−1^ s^−1^, and without any evident progressive inhibition (see Section S6.2 of ESI†). These results indicate that the reaction of TBAT with BzBr at 335 K in MeCN initially proceeds with significant flux *via* both the dissociative and direct transfer pathways.

## Conclusions

The speciation of tetra-*n*-butylammonium difluorotriphenylsilicate ([Ph_3_SiF_2_^−^][^*n*^Bu_4_N^+^], TBAT) and the mechanism of its reaction with three fluoride acceptors has been studied in detail by a range of ^1^H/^19^F NMR-spectroscopic and kinetic methods. A combination of ^1^H 1D NOESY and ^1^H diffusion analysis showed the Ph_3_SiF_2_^−^ and ^*n*^Bu_4_N^+^ ions to be strongly paired in THF-*d*_8_, and in MeCN-*d*_3_, but separated in DMSO-*d*_6_. A series of ^19^F CEST NMR experiments identified that the ion-pairs undergo endergonic interconversion with fluorotriphenylsilane (FTPS) and tetra-*n*-butylammonium fluoride (TBAF), both of which are below the detection limit in standard ^19^F pulse-acquire NMR spectra. TBAF undergoes further equilibria and decomposition leading, *inter alia*, to the formation of tetra-*n*-butylammonium bifluoride (TBABF).

The kinetics of degenerate fluoride transfer from TBAT to FTPS, and to 2-naphthalenyl fluorosulfate (ARSF), were then studied by ^19^F magnetisation transfer. The rate of exchange between TBAT and FTPS in THF is much more rapid than the longitudinal relaxation timescale of the spins, increases with time, and is profoundly accelerated by traces of water. Addition of a combination of 2,6-di-*tert*-butylpyridine (DTBP) and 3 Å molecular sieves (3 Å-MS) to the samples prepared in gas-tight sealed NMR tubes under N_2_ afforded a stabilised, anhydrous, non-acidic medium suitable for detailed and systematic kinetic analysis. The exchange rates were analysed by magnetisation transfer at various concentrations of FTPS, and the kinetics characterised using a model that includes direct (*k*_2_) transfer from [Ph_3_SiF_2_^−^] and indirect transfer (*k*_1_) *via* TBAF. The standard state partitioning was estimated to be 
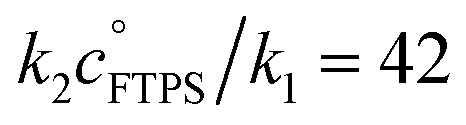
, and thus at 1 m FTPS the transfer is near-exclusively direct (>97%), while at 1 mm FTPS it is near-exclusively dissociative (>95%). The analogous system in MeCN undergoes very rapid fluoride exchange, approaching the limit of practicality of the measurement method, and is only transiently stabilised by 2,6-di-*tert*-butylpyridine and 3 Å molecular sieves.

Exchange of fluoride between TBAT and ARSF was analysed by magnetisation transfer in MeCN at 300 K, again using a model that includes direct (*k*_4_) transfer from [Ph_3_SiF_2_^−^] and indirect transfer (*k*_3_) *via* TBAF. The exchange rate is very much slower than between TBAT and FTPS, and approaches the longitudinal relaxation timescale of the spins. Moreover, the addition of FTPS (4 mm) results in the rate of transfer between TBAT and ARSF being attenuated to the limits of detection. The standard state partitioning was estimated to be 
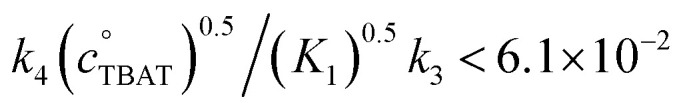
, and thus the direct exchange pathway would only become favoured over the dissociative pathway at unachievable TBAT concentrations (>67 M). On heating to 335 K the system undergoes slow decomposition, resulting in co-generation of FTPS, [FSO_2_O^−^][^*n*^Bu_4_N^+^] and SO_2_F_2_, and gradual inhibition of the rate of fluoride exchange between TBAT and ARSF. The reaction of benzyl bromide (BzBr) with TBAT in MeCN-*d*_3_ at 335 K proceeds with non-degenerate fluoride transfer and is progressively and exogenously inhibited by FTPS, again due to competing dissociative and direct transfer mechanisms. The *initial* standard state partitioning in this case is estimated to be 
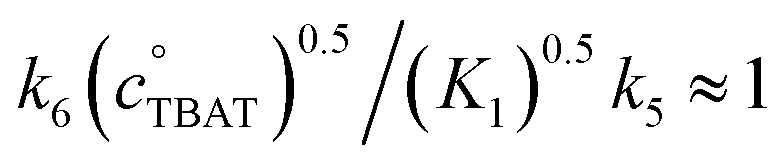
.

**Scheme 3:**
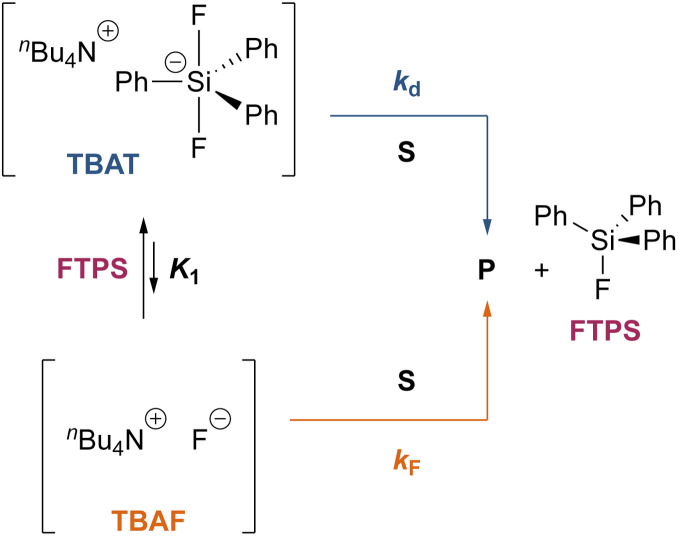
Generic processes involving a substrate ‘*S*’ undergoing stoichiometric reaction with TBAT to generate product ‘*P*’ *via* direct (*k*_d_) and dissociative (*k*_F_) pathways.

Overall, the investigation shows that both dissociative and direct pathways contribute to fluoride transfer from TBAT to fluoride acceptors in THF, and in MeCN. The rate of transfer, and the pathway partitioning, are strongly dependent on the solvent, the presence of water, the affinity of the substrate to fluoride, and the concentrations of TBAT, the substrate and FTPS. The most common application of TBAT is for stoichiometric non-degenerate fluoride transfer. Under these conditions, the reaction efficiency (rate) and selectivity (*e.g.*, addition *versus* elimination) will be dependent on the pathways and their partitioning. The situation can be generalised for a substrate, ‘*S*’, undergoing stoichiometric reaction with TBAT *via* direct (*k*_d_) or dissociative (*k*_F_) pathways, to generate product, ‘*P*’, plus FTPS, Scheme 3 (see Section S7.2.4 of ESI†). Since *K*_1_ is small, soon after the start of reaction the rate and pathway fractionations (*f*_d_ and *f*_F_) can be approximated by eqn (25) and (26), respectively.25
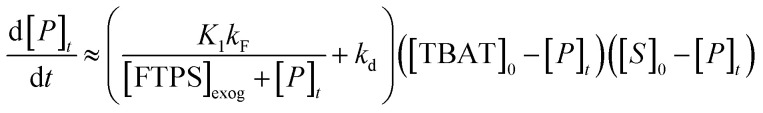
26
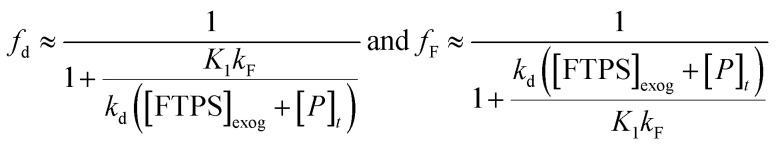


For systems where the direct pathway is desired (*f*_d_ ≫ *f*_F_), then high substrate concentrations, together with the use of exogenous FTPS, will be beneficial. Conversely, where the dissociative pathway is desired (*f*_F_ ≫ *f*_d_), then low concentrations of both substrate and TBAT will be beneficial, albeit at the cost of significantly attenuated rate.

## Data availability

Experimental data, NMR spectra, derivation of kinetic models, and further discussion and analysis can be found in the ESI.†

## Author contributions

MMK conducted the experimental work. MMK and GCL-J analysed the data. All authors contributed to the preparation of the manuscript.

## Conflicts of interest

There are no conflicts to declare.

## Supplementary Material

SC-015-D3SC05776C-s001
