# Supplementary material for: Speciation and kinetics of fluoride transfer from tetra-n-butylammonium difluorotriphenylsilicate (‘TBAT’)
Source: Chem Sci. 2023 Dec 7;15(12):4331–40. doi: 10.1039/d3sc05776c (PMC10952091; doi:10.1039/d3sc05776c)
Supplement: SC-015-D3SC05776C-s001 [file SC-015-D3SC05776C-s001.pdf]

Electronic Supplementary Material (ESI) for Chemical Science.
This journal is © The Royal Society of Chemistry 2023

Supporting information

Speciation and kinetics of fluoride transfer from tetra-*n*-butylammonium difluorotriphenylsilicate (‘TBAT’)

Maciej M. Kucharski,[a] Allan J. B. Watson [b] and Guy C. Lloyd-Jones *[a]

[a.] *School of Chemistry, University of Edinburgh, David Brewster Road, Edinburgh, EH9 3FJ, U.K.*
[b.] *School of Chemistry, University of St Andrews, North Haugh, St Andrews, KY16 9ST, U.K.*

Email: guy.lloyd-jones@ed.ac.uk

Contents

S1. General information 5

S1.1. Reagents and solvents 5

S1.2. Sample preparation 6

S1.3. NMR spectroscopy 7

S1.4. Data analysis and model fitting 8

S2. Solution-phase NMR spectroscopy of TBAT 8

S2.1. Assignment of ^{1}H and ^{13}C{^{1}H} NMR spectra 8

S2.2. ^{19}F NMR spectra 14

S3. Longitudinal relaxation time measurements 16

S3.1. General information 16

S3.2. ^{1}H longitudinal relaxation time measurements 17

S3.2.1. Tetra-*n*-butylammonium difluorotriphenylsilicate (TBAT) 17

S3.2.2. Tetra-*n*-butylammonium tetrakis[3,5-bis(trifluoromethyl)phenyl]borate (TBABARF) 23

S3.3. ^{19}F longitudinal relaxation time measurements 24

S3.3.1. Tetra-*n*-butylammonium difluorotriphenylsilicate (TBAT) 24

S3.3.2. Fluorotriphenylsilane (FTPS) 31

S3.3.3. 2-Naphthalenyl fluorosulfate (ARSF) 33

S4. Speciation of TBAT 36

S4.1. ^{1}H diffusion analysis 36

S4.1.1. Relative diffusion coefficients of $Ph_3SiF_2^-$ and $^{n}Bu_4N^+$ 37

S4.1.2. Diffusion coefficients of $Ph_3SiF_2^-$ and $^{n}Bu_4N^+$, relative to internal standard 40

S4.1.3. Diffusion coefficients of $BARF^-$ and $^{n}Bu_4N^+$, relative to internal standard 41

S4.2. ^{1}H 1D NOESY 42

S4.3. ^{19}F chemical exchange saturation transfer (CEST) 44

S5. Magnetisation transfer NMR spectroscopy 49

S5.1. General information 49

S5.2. TBAT/FTPS 50
S5.2.1. Kinetic model 50
S5.2.2. Magnitude of exchange rate between TBAT and FTPS in THF 62
S5.2.3. Initial titrations of TBAT with FTPS in THF 64
S5.2.4. Condition-sensitivity and temporal instability of the system in THF 68
S5.2.5. Effect of [FTPS] on exchange rate in the stabilised system in THF 78
S5.2.6. Proof of dissociative pathway in the non-stabilised system in THF 86
S5.2.7. Exchange in MeCN 88
S5.3. TBAT/ARSF 92
S5.3.1. Kinetic model 92
S5.3.2. ^{19}F selective inversion-recovery in THF 99
S5.3.3. Selective inversion-recovery in MeCN and inhibition by exogenous FTPS ... 101
S5.3.4. Temporal profile 105
S6. Fluorination of benzyl bromide 111
S6.1. Fluorination of BzBr in the absence of exogenous FTPS 111
S6.2. Fluorination of BzBr in the presence of exogenous FTPS 113
S7. Appendices 115
S7.1. Principles of magnetisation transfer experiments 115
S7.1.1. Chemical exchange saturation transfer (CEST) 115
S7.1.2. Magnetisation transfer *via* selective inversion 116
S7.1.3. Selective pulses 120
S7.2. Mathematics 121
S7.2.1. Relaxation rate constants of TBAT$^{▼▼}$ and TBAT$^{▼▲}$, relative to T_1^{TBAT} 121
S7.2.2. Derivation of constants c_1–c_4 122
S7.2.3. Dissociative pathway in the non-stabilised TBAT/FTPS system 123
S7.2.4. Stoichiometric reaction between substrate ‘S’ and TBAT 125
S7.3. Water content 127
S7.3.1. FTPS 127
S7.3.2. THF and MeCN 128

S8. References.. 129

S1. General information

S1.1. Reagents and solvents

Reagents were obtained from commercial sources (tetra-*n*-butylammonium difluorotriphenylsilicate, TBAT, and 2-naphthalenyl fluorosulfate, ARSF, from Sigma-Aldrich; fluorotriphenylsilane, FTPS, from Fluorochem; benzyl bromide, BzBr, from Sigma-Aldrich; sodium tetrakis[3,5-bis(trifluoromethyl)phenyl]borate, NaBARF, from Thermo Scientific; tetra-*n*-butylammonium chloride, TBACl, from VWR International). TBAT, ARSF, FTPS and BzBr were stored in a nitrogen-filled glovebox (MBraun) and used as received. NaBARF and TBACl were stored under ambient conditions and used as received. Tetra-*n*-butylammonium tetrakis[3,5-bis(trifluoromethyl)phenyl]borate, TBABARF, was prepared by ion metathesis of NaBARF and TBACl in water,[S1] dried *in vacuo* and stored in the glovebox.

^{19}F NMR internal standards were obtained from commercial sources (1-fluoronaphthalene, NAPH, from Fluorochem; fluorobenzene, PhF, from Sigma-Aldrich), distilled, degassed (freeze-pump-thaw, five cycles) and stored in the glovebox under activated 3 Å molecular sieves (3Å-MS) for at least 72 hours prior to use. ^{1}H NMR internal standard, 1,3,5-trimethoxybenzene (TMB), was obtained from a commercial source (Sigma-Aldrich), stored in the glovebox and used as received.

Tetrahydrofuran (THF; anhydrous and inhibitor-free) was dispensed from a solvent purification system (MBraun SPS-800, equipped with alumina columns under positive pressure of argon) fitted inside the glovebox and stored in the glovebox under activated 3Å-MS for at least 72 hours prior to use.[S2] Acetonitrile (MeCN; anhydrous) was obtained from a commercial source (Sigma-Aldrich) and stored in the glovebox under activated 3Å-MS for at least 72 hours prior to use.[S2]

Deuteriated solvents were obtained from commercial sources (THF-d_8, 99.5% D, and MeCN-d_3, 99.8% D, from Cambridge Isotope Laboratories; DMSO-d_6, 99.8% D, from VWR International) and stored in the glovebox under activated 3Å-MS for at least 72 hours prior to use.[S2]

Deionised water (RO grade) was sparged by nitrogen for 12 hours and used immediately.

Molecular sieves (3 Å beads, 1–2 mm, 3Å-MS) were obtained from a commercial source (Alfa Aesar), activated by heating in a microwave oven for 30 seconds, followed by cooling to room temperature *in vacuo* (five heating-cooling cycles), and stored in the glovebox.

Additives to solutions used in temporal stability experiments (other than 3Å-MS) were either obtained from commercial sources (2,6-di-*tert*-butylpyridine, DTBP, from Acros Organics;

hexamethyldisiloxane, TMS_2O, from Sigma-Aldrich), stored in the glovebox and used as received; or prepared in-house (powdered glass, prepared by grinding a Fisher Scientific borosilicate glass Pasteur pipette; Figure S1.1) and stored in the glovebox.

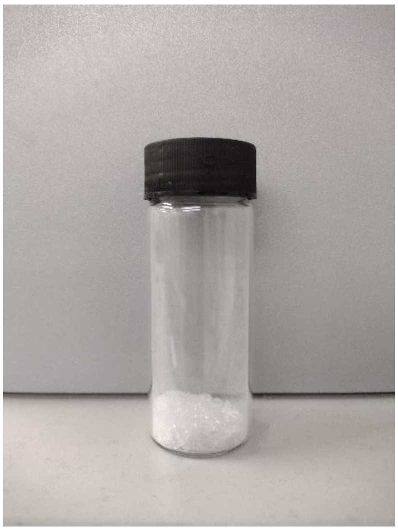

Figure S1.1: Powdered glass prepared from a Fisher Scientific borosilicate glass Pasteur pipette.

Room temperature corresponds to ~21 °C. Ambient conditions refer to a system (*e.g.* reaction mixture) open to air and kept at room temperature.

S1.2. Sample preparation

Unless otherwise stated, solutions for measurements were prepared in the glovebox, in glassware dried according to the following procedure: all glassware (including the NMR tubes), as well as syringes, needles and spatulas were dried overnight in a vacuum oven (60–65 °C, 200 mbar) and transferred to the glovebox antechamber for two hours, prior to introducing them into the glovebox.

Unless otherwise stated, the solutions were prepared freshly by weighing the solutes directly into a 1 mL volumetric flask and dissolving them in the solvent up to the 1 mL mark. Where appropriate, the time of preparation of a solution was noted and times of the NMR measurements are reported relative to it. The solution (~0.3–0.5 mL) was then transferred to a (gas-tight) J Young® valve capped NMR tube (GPE Scientific) and used for the NMR measurements. Any additives were added directly to the NMR tube before or after the addition of the solution; or to the volumetric flask containing the solution before transferring it to the NMR tube.

Where a solution was titrated, aliquots were added directly to the NMR tube inside the glovebox, and the NMR tube thoroughly shaken (manually) for at least one minute before reinserting it in the probe. In titrations with solid aliquots, small vials (prepared by flame-sealing

and detaching the upper part of a standard Norell borosilicate NMR tube) were used to aid the addition. In titrations with liquids, a 10 μL glass microsyringe was used.

S1.3. NMR spectroscopy

Unless otherwise stated, all NMR spectra were acquired on a Bruker Avance III 400 MHz NMR spectrometer equipped with a Prodigy cryoprobe (^{1}H 400 MHz, ^{13}C{^{1}H} 101 MHz, ^{19}F 377 MHz) in either manual or automation mode (without spinning). In ^{1}H and ^{13}C{^{1}H} NMR experiments, locking (tuned to ^{2}D frequency), tuning (^{1}H or ^{13}C{^{1}H}) and shimming (^{1}H) were performed prior to acquisition for each insertion of the sample into the probe and for each temperature change (in variable temperature experiments). In ^{19}F NMR experiments, the lock was off, shimming (^{1}H) and tuning (^{19}F) were performed prior to acquisition for each insertion of the sample into the probe and for each temperature change. The receiver gain was optimised for each NMR acquisition, regardless of acquisition mode. The probe temperature was 300 K, unless otherwise stated. Chemical shifts are reported in parts per million (ppm), relative to residual solvent signals for ^{1}H (THF-d_7 δ_H 1.72 ppm, 3.58 ppm; MeCN-d_2 δ_H 1.94 ppm; DMSO-d_5 δ_H 2.50 ppm) and ^{13}C{^{1}H} (THF-d_8 δ_C 25.37 ppm, 67.57 ppm; MeCN-d_3 δ_C 1.32 ppm, 118.26 ppm), and to NAPH for ^{19}F (δ_F = –123.95 ppm). Where $^{1}\Delta^{19}$F(^{32}S,^{34}S) isotope shifts are observed, the chemical shift of the major signal (^{32}S^{19}F) is reported, followed by $^{1}\Delta^{19}$F(^{32}S,^{34}S).

MestReNova (versions 14.2.1 and 14.3.0) was used to process all data, except diffusion measurements, which were processed using Bruker's Dynamics Center (version 2.5.6). Processing and analysis of the NMR spectra were performed individually (pulse-acquire, 1D NOESY) or in stack (inversion-recovery, CEST, selective inversion-recovery). First, chemical shifts of the signals were referenced as described above. The spectra were then phase- and baseline-corrected, as follows, and the appropriate signals integrated. To each ^{1}H NMR spectrum, a manual zero-order phase correction was applied, with the centre of the spectrum used as pivot. A Whittaker smoother baseline correction was then applied on the entire spectral window with an auto-detected filter and smooth factor. To each ^{19}F NMR spectrum, manual zero- and first-order phase corrections were applied, with the highest intensity signal (of the signals of interest) used as pivot; in selective inversion-recovery NMR spectra, the initially non-inverted signal was used as pivot; and only zero-order phase correction was applied in the NMR spectra where one signal was of interest. A Whittaker smoother baseline correction was then applied on a frequency range covering the signals, with auto-detected filter and smooth factor.

Quantitative ^{1}H and ^{19}F pulse-acquire NMR spectra were acquired using a standard pulse-acquire sequence (zg), where a relaxation delay, D_1, was followed by a hard $\pi/2$ pulse

and acquisition (with acquisition time t_{acq} = 4 s for ^{1}H and t_{acq} = 1.5 s for ^{19}F). $D_1 \geq 5T_1 - t_{acq}$ was used to achieve at least 99.3% longitudinal magnetisation recovery, where T_1 was the longest longitudinal relaxation time constant among spins of interest in the system. The NMR spectra were acquired with 0 dummy scans and 1 scan (unless otherwise stated). Due to a broad frequency range of ^{19}F nuclei, quantification of each signal (TBAT, FTPS, ARSF, PhF) against the internal standard (NAPH) in a given system was performed in a separate experiment with the transmitter resonance placed at approximately the average frequency of that signal and NAPH. In each case (^{1}H and ^{19}F NMR spectra), the spectral window was wide enough to cover the signals of interest.

In ^{19}F NMR spectra, the concentrations of the species of interest, [S], were calculated based on the concentrations of NAPH, [NAPH], and the integral ratios of the signals (I_{eq}^{S}/I_{eq}^{NAPH}), as presented in Equation S1.1 (n_F = 2 for TBAT; n_F = 1 for FTPS, ARSF and PhF).

S1.1

$$[\mathrm{S}] = \frac{[\mathrm{NAPH}] I_{\mathrm{eq}}^{\mathrm{S}}}{n_{\mathrm{F}} I_{\mathrm{eq}}^{\mathrm{NAPH}}}$$

S1.4. Data analysis and model fitting

Microsoft Excel was used in the analysis and plotting of all numerical data presented in this work. Where models were fitted to numerical data, non-linear regression was used with a number of parameters specified in the corresponding sections. The Solver add-in was used for this purpose (minimising sum of squares, by changing variable parameter cells). No constraints were placed on the fits and the solving method used was generalised reduced gradient (GRG) nonlinear. Root mean square error (herein referred to as *R*) was used to assess goodness of the non-linear fits, whereas R-squared (R^2) was used for linear fits. Where an average of several values was taken, it is given with a 95%-confidence interval.

S2. Solution-phase NMR spectroscopy of TBAT

S2.1. Assignment of ^{1}H and ^{13}C{^{1}H} NMR spectra

The ^{1}H NMR spectra (8 scans) of TBAT in THF-d_8 and MeCN-d_3 are presented in Figure S2.1.

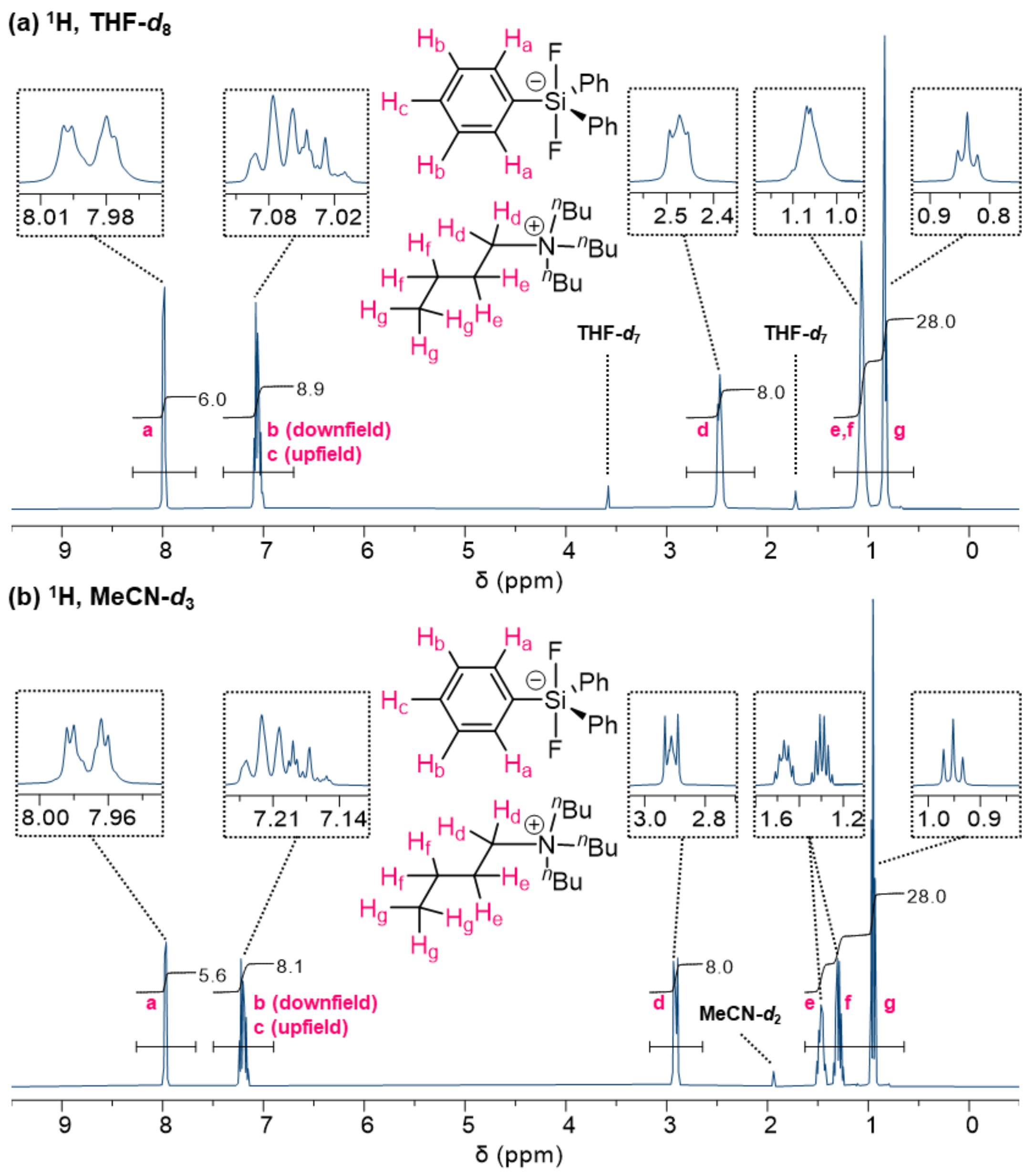

Figure S2.1: ^{1}**H NMR spectra of TBAT in (a) THF-d_8 and (b) MeCN-d_3.**

Aliphatic protons ($^n Bu_4N^+$)

In both solvents, the most upfield signal (triplet; THF-d_8: 0.84 ppm, $^3J_{HH}$ = 6.70 Hz; MeCN-d_3: 0.95 ppm, $^3J_{HH}$ = 7.35 Hz) was readily assigned as $C(H_g)_3$; and the most downfield aliphatic one (multiplet; THF-d_8: 2.45–2.49 ppm; MeCN-d_3: 2.89–2.93 ppm) – to $C(H_d)_2N$. The remaining $C(H_{e,f})_2$ signals are not resolved in THF-d_8 (multiplet, 1.00–1.12 ppm). In MeCN-d_3, these signal are resolved: $C(H_f)_2$ (apparent sextet; 1.30 ppm, $^3J_{HH}$ = 7.35 Hz); $C(H_e)_2$ (multiplet; 1.43–1.51 ppm).

Aromatic protons ($Ph_3SiF_2^-$)

In both solvents, there are two signals in the aromatic region: a downfield doublet of doublets (THF-d_8: 7.99 ppm, $^3J_{HH}$ = 7.87 Hz, $^4J_{HH}$ = 1.60 Hz; MeCN-d_3: 8.09 ppm, $^3J_{HH}$ = 8.00 Hz, $^4J_{HH}$ = 1.58 Hz) and an upfield multiplet (THF-d_8: 7.00–7.10 ppm; MeCN-d_3: 7.15–7.25 ppm). The ratio of integrals (2:3 downfield-to-upfield) indicates that C(H_c) is part of the multiplet, and the splitting pattern of the doublet of doublets (one *ortho* and one *meta* coupling) – that it is C(H_a). The upfield multiplet was analysed further with the aid of ^{1}H–^{1}H COSY (pulse sequence: cosygpmfqf; 128 dummy scans, 4 scans, D_1 = 1.50 s, t_{acq} = 0.262 s); the aromatic regions of these spectra are presented in Figure S2.2. C(H_a) and the left-hand-side of the multiplet show strong coupling, indicating that the latter is C(H_b). Weaker coupling is shown between C(H_a) and the right-hand-side of the multiplet, C(H_c). Within the multiplet, C(H_b) and C(H_c) show strong coupling to each other (overlapping diagonal and cross-peaks).

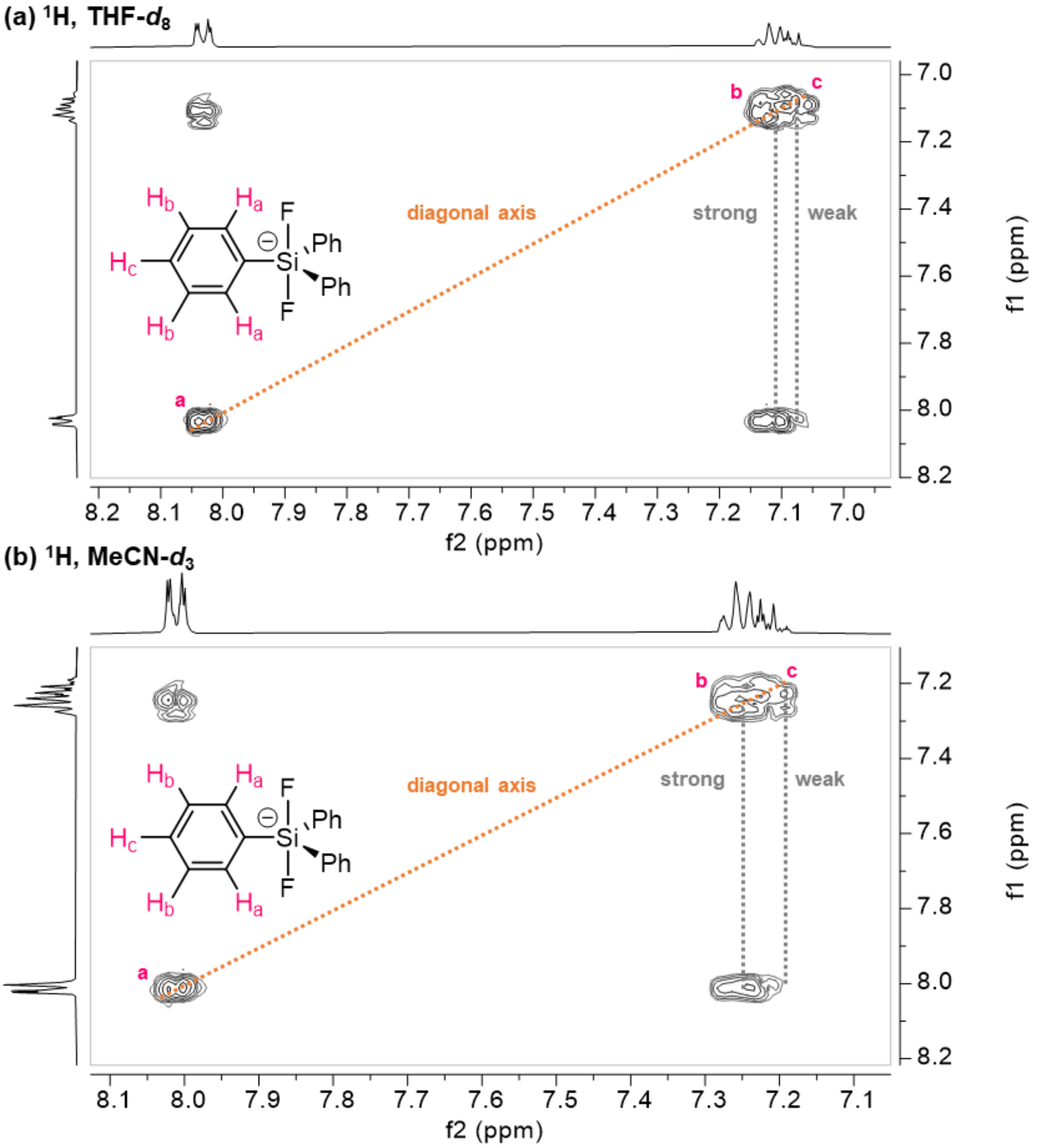

Figure S2.2: Aromatic regions of the ^{1}H–^{1}H COSY NMR spectra of TBAT in (a) THF-d_8 and (b) MeCN-d_3.

<u>^{13}C{^{1}H} NMR spectra and ^{1}H–^{13}C correlations</u>

This assignment of the aromatic ^{1}H signals was confirmed by ^{1}H–^{13}C HSQC (pulse sequence: hsqcetgpsp; 128 dummy scans, 2 scans, D_1 = 1.50 s, t_{acq} = 0.262 s) and HMBC (pulse sequence: hmbcetgpl2nd; 128 dummy scans, 2 scans, D_1 = 1.50 s, t_{acq} = 0.262 s) experiments. The ^{13}C{^{1}H} NMR spectra (pulse sequence: zgpg30; 0 dummy scans; 256 scans; D_1 = 2 s; t_{acq} = 1.26 s) of TBAT are presented in Figure S2.3 (the aliphatic ^{13}C signals were assigned based on ^{1}H–^{13}C HSQC). The aromatic regions of the spectra show: a triplet (THF-d_8: 152.7 ppm, $^{2}J_{FC}$ = 41.9 Hz; MeCN-d_3: 151.8 ppm, $^{2}J_{FC}$ = 41.5 Hz), a second triplet (THF-d_8: 138.6 ppm, $^{3}J_{FC}$ = 7.15 Hz; MeCN-d_3: 138.0 ppm, $^{3}J_{FC}$ = 6.95 Hz), and further upfield – a singlet (THF-d_8: 126.9 ppm; MeCN-d_3: 127.4 ppm) and a broad signal (perhaps a triplet with very weak

coupling; THF-d_8: 126.9 ppm, $^4J_{FC}$ < 2 Hz; MeCN-d_3: 127.3 ppm, $^4J_{FC}$ < 2 Hz), which are partially resolved. Based on the splitting patterns of the signals and measured couplings, the signals were assigned as (from downfield to upfield and with respect to the Si substituent): *ipso*, *ortho*, *para*, *meta*. Indeed, the aromatic regions of the ^1H–^{13}C HSQC spectra (Figure S2.4 and Figure S2.5 for THF-d_8 and MeCN-d_3, respectively) show correlations between C_2 and H_a, C_3 and H_b, and C_4 and H_c (no correlation was observed for C_1). In the ^1H–^{13}C HMBC spectra (aromatic regions, Figure S2.4 and Figure S2.5 for THF-d_8 and MeCN-d_3, respectively), C_1 shows strong correlations with H_a and H_b, and a weak one with H_c. C_2 shows a single-bond correlation with H_a (which is not uncommon in HMBC), a strong correlation with H_c and a weaker one with H_b.

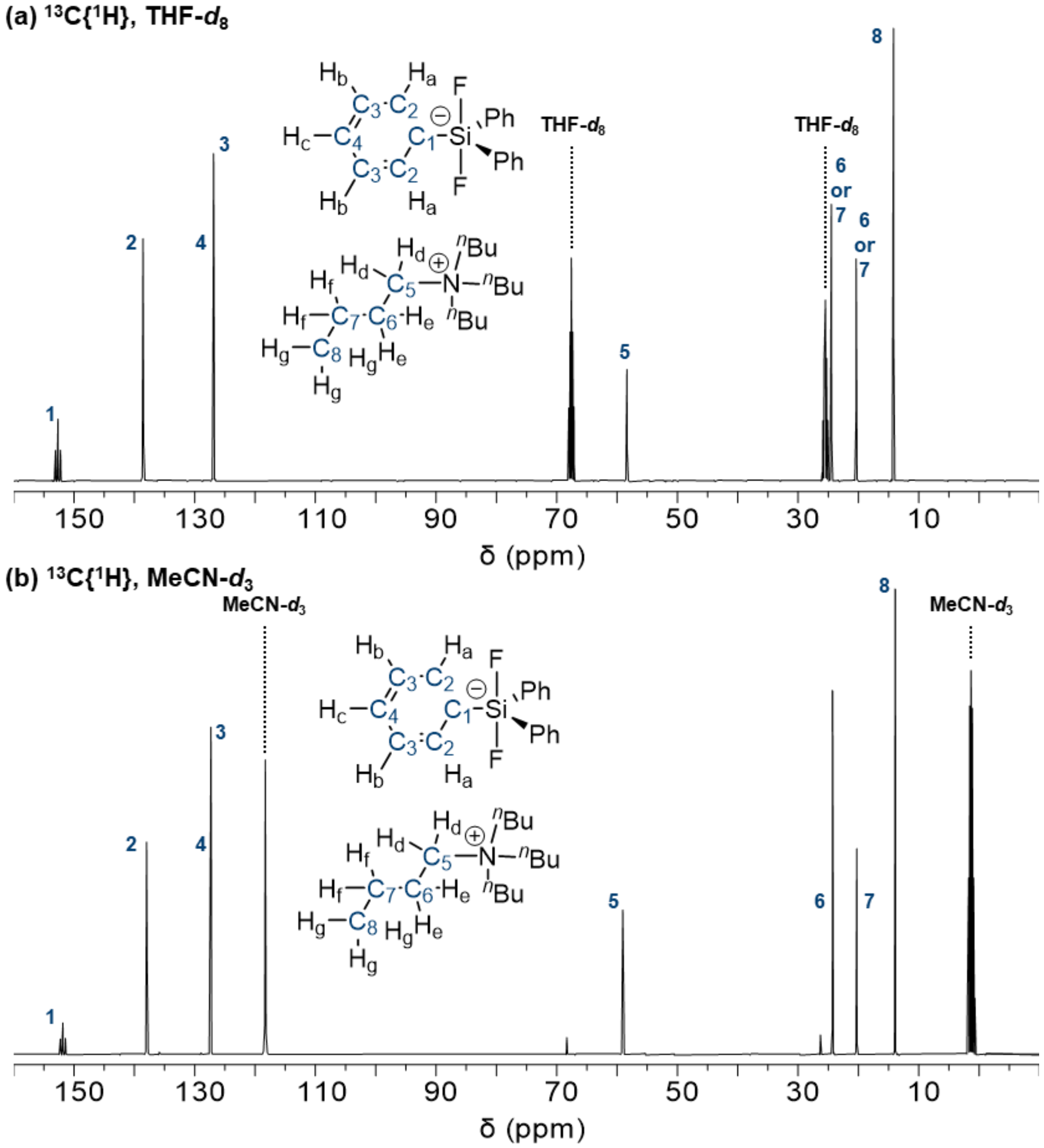

Figure S2.3: **$^{13}C\{^1H\}$ NMR spectra of TBAT in (a) THF-d_8 and (b) MeCN-d_3.**

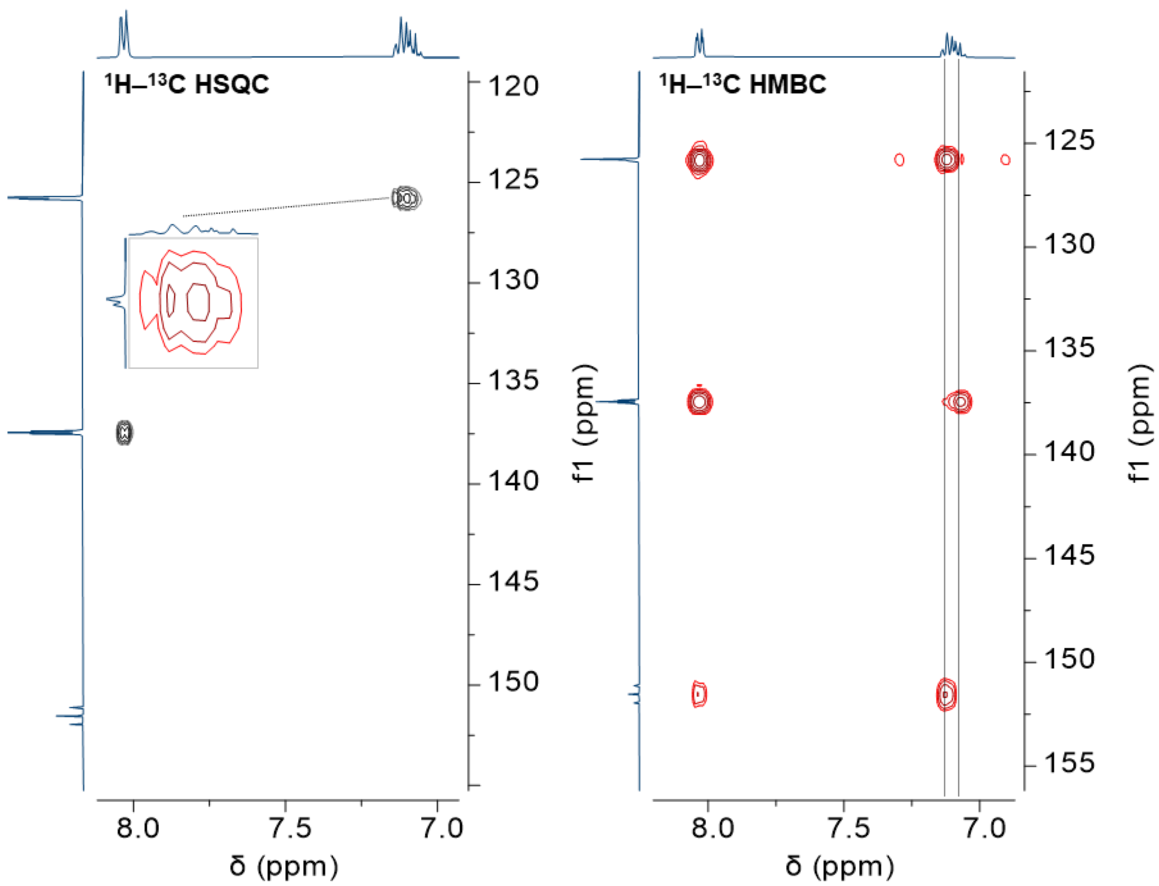

Figure S2.4: Aromatic regions of the ^{1}H–^{13}C HSQC and ^{1}H–^{13}C HMBC spectra of TBAT in THF-d_8.

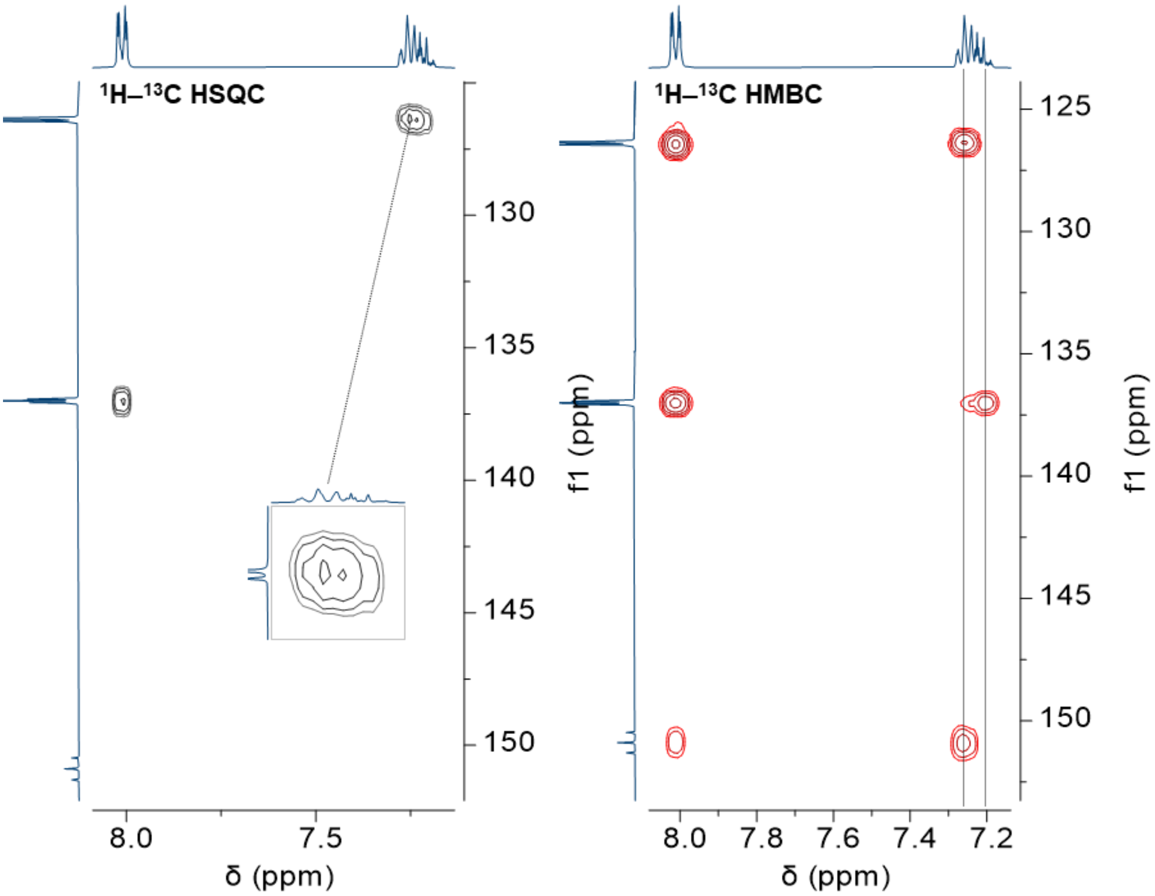

Figure S2.5: Aromatic regions of the ^{1}H–^{13}C HSQC and ^{1}H–^{13}C HMBC spectra of TBAT in MeCN-d_3.

S2.2. ^{19}F NMR spectra

^{19}F NMR spectra (8 scans for the solutions in THF) of TBAT in THF and MeCN are presented in Figure S2.6, and show one singlet for the two chemically and magnetically equivalent fluorine atoms in the $Ph_3SiF_2^-$ anion. ^{29}Si and ^{13}C satellites are also present. The J-coupling values, $^{1}J_{SiF}$ = (253.8 ± 0.4) Hz and $^{2}J_{FC}$ = (41.4 ± 0.3) Hz, determined from the ^{19}F pulse-acquire NMR spectra of solutions of TBAT in THF and MeCN described in Section S3.3.1, are consistent with the ^{29}Si and $^{13}C\{^{1}H\}$ NMR data (both in $CDCl_3$) reported by DeShong.[S3]

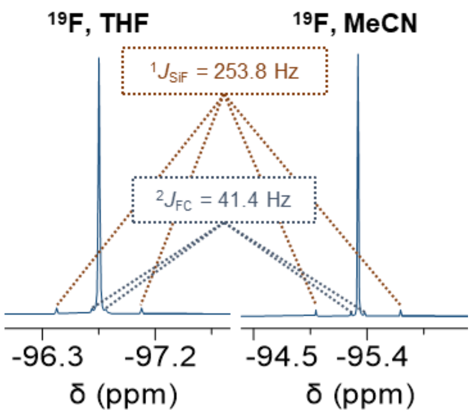

Figure S2.6: ^{19}F NMR spectra of TBAT in (a) THF and (b) MeCN.

Based on the ^{19}F pulse-acquire NMR spectra of solutions of TBAT in THF and MeCN described in Section S3.3.1, the dependence of the chemical shift of TBAT, δ^{TBAT}, on concentration, [TBAT], was determined in both solvents at 300 K (Table S2.1).

Table S2.1: Dependence of ^{19}F chemical shift of TBAT on concentration in THF and MeCN at 300 K.

Solvent	[TBAT] (mM)	δ^{TBAT} (ppm)
THF	10.3	–96.76
THF	17.4	–96.52
THF	25.6	–96.31
THF	44.9	–96.01
THF	59.2	–95.85
THF	76.6	–95.73
THF	102	–95.60
THF	122	–95.53
THF	141	–95.43
THF	161	–95.39
THF	186	–95.35
THF	208	–95.32
MeCN	79.4	–95.33
MeCN	98.8	–95.33
MeCN	121	–95.33
MeCN	142	–95.34
MeCN	169	–95.35
MeCN	187	–95.35
MeCN	207	–95.36
MeCN	229	–95.36
MeCN	14.1	–95.35
MeCN	34.0	–95.34
MeCN	53.4	–95.33
MeCN	72.7	–95.33

An empirical model was developed to fit δ^{TBAT} to concentration, [TBAT], in THF at 300 K (Equation S2.1 and Figure S2.7; R = 0.03).

S2.1

$$\frac{\delta^{\mathrm{TBAT}}}{\mathrm{ppm}} \approx 0.49 \ln \frac{[\mathrm{TBAT}]}{\mathrm{mM}} - 97.9$$

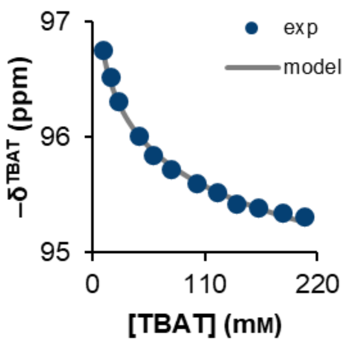

Figure S2.7: A plot of experimental ("exp") and (empirical) model ^{19}F chemical shift of TBAT against concentration in THF (at 300 K).

In contrast, the chemical shift of TBAT in MeCN, δ^{TBAT} = (–95.3 ± 0.0) ppm, was independent of [TBAT] at 300 K.

S3. Longitudinal relaxation time measurements

S3.1. General information

^{1}H and ^{19}F longitudinal relaxation time constants, T_1, were determined using a standard non-selective inversion-recovery sequence (t1ir), where after a relaxation delay, D_1, a hard π pulse was used to invert the spins, followed by variable delay, τ. The resulting longitudinal magnetisations were detected using a hard π/2 pulse and acquisition (with acquisition time t_{acq} = 4 s for ^{1}H and t_{acq} = 1.5 s for ^{19}F). This pulse sequence was repeated for a series of incremented variable delays, τ (9, 13 or 17 per experiment), which were optimised based on the longitudinal relaxation time constants of the spins. The first spectrum in each inversion-recovery experiment (τ = 0) was discarded from data analysis due to phase distortion. $D_1 \geq 5T_1 - t_{acq}$ was used to achieve at least 99.3% longitudinal magnetisation recovery, where T_1 was the longest longitudinal relaxation time constant among spins of interest in the system. The experiments were performed with 0 dummy scans and 1 scan (unless otherwise stated). Due to a broad frequency range of ^{19}F nuclei, the transmitter resonance in ^{19}F inversion-recovery experiments was placed according to signals of interest in a given system. If two signals were measured in a single experiment, this was on approximately the average frequency of the two signals and if one signal was measured – on the frequency of the signal. In each case (^{1}H and ^{19}F NMR spectra), the spectral window was wide enough to cover the signals of interest. The variable delay lists used in these experiments are given where appropriate.

To the experimental integrals within each dataset was fitted a three-parameter (I_{eq}, x, T_1) first-order longitudinal relaxation model, as presented in Equation S3.1, where I_τ is the integral

at variable delay τ, I_{eq} is the integral at magnetic equilibrium (*i.e.*, fully recovered longitudinal magnetisation) and x is the correction parameter for imperfect inversion ($0 \leq x \leq 2$). The experimental and model integrals were then converted into fractional magnetisations for plotting (Equation S5.3).

S3.1

$$I_\tau = I_{eq}\left(1 - xe^{-\frac{\tau}{T_1}}\right)$$

S3.2. ^{1}H longitudinal relaxation time measurements

S3.2.1. Tetra-*n*-butylammonium difluorotriphenylsilicate (TBAT)

^{1}H longitudinal relaxation time constants of TBAT were measured in order to ensure sufficient recovery of the signals in speciation experiments. The variable delays used in the experiments were, τ (s) {1, 2, 5, 7.5, 10, 12, 15, 20}. The ^{1}H inversion-recovery experiment was then repeated for the solutions in MeCN-d_3 due to incomplete relaxation of $Ph_3SiF_2^-$. The variable delays used in these new experiments were: τ (s) {5, 10, 15, 25, 35, 50, 65, 80} for 40 mM TBAT in MeCN-d_3 at 335 K, and τ (s) {2, 5, 10, 15, 20, 30, 45, 60} for all other solutions in MeCN-d_3.

All signals corresponding to $Ph_3SiF_2^-$ ("–") were integrated together, as were ones corresponding to $^nBu_4N^+$ ("+"); except for the solution of TBAT in DMSO-d_6, where the most downfield signal corresponding to $^nBu_4N^+$ was excluded due to proximity to the residual solvent signal.

The determined ^{1}H longitudinal relaxation fitting parameters (I_{eq}, x and T_1) and root mean square errors (R) of $Ph_3SiF_2^-$ and $^nBu_4N^+$ in solutions used for ion pairing studies in the absence of internal diffusion standard (Section S4.1.1) are presented in Table S3.1. The plots of their experimental and model fractional magnetisations, m^- and m^+, against variable delay, τ, are presented in Figure S3.1–Figure S3.4.

Table S3.1: Longitudinal relaxation fitting parameters and root mean square errors for $Ph_3SiF_2^-$ ("–") and $^nBu_4N^+$ ("+") in ^1H inversion-recovery experiments of solutions in Section S4.1.1.

Solvent	[TBAT] (mM)[a]	T (K)	$10^{-3}I_{eq}$		x		T_1 (s)		$10^{-3}R$	
			–	+	–	+	–	+	–	+
THF-d_8	220	300	63.1	152	1.78	1.06	2.06	0.954	0.2	0
THF-d_8	220	310	62.8	151	1.78	1.07	2.34	1.08	0.2	1
THF-d_8	220	320	68.6	165	1.78	1.14	2.68	1.16	0.3	1
THF-d_8	110	300	55.3	133	1.79	1.07	2.34	1.02	0.2	1
THF-d_8	110	310	58.7	141	1.80	1.14	2.66	1.11	0.2	1
THF-d_8	110	320	55.7	134	1.81	1.20	2.99	1.24	0.2	1
THF-d_8	30	300	64.6	156	1.81	1.13	2.81	1.14	0.2	1
THF-d_8	30	310	63.5	154	1.82	1.18	3.20	1.29	0.2	2
THF-d_8	30	320	67.3	164	1.84	1.28	3.56	1.39	0.1	2
MeCN-d_3	220	300	66.7	154	1.79	1.51	6.64	1.49	0.3	1
MeCN-d_3	220	320	63.2	155	1.82	1.57	8.46	1.95	0.2	2
MeCN-d_3	220	335	59.9	158	1.88	1.57	8.49	2.34	0.9	2
MeCN-d_3	120	300	56.3	140	1.81	1.55	8.02	1.65	0.3	1
MeCN-d_3	120	320	52.7	144	1.88	1.61	8.52	2.11	0.3	2
MeCN-d_3	120	335	55.8	138	1.90	1.66	9.25	2.38	0.6	2
MeCN-d_3	40	300	44.9	156	1.83	1.61	9.42	1.83	0.2	2
MeCN-d_3	40	320	66.0	160	1.85	1.64	11.6	2.39	0.2	3
MeCN-d_3	40	335	43.1	171	1.81	1.70	13.0	2.66	0.1	2

[a] To the nearest 10 mM.

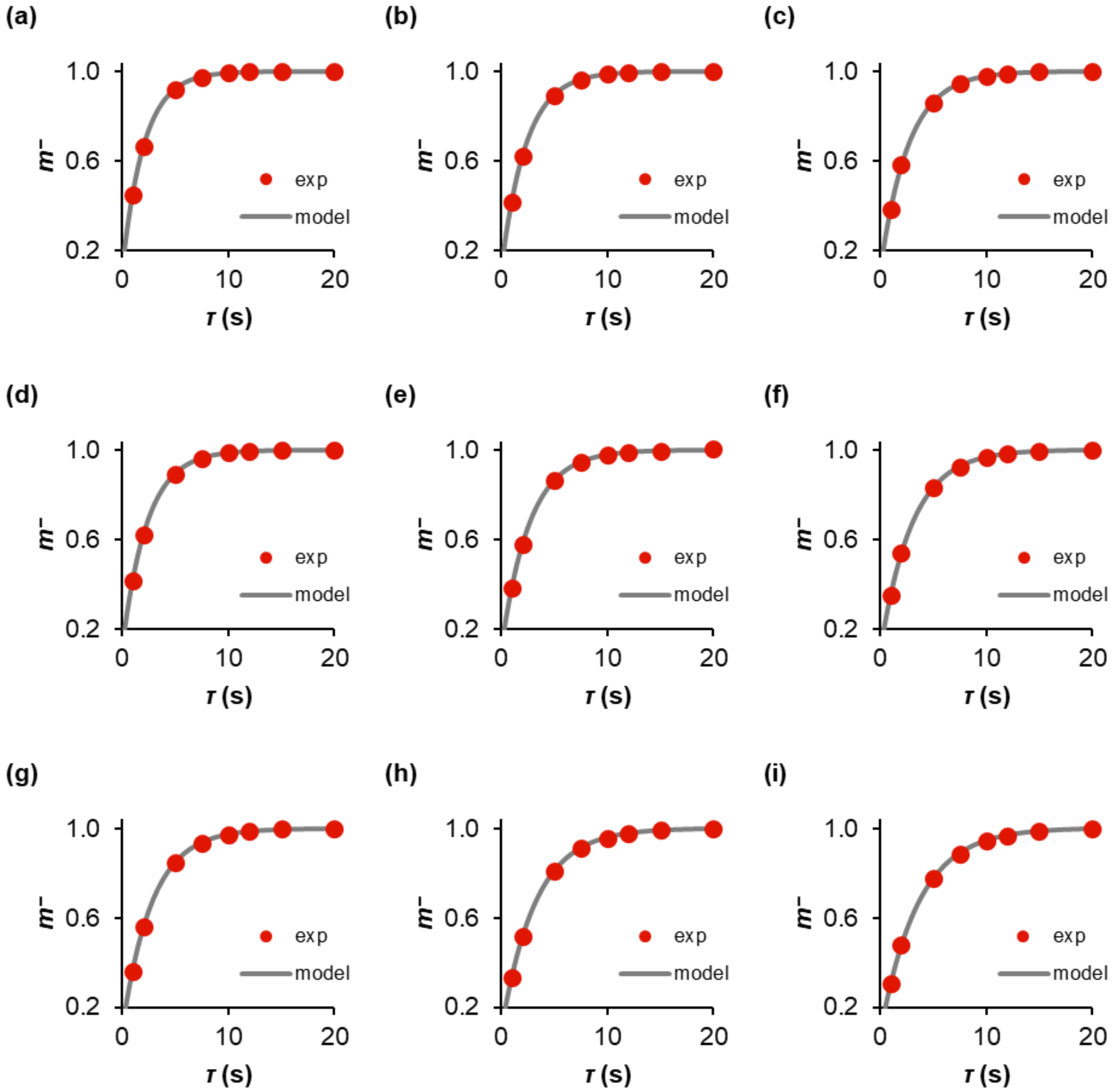

Figure S3.1: Plots of experimental ("exp") and model fractional magnetisations of $Ph_3SiF_2^-$, m^-, in ^{1}H longitudinal relaxation constant measurements of TBAT in THF-d_8. (a) 220 mM at 300 K; (b) 220 mM at 310 K; (c) 220 mM at 320 K; (d) 110 mM at 300 K; (e) 110 mM at 310 K; (f) 110 mM at 320 K; (g) 30 mM at 300 K; (h) 30 mM at 310 K; (i) 30 mM at 320 K.

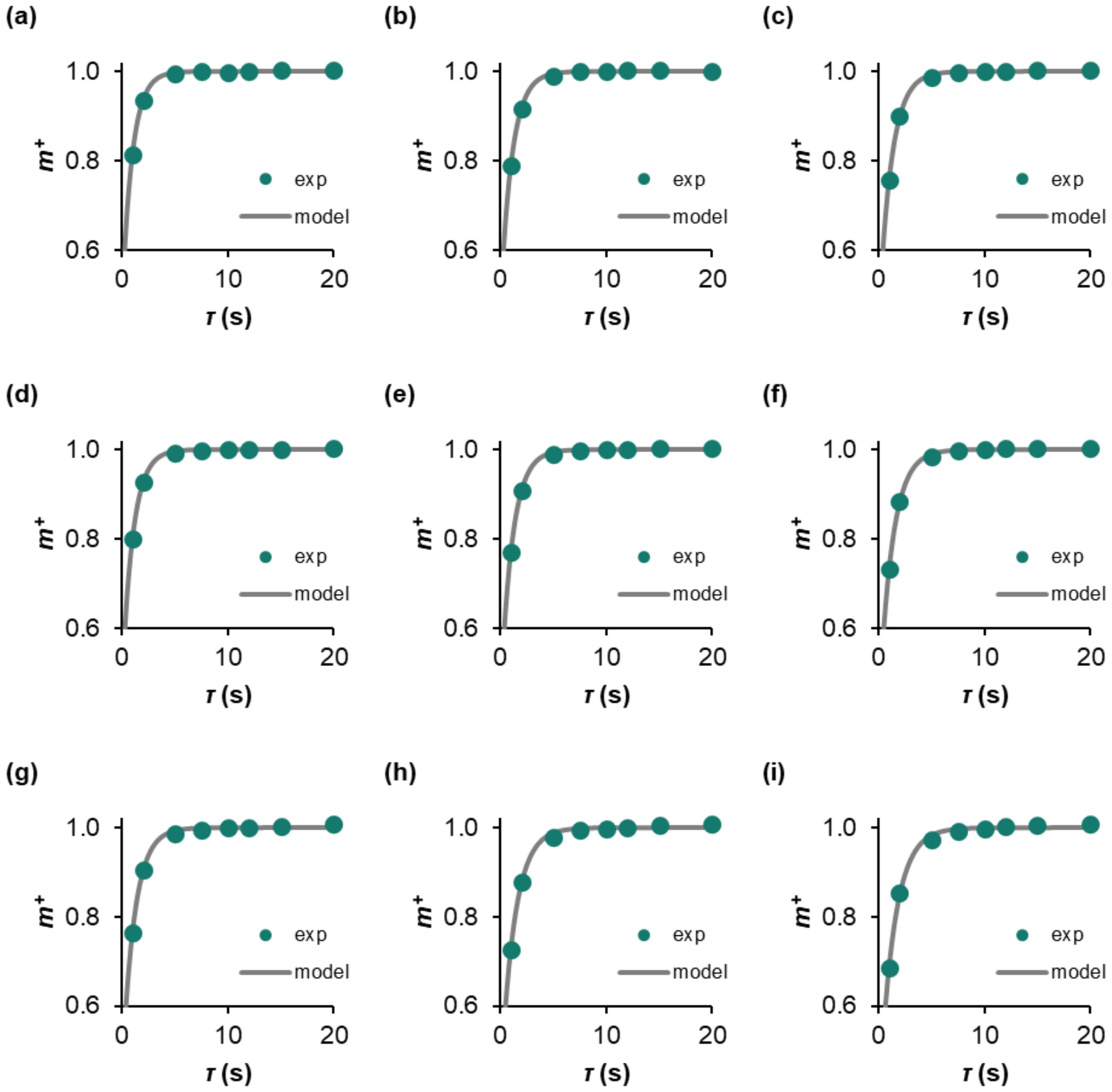

Figure S3.2: Plots of experimental (“exp”) and model fractional magnetisations of ^{n}Bu$_4$N$^+$, m^+, in ^{1}H longitudinal relaxation constant measurements of TBAT in THF-d_8. (a) 220 mM at 300 K; (b) 220 mM at 310 K; (c) 220 mM at 320 K; (d) 110 mM at 300 K; (e) 110 mM at 310 K; (f) 110 mM at 320 K; (g) 30 mM at 300 K; (h) 30 mM at 310 K; (i) 30 mM at 320 K.

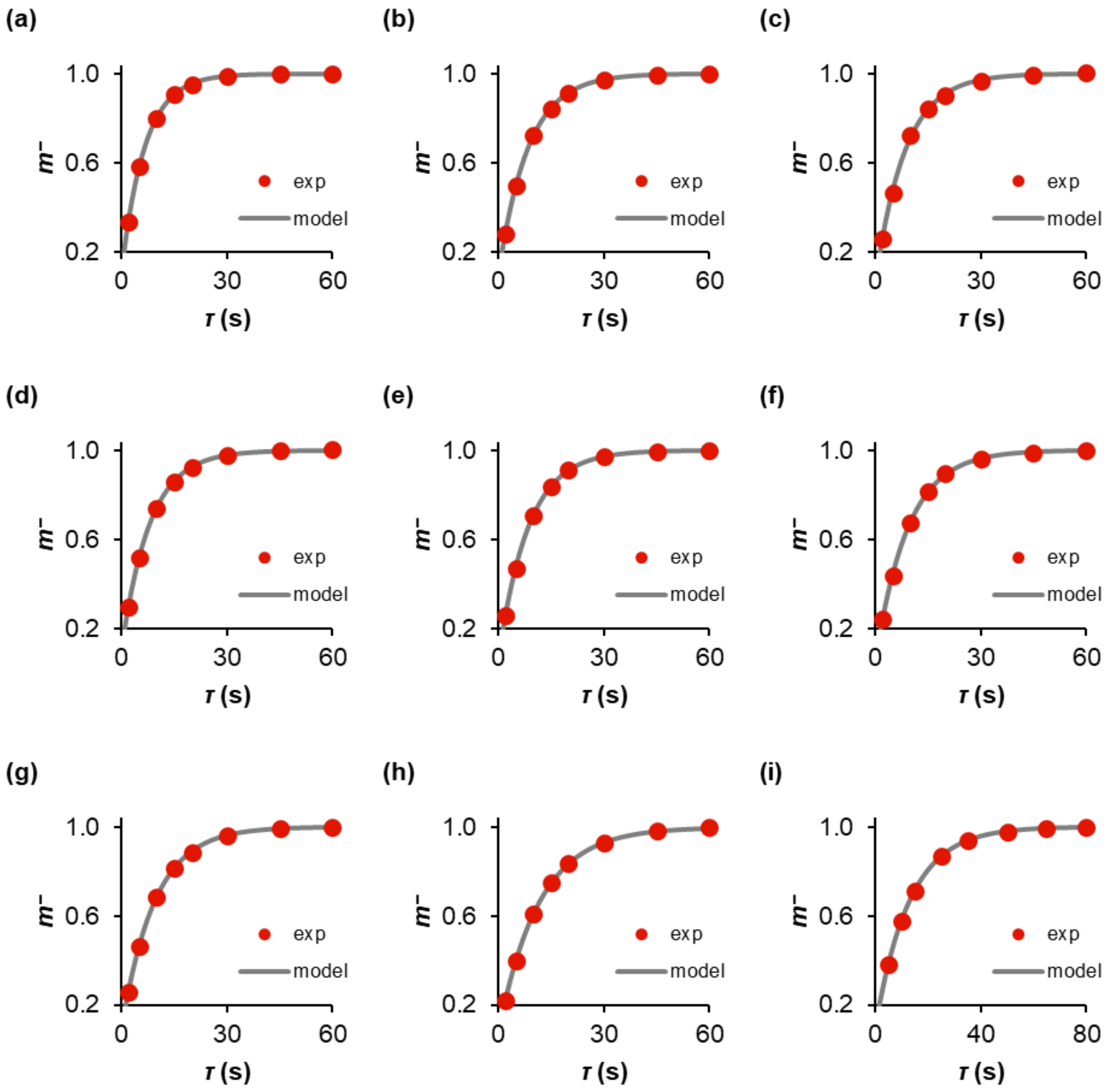

Figure S3.3: Plots of experimental (“exp”) and model fractional magnetisations of $Ph_3SiF_2^-$, m^-, in ^1H longitudinal relaxation constant measurements of TBAT in MeCN-d_3. (a) 220 mM at 300 K; (b) 220 mM at 320 K; (c) 220 mM at 335 K; (d) 120 mM at 300 K; (e) 120 mM at 320 K; (f) 120 mM at 335 K; (g) 40 mM at 300 K; (h) 40 mM at 320 K; (i) 40 mM at 335 K.

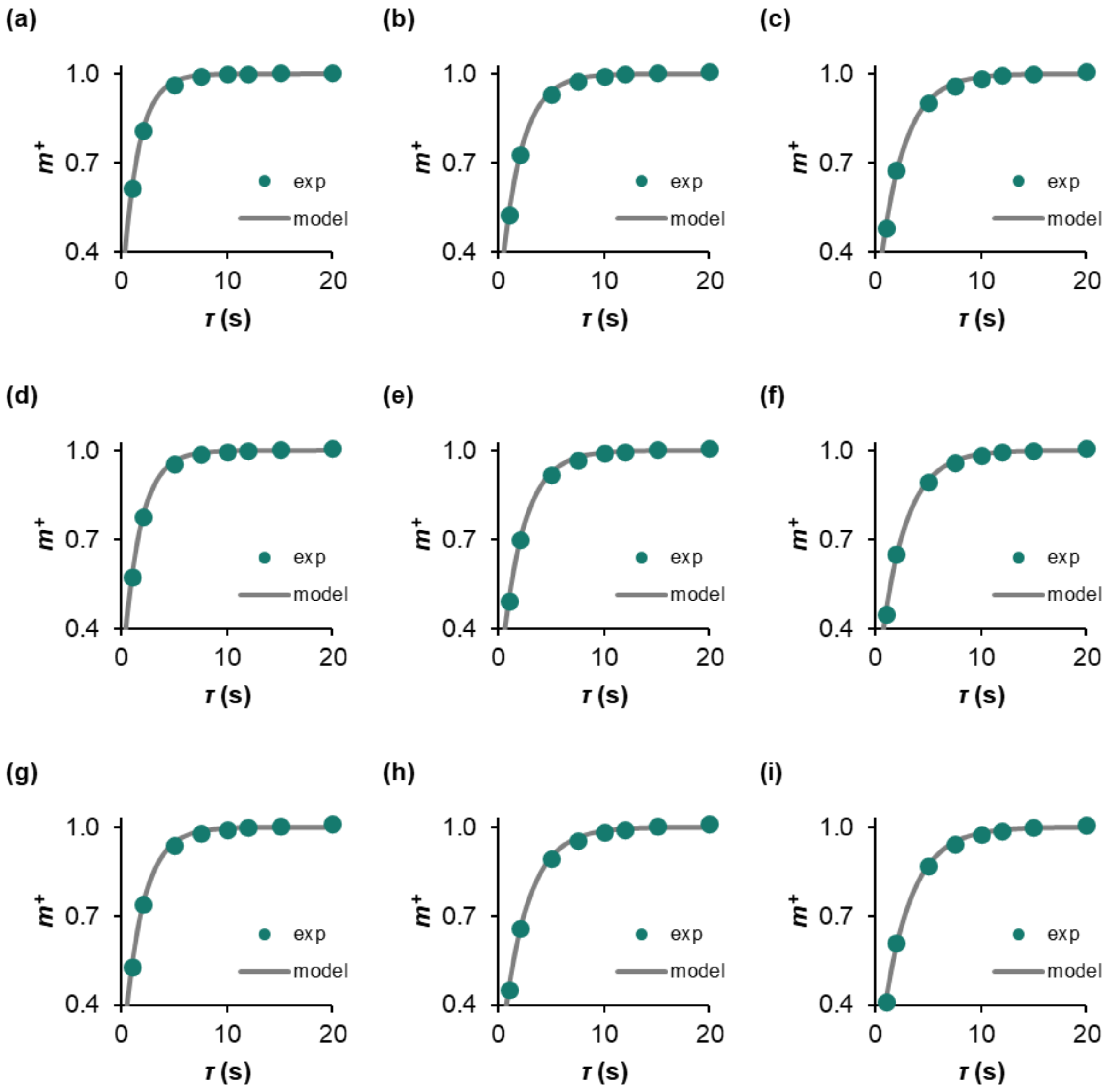

Figure S3.4: Plots of experimental ("exp") and model fractional magnetisations of $^{n}Bu_4N^+$, m^+, in ^{1}H longitudinal relaxation constant measurements of TBAT in MeCN-d_3. (a) 220 mM at 300 K; (b) 220 mM at 320 K; (c) 220 mM at 335 K; (d) 120 mM at 300 K; (e) 120 mM at 320 K; (f) 120 mM at 335 K; (g) 40 mM at 300 K; (h) 40 mM at 320 K; (i) 40 mM at 335 K.

The determined ^{1}H longitudinal relaxation fitting parameters (I_{eq}, x and T_1) and root mean square errors (R) of $Ph_3SiF_2^-$, $^{n}Bu_4N^+$ and the internal diffusion standard (CH_3, "TMB") in solutions used for ion pairing studies in the presence of internal diffusion standard (Section S4.1.2) are presented in Table S3.2. The plots of their experimental and model fractional magnetisations, m^-, m^+ and m^{TMB} against variable delay, τ, are presented in Figure S3.5 and Figure S3.6.

Table S3.2: Longitudinal relaxation fitting parameters and root mean square errors for $Ph_3SiF_2^-$ (“–”), $^nBu_4N^+$ (“+”) and the internal diffusion standard (CH_3, “TMB”) in ^1H inversion-recovery experiments of solutions in Section S4.1.2.

Solvent	[TBAT] (mM)	T (K)	$10^{-3}I_{eq}$		x		T_1 (s)		$10^{-3}R$	
			–	+	–	+	–	+	–	+
THF-d_8[a]	207	300	47.5	114	1.78	1.05	1.99	0.935	0.3	0
DMSO-d_6[b]	1.5	300	1.09	2.37	1.81	1.27	2.90	1.02	0.01	0.01

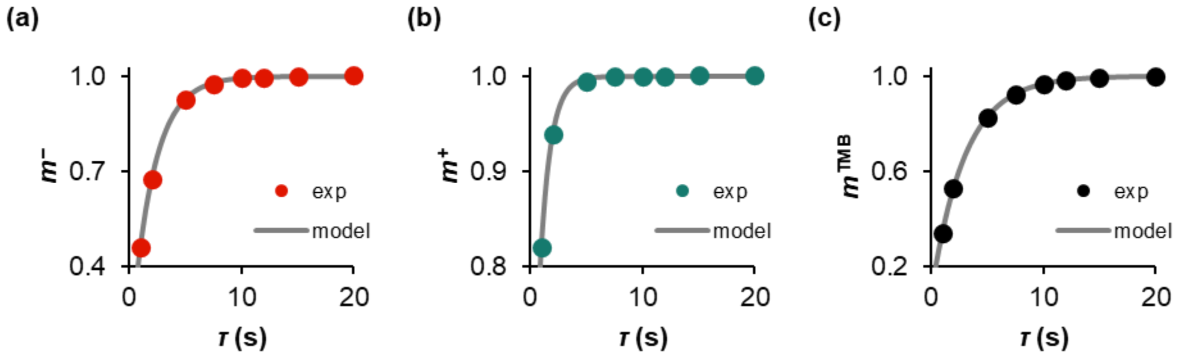

Figure S3.5: Plots of experimental (“exp”) and model fractional magnetisations of (a) $Ph_3SiF_2^-$, m^-, (b) $^nBu_4N^+$, m^+, and (c) TMB (CH_3), m^{TMB}, in ^1H longitudinal relaxation constant measurements of TBAT in THF-d_8 at 300 K (207 mM TBAT, 252 mM TMB).

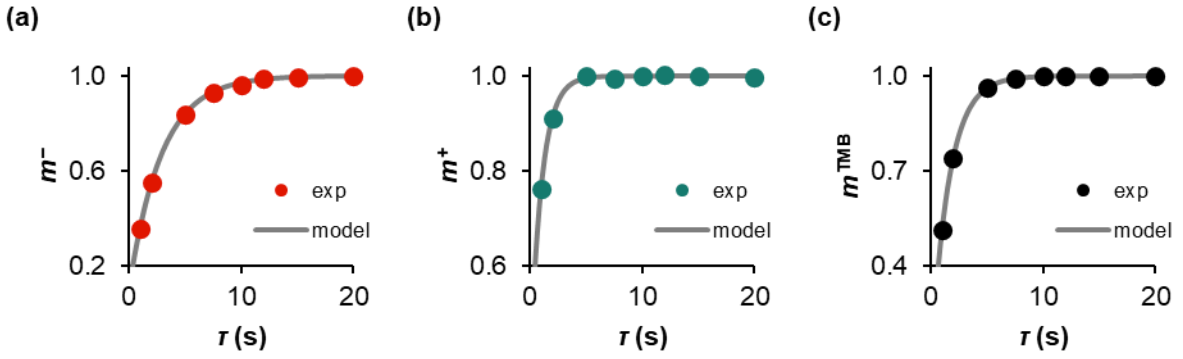

Figure S3.6: Plots of experimental (“exp”) and model fractional magnetisations of (a) $Ph_3SiF_2^-$, m^-, (b) $^nBu_4N^+$, m^+, and (c) TMB (CH_3), m^{TMB}, in ^1H longitudinal relaxation constant measurements of TBAT in DMSO-d_6 at 300 K (1.5 mM TBAT, 263 mM TMB).

S3.2.2. Tetra-*n*-butylammonium tetrakis[3,5-bis(trifluoromethyl)phenyl]borate (TBABARF)

^1H longitudinal relaxation time constants of TBABARF were measured in order to ensure sufficient recovery of the signals in speciation experiments. The variable delays used in the experiments were, τ (s) {1, 2, 5, 7.5, 10, 12, 15, 20}. All signals corresponding to $BARF^-$ (“–”) were integrated together, as were ones corresponding to $^nBu_4N^+$ (“+”).

[a] $10^{-3}I_{eq}^{TMB} = 35.5$, $x^{TMB} = 1.85$, $T_1^{TMB} = 2.99$ s, $10^{-3}R^{TMB} = 0.0$.
[b] $10^{-3}I_{eq}^{TMB} = 125$, $x^{TMB} = 1.83$, $T_1^{TMB} = 1.58$ s, $10^{-3}R^{TMB} = 0$.

The determined ^{1}H longitudinal relaxation fitting parameters (I_{eq}, x and T_1) and root mean square errors (R) of $BARF^{-}$, $^{n}Bu_4N^{+}$ and the internal diffusion standard (CH_3, "TMB") in the solution used for ion pairing studies in the presence of internal diffusion standard (Section S4.1.3) are presented in Table S3.3. The plots of their experimental and model fractional magnetisations, m^{-}, m^{+} and m^{TMB} against variable delay, τ, are presented in Figure S3.7.

Table S3.3: Longitudinal relaxation fitting parameters and root mean square errors for $BARF^{-}$ ("–"), $^{n}Bu_4N^{+}$ ("+") and the internal diffusion standard (CH_3, "TMB") in ^{1}H inversion-recovery experiment of solution in Section S4.1.3.

Solvent	[TBABARF] (mM)	T (K)	$10^{-3}I_{eq}$		x		T_1 (s)		$10^{-3}R$	
			–	+	–	+	–	+	–	+
THF-d_8[a]	201	300	41.8	115	1.72	1.25	2.70	1.16	0.5	1

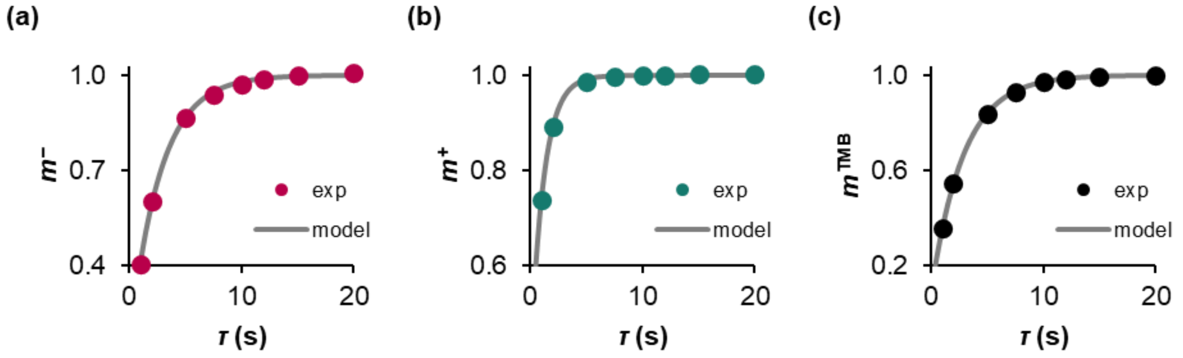

Figure S3.7: Plots of experimental ("exp") and model fractional magnetisations of (a) $BARF^{-}$, m^{-}, (b) $^{n}Bu_4N^{+}$, m^{+}, and (c) TMB (CH_3), m^{TMB}, in ^{1}H longitudinal relaxation constant measurement of TBABARF in THF-d_8 at 300 K (201 mM TBABARF, 256 mM TMB).

S3.3. ^{19}F longitudinal relaxation time measurements

^{19}F longitudinal relaxation time constants of TBAT, FTPS and ARSF were measured in solutions of these species in THF and in MeCN (at 300 K in both solvents).

S3.3.1. Tetra-*n*-butylammonium difluorotriphenylsilicate (TBAT)

<u>Solutions of TBAT in THF</u>

Three solutions were prepared in the glovebox by weighing TBAT and NAPH into 1 mL volumetric flasks and dissolving them in THF up to the 1 mL mark. The concentrations in the solutions are presented in Table S3.4. The solutions were transferred to J Young® valve capped NMR tubes. For each solution, ^{19}F pulse-acquire (8 scans) and ^{19}F inversion-recovery experiments were run at 300 K. Each solution was then titrated with three solid aliquots of TBAT. ^{19}F pulse-acquire and ^{19}F inversion-recovery experiments were run (at 300 K) after each aliquot addition. The variable delays used in the inversion-recovery experiments were, τ

[a] $10^{-3}I_{eq}^{TMB}$ = 40.4, x^{TMB} = 1.83, T_1^{TMB} = 2.88 s, $10^{-3}R^{TMB}$ = 0.0.

(s) {0.2, 0.5, 1, 1.5, 2, 3, 5, 7.5, 10, 12.5, 15, 20}. The ^{19}F inversion-recovery experiments were repeated for the original three solutions to determine T_1^{NAPH}. The variable delays used in these new experiments were, τ (s) {1, 2, 5, 7.5, 10, 12.5, 15, 20, 25, 30, 35, 40}.

Table S3.4: Concentrations of TBAT and NAPH in the solutions of TBAT in THF.

Entry	Concentration (mM)	
	TBAT	NAPH
1	9.50	185
2	59.5	211
3	141	529

The concentrations of TBAT (based on integration against NAPH), [TBAT], the determined ^{19}F longitudinal relaxation fitting parameters (I_{eq}, x and T_1) and root mean square errors (R) are presented in Table S3.5. The plots of experimental and model fractional magnetisations, m^{TBAT}, against variable delay, τ, are presented in Figure S3.8 and the plots for NAPH – in Figure S3.9. An empirical model was fitted that describes T_1^{TBAT} as a function of [TBAT] at 300 K (Equation S3.2 and Figure S3.10; R = 0.02).

Table S3.5: Longitudinal relaxation fitting parameters and root mean square errors of TBAT in THF.

Entry	[TBAT] (mM)	$10^{-3}I_{eq}^{TBAT}$	x^{TBAT}	T_1^{TBAT} (s)	$10^{-3}R^{TBAT}$
1[ab]	10.3	1.30	1.85	2.00	0.00
1, aliq. 1[c]	17.4	1.07	1.86	1.84	0.00
1, aliq. 2[d]	25.6	0.797	1.86	1.74	0.004
1, aliq. 3	44.9	0.685	1.87	1.58	0.002
2[e]	59.2	0.950	1.85	1.52	0.003
2, aliq. 1	76.6	1.22	1.84	1.47	0.00
2, aliq. 2	102	1.60	1.84	1.41	0.00
2, aliq. 3	122	1.89	1.84	1.37	0.00
3[f]	141	2.20	1.84	1.28	0.01
3, aliq. 1	161	2.50	1.83	1.27	0.01
3, aliq. 2	186	2.82	1.83	1.23	0.01
3, aliq. 3	208	3.15	1.83	1.22	0.02

[a] 8 scans in ^{19}F inversion-recovery experiment for T_1^{TBAT}.
[b] $10^{-3}I_{eq}^{NAPH}$ = 1.41, x^{NAPH} = 1.86, T_1^{NAPH} = 7.38 s, $10^{-3}R^{NAPH}$ = 0.00.
[c] 4 scans in ^{19}F inversion-recovery experiment for T_1^{TBAT}.
[d] 2 scans in ^{19}F inversion-recovery experiment for T_1^{TBAT}.
[e] $10^{-3}I_{eq}^{NAPH}$ = 1.53, x^{NAPH} = 1.85, T_1^{NAPH} = 7.07 s, $10^{-3}R^{NAPH}$ = 0.00.
[f] $10^{-3}I_{eq}^{NAPH}$ = 4.23, x^{NAPH} = 1.84, T_1^{NAPH} = 6.50 s, $10^{-3}R^{NAPH}$ = 0.01.

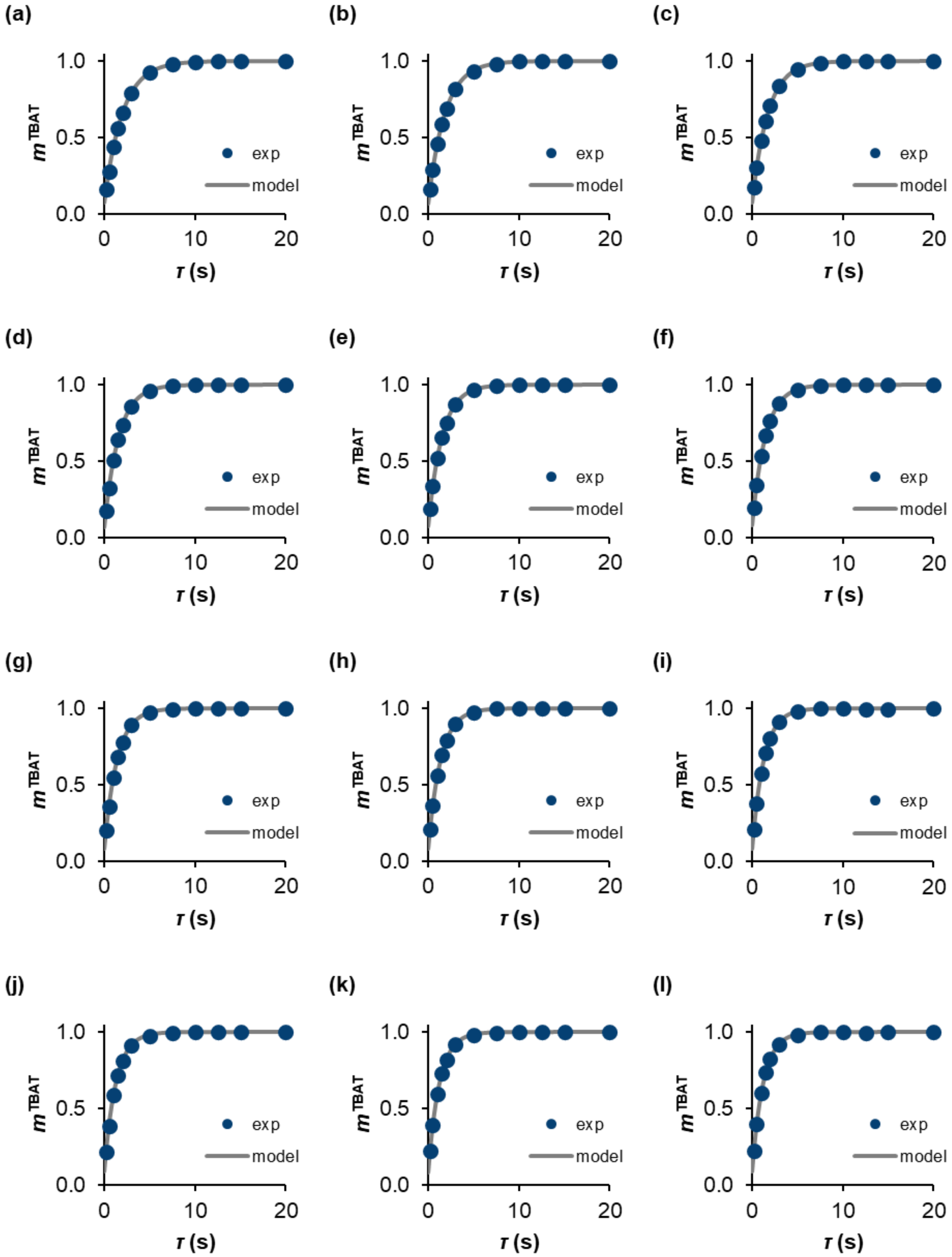

Figure S3.8: Plots of experimental ("exp") and model fractional magnetisations of TBAT, m^{TBAT}, in ^{19}F longitudinal relaxation constant measurements of TBAT in THF at 300 K. (a) 10.3 mM; (b) 17.4 mM; (c) 25.6 mM; (d) 44.9 mM; (e) 59.2 mM; (f) 76.6 mM; (g) 102 mM; (h) 122 mM; (i) 141 mM; (j) 161 mM; (k) 186 mM; (l) 208 mM.

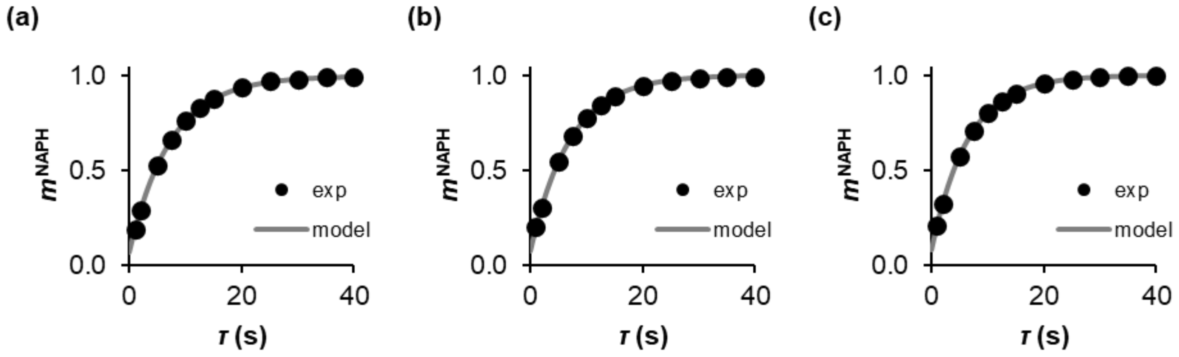

Figure S3.9: Plots of experimental ("exp") and model fractional magnetisations of NAPH, m^{NAPH}, in ^{19}F longitudinal relaxation constant measurements of NAPH in THF at 300 K. (a) 185 mM; (b) 211 mM; (c) 529 mM.

S3.2

$$\frac{T_1^{\mathrm{TBAT}}}{\mathrm{s}} \approx -0.26 \ln \frac{[\mathrm{TBAT}]}{\mathrm{mM}} + 2.6$$

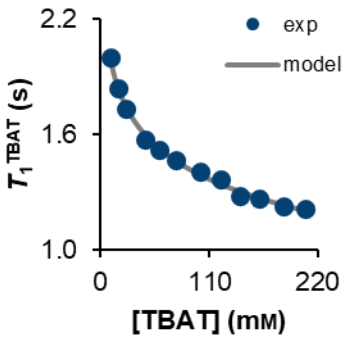

Figure S3.10: Plots of experimental ("exp") and (empirical) model longitudinal relaxation time of TBAT against concentration in THF at 300 K.

<u>Solutions of TBAT in MeCN</u>

Three solutions were prepared in the glovebox by weighing TBAT and NAPH into 1 mL volumetric flasks and dissolving them in NAPH up to the 1 mL mark. The concentrations in the solutions are presented in Table S3.6. The solutions were transferred to J Young® valve capped NMR tubes. For each solution, ^{19}F pulse-acquire and ^{19}F inversion-recovery experiments were run at 300 K. Each solution was then titrated with three solid aliquots of TBAT. ^{19}F pulse-acquire and ^{19}F inversion-recovery experiments were run (at 300 K) after each aliquot addition. The variable delays used in the experiments were, *τ* (s) {0.5, 1, 2, 3, 4, 5, 7, 10, 15, 20, 30, 40}.

Table S3.6: Concentrtions of TBAT and NAPH in solutions.

Entry	Concentration (mM)	
	TBAT	NAPH
1	83.7	145
2	166	159
3	14.1	94.9

The concentrations of TBAT (based on integration against NAPH), [TBAT], the determined ^{19}F longitudinal relaxation fitting parameters (I_{eq}, x and T_1) and root mean square errors (R) are presented in Table S3.7. The plots of experimental and model fractional magnetisations, m^{TBAT}, against variable delay, τ, are presented in Figure S3.11. An empirical model was fitted that describes T_1^{TBAT} as a function of [TBAT] at 300 K (Equation S3.3 and Figure S3.12; R = 0.05).

Table S3.7: Longitudinal relaxation fitting parameters and root mean square errors of TBAT in MeCN.

Entry	[TBAT] (mM)	$10^{-3}I_{eq}^{TBAT}$	x^{TBAT}	T_1^{TBAT} (s)	$10^{-3}R^{TBAT}$
1	79.4	1.30	1.86	6.06	0.00
1, aliq. 1	98.8	1.60	1.85	5.71	0.00
1, aliq. 2	121	1.94	1.85	5.33	0.00
1, aliq. 3	142	2.24	1.84	5.21	0.00
2	169	2.63	1.84	4.94	0.00
2, aliq. 1	187	2.89	1.83	4.83	0.01
2, aliq. 2	207	3.15	1.84	4.65	0.01
2, aliq. 3	229	3.43	1.83	4.46	0.01
3	14.1	0.213	1.87	7.14	0.001
3, aliq. 1	34.0	0.505	1.86	6.73	0.002
3, aliq. 2	53.4	0.840	1.86	6.42	0.001
3, aliq. 3	72.7	1.12	1.86	6.22	0.00

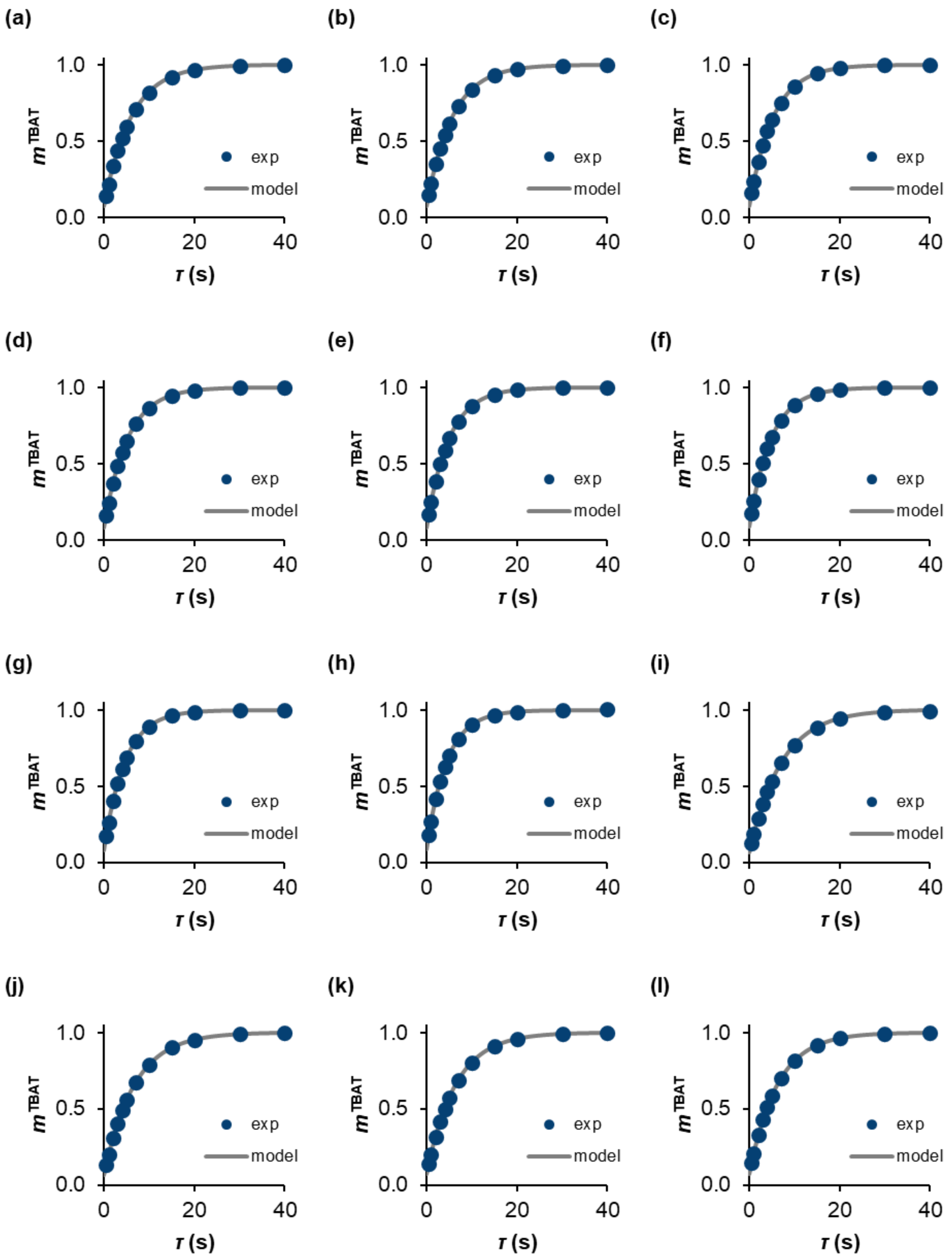

Figure S3.11: Plots of experimental ("exp") and model fractional magnetisations of TBAT, m^{TBAT}, in ^{19}F longitudinal relaxation constant measurements of TBAT in MeCN at 300 K. (a) 79.4 mM; (b) 98.8 mM; (c) 121 mM; (d) 142 mM; (e) 169 mM; (f) 187 mM; (g) 207 mM; (h) 229 mM; (i) 14.1 mM; (j) 34.0 mM; (k) 53.4 mM); (l) 72.7 mM.

S3.3

$$\frac{T_1^{\mathrm{TBAT}}}{\mathrm{s}} \approx 32.0\left(\frac{[\mathrm{TBAT}]}{\mathrm{M}}\right)^2 - 20.1\frac{[\mathrm{TBAT}]}{\mathrm{M}} + 7.4$$

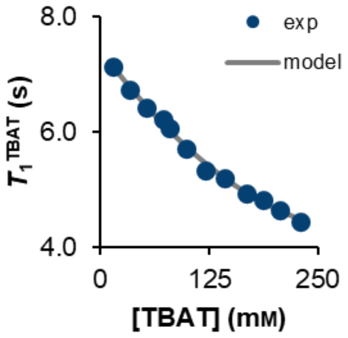

Figure S3.12: Plots of experimental ("exp") and (empirical) model longitudinal relaxation time of TBAT against concentration in MeCN at 300 K.

Saturated solutions of TBAT in THF and MeCN for ^{19}F CEST experiments

The variable delays used in the experiments were, τ (s) {0.1, 0.5, 1, 2, 3, 5, 7.5, 10} for THF and τ (s) {1, 3, 5, 7.5, 10, 12.5, 15, 20} for MeCN.

The determined ^{19}F longitudinal relaxation fitting parameters (I_{eq}, x and T_1) and root mean square errors (R) of TBAT in solutions used for ^{19}F CEST experiments (Section S4.3) are presented in Table S3.8. The plots of their experimental and model fractional magnetisations, m^{TBAT}, against variable delay, τ, are presented in Figure S3.13.

Table S3.8: Longitudinal relaxation fitting parameters and root mean square errors of TBAT in saturated solutions in THF and MeCN.

Solvent	[TBAT]	$10^{-3}I_{\mathrm{eq}}^{\mathrm{TBAT}}$	x^{TBAT}	T_1^{TBAT} (s)	$10^{-3}R^{\mathrm{TBAT}}$
THF	Saturated	8.95	1.85	1.10	0.03
MeCN	Saturated	8.16	1.83	3.60	0.01

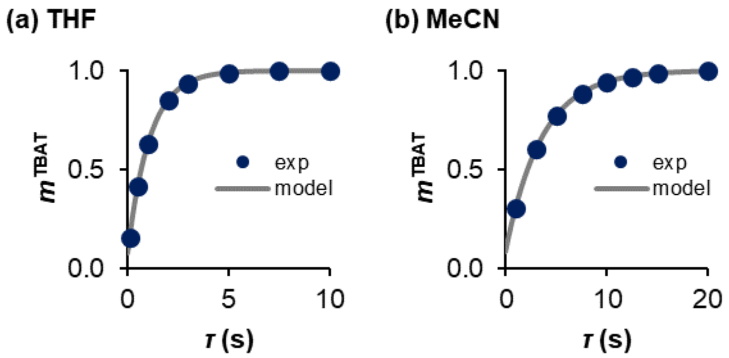

Figure S3.13: Plots of experimental ("exp") and model fractional magnetisations of TBAT, m^{TBAT}, in ^{19}F longitudinal relaxation constant measurements of TBAT in saturated solutions in (a) THF and (b) MeCN (both at 300 K).

S3.3.2. Fluorotriphenylsilane (FTPS)

Solutions of FTPS in THF

Two solutions were prepared in the glovebox by weighing FTPS and NAPH into 1 mL volumetric flasks and dissolving them in THF up to the 1 mL mark. The concentrations in the solutions are presented in Table S3.9. The solutions were transferred to J Young® valve capped NMR tubes. The more concentrated solution was then diluted twice, by a factor of ~2 each time (*i.e.* by a factor ~2 and ~4 with respect to the original solution; ~100 mᴍ and ~50 mᴍ FTPS, respectively), by adding THF up to the 1 mL mark of the volumetric flask containing ~0.5 mL of the solution, mixing the solution thoroughly, transferring ~0.5 mL of the new solution to a fresh J Young® valve capped NMR tube, and repeating the process (both dilutions were performed in the glovebox). For each solution, ^{19}F pulse-acquire (8 scans) and ^{19}F inversion-recovery experiments were run at 300 K. The variable delays used in the experiments were, τ (s) {1, 3, 5, 7, 10, 15, 20, 30, 40, 50, 60, 70}.

Table S3.9: Concentrations of FTPS and NAPH in solutions.

Entry	Concentration (mᴍ)	
	FTPS	NAPH
1	9.48[a]	61.2
2	209[b]	472

Based on the ^{19}F pulse-acquire spectra, the chemical shift of FTPS (referenced to NAPH), δ^{FTPS} = (–169.76 ± 0.01) ppm, is independent of its concentration.

The determined ^{19}F longitudinal relaxation fitting parameters (I_{eq}, x and T_1) and root mean square errors (R) are presented in Table S3.10. The plots of experimental and model fractional magnetisations, m^{FTPS}, against variable delay, τ, are presented in Figure S3.14. Although there is a decreasing trend of T_1^{FTPS} with increased concentration, a concentration change from 10 mᴍ to 210 mᴍ results in a 10% decrease in T_1^{FTPS}. For comparison, a 40% decrease in T_1^{TBAT} is observed for the same concentration change (Section S3.3.1). Hence, T_1^{FTPS} can be assumed independent of concentration and approximated to the average of the four values, (12.0 ± 0.5) s.

[a] [FTPS] = 10.4 mᴍ based on integral ratio of FTPS and NAPH in ^{19}F pulse-acquire NMR experiment.
[b] [FTPS] = (209 ± 2) mᴍ based on integral ratios of FTPS and NAPH in ^{19}F pulse-acquire NMR experiments.

Table S3.10: Longitudinal relaxation fitting parameters and root mean square errors of FTPS in THF.

Entry	[FTPS] (mM)[a]	$10^{-3}I_{eq}^{FTPS}$	x^{FTPS}	T_1^{FTPS} (s)	$10^{-3}R^{FTPS}$
1	10	1.91	1.87	12.4	0.01
2	210	4.92	1.87	11.2	0.02
2, *d.f.* ~2	100	1.87	1.86	12.0	0.01
2, *d.f.* ~4[b]	50	1.29	1.86	12.3	0.01

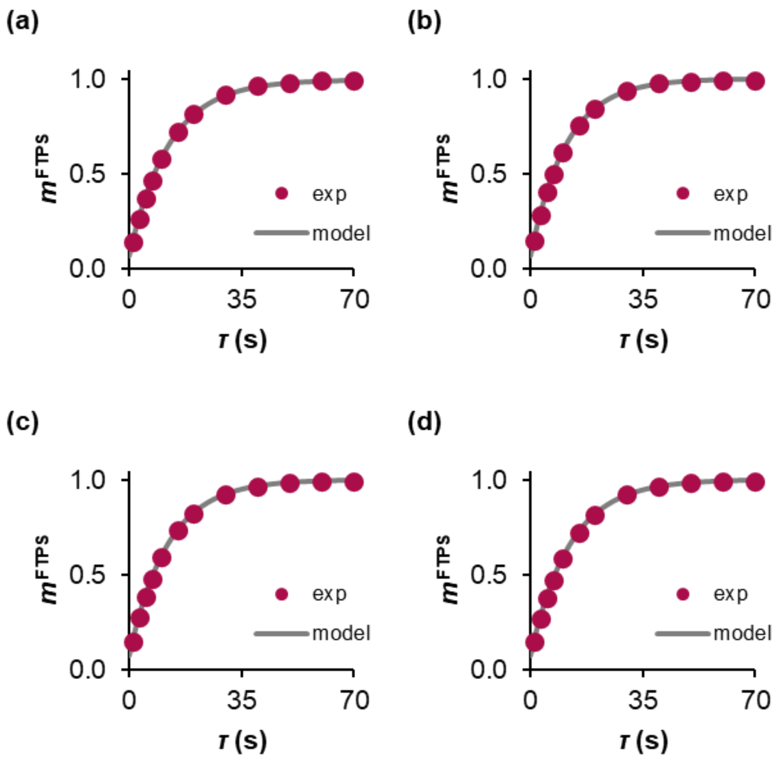

Figure S3.14: Plots of experimental ("exp") and model fractional magnetisations of FTPS, m^{FTPS}, in ^{19}F longitudinal relaxation constant measurements of FTPS in THF at 300 K. (a) ~10 mM; (b) ~210 mM; (c) ~100 mM; (d) ~50 mM.

<u>Solutions of FTPS in MeCN</u>

A solution was prepared in the glovebox by weighing FTPS (41.3 mM) and NAPH (182 mM) into a 1 mL volumetric flask and dissolving them in MeCN up to the 1 mL mark. The solution was transferred to a J Young® valve capped NMR tube. ^{19}F pulse-acquire and ^{19}F inversion-recovery experiments were run at 300 K. The solution was then titrated with three solid aliquots of FTPS (all titrations were performed in the glovebox). ^{19}F pulse-acquire and ^{19}F inversion-recovery experiments were run (at 300 K) after each aliquot addition. The variable delays used in the experiments were, *τ* (s) {1, 2, 3, 5, 7, 10, 15, 20, 30, 45, 60, 80}.

[a] To the nearest 10 mM.
[b] 2 scans in ^{19}F inversion-recovery experiment for T_1^{FTPS}.

Based on the ^{19}F pulse-acquire spectra, the chemical shift of FTPS (referenced to NAPH), δ^{FTPS} = (–169.07 ± 0.01) ppm, is independent of its concentration.

The determined ^{19}F longitudinal relaxation fitting parameters (I_{eq}, x and T_1) and root mean square errors (R) are presented in Table S3.11. The plots of experimental and model fractional magnetisations, m^{FTPS}, against variable delay, τ, are presented in Figure S3.15. T_1^{FTPS} = (16.6 ± 1.0) s was therefore found independent of [FTPS] in the given concentration range.

Table S3.11: Longitudinal relaxation fitting parameters and root mean square errors of FTPS in MeCN.

Entry	[FTPS] (mM)	$10^{-3}I_{eq}^{FTPS}$	x^{FTPS}	T_1^{FTPS} (s)	$10^{-3}R^{FTPS}$
1	40.2	0.846	1.90	16.1	0.011
1, aliq. 1	63.1	1.29	1.90	15.4	0.02
1, aliq. 2	105	2.16	1.87	17.8	0.01
1, aliq. 3	164	3.34	1.87	17.0	0.03

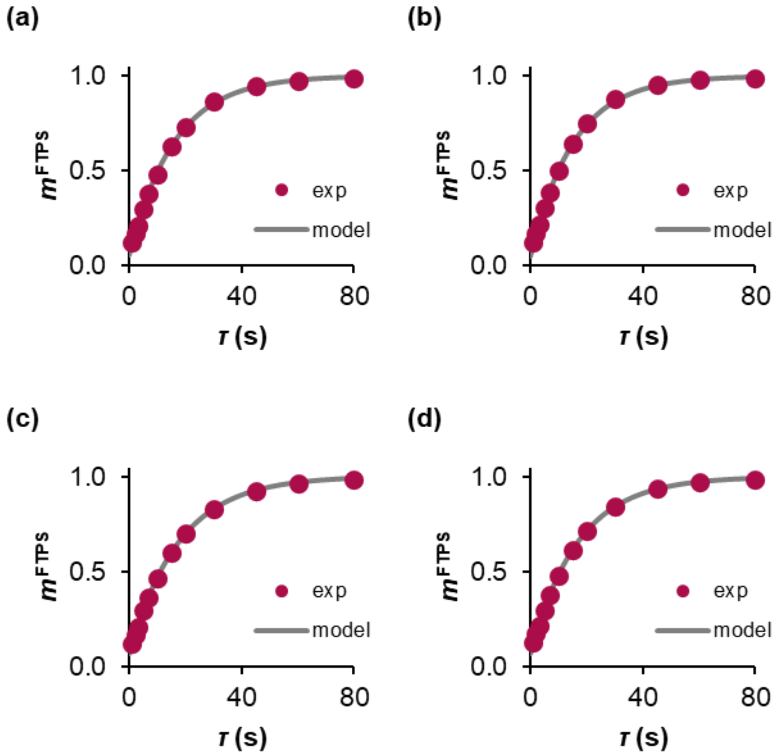

Figure S3.15: Plots of experimental ("exp") and model fractional magnetisations of FTPS, m^{FTPS}, in ^{19}F longitudinal relaxation constant measurements of FTPS in MeCN at 300 K. (a) 40.2 mM; (b) 63.1 mM; (c) 105 mM; (d) 164 mM.

S3.3.3. 2-Naphthalenyl fluorosulfate (ARSF)

Solutions of ARSF in THF

A solution was prepared in the glovebox by weighing ARSF (51.5 mM) and NAPH (118 mM) into a 1 mL volumetric flask and dissolving them in THF up to the 1 mL mark. The solution was transferred to a J Young® valve capped NMR tube. The solution was then diluted twice,

by a factor of ~2 each time (*i.e.* by a factor ~2 and ~4 with respect to the original solution; ~25 mM and ~15 mM ARSF, respectively), by adding THF up to the 1 mL mark of the volumetric flask containing ~0.5 mL of the original solution, mixing the solution thoroughly, transferring ~0.5 mL of the new solution to a fresh J Young® valve capped NMR tube, and repeating the process (both dilutions were performed in the glovebox). For each solution, ^{19}F pulse-acquire and ^{19}F inversion-recovery experiments were run at 300 K. The variable delays used in the experiments were, τ (s) {0.1, 0.2, 0.5, 0.75, 1, 2, 3, 4, 6, 8, 10, 12}.

Based on the ^{19}F pulse-acquire spectra, the chemical shift of ARSF (referenced to NAPH), δ^{ARSF} = (37.46 ± 0.01) ppm, and the isotope shift, $^{1}\Delta^{19}F(^{32}S,^{34}S)$ = (43.6 ± 0.0) ppb, are independent of concentration.

The determined ^{19}F longitudinal relaxation fitting parameters (I_{eq}, x and T_1) and root mean square errors (R) are presented in Table S3.12. The plots of experimental and model fractional magnetisations, m^{ARSF}, against variable delay, τ, are presented in Figure S3.16. T_1^{ARSF} = (2.14 ± 0.01) s was therefore found independent of [ARSF] in the given concentration range.

Table S3.12: Longitudinal relaxation fitting parameters and root mean square errors of ARSF in THF.

[ARSF] (mM)[a]	I_{eq}^{ARSF}	x^{ARSF}	T_1^{ARSF} (s)	R^{ARSF}
50	1219	1.83	2.14	9
25	688	1.84	2.15	5
15	288	1.85	2.14	2

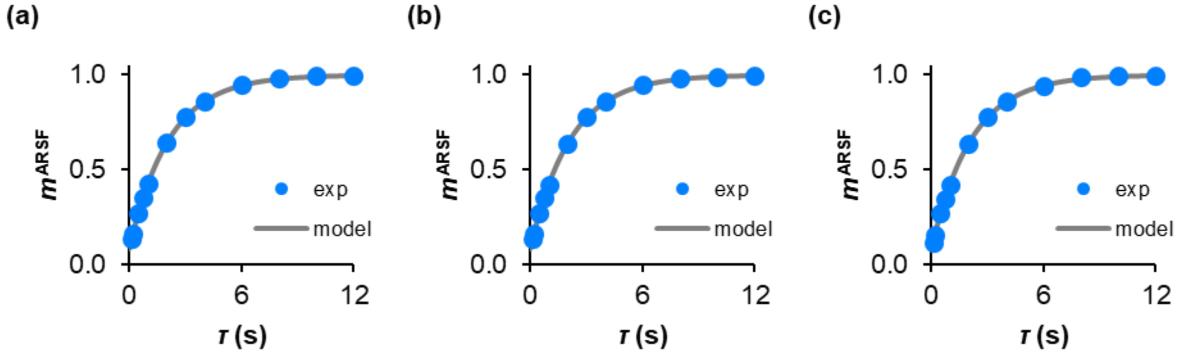

Figure S3.16: Plots of experimental and model fractional magnetisations of ARSF, m^{ARSF}, in ^{19}F longitudinal relaxation constant measurements of ARSF in THF at 300 K. (a) ~50 mM; (b) ~25 mM; (c) ~15 mM.

<u>Solutions of ARSF in MeCN</u>

Two solutions were prepared in the glovebox by weighing ARSF and NAPH into 1 mL volumetric flasks and dissolving them in MeCN up to the 1 mL mark. The concentrations in the solutions are presented in Table S3.13. The solutions were transferred to J Young® valve

[a] To the nearest 5 mM.

capped NMR tubes. The more concentrated solution was then diluted by a factor of ~2 (~200 mᴍ ARSF), by adding MeCN up to the 1 mL mark of the volumetric flask containing ~0.5 mL of the solution, mixing the solution thoroughly and transferring ~0.5 mL of the new solution to a fresh J Young® valve capped NMR tube (in the glovebox). For each solution, ^{19}F pulse-acquire and ^{19}F inversion-recovery experiments were run at 300 K. The variable delays used in the experiments were, τ (s) {0.2, 0.5, 1, 1.5, 2, 3, 5, 7.5, 10, 12.5, 15, 20}.

Table S3.13: Concentrations of ARSF and NAPH in solutions.

Entry	Concentration (mᴍ)	
	ARSF	NAPH
1	43.0[a]	64.3
2	394[b]	281

Based on the ^{19}F pulse-acquire spectra, the chemical shift of ARSF (referenced to NAPH), δ^{ARSF} = (38.06 ± 0.04) ppm (a very small linear change of the chemical shift was observed; this is however negligible considered its magnitude and the concentration range and may be due to experimental/analysis error), and the isotope shift, $^{1}\Delta^{19}F(^{32}S,^{34}S)$ = (43.6 ± 0.0) ppb, are independent of concentration.

The determined ^{19}F longitudinal relaxation fitting parameters (I_{eq}, x and T_1) and root mean square errors (R) are presented in Table S3.14. The plots of experimental and model fractional magnetisations, m^{ARSF}, against variable delay, τ, are presented in Figure S3.17. Although there seems to be a decreasing trend of T_1^{ARSF} with increased concentration, a concentration change from 40 mᴍ to 390 mᴍ results in a 5% decrease in T_1^{ARSF}, which is negligible considering the concentration range. Hence, T_1^{ARSF} can be approximated to the average of the three values, (2.49 ± 0.07) s.

Table S3.14: Longitudinal relaxation fitting parameters and root mean square errors of ARSF in MeCN.

Entry	[ARSF] (mᴍ)[c]	I_{eq}^{ARSF}	x^{ARSF}	T_1^{ARSF} (s)	R^{ARSF}
1	40	1598	1.87	2.55	5
2	390	13038	1.86	2.42	11
2, *d.f.* ~2	200	7577	1.86	2.50	19

[a] [ARSF] = 41.8 mᴍ based on integral ratio of ARSF and NAPH in ^{19}F pulse-acquire NMR experiment.
[b] [ARSF] = (393 ± 0) mᴍ based on integral ratios of ARSF and NAPH in ^{19}F pulse-acquire NMR experiments.
[c] To the nearest 10 mᴍ.

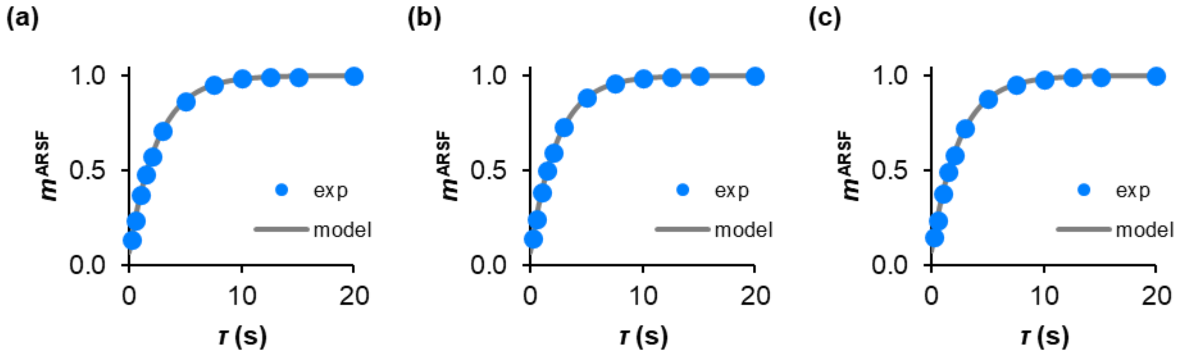

Figure S3.17: Plots of experimental ("exp") and model fractional magnetisations of ARSF, m^{ARSF}, in ^{19}F longitudinal relaxation constant measurements of ARSF in MeCN at 300 K. (a) ~40 mM; (b) ~390 mM; (c) ~200 mM.

S4. Speciation of TBAT

S4.1. ^{1}H diffusion analysis

^{1}H diffusion analyses of the appropriate spins were performed using a longitudinal-eddy-current delay double stimulated-echo pulse sequence with a bipolar gradient pulse pair and two spoil gradients (ledbpgp2s; SINE.100 gradient pulses and spoil gradients, duration of gradient pulse: $\delta/2$ variable between experiments, duration of spoil gradient: 600 µs).[S4] The delay for gradient recovery and eddy current suppression were of 200 µs and 5.00 ms, respectively. The diffusion time, Δ, and duration of gradient pulse, $\delta/2$, were optimised for each experiment and are given where appropriate. The pulse sequence was repeated for a series of 16 gradient strengths, g (G/mm) {0.170, 0.851, 1.19, 1.45, 1.68, 1.87, 2.05, 2.21, 2.37, 2.51, 2.64, 2.77, 2.89, 3.01, 3.13, 3.24}. A relaxation delay $D_1 \geq 5T_1 - t_{acq}$ (acquisition time, t_{acq} = 4 s) was used to achieve at least 99.3% recovery of the longitudinal magnetisations of the spins, where T_1 was the longest of the longitudinal relaxation time constants of the spins of interest in a given system. The experiments were performed with 4 dummy scans. For solutions in THF-d_8 and DMSO-d_6 – 8 scans were acquired, and for solutions in MeCN-d_3 – 4 scans (due to significantly longer longitudinal relaxation).

Integration of the appropriate signals was performed manually. To the experimental integrals at each gradient strength was fitted a two-parameter (I_0, D) model as presented in Equation S4.1, where I_g is the integral at gradient strength g, I_0 is the integral at gradient strength g = 0, $\gamma = 2.68 \cdot 10^4$ rad s^{-1} G^{-1} is the magnetogyric ratio of ^{1}H and D is the translational diffusion coefficient. The experimental and model integrals were then normalised by dividing them by the determined I_0.

S4.1

$$I_g = I_0 e^{-(\gamma g \delta)^2\left(\Delta - \frac{\delta}{3}\right)D}$$

S4.1.1. Relative diffusion coefficients of $Ph_3SiF_2^-$ and $^nBu_4N^+$

Six solutions were prepared in the glovebox by weighing variable amounts of TBAT into vials and dissolving each sample in either THF-d_8 or MeCN-d_3 (~0.5 mL). The solutions were transferred to J Young® valve capped NMR tubes. For each solution, ^{1}H inversion-recovery and ^{1}H diffusion analysis experiments were run at three temperatures: 300 K, 310 K and 320 K for solutions in THF-d_8; 300 K, 320 K and 335 K for solutions in MeCN-d_3. All signals corresponding to $Ph_3SiF_2^-$ ("–") were integrated together, as were ones corresponding to $^nBu_4N^+$ ("+"). The determined ^{1}H diffusion fitting parameters (I_0 and D) and root mean square errors (R) for $Ph_3SiF_2^-$ and $^nBu_4N^+$ are presented in Table S4.1. The experimental and model I_g^-/I_0^- and I_g^+/I_0^+ against g are presented in Figure S4.1 (THF-d_8) and Figure S4.2 (MeCN-d_3).

Table S4.1: ^{1}H diffusion fitting parameters and root mean square errors in ^{1}H diffusion analyses of $Ph_3SiF_2^-$ ("–") and $^nBu_4N^+$ ("+").

Solvent	[TBAT] (mM)[a]	T (K)	Δ (ms), δ (ms)	$10^{-8}I_0$		D (mm^2/h)		$10^{-8}R$	
				–	+	–	+	–	+
THF-d_8	220	300	100, 2.20	214	430	2.19	2.20	3	6
THF-d_8	220	310	75.0, 2.20	199	421	4.07	4.13	2	5
THF-d_8	220	320	75.0, 2.20	201	430	3.53	3.60	12	25
THF-d_8	110	300	60.0, 2.20	205	440	4.96	5.05	1	1
THF-d_8	110	310	75.0, 2.20	189	404	6.85	7.04	3	4
THF-d_8	110	320	60.0, 2.00	189	418	19.4	19.2	2	5
THF-d_8	30	300	75.0, 2.20	190	409	3.15	3.21	1	1
THF-d_8	30	310	75.0, 2.20	180	393	3.76	3.84	1	3
THF-d_8	30	320	60.0, 2.00	193	434	12.1	12.1	1	2
MeCN-d_3	220	300	75.0, 2.20	112	248	4.85	4.65	1	3
MeCN-d_3	220[b]	320	75.0, 1.60	101	238	11.7	11.8	3	6
MeCN-d_3	220	335	75.0, 1.60	98.5	237	21.2	21.9	5.7	14
MeCN-d_3	120	300	75.0, 2.20	103	233	5.27	5.08	1	3
MeCN-d_3	120	320	75.0, 1.60	98.9	230	12.3	12.1	0.8	2
MeCN-d_3	120	335	75.0, 1.60	117	277	61.4	61.8	3	7
MeCN-d_3	40	300	60.0, 2.00	94.5	216	5.62	5.44	0.6	1
MeCN-d_3	40[c]	320	60.0, 2.00	93.7	222	8.34	7.97	0.6	2
MeCN-d_3	40[d]	335	60.0, 2.00	109	261	37.3	36.8	2	5

[a] To the nearest 10 mM.
[b] 2 scans in ^{1}H diffusion analysis experiment.
[c] For unknown reason, ^{1}H diffusion analysis experiment failed to run for $D_1 \geq 5T_1^- - 4.0$ s (53.9 s); D_1 was therefore decreased to 44.0 s, which resolved the issue and allowed for 98.4% longitudinal relaxation of $Ph_3SiF_2^-$.
[d] For unknown reason, ^{1}H diffusion analysis experiment failed to run for $D_1 \geq 5T_1^- - 4.0$ s (61.0 s); D_1 was therefore decreased to 44.0 s, which resolved the issue and allowed for 97.5% longitudinal relaxation of $Ph_3SiF_2^-$.

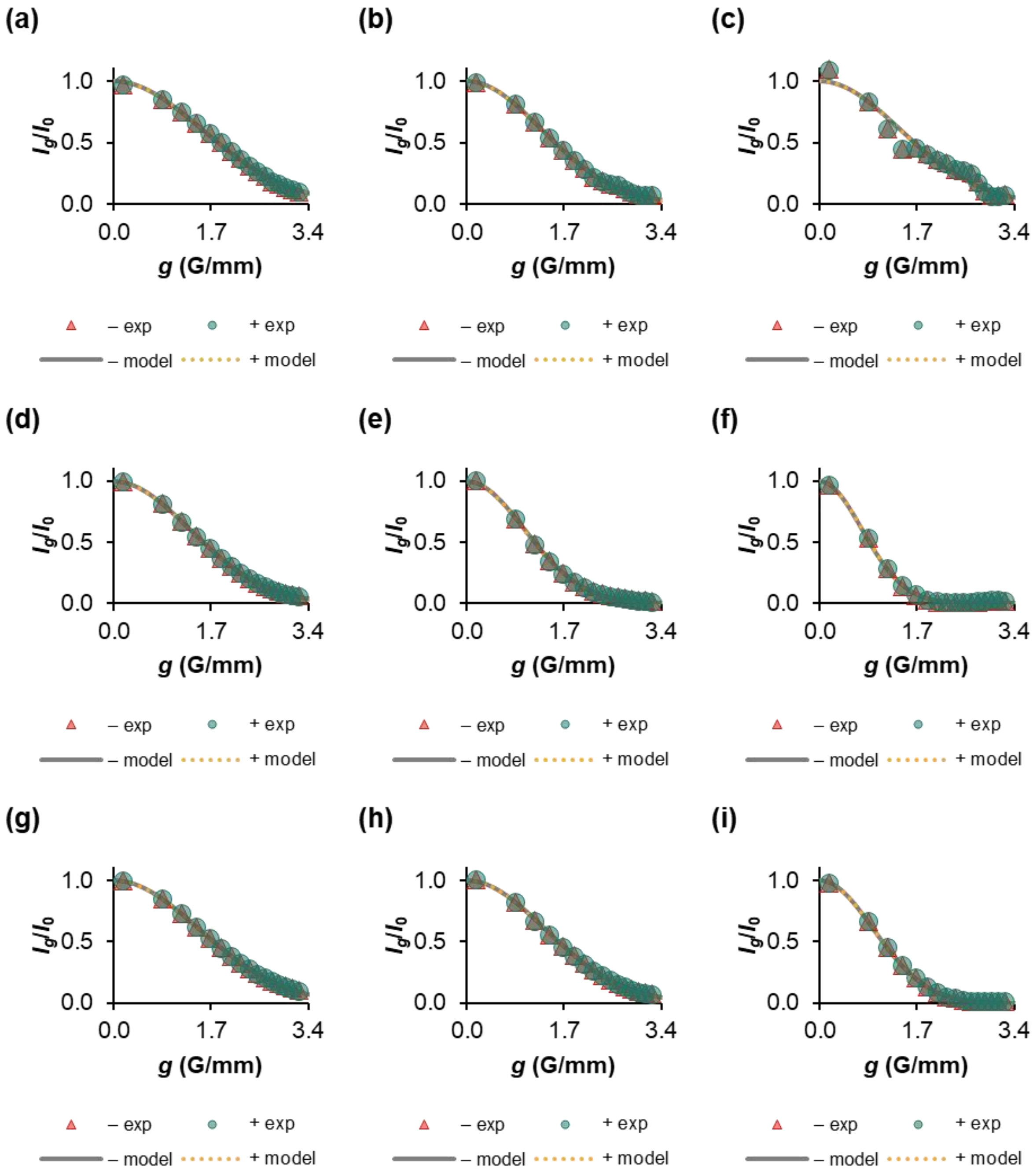

Figure S4.1: Plots of experimental ("exp") and model I_g/I_0 of $Ph_3SiF_2^-$ ("–") and $^nBu_4N^+$ ("+") in ^1H diffusion analysis of TBAT in THF-d_8. (a) 220 mM at 300 K; (b) 220 mM at 310 K; (c) 220 mM at 320 K; (d) 110 mM at 300 K; (e) 110 mM at 310 K; (f) 110 mM at 320 K; (g) 30 mM at 300 K; (h) 30 mM at 310 K; (i) 30 mM at 320 K.

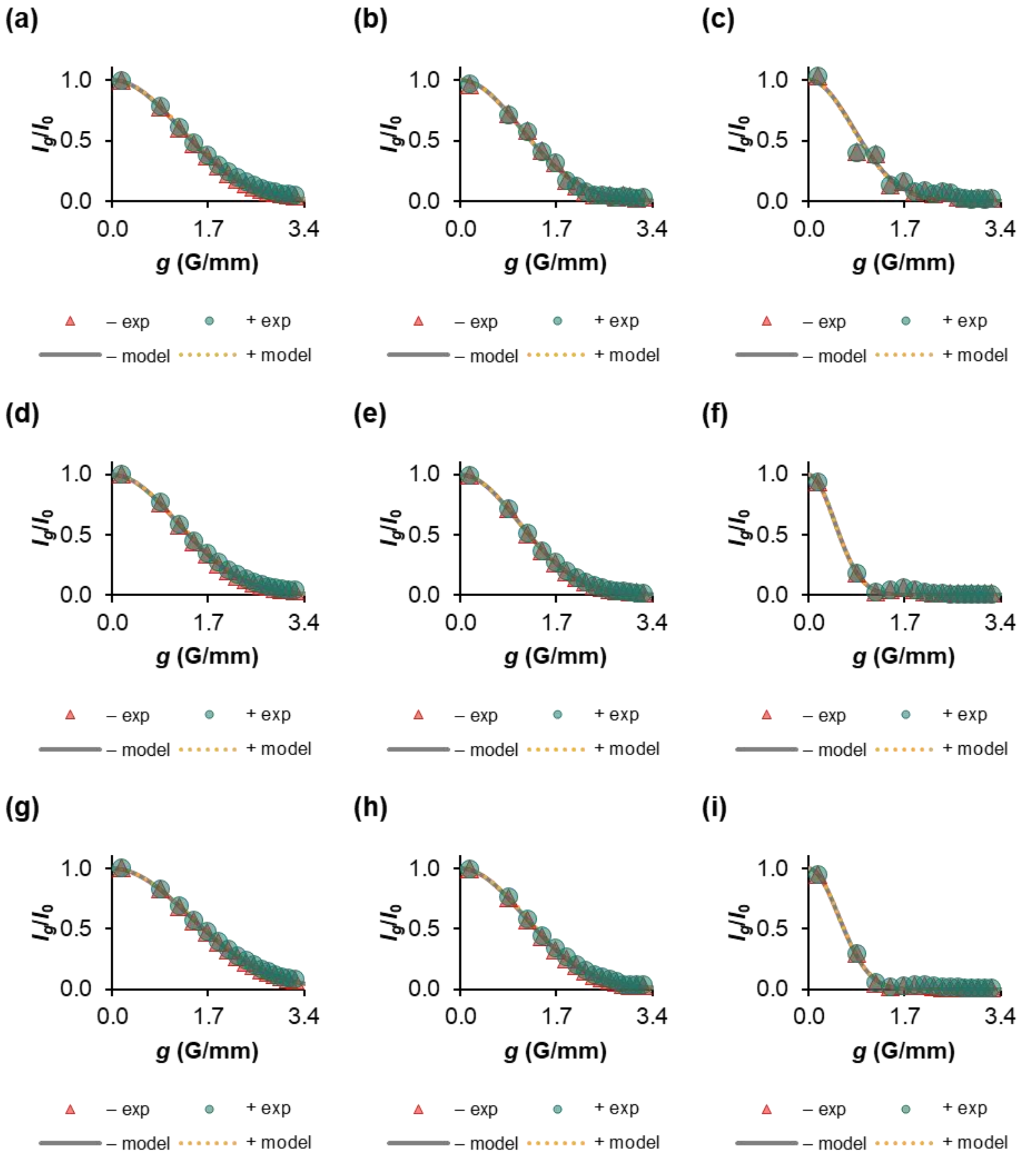

Figure S4.2: Plots of experimental (“exp”) and model I_g/I_0 of $Ph_3SiF_2^–$ (“–”) and $^nBu_4N^+$ (“+”) in ^1H diffusion analysis of TBAT in MeCN-d_3. (a) 220 mM at 300 K; (b) 220 mM at 320 K; (c) 220 mM at 335 K; (d) 120 mM at 300 K; (e) 120 mM at 320 K; (f) 120 mM at 335 K; (g) 40 mM at 300 K; (h) 40 mM at 320 K; (i) 40 mM at 335 K.

The translational diffusion coefficients of $Ph_3SiF_2^–$ relative to $^nBu_4N^+$, $D^–/D^+$, are reported for each dataset in Table S4.2. The determined ratio $D^–/D^+$ is nearly unity across all entries in Table S4.2 (average of 0.987 ± 0.008 in THF-d_8 and 1.02 ± 0.02 in MeCN-d_3), indicating strong ion pairing of $Ph_3SiF_2^–$ and $^nBu_4N^+$ in solution under these conditions, provided that the ions do not coincidentally exhibit identical diffusive properties.

Table S4.2: The ratios of translational diffusion coefficients of $Ph_3SiF_2^-$ and $^nBu_4N^+$, D^-/D^+, in THF-d_8 and MeCN-d_3.

Solvent	[TBAT] (mM)	Temperature (K)	D^-/D^+
THF-d_8	220	300	0.994
THF-d_8	220	310	0.984
THF-d_8	220	320	0.981
THF-d_8	110	300	0.980
THF-d_8	110	310	0.973
THF-d_8	110	320	1.01
THF-d_8	30	300	0.981
THF-d_8	30	310	0.980
THF-d_8	30	320	1.00
MeCN-d_3	220	300	1.04
MeCN-d_3	220	320	0.986
MeCN-d_3	220	335	0.971
MeCN-d_3	120	300	1.04
MeCN-d_3	120	320	1.01
MeCN-d_3	120	335	0.994
MeCN-d_3	40	300	1.03
MeCN-d_3	40	320	1.05
MeCN-d_3	40	335	1.01

S4.1.2. Diffusion coefficients of $Ph_3SiF_2^-$ and $^nBu_4N^+$, relative to internal standard

Two solutions were prepared in the glovebox by weighing TBAT and TMB into 1 mL volumetric flasks and dissolving them in DMSO-d_6 and THF-d_8, respectively, up to the 1 mL mark. The solutions were transferred to J Young® valve capped NMR tubes. For each solution ^{1}H inversion-recovery and ^{1}H diffusion analysis experiments were run at 300 K. All signals corresponding to $Ph_3SiF_2^-$ ("–") were integrated together, as were ones corresponding to $^nBu_4N^+$ ("+"); except for the solution of TBAT in DMSO-d_6, where the most downfield signal corresponding to $^nBu_4N^+$ was excluded due to proximity to the residual solvent signal. The methyl (CH_3) signal of TMB was used as internal diffusion standard (due to comparable longitudinal relaxation time to TBAT). The determined ^{1}H diffusion fitting parameters (I_0 and D) and root mean square errors (R) of $Ph_3SiF_2^-$, $^nBu_4N^+$ and TMB (CH_3) are presented in Table S4.3. The experimental and model I_g^-/I_0^-, I_g^+/I_0^+ and I_g^{TMB}/I_0^{TMB} against g are presented in Figure S4.3.

Table S4.3: ^{1}H iffusion fitting parameters and root mean square errors in ^{1}H diffusion analysis of $Ph_3SiF_2^-$ ("–"), $^nBu_4N^+$ ("+") and TMB (CH_3).

Solvent	[TBAT] (mM)	T (K)	Δ (ms), δ (ms)	$10^{-8}I_0$		D (mm²/h)		$10^{-8}R$	
				–	+	–	+	–	+
DMSO-d_6[a]	1.5[b]	300	100, 4.00	0.403	0.797	1.20	0.994	0.005	0.008
THF-d_8[c]	207[d]	300	100, 2.20	59.0	117	2.52	2.51	0.5	1

[a] 263 mM TMB: $10^{-8}I_0^{TMB}$ = 48.8, D_T^{TMB} = 1.70 mm²/h, $10^{-8}R^{TMB}$ = 0.3.
[b] The concentration of TBAT is given with two significant figures due to limit of balance accuracy.
[c] 252 mM TMB: $10^{-8}I_0^{TMB}$ = 43.4, D_T^{TMB} = 5.39 mm²/h, $10^{-8}R^{TMB}$ = 0.4.
[d] 4 scans in ^{1}H diffusion analysis experiment.

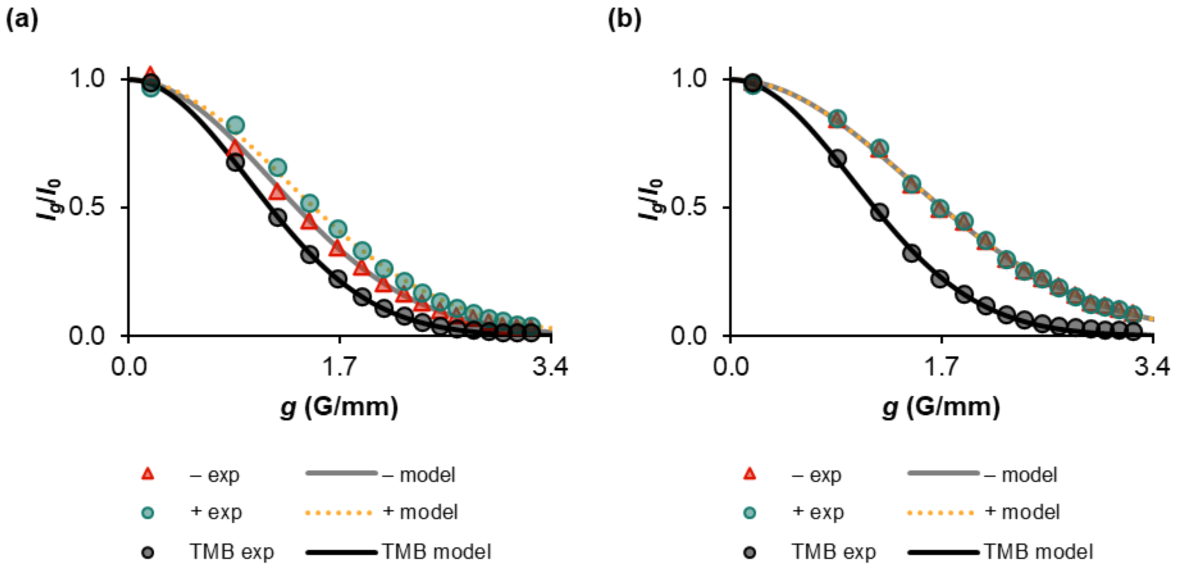

Figure S4.3: Plots of experimental ("exp") and model I_g/I_0 of $Ph_3SiF_2^-$ ("–"), $^nBu_4N^+$ ("+") and TMB (CH_3) in ^1H diffusion analysis of (a) 1.5 mM TBAT in DMSO-d_6 and (b) 207 mM TBAT in THF-d_8 (both at 300 K).

The ratio of diffusion coefficients in the solution in DMSO-d_6 showed $D^-/D^+ = 1.21$. This value is indicative of vastly different diffusive properties of $Ph_3SiF_2^-$ and $^nBu_4N^+$ in dilute solutions of TBAT in DMSO-d_6. This result confirms that the identical diffusion properties of the cation and anion in THF-d_8 and MeCN-d_3 were indeed a result of ion pairing rather than similar diffusion coefficients of the separate ions in these solutions. Moreover, the diffusion coefficients of $Ph_3SiF_2^-$ and $^nBu_4N^+$ relative to TMB were $D_-/D_{TMB} = 0.710$ and $D_+/D_{TMB} = 0.586$, respectively, in 1.5 mM TBAT in DMSO-d_6. These values are significantly higher than $D_-/D_{TMB} = 0.467$ and $D_+/D_{TMB} = 0.466$ for 207 mM TBAT in THF-d_8.

S4.1.3. Diffusion coefficients of BARF$^-$ and ^{n}Bu$_4$N$^+$, relative to internal standard

A solution was prepared in the glovebox by weighing TBABARF and TMB into a 1 mL volumetric flask and dissolving them in THF-d_8 up to the 1 mL mark. The solution was transferred to a J Young® valve capped NMR tube. ^1H inversion-recovery and ^1H diffusion analysis experiments were run at 300 K. All signals corresponding to BARF$^-$ ("–") were integrated together, as were ones corresponding to $^nBu_4N^+$ ("+"). The methyl (CH_3) signal of TMB was used as internal diffusion standard (due to comparable longitudinal relaxation time to TBABARF). The determined ^1H diffusion fitting parameters (I_0 and D) and root mean square errors (R) of BARF$^-$, $^nBu_4N^+$ and TMB (CH_3) are presented in Table S4.4. The experimental and model I_g^-/I_0^-, I_g^+/I_0^+ and I_g^{TMB}/I_0^{TMB} against g are presented in Figure S4.4.

Table S4.4: ^{1}H diffusion fitting parameters and root mean square errors in ^{1}H diffusion analysis of BARF$^-$ ("–"), ^{n}Bu$_4$N$^+$ ("+") and TMB (CH$_3$).

Solvent	[TBABARF] (mM)	*T* (K)	Δ (ms), δ (ms)	$10^{-8}I_0$		*D* (mm^2/h)		$10^{-8}R$	
				–	+	–	+	–	+
THF-d_8[a]	201[b]	300	100, 2.20	36.7	86.7	2.78	2.99	0.7	1.6

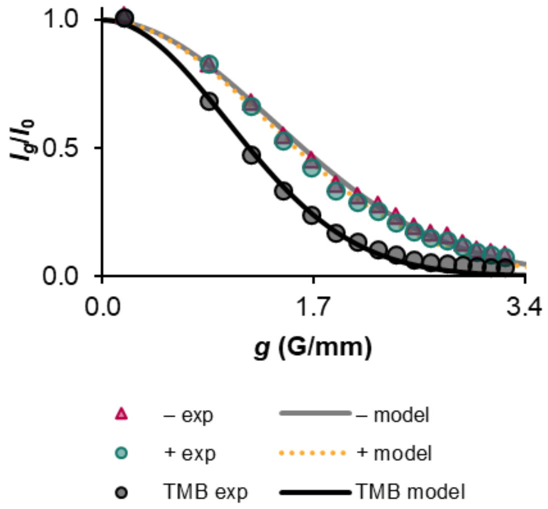

Figure S4.4: Plots of experimental ("exp") and model I_g/I_0 of BARF$^-$ ("–"), ^{n}Bu$_4$N$^+$ ("+") and TMB (CH$_3$) in ^{1}H diffusion analysis of TBABARF in THF-d_8 (201 mM TBABARF, 256 mM TMB; 300 K).

The diffusion analysis of TBABARF gave D^-/D^+ = 0.931, indicating dissociation of the salt in THF. Moreover, D_+/D_{TMB} = 0.576 TBABARF in THF-d_8 is nearly identical to the D_+/D_{TMB} of 0.586 for ^{n}Bu$_4$N$^+$ in 1.5 mM TBAT in DMSO-d_6.

S4.2. ^{1}H 1D NOESY

^{1}H 1D NOESY experiments were performed for two samples prepared analogously to those used for diffusion analysis (TBAT in THF-d_8 and MeCN-d_3, ~210 mM in each), using a standard 1D selective gradient NOESY pulse sequence (selnogp) with two gradient pulses in the selective refocusing period and two gradient pulses in the mixing period (SMSQ10.100, duration: 1.000 ms), a single frequency-selective refocusing π pulse in the selective refocusing period (Gaus1, duration: 8.820 ms, bandwidth: 100.0 Hz) and a single non-selective π pulse in the mixing period.[S5–S7] A delay for gradient recovery of 200 μs was used. A relaxation delay $D_1 \geq 5T_1 - t_{acq}$ was used to achieve at least 99.3% recovery of the longitudinal magnetisations of the spins, where T_1 was the longest of the longitudinal relaxation time constants of the spins of interest in a given system (7 s for the sample in THF-d_8 and 30 s for one in MeCN-d_3). A mixing time of 0.5 s was used for THF-d_8 and 1.0 s for MeCN-d_3. The spectra were acquired

[a] 256 mM TMB: $10^{-8}I_0^{TMB}$ = 36.6, D_T^{TMB} = 5.19 mm^2/h, $10^{-8}R^{TMB}$ = 0.8.
[b] 4 scans in ^{1}H diffusion analysis experiment.

with t_{acq} = 4.0 s, 4 dummy scans and 128 scans. The centre frequencies for selective refocusing were 7.10 ppm and 8.03 ppm (in two separate experiments for each sample).

The resulting spectra are presented in Figure S4.5, which shows the presence of ion pairing in both solvents (selective perturbation of either aromatic proton signal resulted in NOE to the aliphatic protons, and the remaining aromatic protons).

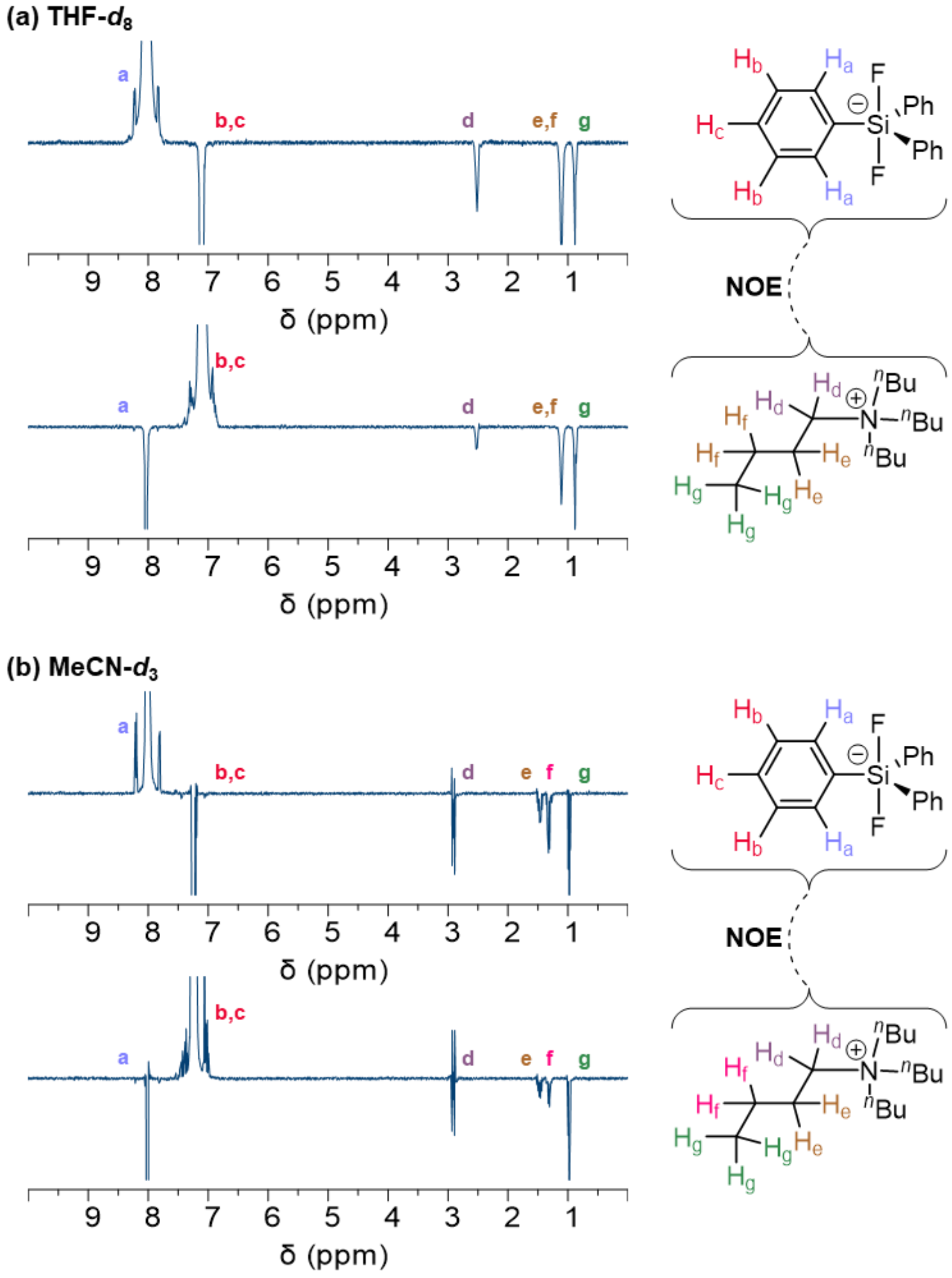

Figure S4.5: **^{1}H NOE spectra showing evidence for interactions between aromatic and aliphatic protons in solutions of 210 mM TBAT in (a) THF-*d*$_8$ and (b) MeCN-*d*$_3$ (at 300 K for both solutions). A structure of the molecular unit of TBAT is included with the NOE contacts between $Ph_3SiF_2^-$ and $^nBu_4N^+$ indicated by dashed lines. Due to the overlap of *meta*- and *para*- protons in ^{1}H NMR spectra of the salt in both solvents, the exact NOE contacts for these spins are unclear. In THF-*d*$_8$, two CH_2 environments overlap, and therefore the contacts corresponding to these spins are also unclear. (The initially perturbed signal is clipped in all spectra).**

S4.3. ^{19}F chemical exchange saturation transfer (CEST)

A standard CEST pulse sequence was used in ^{19}F chemical exchange saturation transfer (CEST) experiments.[S8,S9] After a relaxation delay, D_1, a series of n identical soft (frequency-selective) π/2 pulses (Gaus1, duration: 5.600 ms/pulse, bandwidth: 378.9 Hz) continuously saturated a selected frequency range over saturation time, t_{sat} = (5.600n) ms. The resulting longitudinal magnetisations were detected using a hard π/2 pulse and acquisition (with t_{acq} acquisition time). This pulse sequence was repeated for a series of frequency ranges. For each ^{19}F CEST experiment, t_{sat} was equal to the longitudinal relaxation time constant of TBAT in the given system, $t_{sat} = T_1^{TBAT}$ (1.10 s for the solutions in THF and 3.60 s – in MeCN); with n adjusted automatically to achieve this. $D_1 = 5T_1^{TBAT} - t_{acq} - t_{sat} = 4T_1^{TBAT} - t_{acq}$ was used to achieve 99.3% recovery of the longitudinal magnetisation of TBAT. The spectra were acquired with t_{acq} = 1.5 s acquisition time, 0 dummy scans, and 128 scans for the solutions in THF and 64 scans – in MeCN. The transmitter resonance was placed at –96 ppm, the spectral window was 200 ppm, and the frequency ranges were saturated from downfield to upfield.

The signal was integrated and the integrals, relative to an arbitrary baseline, plotted against the centre frequencies of saturation pulses.

Saturated solutions of TBAT in THF and MeCN

Two saturated solutions of TBAT, one in THF and one in MeCN, were prepared freshly in the glovebox by vigorously stirring an excess of solid TBAT in ~1 mL of the appropriate solvent for 15 min, and decanted into J Young® valve capped NMR tubes. ^{19}F inversion-recovery and ^{19}F CEST experiments were run at 300 K. The ^{19}F experiment was repeated for the solution in THF, with a smaller number of saturation frequencies – around –150 ppm, due to insufficient data density in the first measurement. For each sample, the absolute integrals of TBAT were normalised by dividing them by the “baseline integral”, arbitrarily chosen from data points where visually no saturation occurred. The resulting CEST plots are presented in Figure S4.6.

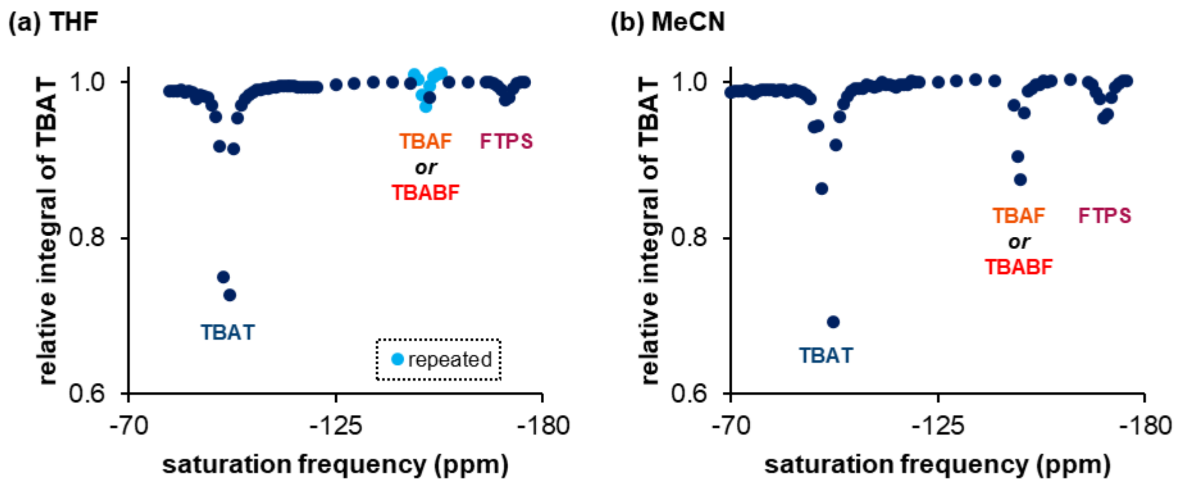

Figure S4.6: ^{19}F CEST NMR spectra of saturated solutions of TBAT in (a) THF and (b) MeCN (both at 300 K). In each solvent, a pair of signals of similar shape and size was observed at approximately –150 ppm and at –170 ppm. The upfield signal was readily identified as arising from FTPS, whereas the downfield one could correspond to TBAF or TBABF. The relative integrals of TBAT are ratios of absolute (measured) integrals of TBAT and the "baseline integral", arbitrarily chosen from data points where visually no saturation occurred.

Both experiments showed an expected large decrease of the integral of TBAT upon saturation of its resonance frequency. In each solvent, a pair of small dips of similar shape and size was also observed (more so in THF), with the relative decrease of magnetisation of TBAT significantly larger in MeCN than THF (~5–15% and ~2%, respectively). The upfield dip, at –170 ppm in both solvents, was readily assigned to FTPS. The frequency of the downfield one is –149 ppm in THF and –147 ppm in MeCN. It is unclear whether the downfield dip corresponds to TBAF, the chemical shift of which is significantly dependent on the conditions, according to literature reports. Anhydrous TBAF resonates at –86 ppm and –72 ppm in THF and MeCN-d_3, respectively, and the signal moves upfield as the water content in the solution is increased.[S10] The upfield extreme of the chemical shift of TBAF in these two is not known. Alternatively, this signal could correspond to tetra-*n*-butylammonium bifluoride (TBABF), which forms in equilibrium from TBAF in the presence of any adventitious water (Scheme S4.1; δ_F^{TBABF} = –147 ppm, THF and MeCN-d_3).[S10] Nevertheless, according to Scheme S4.1, any reversible formation of TBABF in the solutions would have had to occur *via* TBAF.[S10]

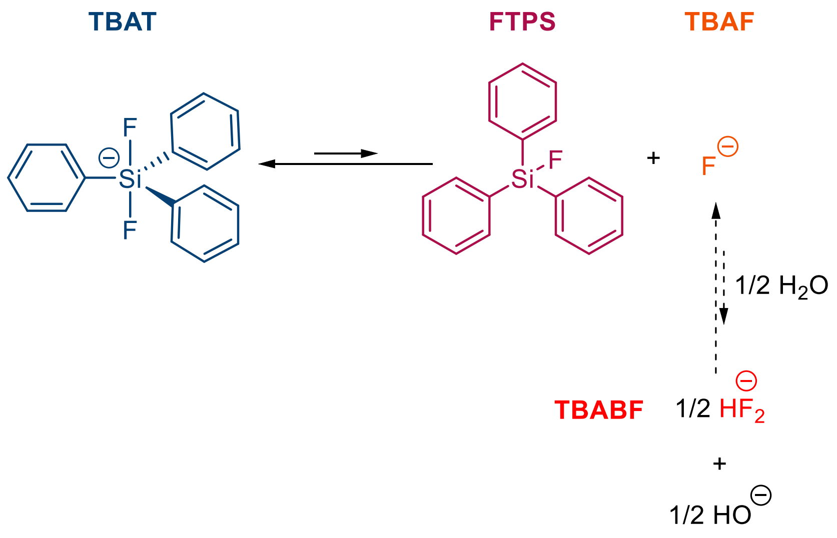

Scheme S4.1: A proposed scheme for the dissociation of TBAT in THF and MeCN. In the presence of water, TBABF may form in equilibrium from TBAF.[S10] (The tetra-*n*-butylammonium counterion was omitted for clarity).

Saturated solutions of TBAT in THF and MeCN in the presence of exogenous FTPS

Two saturated solutions of TBAT, one in THF and one in MeCN, were prepared freshly in the glovebox by vigorously stirring an excess of solid TBAT in ~1 mL of the appropriate solvent for 15 min, and decanted into J Young® valve capped NMR tubes. FTPS (~3 mg added to the NMR tubes, each containing ~0.5 mL of the solution; hence corresponding to ~20 mM FTPS) was added to both samples (FTPS was detected in a ^{19}F pulse-acquire NMR spectrum of the solution in THF, ~5 mol% FTPS by integration against TBAT; in MeCN, the FTPS signal was just above qualitative detection limit in the ^{19}F pulse-acquire NMR spectrum, due to very significant line broadening; the pulse-acquire NMR spectra were acquired with 16 scans). ^{19}F CEST experiments were run at 300 K. For each sample, the absolute integrals of TBAT were normalised by dividing them by the "baseline integral", analogously to above for the sample in THF. Due to saturation of the TBAT signal occurring for the entire frequency range in MeCN, the integral corresponding to the most downfield data point was selected as the arbitrary "baseline integral". The resulting CEST plots are presented in Figure S4.7.

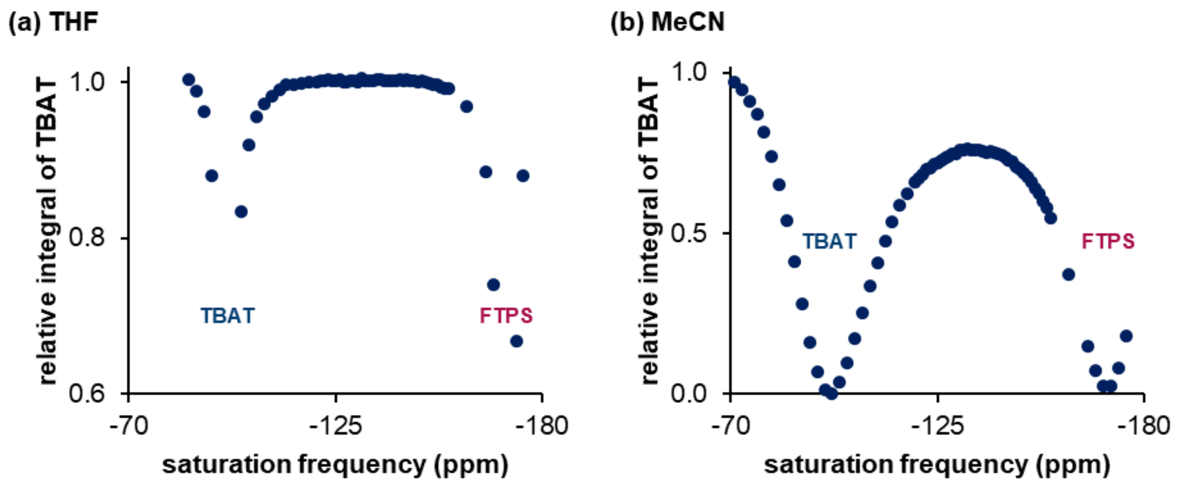

Figure S4.7: ^{19}F CEST NMR spectra of saturated solutions of TBAT in (a) THF and (b) MeCN, in the presence of exogenous FTPS (both at 300 K).

In the presence of exogenous FTPS, the dip at –170 ppm became larger for both solutions. The dips corresponding to TBAT and FTPS also exhibited broadening (or more precisely – saturation over a broader frequency range). However, in THF, the saturation transfer from the signal at –147 ppm was supressed to below the detection limit by the addition of FTPS. In MeCN, the line broadening of both TBAT and FTPS dips covered the entire saturation frequency range. Although the two major dips (TBAT and FTPS) overlap with any TBAF/TBABF previously observed at –149 ppm, no specific saturation transfer maxima were observed at that frequency, which was significant in the absence of FTPS. The suppression of saturation transfer from TBAF/TBABF by addition of FTPS to saturated TBAT in MeCN can therefore be concluded. As, according to the Le Chatelier's principle, exogenous FTPS shifts the equilibrium between the species towards TBAT, these results were expected and confirm the low degree of reversible dissociation of TBAT into FTPS and TBAF in THF and MeCN.

Saturated solutions of TBAT in THF and MeCN in the presence of molecular sieves (3Å-MS)

Two saturated solutions of TBAT, one in THF and one in MeCN, were prepared freshly in the glovebox by vigorously stirring an excess of solid TBAT in ~1 mL of the appropriate solvent for 15 min, and decanted into J Young® valve capped NMR tubes. 3Å-MS (~40 mg) were added to the samples in the NMR tubes in such a way that the sieves remained in the top compartments of the tubes (achieved with the aid of filter papers). The NMR tubes were then inverted, for the solutions inside them to come to contact with the molecular sieves, and left in such position for ~2 months. After this time, the tubes were inverted back to the "standard" position, the sieves removed from their top compartments (inside the glovebox). ^{19}F CEST experiments were run at 300 K. The absolute integrals of TBAT for the sample in THF were normalised by dividing them by the "baseline integral", chosen analogously to above for the

sample in THF. In MeCN, a new signal appeared in the ^{19}F pulse-acquire NMR spectrum (16 scans), at approximately –125 ppm and with an integral of ~0.004 relative to TBAT. The ^{19}F CEST profile shows that the group giving rise to this signal does not exchange with TBAT, as no saturation transfer was observed for –125 ppm. The “baseline integral” increased significantly over the duration of the experiment (~20 hours, saturation frequencies from downfield to upfield), and the reason for this phenomenon is unclear (the integral corresponding to the most upfield signal was used as “baseline integral”). A linear correction was therefore applied to the baseline. The resulting CEST plots are presented in Figure S4.8.

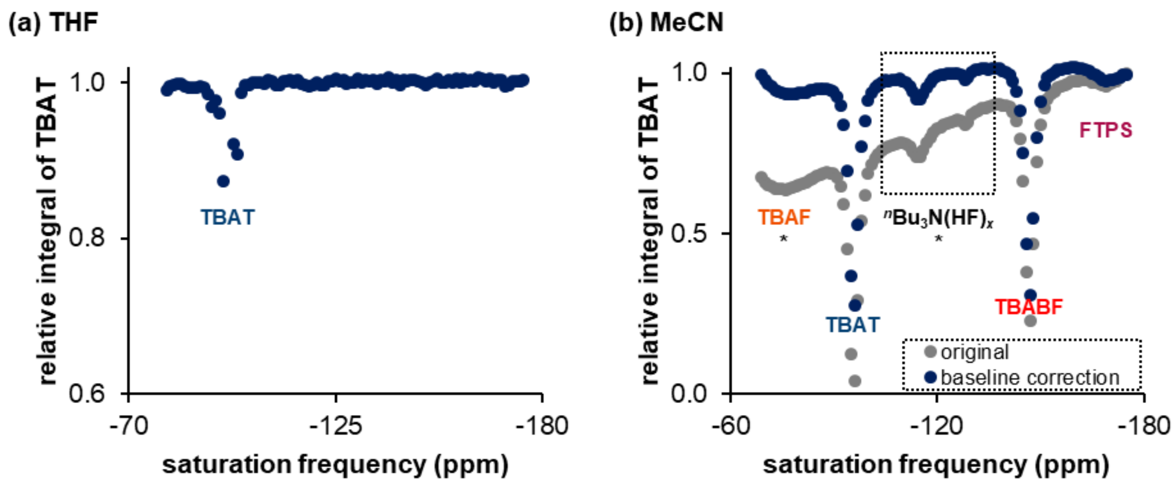

Figure S4.8: ^{19}F CEST NMR spectra of saturated solutions of TBAT in (a) THF and (b) MeCN (both at 300 K), after storage in the presence of molecular sieves. An asterisk (*) indicates a tentative assignment.

The CEST profile of the solution in THF shows the suppression of both FTPS and TBAF/TBABF signals to below the detection limit. This observation may be indicative of either (i) the equilibrium of TBAT dissociation shifting towards recombination of the dissociation products (*i.e.* the degree of TBAT dissociation decreasing), and/or (ii) the rate of saturation transfer (exchange) between the spins decreasing after storage in the presence of molecular sieves.

The corresponding solution in MeCN saw five dips apart from the saturation of TBAT itself. From downfield to upfield, the dips were found at: –77 ppm, –115 ppm, –128 ppm, –147 ppm and –169 ppm. Based on the discussion above, the dip at –169 ppm was readily assigned as FTPS. The dip at –147 ppm gave significantly more saturation transfer than for the solution in Figure S4.6b. Considering that the system was dried with molecular sieves, we confidently assigned the signal to TBABF for this solution, as any TBAF would likely move downfield due to a lesser degree of hydration. Indeed, a broad dip of similar intensity to one at –169 ppm (FTPS) was observed at –77 ppm, which is only slightly upfield from anhydrous TBAF in MeCN (–72 ppm).[S10] Therefore, we tentatively assigned this spin as TBAF. In anhydrous MeCN, the

equilibrium of TBABF formation from TBAF should be supressed and hence we propose that TBABF was formed in the solution from the decomposition of TBAF *via* Hofmann elimination,[S11] rather than from the equilibrium in Scheme S4.1. The proposed irreversible decomposition, and the resulting equilibrium network, are presented in Scheme S4.2. Analogously to the system in Scheme S4.1, TBAT dissociated reversibly into FTPS and TBAF. However, due to the enhanced basicity of the less hydrated fluoride in TBAF, the salt decomposed (to a low degree) to form tributylamine, but-1-ene and TBABF; perhaps *via* formation of HF in the first step, followed by an equilibrium reaction between HF and TBAF. The intermediacy of HF could explain the additional signals (–115 ppm and –128 ppm in CEST, and –125 ppm in pulse-acquire spectrum), as a series of $^{n}Bu_3N(HF)_x$ complexes could form in solutions of $^{n}Bu_3N$ and HF. Thus, we tentatively assigned these three unknown spins as such adducts of different stoichiometries.

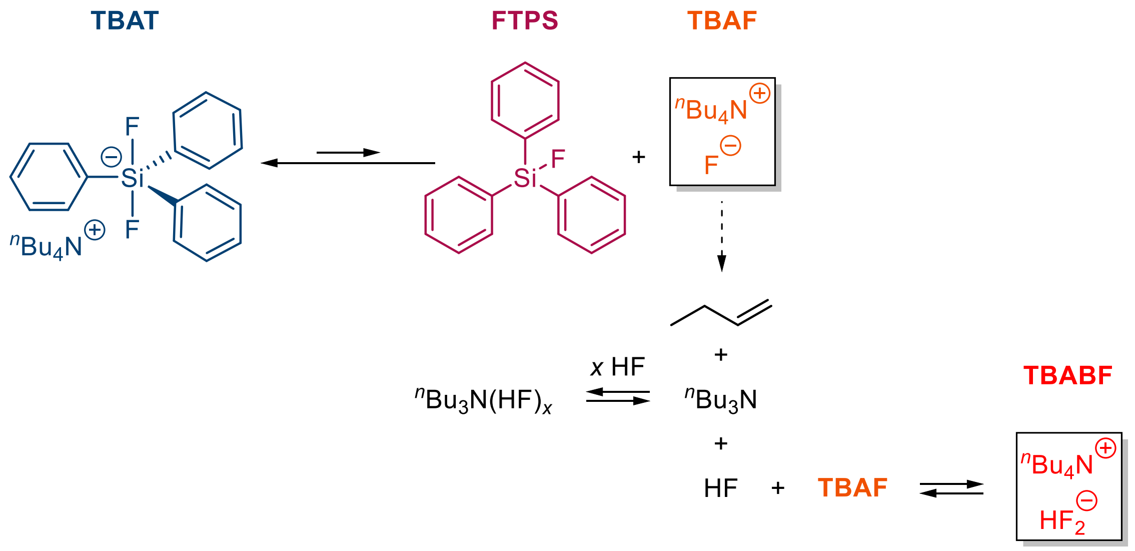

Scheme S4.2: A proposed scheme for the dissociation of TBAT in anhydrous MeCN, and the subsequent Hofmann elimination from the anhydrous TBAF (dashed arrow).[S11]

S5. Magnetisation transfer NMR spectroscopy

S5.1. General information

The pulse sequence used in ^{19}F selective inversion-recovery experiments is presented in Figure S5.1 (t1ir, modified by replacing the non-selective π pulse by a selective shaped π pulse). After a relaxation delay, D_1, a soft π pulse (iSnob, duration: 1.295 ms, bandwidth: 1.200 kHz) was used to selectively invert a range of frequencies, followed by variable delay, τ. The resulting longitudinal magnetisations were detected using a hard π/2 pulse and acquisition (with t_{acq} = 1.5 s acquisition time). This pulse sequence was repeated for a series of incremented variable delays, τ, which were optimised based on the longitudinal relaxation time

constants of the spins and the rates of exchange (17 or 25 for TBAT/FTPS exchange and 17 for TBAT/ARSF exchange). The first spectrum in each selective inversion-recovery experiment (τ = 0) was discarded from data analysis due to phase distortion. $D_1 \geq 5T_1 - t_{acq}$ was used to achieve at least 99.3% longitudinal magnetisation recovery, where T_1 was the longest longitudinal relaxation time constant among spins of interest in the system. The experiments were performed with 0 dummy scans and 1 scan. The transmitter resonance was placed on one of the two exchanging signals (TBAT for TBAT/FTPS system and ARSF for TBAT/ARSF system). The centre of the soft π pulse was placed on the other signal in each two-spin system. The variable delay lists used in these experiments are given where appropriate.

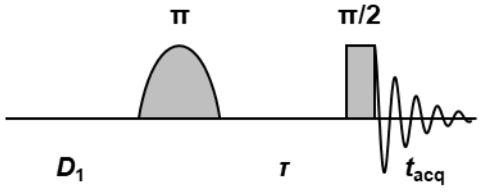

Figure S5.1: The pulse sequence used for magnetisation transfer experiments in this study.

To the experimental integrals within each dataset was fitted a model dependent upon the system, as derived below.

S5.2. TBAT/FTPS

S5.2.1. Kinetic model

The two proposed pathways of fluoride transfer from TBAT are presented in Scheme S5.1. In the dissociative fluoride transfer pathway (process 1, Scheme S5.1a), TBAT first dissociates reversibly into FTPS and TBAF. TBAF is then the active fluorinating agent in the (irreversible or reversible) reaction with the electrophile (E^+). In the direct fluoride transfer pathway (process 2, Scheme S5.1b), TBAT reacts directly with E^+ in a bimolecular elementary reaction (irreversibly or reversibly).

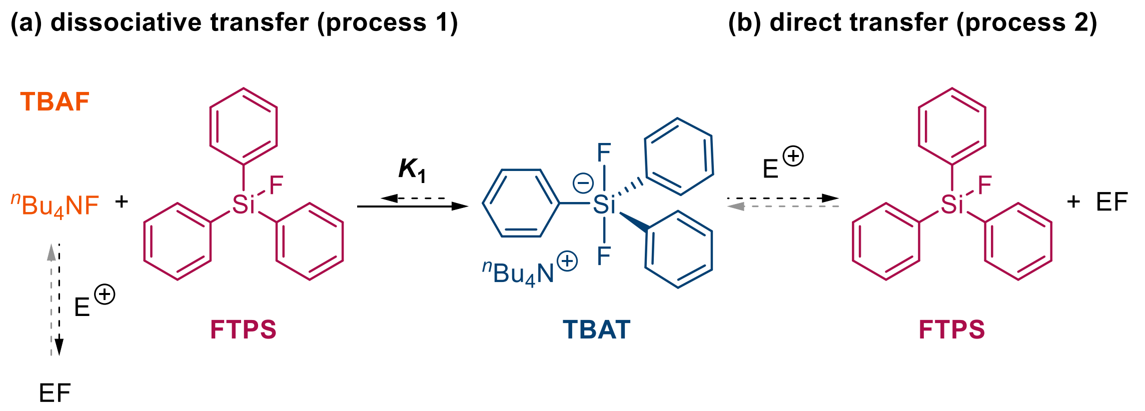

Scheme S5.1: Two proposed pathways of fluoride transfer from TBAT. (a) Dissociative transfer *via* TBAF (process 1) and (b) direct bimolecular transfer (process 2).

Thermodynamics of TBAT dissociation into FTPS and TBAF

As addressed in ^{19}F CEST experiments, TBAT in solution dissociates into FTPS and TBAF with the equilibrium constant $K_1 << 1$. Consider TBAT and FTPS dissolved in the solvent in such amounts that their initial concentrations (before the system "starts" equilibrating) are $[TBAT]_0$ and $[FTPS]_0$, respectively ($[TBAF]_0 = 0$). When the system reaches equilibrium, the equilibrium concentrations of the three species are: $[TBAT] = [TBAT]_0 - x$, $[FTPS] = [FTPS]_0 + x$, $[TBAF] = x$, where x is the concentration of TBAT which dissociates into FTPS and TBAF. Since $K_1 << 1$ and assuming $x << [FTPS]_0$, the equilibrium concentrations of TBAT and FTPS can be approximated to their initial concentrations, $[TBAT] \approx [TBAT]_0$ and $[FTPS] \approx [FTPS]_0$. If no further equilibria are present in the system, the equilibrium concentration of TBAF can be approximated to $[TBAF] \approx K_1[TBAT]_0/[FTPS]_0$.

FTPS as an electrophile in TBAT/FTPS exchange

If TBAT is mixed with FTPS, the latter being the electrophile, both the direct and dissociative pathways can in principle be interrogated *via* ^{19}F magnetisation transfer between the two spins. The overall rate constants of the two processes are, as shown in Scheme S5.2, k_2 for the direct fluoride transfer (in either direction, as the net equilibrium reaction is "0", *i.e.* degenerate, with $\Delta G° = 0$), k_1 for dissociation of TBAT into FTPS and TBAF and k_{-1} for the recombination of FTPS and TBAF into TBAT ($K_1 = k_1/k_{-1} << 1$). Since there are two chemically equivalent fluorine atoms in the difluorotriphenylsilicate anion, the corresponding rate constants per fluorine atom in TBAT are $k_1/2$ and $k_2/2$.

(a) dissociative transfer (process 1)

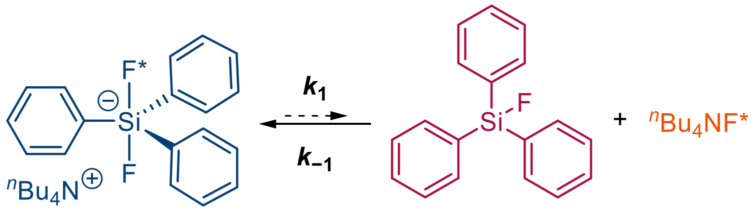

TBAT FTPS TBAF

(b) direct transfer (process 2)

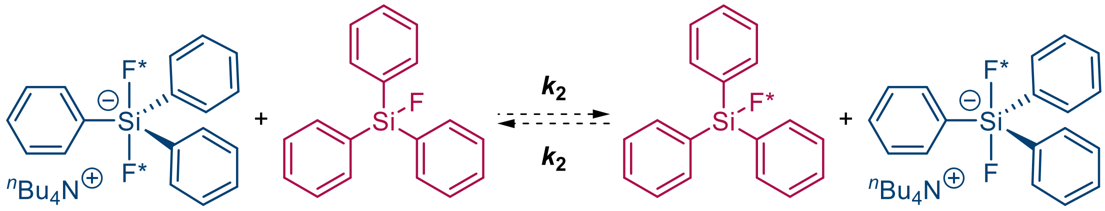

TBAT FTPS FTPS TBAT

Scheme S5.2: Two proposed pathways of fluoride transfer from TBAT to FTPS. (a) Dissociative transfer *via* TBAF, where $K_1 = k_1/k_{-1} << 1$. (b) Direct bimolecular transfer (the rate constants in each direction, k_2, are identical, as $\Delta G° = 0$). In each transfer pathway, exchanging fluorine atoms are marked with an asterisk for clarity.

<u>Magnetisation states of TBAT, FTPS and TBAF</u>

The kinetic models for the magnetisation transfer between the spins in this study are derived based on the discrete inverted and non-inverted spin formalism.[S12] A spin-half nucleus (here, ^{19}F) can exist in two chemically equivalent magnetisation states, herein referred to as magnetised (*i.e.* at magnetic equilibrium along the +*z* axis, $^{19}F^{\blacktriangle}$) and inverted (to the –*z* axis, $^{19}F^{\blacktriangledown}$). Hence, a species S with *n* equivalent ^{19}F nuclei can exist in (n + 1) chemically equivalent magnetic states. Each of the magnetised spins in S contributes $+m_z$, and each of the inverted ones $-m_z$ to the overall longitudinal magnetisation of S, M_z^S (Equations S5.1 and S5.2, where m_z is the longitudinal magnetisation of a single ^{19}F spin at magnetic equilibrium, *i.e.* $^{19}F^{\blacktriangle}$; $M_{z,eq}^S$ is the longitudinal magnetisation of S at magnetic equilibrium; and $N_{\blacktriangle}^S$ and $N_{\blacktriangledown}^S$ are the number of inverted and magnetised spins in S, respectively). For example, an equimolar mixture of FTPS$^{\blacktriangledown}$ and FTPS$^{\blacktriangle}$ shows a null in a ^{19}F NMR spectrum.

Here is introduced the fractional magnetisation of S, m^S, which is the molar fraction of magnetised ^{19}F spins in S; and its relationship to M_z^S and $M_{z,eq}^S$ (Equation S5.3). The equilibrium fractional magnetisation, m_{eq}^S is 1 (and the fractional magnetisation when all spins are inverted is 0).

S5.1

$$M_z^S = \left(N_{\blacktriangle}^S - N_{\blacktriangledown}^S\right)m_z$$

S5.2

$$M_{z,\mathrm{eq}}^{\mathrm{S}} = \left(N_{\blacktriangle}^{\mathrm{S}} + N_{\blacktriangledown}^{\mathrm{S}}\right) m_z$$

S5.3

$$m^{\mathrm{S}} = \frac{N_{\blacktriangle}^{\mathrm{S}}}{N_{\blacktriangle}^{\mathrm{S}} + N_{\blacktriangledown}^{\mathrm{S}}} = \frac{M_z^{\mathrm{S}} + M_{z,\mathrm{eq}}^{\mathrm{S}}}{2M_{z,\mathrm{eq}}^{\mathrm{S}}}$$

FTPS and TBAF therefore can exist in two magnetisation states each and their fractional magnetisations are given by Equations S5.4 and S5.5. TBAT, which possesses two equivalent ^{19}F nuclei, can exist in three magnetisation states, and its fractional magnetisation is given by Equation S5.6.

S5.4

$$m^{\mathrm{FTPS}} = \frac{[\mathrm{FTPS}^{\blacktriangle}]}{[\mathrm{FTPS}]} = \frac{[\mathrm{FTPS}^{\blacktriangle}]}{[\mathrm{FTPS}^{\blacktriangledown}] + [\mathrm{FTPS}^{\blacktriangle}]} = \frac{M_z^{\mathrm{FTPS}} + M_{z,\mathrm{eq}}^{\mathrm{FTPS}}}{2M_{z,\mathrm{eq}}^{\mathrm{FTPS}}}$$

S5.5

$$m^{\mathrm{TBAF}} = \frac{[\mathrm{TBAF}^{\blacktriangle}]}{[\mathrm{TBAF}]} = \frac{[\mathrm{TBAF}^{\blacktriangle}]}{[\mathrm{TBAF}^{\blacktriangledown}] + [\mathrm{TBAF}^{\blacktriangle}]} = \frac{M_z^{\mathrm{TBAF}} + M_{z,\mathrm{eq}}^{\mathrm{TBAF}}}{2M_{z,\mathrm{eq}}^{\mathrm{TBAF}}}$$

S5.6

$$m^{\mathrm{TBAT}} = \frac{[\mathrm{TBAT}^{\blacktriangledown\blacktriangle}] + 2[\mathrm{TBAT}^{\blacktriangle\blacktriangle}]}{2[\mathrm{TBAT}]} = \frac{[\mathrm{TBAT}^{\blacktriangledown\blacktriangle}] + 2[\mathrm{TBAT}^{\blacktriangle\blacktriangle}]}{2([\mathrm{TBAT}^{\blacktriangledown\blacktriangledown}] + [\mathrm{TBAT}^{\blacktriangledown\blacktriangle}] + [\mathrm{TBAT}^{\blacktriangle\blacktriangle}])} = \frac{M_z^{\mathrm{TBAT}} + M_{z,\mathrm{eq}}^{\mathrm{TBAT}}}{2M_{z,\mathrm{eq}}^{\mathrm{TBAT}}}$$

<u>Longitudinal relaxation of TBAT, FTPS and TBAF</u>

Longitudinal relaxation schemes of TBAT, FTPS and TBAF are presented in Scheme S5.3. Since FTPS and TBAF possess one ^{19}F nucleus each and therefore exist in two magnetisation states, their longitudinal relaxation follows simple first order kinetics with the rate constant $k_{\mathrm{S}} = 1/T_1^{\mathrm{S}}$ (T_1^{S} is the longitudinal relaxation time constant of S). However, the existence of two chemically and magnetically equivalent ^{19}F nuclei in TBAT introduces a complication in determining the relative rate constants of the TBAT$^{\blacktriangledown\blacktriangledown}$ → TBAT$^{\blacktriangledown\blacktriangle}$ and TBAT$^{\blacktriangledown\blacktriangle}$ → TBAT$^{\blacktriangle\blacktriangle}$ events, and their relationship to the net relaxation time constant, T_1^{TBAT}. These two processes were found to be described by the rate constants $2/T_1^{\mathrm{TBAT}}$ and $1/T_1^{\mathrm{TBAT}}$, respectively, as derived in Section S7.2.1.

$$\mathrm{TBAT}^{\blacktriangledown\blacktriangledown} \xrightarrow{2/T_1^{\mathrm{TBAT}}} \mathrm{TBAT}^{\blacktriangledown\blacktriangle} \xrightarrow{1/T_1^{\mathrm{TBAT}}} \mathrm{TBAT}^{\blacktriangle\blacktriangle}$$

$$\mathrm{FTPS}^{\blacktriangledown} \xrightarrow{1/T_1^{\mathrm{FTPS}}} \mathrm{FTPS}^{\blacktriangle}$$

$$\mathrm{TBAF}^{\blacktriangledown} \xrightarrow{1/T_1^{\mathrm{TBAF}}} \mathrm{TBAF}^{\blacktriangle}$$

Scheme S5.3: Longitudinal relaxation schemes of TBAT, FTPS and TBAF.

Magnetisation transfer rate laws

The dissociative and direct transfer pathways (process 1 and process 2, respectively), accounting for all (chemically degenerate) magnetisation states of TBAT, FTPS and TBAF are presented in Scheme S5.4. Based on the scheme, rate laws can be derived that describe the temporal changes ($d/d\tau$, where τ is the variable delay) in fractional magnetisations of TBAT, FTPS and TBAF in systems of these three spins.

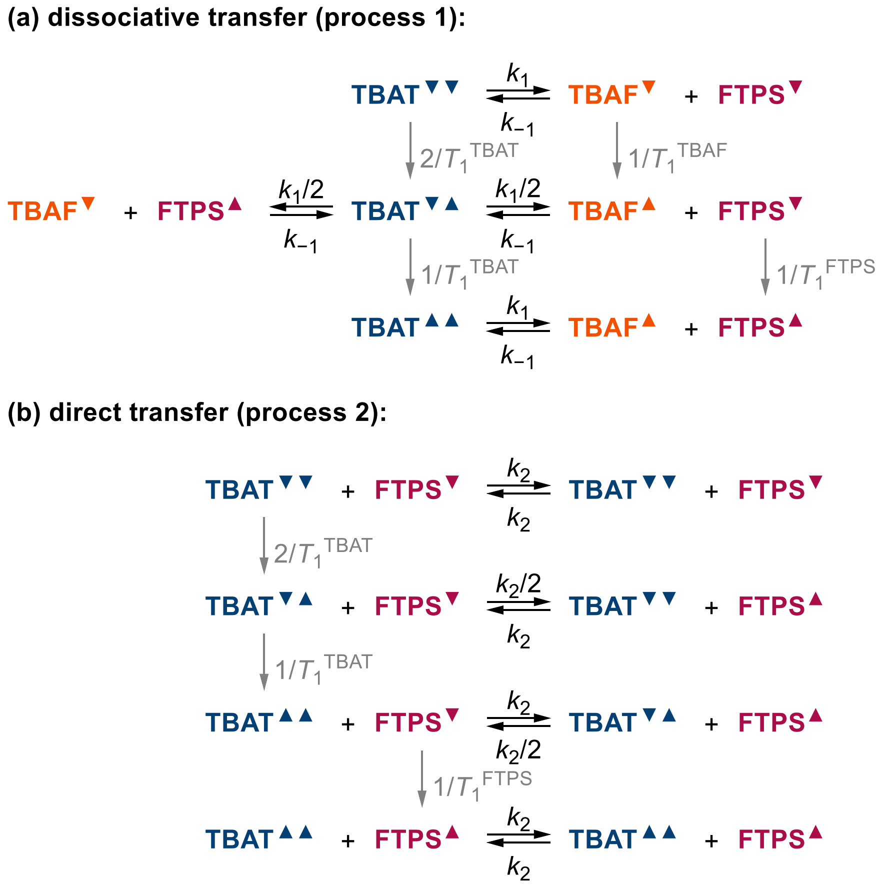

Scheme S5.4: The breakdown of the (a) dissociative and (b) direct transfer exchange pathways between TBAT, FTPS and TBAF, accounting for their longitudinal magnetisation states. Note the rate constants of the chemically degenerate equilibrium transfer processes relative to the overall rate constants, k_1 and k_2, depending on the magnetisation states of the spins.

Longitudinal (T_1) relaxation:

S5.7

$$\left(\frac{d[\mathrm{TBAT}^{\blacktriangledown\blacktriangle}]}{d\tau}\right)_{T_1} = \frac{2}{T_1^{\mathrm{TBAT}}}[\mathrm{TBAT}^{\blacktriangledown\blacktriangledown}] - \frac{1}{T_1^{\mathrm{TBAT}}}[\mathrm{TBAT}^{\blacktriangledown\blacktriangle}]$$

S5.8

$$\left(\frac{d[\mathrm{TBAT}^{\blacktriangle\blacktriangle}]}{d\tau}\right)_{T_1} = \frac{1}{T_1^{\mathrm{TBAT}}}[\mathrm{TBAT}^{\blacktriangledown\blacktriangle}]$$

S5.9

$$\left(\frac{d[\mathrm{FTPS}^{\blacktriangle}]}{d\tau}\right)_{T_1} = \frac{1}{T_1^{\mathrm{FTPS}}}[\mathrm{FTPS}^{\blacktriangledown}]$$

S5.10

$$\left(\frac{d[\mathrm{TBAF}^{\blacktriangle}]}{d\tau}\right)_{T_1} = \frac{1}{T_1^{\mathrm{TBAF}}}[\mathrm{TBAF}^{\blacktriangledown}]$$

Dissociative transfer (process 1, “P1”):

S5.11

$$\left(\frac{d[\mathrm{TBAT}^{\blacktriangledown\blacktriangle}]}{d\tau}\right)_{\mathrm{P1}} = k_{-1}[\mathrm{FTPS}^{\blacktriangledown}][\mathrm{TBAF}^{\blacktriangle}] + k_{-1}[\mathrm{FTPS}^{\blacktriangle}][\mathrm{TBAF}^{\blacktriangledown}] - k_1[\mathrm{TBAT}^{\blacktriangledown\blacktriangle}]$$

S5.12

$$\left(\frac{d[\mathrm{TBAT}^{\blacktriangle\blacktriangle}]}{d\tau}\right)_{\mathrm{P1}} = k_{-1}[\mathrm{FTPS}^{\blacktriangle}][\mathrm{TBAF}^{\blacktriangle}] - k_1[\mathrm{TBAT}^{\blacktriangle\blacktriangle}]$$

S5.13

$$\left(\frac{d[\mathrm{FTPS}^{\blacktriangle}]}{d\tau}\right)_{\mathrm{P1}} = \frac{k_1}{2}[\mathrm{TBAT}^{\blacktriangledown\blacktriangle}] + k_1[\mathrm{TBAT}^{\blacktriangle\blacktriangle}] - k_{-1}[\mathrm{FTPS}^{\blacktriangle}][\mathrm{TBAF}^{\blacktriangledown}] - k_{-1}[\mathrm{FTPS}^{\blacktriangle}][\mathrm{TBAF}^{\blacktriangle}]$$

S5.14

$$\left(\frac{d[\mathrm{TBAF}^{\blacktriangle}]}{d\tau}\right)_{\mathrm{P1}} = \frac{k_1}{2}[\mathrm{TBAT}^{\blacktriangledown\blacktriangle}] + k_1[\mathrm{TBAT}^{\blacktriangle\blacktriangle}] - k_{-1}[\mathrm{TBAF}^{\blacktriangle}][\mathrm{FTPS}^{\blacktriangledown}] - k_{-1}[\mathrm{TBAF}^{\blacktriangle}][\mathrm{FTPS}^{\blacktriangle}]$$

Direct transfer (process 2, “P2”):

S5.15

$$\left(\frac{d[\mathrm{TBAT}^{\blacktriangledown\blacktriangle}]}{d\tau}\right)_{\mathrm{P2}} = k_2[\mathrm{TBAT}^{\blacktriangledown\blacktriangledown}][\mathrm{FTPS}^{\blacktriangle}] + k_2[\mathrm{TBAT}^{\blacktriangle\blacktriangle}][\mathrm{FTPS}^{\blacktriangledown}] - \frac{k_2}{2}[\mathrm{TBAT}^{\blacktriangledown\blacktriangle}][\mathrm{FTPS}^{\blacktriangledown}] - \frac{k_2}{2}[\mathrm{TBAT}^{\blacktriangledown\blacktriangle}][\mathrm{FTPS}^{\blacktriangle}]$$

S5.16

$$\left(\frac{d[\mathrm{TBAT}^{\blacktriangle\blacktriangle}]}{d\tau}\right)_{\mathrm{P2}} = \frac{k_2}{2}[\mathrm{TBAT}^{\blacktriangledown\blacktriangle}][\mathrm{FTPS}^{\blacktriangle}] - k_2[\mathrm{TBAT}^{\blacktriangle\blacktriangle}][\mathrm{FTPS}^{\blacktriangledown}]$$

S5.17

$$\left(\frac{d[\mathrm{FTPS}^{\blacktriangle}]}{d\tau}\right)_{\mathrm{P2}} = \frac{k_2}{2}[\mathrm{TBAT}^{\blacktriangledown\blacktriangle}][\mathrm{FTPS}^{\blacktriangledown}] + k_2[\mathrm{TBAT}^{\blacktriangle\blacktriangle}][\mathrm{FTPS}^{\blacktriangledown}] - k_2[\mathrm{TBAT}^{\blacktriangledown\blacktriangledown}][\mathrm{FTPS}^{\blacktriangle}] - \frac{k_2}{2}[\mathrm{TBAT}^{\blacktriangledown\blacktriangle}][\mathrm{FTPS}^{\blacktriangle}]$$

The following is noted (Equations S5.4–S5.6, recalled):

S5.4, recalled

$$m^{\mathrm{FTPS}} = \frac{[\mathrm{FTPS}^{\blacktriangle}]}{[\mathrm{FTPS}]} = \frac{[\mathrm{FTPS}^{\blacktriangle}]}{[\mathrm{FTPS}^{\blacktriangledown}] + [\mathrm{FTPS}^{\blacktriangle}]}$$

S5.5, recalled

$$m^{\mathrm{TBAF}} = \frac{[\mathrm{TBAF}^{\blacktriangle}]}{[\mathrm{TBAF}]} = \frac{[\mathrm{TBAF}^{\blacktriangle}]}{[\mathrm{TBAF}^{\blacktriangledown}] + [\mathrm{TBAF}^{\blacktriangle}]}$$

S5.6, recalled

$$m^{\mathrm{TBAT}} = \frac{[\mathrm{TBAT}^{\blacktriangledown\blacktriangle}] + 2[\mathrm{TBAT}^{\blacktriangle\blacktriangle}]}{2[\mathrm{TBAT}]} = \frac{[\mathrm{TBAT}^{\blacktriangledown\blacktriangle}] + 2[\mathrm{TBAT}^{\blacktriangle\blacktriangle}]}{2([\mathrm{TBAT}^{\blacktriangledown\blacktriangledown}] + [\mathrm{TBAT}^{\blacktriangledown\blacktriangle}] + [\mathrm{TBAT}^{\blacktriangle\blacktriangle}])}$$

Hence:

S5.18

$$\left(\frac{dm^{\mathrm{TBAT}}}{d\tau}\right)_{T_1} = \frac{1}{T_1^{\mathrm{TBAT}}}(1 - m^{\mathrm{TBAT}})$$

S5.19

$$\left(\frac{dm^{\mathrm{FTPS}}}{d\tau}\right)_{T_1} = \frac{1}{T_1^{\mathrm{FTPS}}}(1 - m^{\mathrm{FTPS}})$$

S5.20

$$\left(\frac{dm^{\mathrm{TBAF}}}{d\tau}\right)_{T_1} = \frac{1}{T_1^{\mathrm{TBAF}}}(1 - m^{\mathrm{TBAF}})$$

S5.21

$$\left(\frac{dm^{\mathrm{TBAT}}}{d\tau}\right)_{\mathrm{P1}} = \frac{k_{-1}[\mathrm{FTPS}][\mathrm{TBAF}]}{2[\mathrm{TBAT}]}(m^{\mathrm{FTPS}} + m^{\mathrm{TBAF}}) - k_1 m^{\mathrm{TBAT}}$$

S5.22

$$\left(\frac{dm^{\mathrm{FTPS}}}{d\tau}\right)_{\mathrm{P1}} = \frac{k_1[\mathrm{TBAT}]}{[\mathrm{FTPS}]}m^{\mathrm{TBAT}} - k_{-1}[\mathrm{TBAF}]m^{\mathrm{FTPS}}$$

S5.23

$$\left(\frac{dm^{\mathrm{TBAF}}}{d\tau}\right)_{\mathrm{P1}} = \frac{k_1[\mathrm{TBAT}]}{[\mathrm{TBAF}]}m^{\mathrm{TBAT}} - k_{-1}[\mathrm{FTPS}]m^{\mathrm{TBAF}}$$

S5.24

$$\left(\frac{dm^{\mathrm{TBAT}}}{d\tau}\right)_{\mathrm{P2}} = \frac{k_2}{2}[\mathrm{FTPS}](m^{\mathrm{FTPS}} - m^{\mathrm{TBAT}})$$

S5.25

$$\left(\frac{dm^{\mathrm{FTPS}}}{d\tau}\right)_{\mathrm{P2}} = k_2[\mathrm{TBAT}](m^{\mathrm{TBAT}} - m^{\mathrm{FTPS}})$$

The total temporal change in the fractional magnetisation of a species, $dm^{\mathrm{S}}/d\tau$ is the sum of contributions from relaxation, dissociative (process 1) and direct (process 2) transfer pathways. Hence, summing Equations S5.18, S5.21 and S5.24; S5.19, S5.22 and S5.25; and S5.20 and S5.23, gives the fractional magnetisation rate laws of TBAT, FTPS and TBAF (Equations S5.26, S5.27 and S5.28), respectively.

S5.26

$$\frac{dm^{\mathrm{TBAT}}}{d\tau}=\left(\frac{dm^{\mathrm{TBAT}}}{d\tau}\right)_{T_1}+\left(\frac{dm^{\mathrm{TBAT}}}{d\tau}\right)_{\mathrm{P1}}+\left(\frac{dm^{\mathrm{TBAT}}}{d\tau}\right)_{\mathrm{P2}}$$
$$=\frac{1}{T_1^{\mathrm{TBAT}}}(1-m^{\mathrm{TBAT}})+\frac{k_{-1}[\mathrm{FTPS}][\mathrm{TBAF}]}{2[\mathrm{TBAT}]}(m^{\mathrm{FTPS}}+m^{\mathrm{TBAF}})-k_1m^{\mathrm{TBAT}}$$
$$+\frac{k_2}{2}[\mathrm{FTPS}](m^{\mathrm{FTPS}}-m^{\mathrm{TBAT}})$$

S5.27

$$\frac{dm^{\mathrm{FTPS}}}{d\tau}=\left(\frac{dm^{\mathrm{FTPS}}}{d\tau}\right)_{T_1}+\left(\frac{dm^{\mathrm{FTPS}}}{d\tau}\right)_{\mathrm{P1}}+\left(\frac{dm^{\mathrm{FTPS}}}{d\tau}\right)_{\mathrm{P2}}$$
$$=\frac{1}{T_1^{\mathrm{FTPS}}}(1-m^{\mathrm{FTPS}})+\frac{k_1[\mathrm{TBAT}]}{[\mathrm{FTPS}]}m^{\mathrm{TBAT}}-k_{-1}[\mathrm{TBAF}]m^{\mathrm{FTPS}}$$
$$+k_2[\mathrm{TBAT}](m^{\mathrm{TBAT}}-m^{\mathrm{FTPS}})$$

S5.28

$$\frac{dm^{\mathrm{TBAF}}}{d\tau}=\left(\frac{dm^{\mathrm{TBAF}}}{d\tau}\right)_{T_1}+\left(\frac{dm^{\mathrm{TBAF}}}{d\tau}\right)_{\mathrm{P1}}=\frac{1}{T_1^{\mathrm{TBAF}}}(1-m^{\mathrm{TBAF}})+\frac{k_1[\mathrm{TBAT}]}{[\mathrm{TBAF}]}m^{\mathrm{TBAT}}-k_{-1}[\mathrm{FTPS}]m^{\mathrm{TBAF}}$$

When TBAT is mixed with FTPS, three spins exist in equilibrium – TBAT, FTPS and TBAF. In order to simplify the mathematical analysis, we apply the steady state approximation of the fractional magnetisation of TBAF as a low-concentration intermediate in the magnetisation transfer between TBAT and FTPS (Equation S5.29).

S5.29

$$\frac{dm^{\mathrm{TBAF}}}{d\tau}=\frac{1}{T_1^{\mathrm{TBAF}}}(1-m^{\mathrm{TBAF}})+\frac{k_1[\mathrm{TBAT}]}{[\mathrm{TBAF}]}m^{\mathrm{TBAT}}-k_{-1}[\mathrm{FTPS}]m^{\mathrm{TBAF}}\approx 0$$

Solving Equation S5.29 for m^{TBAF} (ignoring the longitudinal relaxation of TBAF):

S5.30

$$m^{\mathrm{TBAF}}\approx\frac{\frac{1}{T_1^{\mathrm{TBAF}}}+\frac{k_1[\mathrm{TBAT}]}{[\mathrm{TBAF}]}m^{\mathrm{TBAT}}}{\frac{1}{T_1^{\mathrm{TBAF}}}+k_{-1}[\mathrm{FTPS}]}\approx\frac{k_1[\mathrm{TBAT}]}{k_{-1}[\mathrm{FTPS}][\mathrm{TBAF}]}m^{\mathrm{TBAT}}$$

Reintroducing the equilibrium constant of the dissociation of TBAT into FTPS and TBAF (K_1, Equation S5.31), Equation S5.30 is simplified to Equation S5.32, where the fractional magnetisation of TBAF can be approximated to that of TBAT.

S5.31

$$K_1=\frac{k_1}{k_{-1}}=\frac{[\mathrm{FTPS}][\mathrm{TBAF}]}{[\mathrm{TBAT}]}$$

S5.32

$$m^{\mathrm{TBAF}}\approx m^{\mathrm{TBAT}}$$

Inserting Equations S5.31 and S5.32 into Equations S5.26 and S5.27 gives Equations S5.33 and S5.34, which describe the TBAT/FTPS system, where TBAT and FTPS exchange fluoride *via* a low-concentration TBAF intermediate (process 1), directly in a bimolecular reaction (process 2), or *via* both these mechanisms proceeding in parallel.

S5.33

$$\frac{dm^{\mathrm{TBAT}}}{d\tau} = \frac{1}{T_1^{\mathrm{TBAT}}}(1 - m^{\mathrm{TBAT}}) + \frac{k_1 + k_2[\mathrm{FTPS}]}{2}(m^{\mathrm{FTPS}} - m^{\mathrm{TBAT}})$$

S5.34

$$\frac{dm^{\mathrm{FTPS}}}{d\tau} = \frac{1}{T_1^{\mathrm{FTPS}}}(1 - m^{\mathrm{FTPS}}) + \left(\frac{k_1[\mathrm{TBAT}]}{[\mathrm{FTPS}]} + k_2[\mathrm{TBAT}]\right)(m^{\mathrm{TBAT}} - m^{\mathrm{FTPS}})$$

Introducing herein an auxiliary term, α, and the ratio of equilibrium longitudinal magnetisations of TBAT-to-FTPS, r (Equations S5.35 and S5.36), rate laws take the form as in Equations S5.37 and S5.38.

S5.35

$$\alpha = \frac{k_1 + k_2[\mathrm{FTPS}]}{2}$$

S5.36

$$r = \frac{M_{z,\mathrm{eq}}^{\mathrm{TBAT}}}{M_{z,\mathrm{eq}}^{\mathrm{FTPS}}} = \frac{2[\mathrm{TBAT}]}{[\mathrm{FTPS}]}$$

S5.37

$$\frac{dm^{\mathrm{TBAT}}}{d\tau} = \frac{1}{T_1^{\mathrm{TBAT}}}(1 - m^{\mathrm{TBAT}}) + \alpha(m^{\mathrm{FTPS}} - m^{\mathrm{TBAT}})$$

S5.38

$$\frac{dm^{\mathrm{FTPS}}}{d\tau} = \frac{1}{T_1^{\mathrm{FTPS}}}(1 - m^{\mathrm{FTPS}}) + r\alpha(m^{\mathrm{TBAT}} - m^{\mathrm{FTPS}})$$

<u>Integration of the rate laws</u>

Equations S5.37 and S5.38 can be solved analytically in the following way. Rearranging Equations S5.37 and S5.38 for m^{FTPS} and m^{TBAT}, respectively, yields Equations S5.39 and S5.40.

S5.39

$$m^{\mathrm{TBAT}} = \frac{\frac{dm^{\mathrm{FTPS}}}{d\tau} + \left(r\alpha + \frac{1}{T_1^{\mathrm{FTPS}}}\right)m^{\mathrm{FTPS}} - \frac{1}{T_1^{\mathrm{FTPS}}}}{r\alpha}$$

S5.40

$$m^{\mathrm{FTPS}} = \frac{\frac{dm^{\mathrm{TBAT}}}{d\tau} + \left(\alpha + \frac{1}{T_1^{\mathrm{TBAT}}}\right)m^{\mathrm{TBAT}} - \frac{1}{T_1^{\mathrm{TBAT}}}}{\alpha}$$

Inserting Equations S5.39 and S5.40 into Equations S5.37 and S5.38, respectively, and rearranging gives Equations S5.41 and S5.42.

S5.41

$$\frac{d^2m^{\mathrm{TBAT}}}{d\tau^2}+\left(\alpha+\frac{1}{T_1^{\mathrm{TBAT}}}+r\alpha+\frac{1}{T_1^{\mathrm{FTPS}}}\right)\frac{dm^{\mathrm{TBAT}}}{d\tau}+\frac{\alpha T_1^{\mathrm{TBAT}}+r\alpha T_1^{\mathrm{FTPS}}+1}{T_1^{\mathrm{TBAT}}T_1^{\mathrm{FTPS}}}(m^{\mathrm{TBAT}}-1)=0$$

S5.42

$$\frac{d^2m^{\mathrm{FTPS}}}{d\tau^2}+\left(\alpha+\frac{1}{T_1^{\mathrm{TBAT}}}+r\alpha+\frac{1}{T_1^{\mathrm{FTPS}}}\right)\frac{dm^{\mathrm{FTPS}}}{d\tau}+\frac{\alpha T_1^{\mathrm{TBAT}}+r\alpha T_1^{\mathrm{FTPS}}+1}{T_1^{\mathrm{TBAT}}T_1^{\mathrm{FTPS}}}(m^{\mathrm{FTPS}}-1)=0$$

Equations S5.41 and S5.42 are second-order, non-homogeneous ordinary differential equations, which can be solved analytically to give m^{TBAT} and m^{FTPS}, as presented in Equations S5.43–S5.46.

S5.43

$$m^{\mathrm{TBAT}}=c_1e^{(\beta+\gamma)\tau}+c_2e^{(\beta-\gamma)\tau}+1$$

S5.44

$$m^{\mathrm{FTPS}}=c_3e^{(\beta+\gamma)\tau}+c_4e^{(\beta-\gamma)\tau}+1$$

Where:

S5.45

$$\beta=-\frac{\alpha(r+1)+\frac{1}{T_1^{\mathrm{TBAT}}}+\frac{1}{T_1^{\mathrm{FTPS}}}}{2}$$

S5.46

$$\gamma=\frac{\sqrt{\left(\alpha+\frac{1}{T_1^{\mathrm{TBAT}}}-r\alpha-\frac{1}{T_1^{\mathrm{FTPS}}}\right)^2+4r\alpha^2}}{2}$$

S5.35, recalled

$$\alpha=\frac{k_1+k_2[\mathrm{FTPS}]}{2}$$

S5.36, recalled

$$r=\frac{M_{z,\mathrm{eq}}^{\mathrm{TBAT}}}{M_{z,\mathrm{eq}}^{\mathrm{FTPS}}}=\frac{2[\mathrm{TBAT}]}{[\mathrm{FTPS}]}$$

The constants c_1–c_4 depend on the inversion scenario and are given in Equations S5.47–S5.50, where m_0^{TBAT} and m_0^{FTPS} are the fractional magnetisations of TBAT and FTPS at $\tau = 0$ (see Section S7.2.2 for the derivation).

S5.47

$$c_1=\frac{\alpha\left(m_0^{\mathrm{FTPS}}-m_0^{\mathrm{TBAT}}\right)+\left(\beta-\gamma+\frac{1}{T_1^{\mathrm{TBAT}}}\right)(1-m_0^{\mathrm{TBAT}})}{2\gamma}$$

S5.48

$$c_2 = -\frac{\alpha\left(m_0^{\mathrm{FTPS}} - m_0^{\mathrm{TBAT}}\right) + \left(\beta + \gamma + \frac{1}{T_1^{\mathrm{TBAT}}}\right)\left(1 - m_0^{\mathrm{TBAT}}\right)}{2\gamma}$$

S5.49

$$c_3 = \frac{r\alpha\left(m_0^{\mathrm{TBAT}} - m_0^{\mathrm{FTPS}}\right) + \left(\beta - \gamma + \frac{1}{T_1^{\mathrm{FTPS}}}\right)\left(1 - m_0^{\mathrm{FTPS}}\right)}{2\gamma}$$

S5.50

$$c_4 = -\frac{r\alpha\left(m_0^{\mathrm{TBAT}} - m_0^{\mathrm{FTPS}}\right) + \left(\beta + \gamma + \frac{1}{T_1^{\mathrm{FTPS}}}\right)\left(1 - m_0^{\mathrm{FTPS}}\right)}{2\gamma}$$

<u>Further approximations</u>

It was found that in both THF and MeCN, the TBAT/FTPS exchange is significantly faster than relaxation. Additional approximations can therefore be applied in order to simplify the model further for the fractional magnetisation (or integral) of the initially non-inverted signal (here, TBAT) as a function of variable delay.

Firstly, the longitudinal relaxation time constants of TBAT and FTPS in their mixture become equal (T_1^{obs}) and are the weighted average of the longitudinal relaxation time constants of the isolated spins under given conditions (Equation S5.51).[S13]

S5.51

$$T_1^{\mathrm{obs}} = \frac{T_1^{\mathrm{TBAT}} T_1^{\mathrm{FTPS}}(r + 1)}{T_1^{\mathrm{TBAT}} + rT_1^{\mathrm{FTPS}}}$$

Substituting T_1^{TBAT} and T_1^{FTPS} with T_1^{obs} in β and γ, approximates them to:

S5.52

$$\beta \approx -\frac{\alpha(r + 1)}{2} - \frac{1}{T_1^{\mathrm{obs}}}$$

S5.53

$$\gamma \approx \frac{\alpha(r + 1)}{2}$$

The two exponentials in Equations S5.43 and S5.44 now represent separated exchange and relaxation processes (Equations S5.54 and S5.55).

S5.54

$$\beta - \gamma \approx -\alpha(r + 1) - \frac{1}{T_1^{\mathrm{obs}}} \approx -\alpha(r + 1) \approx -2\gamma$$

S5.55

$$\beta + \gamma \approx -\frac{1}{T_1^{\text{obs}}}$$

The constants c_1 and c_2 can therefore be approximated to:

S5.56

$$c_1 \approx -\frac{\Delta m_0}{r+1} + m_0^{\text{TBAT}} - 1$$

S5.57

$$c_2 \approx \frac{\Delta m_0}{r+1}$$

Where Δm_0 is the difference between the fractional magnetisations of TBAT and FTPS immediately after the soft pulse (at $\tau = 0$):

S5.58

$$\Delta m_0 = m_0^{\text{TBAT}} - m_0^{\text{FTPS}}$$

For solutions in THF, the exchange between the spins is slow with respect to the duration of the selective inversion pulse (~1.3 ms), and therefore $\Delta m_0 < 1$ solely due to imperfect inversion of FTPS (*i.e.* $m_0^{\text{TBAT}} \approx 1$ and $m_0^{\text{FTPS}} > 0$). Equation S5.43 is therefore simplified to:

S5.59

$$m^{\text{TBAT}} \approx \frac{\Delta m_0}{r+1}\left[e^{-\alpha(r+1)\tau} - e^{-\frac{\tau}{T_1^{\text{obs}}}}\right] + 1 \approx \frac{1 - m_0^{\text{FTPS}}}{r+1}\left[e^{-\alpha(r+1)\tau} - e^{-\frac{\tau}{T_1^{\text{obs}}}}\right] + 1$$

Which can be expressed as $M_z^{\text{TBAT}}(\tau)$ according to Equation S5.3 (x is the pre-exponential term, which contains the initial magnetisations of the two spins and the ratio of their equilibrium magnetisations):

S5.60

$$M_z^{\text{TBAT}} \approx \frac{M_{z,\text{eq}}^{\text{TBAT}} - rM_{z,0}^{\text{FTPS}}}{r+1}\left[e^{-\alpha(r+1)\tau} - e^{-\frac{\tau}{T_1^{\text{obs}}}}\right] + M_{z,\text{eq}}^{\text{TBAT}} = x\left[e^{-\alpha(r+1)\tau} - e^{-\frac{\tau}{T_1^{\text{obs}}}}\right] + M_{z,\text{eq}}^{\text{TBAT}}$$

The exchange between the spins in MeCN is more rapid than in THF, and no longer negligible with respect to the duration of the soft inversion pulse (~1.3 ms). Therefore, $m_0^{\text{TBAT}} < 1$ and $m_0^{\text{FTPS}} > 0$, and Equation S5.43 is simplified to:

S5.61

$$m^{\text{TBAT}} \approx \frac{\Delta m_0}{r+1} e^{-\alpha(r+1)\tau} + \frac{r(m_0^{\text{TBAT}} - 1) + m_0^{\text{FTPS}} - 1}{r+1} e^{-\frac{\tau}{T_1^{\text{obs}}}} + 1$$

Which can be expressed as $M_z^{\text{TBAT}}(\tau)$ according to Equation S5.3 (x and x' are the two unequal pre-exponential terms):

S5.62

$$M_z^{\mathrm{TBAT}} \approx \frac{M_{z,0}^{\mathrm{TBAT}} - rM_{z,0}^{\mathrm{FTPS}}}{r+1} e^{-\alpha(r+1)\tau} + \frac{r\left(M_0^{\mathrm{TBAT}} + M_0^{\mathrm{FTPS}} - M_{\mathrm{eq}}^{\mathrm{TBAT}}\right) - M_{\mathrm{eq}}^{\mathrm{TBAT}}}{r+1} e^{-\frac{\tau}{T_1^{\mathrm{obs}}}} + M_{z,\mathrm{eq}}^{\mathrm{TBAT}}$$

$$= xe^{-\alpha(r+1)\tau} - x'e^{-\frac{\tau}{T_1^{\mathrm{obs}}}} + M_{z,\mathrm{eq}}^{\mathrm{TBAT}}$$

Therefore, in TBAT/FTPS solutions in THF, to the experimental integrals of TBAT at each variable delay was fitted a three-parameter ($M_{z,\mathrm{eq}}^{\mathrm{TBAT}}$, x, α) model of Equation S5.60.[a] The parameters r and T_1^{obs} were determined from ^{19}F pulse-acquire and ^{19}F inversion-recovery experiments. The experimental and model integrals were then converted to fractional magnetisations (Equation S5.3). In TBAT/FTPS solutions in MeCN, to the experimental integrals of TBAT at each variable delay was fitted a four-parameter ($M_{z,\mathrm{eq}}^{\mathrm{TBAT}}$, x, x', α) model of Equation S5.62. The parameters r and T_1^{obs} were determined from ^{19}F pulse acquire and ^{19}F inversion-recovery experiments. The experimental and model integrals were then converted into fractional magnetisations (Equation S5.3).

S5.2.2. Magnitude of exchange rate between TBAT and FTPS in THF

A solution was prepared in the glovebox by weighing TBAT (52.8 mM), FTPS (84.8 mM) and NAPH (217 mM) into a 1 mL volumetric flask and dissolving them in THF up to the 1 mL mark. The solution was transferred to a J Young® valve capped NMR tube. ^{19}F pulse-acquire, ^{19}F inversion-recovery and ^{19}F selective inversion-recovery experiments were run at 300 K. The variable delays used in the inversion-recovery experiment were, τ (s) {0.25, 1.25, 2.5, 3.75, 5, 7.5, 10, 15}. The variable delays used in the selective inversion-recovery experiment were, τ (s) {0.001, 0.002, 0.005, 0.01, 0.02, 0.05, 0.1, 0.2, 0.5, 1, 2, 5, 7.5, 10, 12.5, 15}.

The concentrations of TBAT and FTPS, based on the NAPH internal standard, were [TBAT] = 52.5 mM and [FTPS] = 83.9 mM (99.5% and 98.9% of the values based on masses, respectively). The ^{19}F longitudinal relaxation fitting parameters (I_{eq}, x and T_1) and root mean square errors (R) obtained for TBAT and FTPS are presented in Table S5.1a. The ^{19}F exchange fitting parameters (I_{eq}, x and α) and root mean square error (R) obtained for (the initially non-inverted) TBAT are presented in Table S5.1b. The pre-exponential term x, determined in the fitting, corresponds to m_0^{FTPS} = 0.0669 (*i.e.* 93.3% inversion of FTPS by the selective pulse). The stack of ^{19}F selective inversion-recovery NMR spectra is presented in Figure S5.2 and the plots of experimental and model fractional magnetisations against variable delay in the non-selective and selective inversion-recovery experiments are presented in Figure S5.3.

[a] Where specified in subsequent sections, $\alpha(r + 1)$ was used as a parameter instead of α.

Table S5.1: The fitting parameters and root mean square errors (*R*) obtained from (a) ^{19}F inversion-recovery of TBAT and FTPS and (b) ^{19}F magnetisation transfer between inverted FTPS and non-inverted TBAT in solution.

(a) ^{19}F inversion-recovery				
**Species**	I_{eq}	x	T_1 (s)	R
TBAT	821	1.35	2.43	1
FTPS	622	1.43	2.47	4
**(b) ^{19}F selective inversion-recovery**				
**Species**	I_{eq}	x	α (s^{-1})	R
TBAT	1782	1476	12.1	8

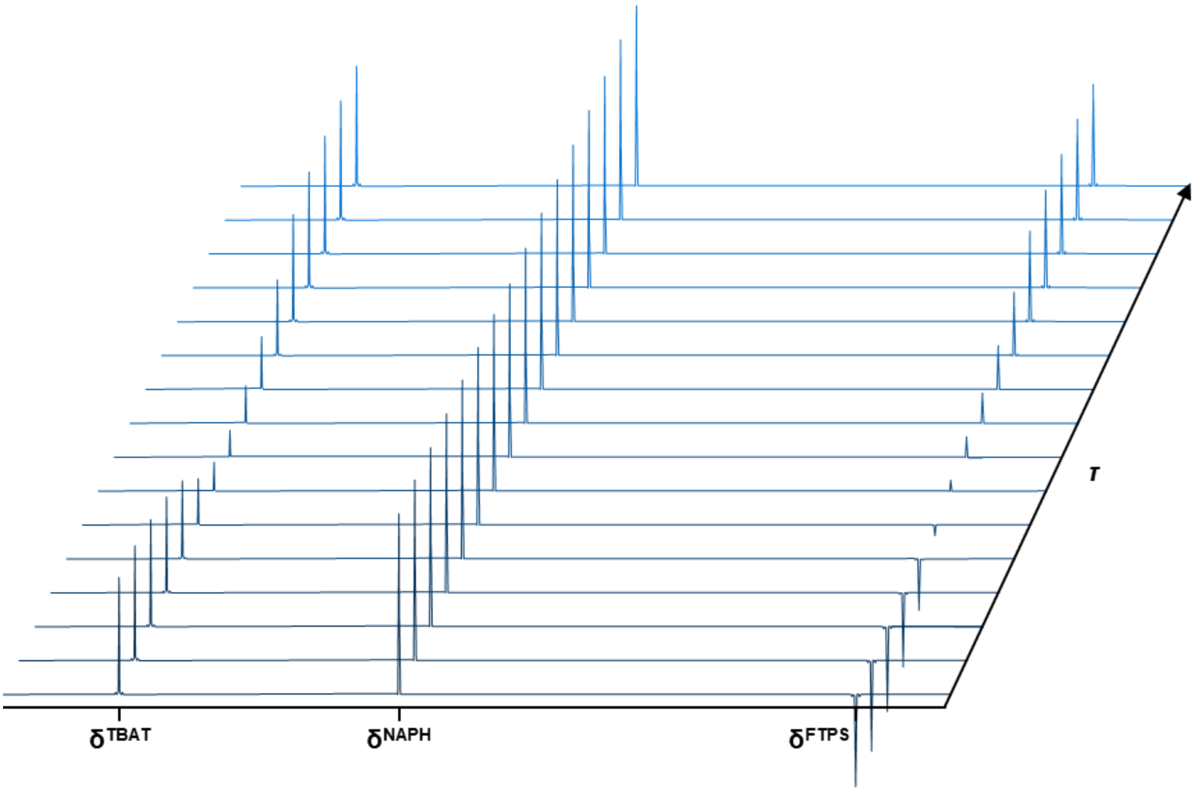

Figure S5.2: ^{19}F magnetisation transfer in a solution of TBAT (52.5 mM) and FTPS (83.9 mM) in THF at 300 K. Selective inversion of FTPS is transferred to TBAT *via* chemical exchange. The extent of the magnetisation transfer and longitudinal relaxation of the spins depends on the variable delay, *τ*. The magnetisation of NAPH (internal standard) is unaffected in the experiment.

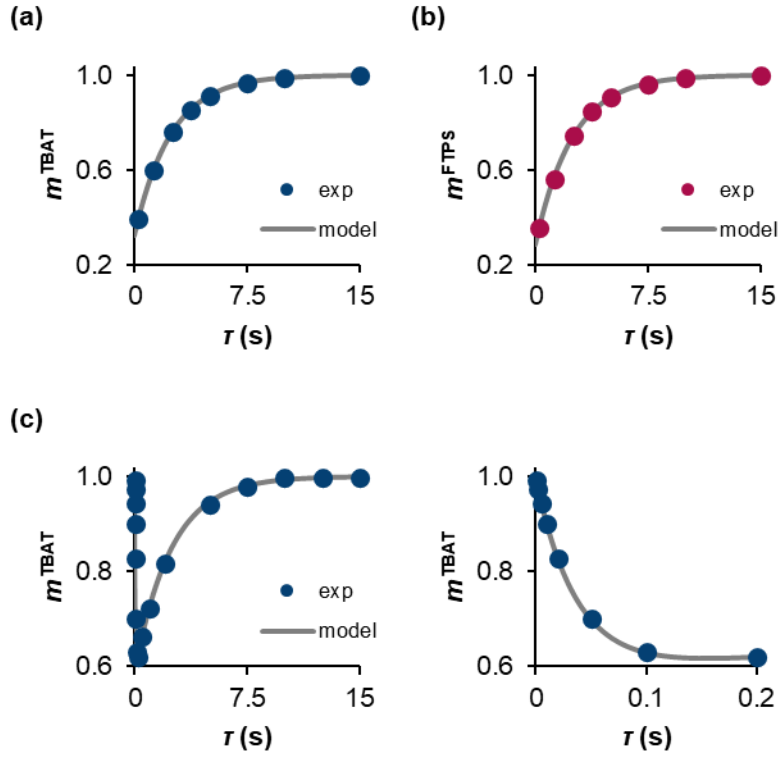

Figure S5.3: Plots of experimental ("exp") and model fractional magnetisations of (a) TBAT and (b) FTPS in ^{19}F longitudinal relaxation constant measurement of the solution containing both spins. (c) ^{19}F magnetisation transfer profile of the initially non-inverted TBAT (left) and an expansion of the plot to early variable delays (right).

Both ^{19}F spins, that of TBAT and of FTPS relaxed with equal rates in a non-selective inversion-recovery experiment. The observed longitudinal relaxation time constant, T_1^{obs} = 2.45 s (an average of 2.43 s and 2.47 s obtained for TBAT and FTPS, respectively), was nearly identical to the weighted average of the values expected for the separated spins under these conditions (2.54 s weighted average from T_1^{TBAT} = 1.56 s and T_1^{FTPS} = 12.0 s, Equation S5.51). This observation was indicative of a very rapid exchange between the ^{19}F spins on the longitudinal relaxation timescale, as confirmed by the magnetisation transfer experiment, αT_1^{obs} = 29.7.

S5.2.3. Initial titrations of TBAT with FTPS in THF

Four solutions were prepared in the glovebox by weighing TBAT, FTPS and NAPH into 1 mL volumetric flasks and dissolving them in THF up to the 1 mL mark. The solutions were transferred to J Young® valve capped NMR tubes. The resulting concentrations in the solutions are presented in Table S5.2. ^{19}F pulse-acquire and ^{19}F selective inversion-recovery experiments were run at 300 K. Each solution was then titrated with five solid aliquots of FTPS, inside the glovebox. After each solid addition, the NMR tube was shaken thoroughly, and the ^{19}F pulse-acquire (for quantitation of FTPS only), as well as ^{19}F selective inversion-recovery experiments were repeated. A series of 24 variable delays were used in the selective inversion-recovery experiments: the first 16 values, τ (s) {0.001, 0.002, 0.003, 0.005, 0.007,

0.01, 0.015, 0.02, 0.025, 0.05, 0.1, 0.15, 0.2, 0.25, 0.5, 1}, were identical for all runs, and the remaining eight – optimised for each solution based on T_1^{obs}.

Table S5.2: Concentrations of TBAT, FTPS and NAPH.

Entry	Solvent	Concentration (mM)		
		TBAT	FTPS	NAPH
1	THF	200	101	215
2	THF	150	106	209
3	THF	100	62.5	197
4	THF	51.8	15.9	296

The concentrations of TBAT (determined for the original solutions and assumed constant throughout the titrations) and FTPS (determined for each solution), based on the NAPH internal standard, are presented in Table S5.3, along with the T_1^{obs} values predicted from T_1^{TBAT} and T_1^{FTPS} as isolated spins for these concentrations. The ^{19}F exchange fitting parameters (I_{eq}, x and α; r and T_1^{obs} were used as in Table S5.3) and root mean square error (R) obtained for (the initially non-inverted) TBAT are presented in Table S5.3. A selection of the magnetisation transfer profiles of TBAT (titration of 202 mM TBAT) is presented in Figure S5.4.

Table S5.3: Quantitation of TBAT and FTPS in the initial titration experiments and the results of ^{19}F inversion-recovery measurements.

[TBAT] (mM)	[FTPS] (mM)	T_1^{obs} (s)	I_{eq}	x	α (s^{-1})	R
202	97.0	1.47	5925	2221	14.6	32
202	113	1.51	5820	2453	18.3	34
202	134	1.56	5776	2765	22.9	32
202	155	1.61	5740	3051	27.7	41
202	180	1.68	5715	3374	34.7	38
202	194	1.71	5714	3549	44.2	51
150	104	1.67	4374	2122	7.33	30
150	120	1.73	4348	2324	15.6	51
150	136	1.79	4365	2512	23.3	76
150	155	1.85	4354	2726	32.3	88
150	174	1.92	4391	2951	42.9	104
150	191	1.98	4432	3199	77.4	128
99.0	60.2	1.76	2974	1336	11.3	15
99.0	74.4	1.84	2928	1518	14.6	23
99.0	94.6	1.96	2910	1796	18.6	25
99.0	109	2.04	2896	1958	22.6	23
99.0	133	2.17	2882	2202	26.5	27
99.0	148	2.25	2852	2294	30.9	28
52.9	17.2	1.77	12467	3382	3.11	48
52.9	36.4	2.01	3016	1466	7.21	17
52.9	54.1	2.21	1485	942	11.7	16
52.9	71.3	2.40	1502	1150	14.8	16
52.9	85.5	2.55	1484	1259	17.8	13
52.9	111	2.81	1488	1426	22.7	12

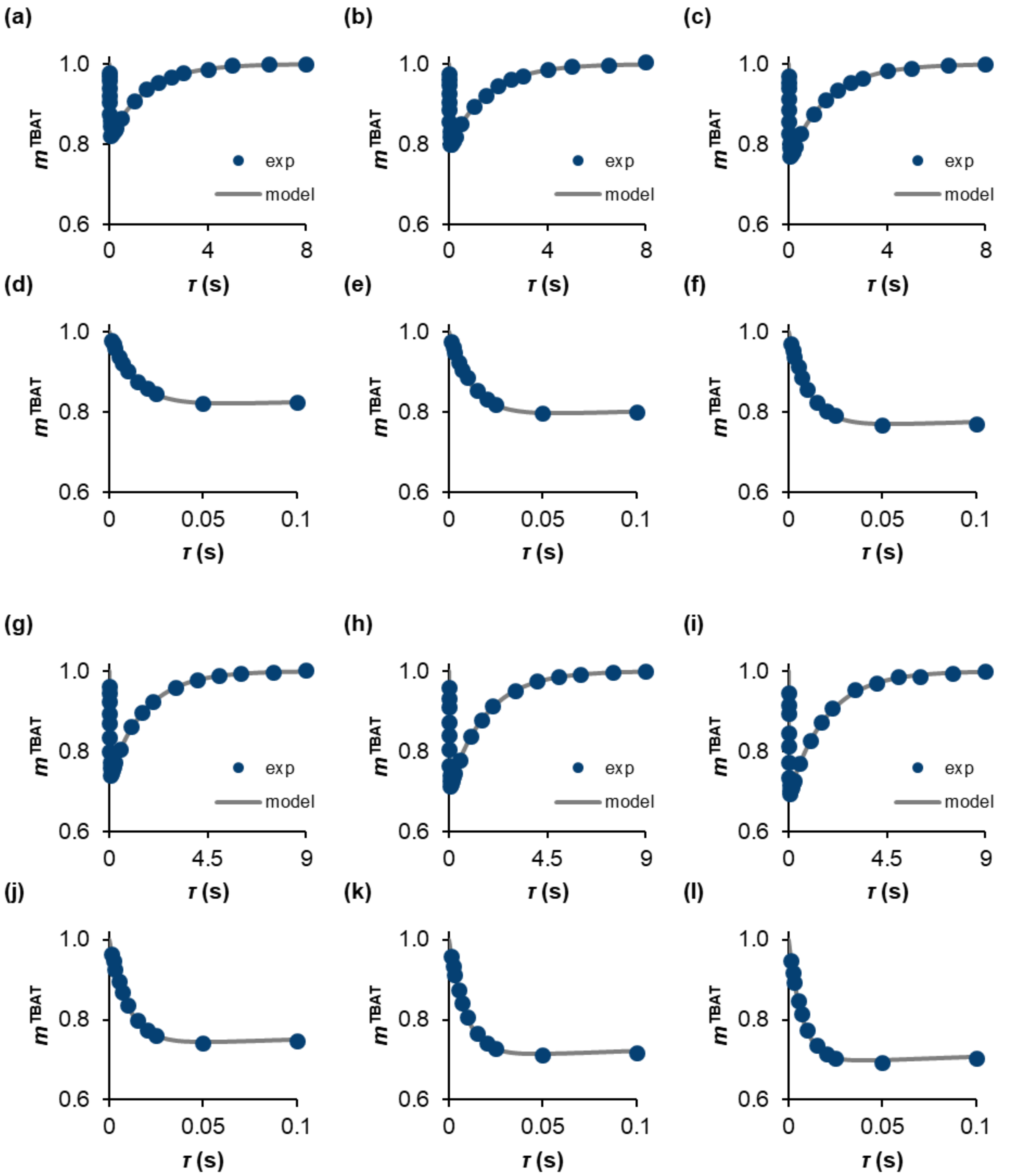

Figure S5.4: [19]F magnetisation transfer profiles of the initially non-inverted TBAT, and expansions of the plots to early variable delays, in titration of TBAT (202 mM) with FTPS (Entry 1 in Table S5.2). (a and d) 97.0 mM FTPS, (b and e) 113 mM FTPS, (c and f) 134 mM FTPS, (g and j) 155 mM FTPS, (h and k) 180 mM FTPS, (i and l) 194 mM FTPS.

The plots of *α* against [FTPS] are presented in Figure S5.5. Titrations of the 52.9 mM and 99.0 mM solutions of TBAT showed linear plots of *α* against [FTPS] with nearly identical slopes of 210 M^{-1} s^{-1} and 217 M^{-1} s^{-1}, respectively. In both cases, the intercepts were slightly negative, and their absolute values were negligible with respect to the slopes (intercept/slope = –1.28 mM and –7.89 mM, respectively). Based on these two datasets, one could therefore postulate

the direct transfer of fluoride from TBAT to FTPS (according to Equation S5.35), with the rate constant of $k_2 \approx 400\ \text{M}^{-1}\ \text{s}^{-1}$ (200 $\text{M}^{-1}\ \text{s}^{-1}$ per exchangeable fluoride), independent of [TBAT]. However, in titrations of the 150 mM and 202 mM solutions of TBAT, only the first five data points gave linear plots of α against [FTPS]. In each case, the solution obtained from the last addition of FTPS exhibited more rapid exchange than expected based on the linearity of the previous measurements. Whilst the slope of the linear part of the plot for 202 mM TBAT (240 $\text{M}^{-1}\ \text{s}^{-1}$) was comparable to the slopes for 52.9 mM and 99.0 mM solutions of TBAT, the slope for the 150 mM solution was much larger (502 $\text{M}^{-1}\ \text{s}^{-1}$). Moreover, the negative intercepts were no longer negligible (intercept/slope = –89.2 mM for 150 mM TBAT and –37.3 mM for 202 mM TBAT). The results obtained from the titrations of the 150 mM and 202 mM solutions of TBAT indicated clearly that the behaviour of the system was more complex than initially assumed. This conclusion was not instantly obvious for the 52.9 mM and 99.0 mM solutions of the reagent. However, α = 17.8 s^{-1} determined for 52.9 mM TBAT and 85.5 mM FTPS in the titration (the fourth aliquot of FTPS, *i.e.* fifth measurement for the sample) was found to be ~50% larger than α = 12.1 s^{-1} determined for the solution in Section S5.2.2, *i.e.* one of nearly identical concentrations of both species (52.5 mM TBAT and 83.9 mM FTPS), but prepared by dissolving the appropriate amounts of both solutes all at once and measured immediately. The upward curvatures in the initially linear plots, considerable negative intercepts of the trend lines in these linear parts, and discrepancy between α determined for two solutions under apparently the same conditions all suggested that the TBAT/FTPS system in THF may be (with respect to the magnetisation transfer rate between the spins): (i) highly sensitive to conditions, which may induce irreproducibility between samples, and/or (ii) temporally-unstable, with the rate of exchange changing over time. These two possibilities were therefore examined next.

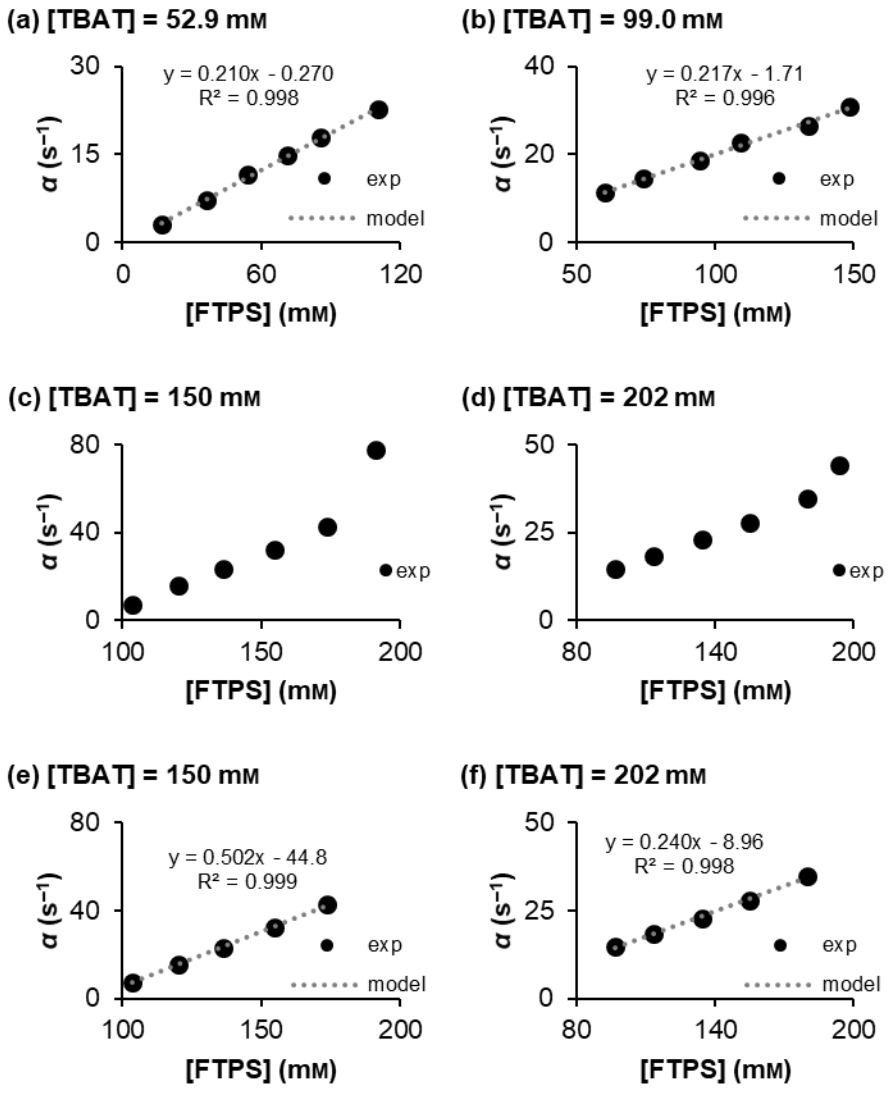

Figure S5.5: Plots of *α* against [FTPS] in titrations of a solution of (a) 52.9 mM TBAT, (b) 99.0 mM TBAT, (c) 150 mM TBAT and (d) 202 mM TBAT in THF with solid aliquots of FTPS. To the plots for (e) 150 mM TBAT, and (f) 202 mM TBAT, linear trend lines ("model") were fitted to data after discarding the last data point (corresponding to the largest [FTPS]) in each set.

S5.2.4. Condition-sensitivity and temporal instability of the system in THF

<u>A representative example of an experiment for the measurement of magnetisation transfer rate increase between TBAT and FTPS in THF over time</u>

A solution was prepared in the glovebox by weighing TBAT (52.8 mM), FTPS (49.8 mM) and NAPH (250 mM) into a 1 mL volumetric flask and dissolving them in THF up to the 1 mL mark. The solution was then split into two parts, which were transferred to J Young® valve capped NMR tubes. The date and time of preparation of the solution was noted. One sample was immediately removed from the glovebox and kept in the spectrometer autosampler between

NMR measurements (sample "AS"). The other one remained in the glovebox, except when NMR measurements were performed ("sample GB").

Eleven sets of ^{19}F pulse-acquire, ^{19}F inversion-recovery and ^{19}F selective inversion-recovery experiments were performed at 300 K on the AS sample, and three sets on the GB one. The variable delays used in the inversion-recovery experiment were, τ (s) {0.1, 0.2, 0.5, 0.75, 1, 2, 3, 4, 6, 8, 10, 12}. The variable delays used in the selective inversion-recovery experiment were, τ (s) {0.001, 0.002, 0.003, 0.005, 0.007, 0.01, 0.015, 0.02, 0.025, 0.05, 0.1, 0.15, 0.2, 0.25, 0.5, 1, 2, 3, 4, 5, 6, 8, 10, 12}.

The date and time of acquisition of each set (more specifically, of the ^{19}F selective inversion-recovery experiment in a given set) were converted to the time interval between preparation of the sample and the acquisition, *t*. The concentrations of TBAT and FTPS based on the NAPH internal standard, as well as the ^{19}F longitudinal relaxation fitting parameters (I_{eq}, x and T_1^{obs}) and root mean square errors (R) are presented in Table S5.4 for both samples at each time, *t*.

Table S5.4: Concentrations of TBAT and FTPS, and longitudinal relaxation fitting parameters (and *R*) for AS and GB samples.

Sample	*t* (h)	[TBAT] (mM)	[FTPS] (mM)	I_{eq}	x	T_1^{obs} (s)	R
AS	0.414	52.3	48.2	780	1.40	2.04	8
AS	1.40	52.1	47.4	792	1.39	2.10	8
AS	2.40	51.5	46.7	791	1.38	2.11	4
AS	3.34	51.2	46.6	867	1.38	2.10	5
AS	4.40	52.4	45.0	770	1.39	2.04	7
AS	5.40	51.4	45.6	811	1.38	2.10	6
AS	7.40	50.8	45.1	814	1.37	2.15	4
AS	14.1	52.4	46.9	804	1.38	2.09	7
AS	25.9	49.9	43.0	887	1.38	2.11	10
AS	36.8	50.0	44.8	897	1.38	2.14	10
AS	50.5	51.0	44.2	894	1.38	2.11	12
GB	13.6	52.0	46.8	808	1.37	2.15	5
GB	25.4	51.8	45.7	884	1.39	2.08	8
GB	37.3	51.4	45.9	887	1.38	2.11	8
GB	50.1	51.8	45.0	N/A	N/A	N/A	N/A

The ^{19}F exchange fitting parameters, I_{eq}, x and $\alpha(r + 1)$, and root mean square error (R) obtained for (the initially non-inverted) TBAT are presented in Table S5.5. A selection of the magnetisation transfer plots is presented in Figure S5.6.

Table S5.5: ^{19}F magnetisation transfer between TBAT and FTPS in the representative rate increase experiment.

Sample	*t* (h)	I_{eq}	*x*	$\alpha(r + 1)$ (s^{-1})	*R*
AS	0.414	1527	838	26.7	8
AS	1.40	1518	835	33.7	7
AS	2.40	1498	827	42.0	7
AS	3.34	1494	818	49.3	10
AS	4.40	1478	816	56.1	8
AS	5.40	1382	777	62.8	7
AS	7.40	1377	776	72.1	8
AS	14.1	1351	764	89.4	11
AS	25.9	1614	972	104	13
AS	36.8	1616	970	118	14
AS	50.5	1607	966	130	17
GB	13.6	1406	807	77	18
GB	25.4	1524	915	97	12
GB	37.3	1647	984	107	17
GB	50.1	1720	1059	117	29

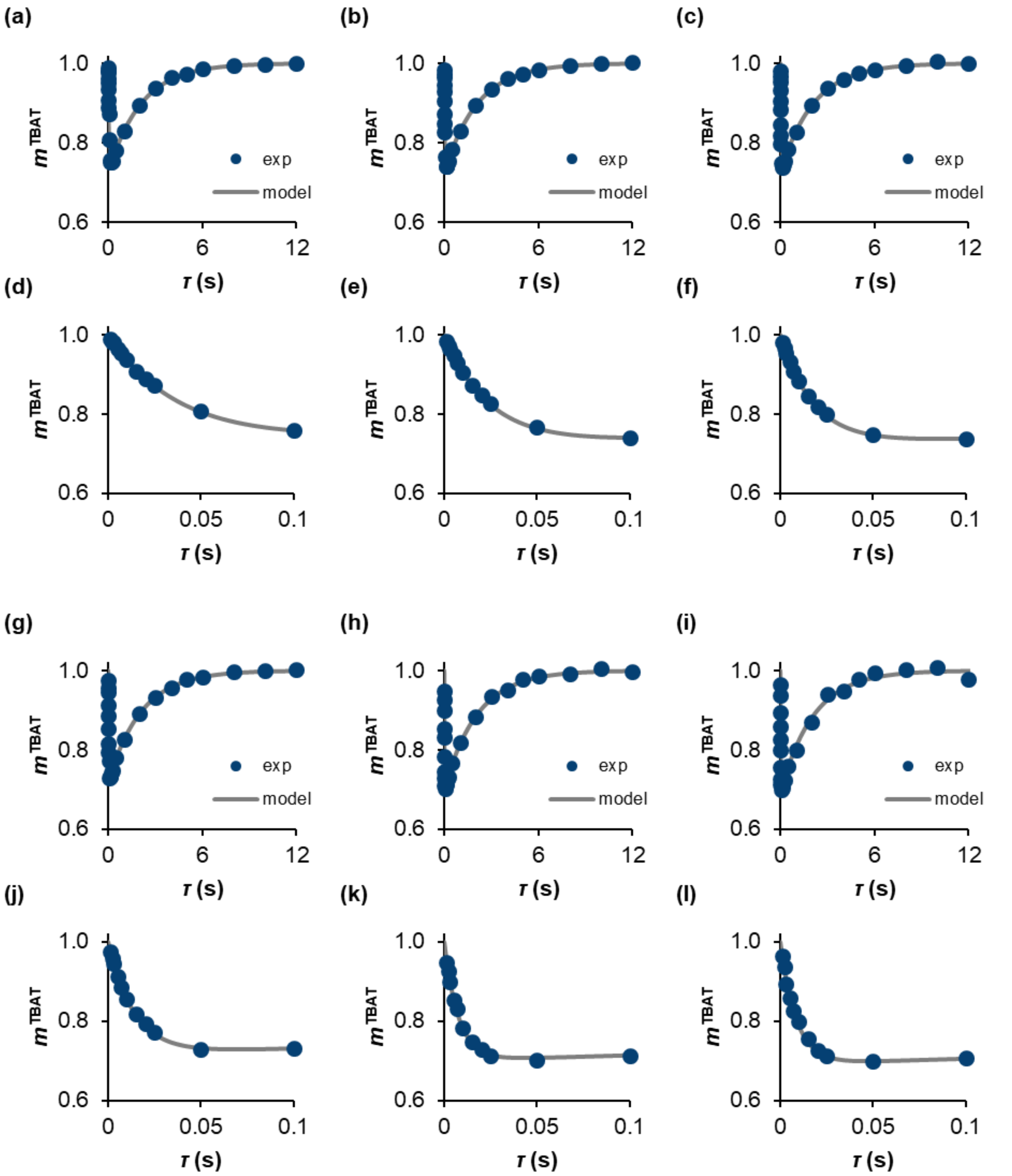

Figure S5.6: ^{19}F magnetisation transfer profiles of the initially non-inverted TBAT, and expansions of the plots to early variable delays, over time; AS: (a and d) 0.414 h, (b and e) 2.40 h, (c and f) 4.40 h, (g and j) 7.40 h, (h and k) 50.5 h; GB: (i and l) 50.1 h.

Temporal evolution of the concentrations, T_1^{obs} and magnetisation transfer rate between TBAT and FTPS are presented in Figure S5.7.

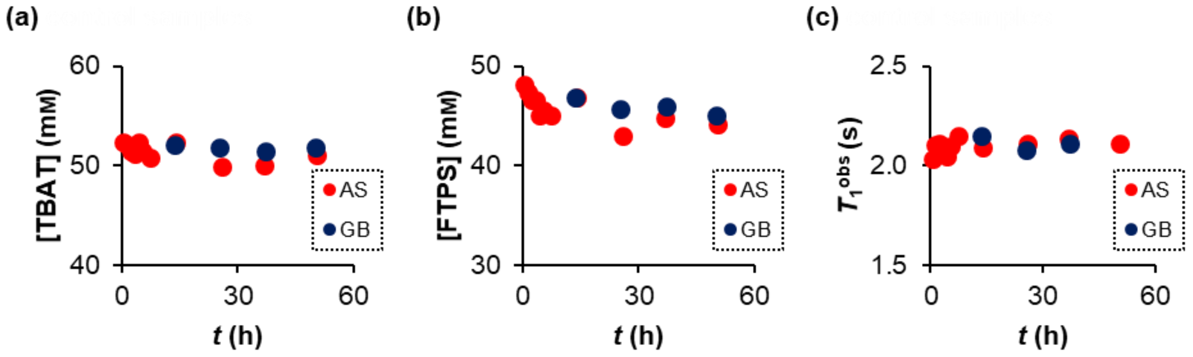

Figure S5.7: Temporal concentration profiles for (a) TBAT and (b) FTPS, and (c) temporal evolution of T_1^{obs} for a mixture of TBAT and FTPS in THF. In all plots, "AS" refers to the sample in a J Young® valve capped NMR tube kept in the autosampler, and "GB" – to the sample in a J Young® valve capped NMR tube stored in the glovebox in between the measurements.

The concentration of TBAT appeared to be constant throughout the experiment, [TBAT] = (51.5 ± 0.4) mM, as did T_1^{obs} = (2.10 ± 0.02) s. However, the plot of [FTPS] against *t* shows apparent decomposition of FTPS initially (a 6.5% decrease of [FTPS] over 7.4 hours), followed by a somewhat noisy plateau for the later measurements, where the absolute difference between the two extreme concentrations is larger than in the first 7.4 hours. It is unclear whether this initial apparent decrease in [FTPS] was due to decomposition. Both signals were found to exhibit some line broadening over time (which in itself was indicative of an increasing exchange rate for the exchanging spins time, Figure S5.8a). For FTPS, the error introduced by broadening could be larger than the one for TBAT, as the signal appears in a region of significant baseline distortion. Moreover, no new signals appeared above the detection limit in the ^{19}F NMR spectra of the solution. We tried to use the longitudinal relaxation time as another way of quantitating [FTPS], as T_1^{obs} is a (known) function of both [TBAT] and [FTPS] in THF (at 300 K). However, as presented in Figure S5.8b, where a recalled plot of experimental T_1^{obs} *vs* *t* from Figure S5.7c is overlaid with theoretical T_1^{obs} calculated from the experimental concentrations of the spins, any potential changes in T_1^{obs} were too small to detect. Figure S5.9a shows that the rate of magnetisation transfer between TBAT and FTPS in the solution increased over time. The plot is initially (the first 5.4 hours; Figure S5.9b) linear with a slope of 7.32 s^{-1} h^{-1} and an intercept of 23.9 s^{-1}. Importantly, the apparent decrease of [FTPS] over the first 5.4 hours is negligible in comparison to the corresponding increase in the observed rate constant of magnetisation transfer, $\alpha(r + 1)$; approximately 5% and 140%, respectively. In other words, this observed increase of the rate of magnetisation transfer over time was a result of the increase of the observed rate constant(s) of exchange, k_1 and/or k_2. Lastly, Figure S5.9c shows the later part of the $\alpha(r + 1)$ *vs* *t* plot, where the rate of the increase of magnetisation transfer rate seemed to decrease, albeit was still linear. Magnetisation transfer in the control sample ("GB"), which was kept inside the glovebox prior to the first measurement at 13.6 hours

(and between any two measurements), showed similar behaviour to the one which was kept in the autosampler (*i.e.* ambient surroundings) at all times. The slopes and intercepts nearly overlayed for both samples (4% and 10% smaller for “GB”, respectively). Therefore, the use of J Young® valve capped NMR tubes offered systems sufficiently gas-tight for the surroundings not to interfere with the samples (*i.e.* the rate increase in both samples was inherent to the solution, and not due to adventitious air or moisture).

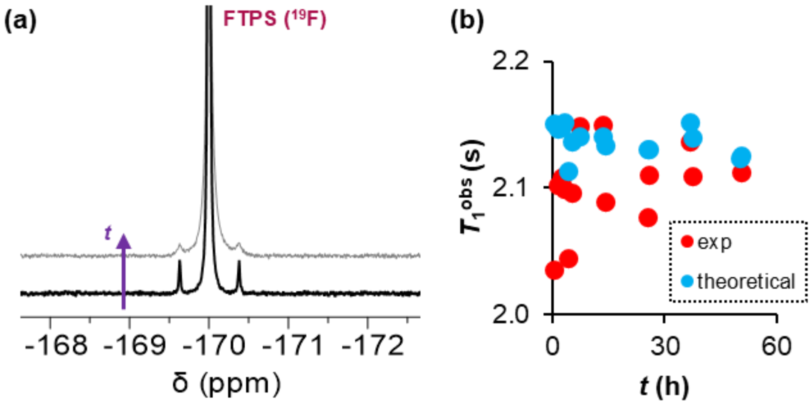

Figure S5.8: (a) Line broadening of the FTPS signal (^{19}F) between the first and last measurement for the “AS” sample (the spectra were phase- and baseline-corrected). (b) Although T_1^{obs} seemed like an attractive alternative to monitoring the broadening signal of FTPS over time, any changes in [FTPS], if present, affected T_1^{obs} negligibly.

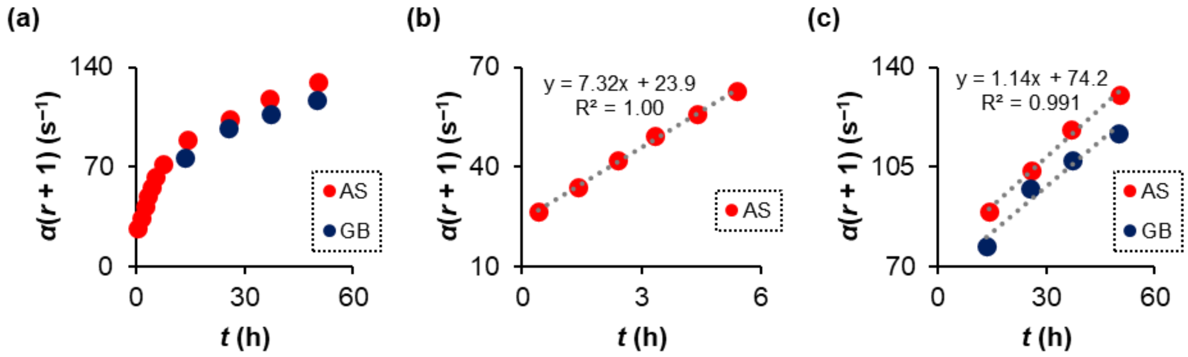

Figure S5.9: (a) Temporal evolution of the rate of magnetisation transfer for a mixture of TBAT and FTPS in THF. (b) An expansion of the plot in (a) showing the first six measurements on the “AS” sample. (c) An expansion of the plot in (a) showing the last four measurements on the “AS” sample, and all four measurements on the “GB” sample. In all plots, “AS” refers to the sample in a J Young® valve capped NMR tube kept in the autosampler, and “GB” – to the sample in a J Young® valve capped NMR tube stored in the glovebox in between the measurements.

<u>Effect of ‘operations’ on NMR tube on the magnetisation transfer rate</u>

A sample was prepared analogously to one above and with similar concentrations of the spins (45.9 mM TBAT and 50.7 mM FTPS). ^{19}F magnetisation transfer rate was again measured over time, this time however, certain operations were performed on the sample, as follows. The first measurement was taken as for the previous solution. Before the second

measurement, the NMR tube was shaken thoroughly for one minute (without opening the system to air). Before the third and the fourth measurements, the NMR tube was introduced into the glovebox, opened to the glovebox atmosphere (N_2) for one minute each time, and then closed again. Before the fifth and the sixth measurements, the NMR tube was also introduced into the glovebox, but was not opened. Instead, the bottom seal of the cap was loosened and, whilst the NMR tube was still closed, the bulb compartment of it was rinsed with its contents (the solution). The bulb compartment was then emptied (back to the bottom of the NMR tube) and the NMR tube resealed fully. Finally before the seventh measurement, the NMR tube was open to air for ~1 s, closed and shaken thoroughly.

The model was fitted to the experimental data – with T_1^{obs} as the fourth fitting parameter; the ^{19}F exchange fitting parameters, I_{eq}, x, T_1^{obs} and $\alpha(r + 1)$, and root mean square error (R) obtained for (the initially non-inverted) TBAT are presented in Table S5.6. The plot of $\alpha(r + 1)$ *vs* t is presented in Figure S5.10, overlaid with the data from the inherent rate increase experiment (“AS”).

Table S5.6: ^{19}F magnetisation transfer between TBAT and FTPS in the ‘operations’ on the NMR tube experiment.

Entry	t (h)	I_{eq}	x	T_1^{obs} (s)	$\alpha(r + 1)$ (s^{-1})	R
1	0.58	1356	830	2.12	20.5	11
2	1.08	1352	831	2.10	25.4	13
3	1.62	1342	822	2.11	28.2	11
4	2.13	1325	811	2.06	31.9	18
5	2.70	1313	804	2.00	36.3	11
6	3.25	1297	788	2.12	66.4	12
7	3.73	1123	674	2.09	154	9

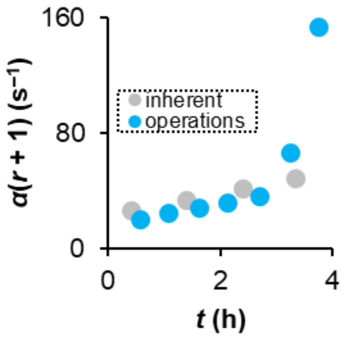

Figure S5.10: Temporal evolution of the magnetisation transfer rate as a response to performing operations on the NMR tube (described in the main text). The corresponding inherent rate increase in the “AS” system from Figure S5.7 is also presented (grey points).

The magnetisation transfer rate constants, $\alpha(r + 1)$, for the first five measurements were a linear function of time, with a nearly identical slope to one for the inherent exchange rate increase (7.21 s^{-1} h^{-1} *cf.* 7.32 s^{-1} h^{-1}; intercept = 16.7 s^{-1}, R^2 = 0.993). This initial increase of $\alpha(r + 1)$ indicated that no rate-increasing factors other than the inherent one(s) observed

previously were present for these five measurements. However, the sixth measurement gave a larger rate constant increase than expected from the initial linearity (66.4 s^{-1} *cf.* expected 40.2 s^{-1}). A possible rationale behind this observation is that the bulb compartments of J Young® valve capped NMR tubes (used throughout this study) are not perfectly sealed from the surroundings. Normally, the seals are good enough, so that even if any air and moisture is present in the "bulb", briefly applying vacuum in the antechamber, prior to introducing the NMR tube into the glovebox, removes it. Therefore, the first rinsing of the bulb compartment with the solution did not introduce any air and moisture to the system. However, it left a trace amount of the solution in the bulb compartment, which absorbed the air and moisture when the NMR tube was removed from the glovebox for the fifth measurement. This trace amount of the compromised solution was then introduced back into the sample in the second rinsing (prior to the sixth measurement). The absorbed water had a catalysing effect on the exchange rate between the spins. Finally, even very briefly opening the sample to ambient atmosphere resulted in a dramatic increase in the magnetisation transfer rate constant (154 s^{-1} *cf.* expected 43.7 s^{-1}).

Effect of additives on the magnetisation transfer rate

A series of solutions of ~50 mM TBAT and ~50 mM FTPS were prepared with certain additives/modifications, and the response of each system to the applied change was monitored over time, analogously to the experiment described above ('AS' sample). Additives which had little effect on the temporal evolution of the magnetisation transfer rate include 2,6-di-*tert*-butylpyridine (DTBP, ~20 mM), powdered glass (~40 mg) and TMS_2O (~20 mM); Figure S5.11a (overlaid with the data for "AS", grey points). Using a dried Teflon insert had a dramatic effect on the increase of magnetisation transfer rate over time, as presented in Figure S5.11b ("AS" data again included in grey). Initially (t = 0.550 h), $\alpha(r + 1)$ = 30.4 s^{-1} was similar to the value obtained for "AS" (26.7 s^{-1} at t = 0.414 h). It then increased very rapidly to 488 s^{-1} at t = 12.9 h, *i.e.* by approximately 16-fold over 12 hours (*cf.* an approximately 3-fold increase for "AS" over a similar period). Repeating the magnetisation transfer measurements for a further 20 h showed that an apparent stability with respect to the magnetisation transfer rate was achieved over this period. An interesting temporal profile was obtained for the solution in the presence of 3 Å molecular sieve beads (3Å-MS, ~40 mg), whereby the magnetisation transfer rate initially increased and then decreased with time; Figure S5.11c. A control sample (no MS added initially) showed the expected increase of $\alpha(r + 1)$ over time. However, when molecular sieves were added (t ≈ 24 h) to the control sample (the sample was then shaken thoroughly for ~5 min and the magnetisation transfer was measured immediately), $\alpha(r + 1)$ decreased from 58.1 s^{-1} to 9.69 s^{-1}, much closer to $\alpha(r + 1)$ = 7.09 s^{-1} for the sample with MS present initially (although still ~40% larger). Nevertheless, this experiment showed the reversibility of

the process which caused the magnetisation transfer between the spins to increase over time in the absence of MS.

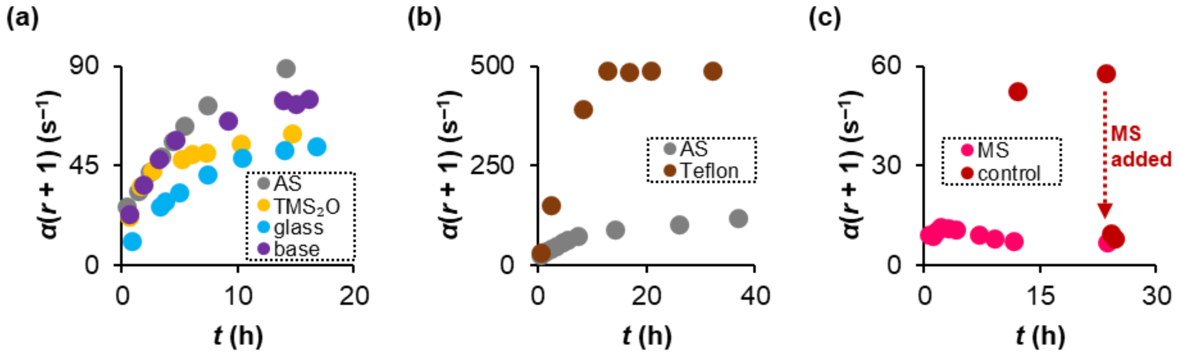

Figure S5.11: Effect of additives on the temporal evolution of the TBAT/FTPS magnetisation transfer. (a) DTBP, powdered glass and TMS_2O had little effect on the increase of $\alpha(r + 1)$ over time. (b) The solution placed in a Teflon insert exhibited a dramatic increase of $\alpha(r + 1)$ to nearly 500 s^{-1} over 12 hours, followed by apparent stabilisation of the magnetisation transfer rate for a further 20 h. (c) In the presence of 3Å-MS, $\alpha(r + 1)$ initially increased, followed by a maximum at $t \approx 2$ h, and a subsequent decrease. Notably, addition of 3Å-MS to a control sample reversed the magnetisation transfer rate increase from prior to the addition.

However, a combination of DTBP (~20 mM) and 3Å-MS (~40 mg) caused the magnetisation transfer rate in the system (50 mM TBAT, 45 mM FTPS) to initially decrease, eventually heading towards temporal stability (Figure S5.12).

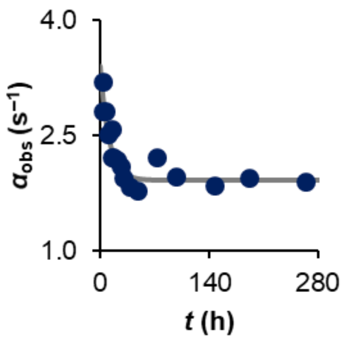

Figure S5.12: The heading towards temporal stability of the TBAT/FTPS exchange rate in the presence of DTBP and MS.

An empirical exponential decay model was fitted to the experimental data with three fitting parameters: α_0 (α immediately after sample preparation), α_s (α in the stabilised system, *i.e.* the value of the plateau) and k_s (the rate constant of the "slowing down" of the exchange); Equation S5.63. The determined fitting parameters were: α_0 = 3.40 s^{-1}, α_s = 1.92 s^{-1}, k_s = 0.0885 h^{-1}. The root mean square error, R = 0.12 s^{-1} (8.1% of the absolute difference between α_0 and α_s), indicated an excellent fit to the experimental data, despite poor shimming resulting from the heterogeneity of the system.

S5.63

$$\alpha_{\mathrm{obs}} = (\alpha_0 - \alpha_{\mathrm{s}})e^{-k_{\mathrm{s}}t} + \alpha_{\mathrm{s}}$$

Once the system was stable with respect to the exchange rate, the NMR tube was shaken thoroughly for ten minutes, which did not seem to affect the system. Neither the addition of more DTBP (further ~20 mM), nor 3Å-MS (further ~20 mg), disturbed the stability of the system, although in both cases α_{obs} changed slightly (11% above and below the model plateau, α_{s}, respectively). These results are presented in Figure S5.13.

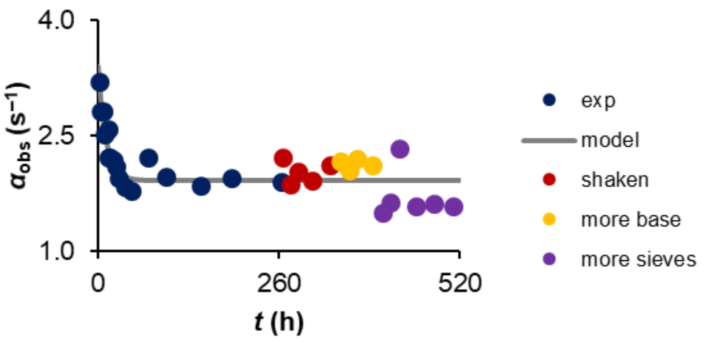

Figure S5.13: Relative insensitivity of the stabilised system to shaking the NMR tube, as well as the addition of more DTBP ("base") and 3Å-MS ("sieves").

Effect of exogenous water on the magnetisation transfer rate

A solution of 59.2 mM TBAT and 67.3 mM FTPS (~0.4 mL in a J Young® valve capped NMR tube) was titrated with five (<1 μL) aliquots of a 129 mM solution of water in THF (in the presence of PhF, used to quantify added water). Magnetisation transfer between TBAT and FTPS was measured prior to the titration and for each aliquot, and a plot of $\alpha_{\mathrm{obs}}(r + 1)$ against the concentration of added water, [H_2O], is presented in Figure S5.14a. The same $\alpha_{\mathrm{obs}}(r + 1)$ data plotted against time (t, from sample preparation) is presented in Figure S5.14b. The magnetisation transfer rate increase between each two measurements was large relative to the inherent rate increase over time (7.32 s^{-1} h^{-1} determined for "AS"). A line of this slope going through the first data point in Figure S5.14b illustrates the extent of this difference. Therefore, it was assumed that the increase of $\alpha_{\mathrm{obs}}(r + 1)$ was solely a result of the added water in the titration.

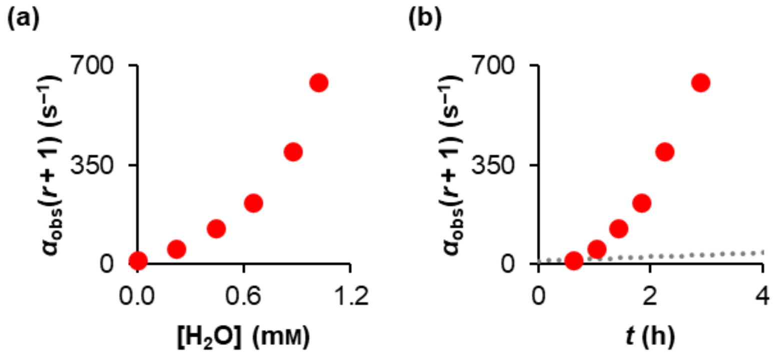

Figure S5.14: (a) Titration of the TBAT/FTPS system in THF with water (at 300 K). (b) The experimental $\alpha_{obs}(r + 1)$ from (a) plotted against *t*, and compared to a linear $\alpha_{obs}(r + 1)$ *vs t* model with a slope of 7.32 s^{-1} h^{-1} going through the first experimental data point.

The order of the reaction with respect to water was determined by assuming a linear dependence of α_{obs} on $[H_2O]^x$, where x is the empirical order with respect to water. The coefficients α_0 and α_{cat} correspond to the background process (unaffected by water) and the water-catalysed process, respectively, Equation S5.64. From the initial measurement (no added water), $\alpha_0(r + 1) = 17.2\ s^{-1}$. The best linear fit was obtained for $x = 2.42 \approx 2$, Figure S5.15; $\alpha_{cat}(r + 1) = 577$, $R^2 = 0.989$; thus indicating that the process predominantly involves interaction with a water dimer, or sequential reaction with two water molecules.

S5.64

$$\alpha_{\text{obs}}(r + 1) = (\alpha_0 + \alpha_{\text{cat}}[\text{H}_2\text{O}]^x)(r + 1)$$

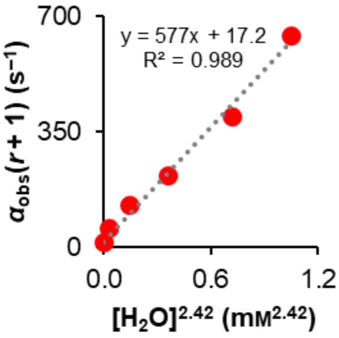

Figure S5.15: The order with respect to water estimated by fitting the model of Equation S5.64 to the experimental data.

S5.2.5. Effect of [FTPS] on exchange rate in the stabilised system in THF

Five solutions were prepared in the glovebox by weighing TBAT, FTPS and NAPH into 1 mL volumetric flasks and dissolving them in THF up to the 1 mL mark. DTBP (5.00 µL, 23.1 mM) was added to each solution *via* a microsyringe, and each solution was shaken thoroughly. The solutions were then transferred to J Young® valve capped NMR tubes (~0.5 mL, each) preloaded with six 3 Å molecular sieve beads (3Å-MS, ~40 mg), and again shaken thoroughly.

The resulting concentrations in the solutions are presented in Table S5.7. The date and time of preparation of each solution was also noted.

Table S5.7: Concentrations of TBAT, FTPS and NAPH in the solution for measurements of exchange rate in the stabilised system.

Entry	Solvent	Concentration (mM)		
		TBAT	FTPS	NAPH
1	THF	101	22.6	363
2	THF	101	62.9	431
3	THF	101	101	279
4	THF	101	144	319
5	THF	101	204	349

Ten sets of ^{19}F pulse-acquire, ^{19}F inversion-recovery and ^{19}F selective inversion-recovery experiments were performed at 300 K for each solution. The variable delays used in the inversion-recovery experiments were, τ (s) {0.1, 0.2, 0.5, 0.75, 1, 2, 3, 4, 6, 10, 15, 20}. The variable delays used in the selective inversion-recovery experiments were, τ (s) {0.001, 0.002, 0.005, 0.01, 0.015, 0.02, 0.025, 0.035, 0.05, 0.075, 0.1, 0.15, 0.2, 0.25, 0.3, 0.4, 0.5, 1, 3, 5, 8, 12, 15, 20}.

The date and time of acquisition of each set (more specifically, of the ^{19}F selective inversion-recovery experiment in a given set) were converted to the time interval between preparation of the sample and the acquisition, *t*.

The concentrations of TBAT and FTPS, based on the NAPH internal standard, as well as the ^{19}F longitudinal relaxation fitting parameters (I_{eq}, x and T_1^{obs}) and root mean square errors (R) are presented in Table S5.1 for each solution at each time, *t* and the corresponding temporal evolutions – in Figure S5.16. The plots of [TBAT] against time show that [TBAT] was indeed equal between the solutions, and constant over the duration of the experiment (340 h from preparation of the samples). A closer examination of the expanded plot showed a possibility of a low-degree of decomposition of TBAT over time for the solutions containing 22.6 mM and 101 mM FTPS, where linear trend lines give very slightly negative slopes (albeit with low R^2). It is unclear whether the apparent decrease of [TBAT] for these two solutions was a result of decomposition or experimental error (perhaps from poor shimming). However, no decrease was observed for the remaining three solutions and hence the latter seemed likely. Even if decomposition was responsible for the apparent decrease of [TBAT] in the two solutions, only 1.4% and 1.7% of TBAT decomposed in the 22.6 mM and 101 mM FTPS solutions, respectively, based on the linear trend lines. Therefore, the concentration of TBAT was assumed to be equal and independent of time for all five systems. An average of all the 50 experimental concentrations was taken as the “true” concentration in these experiments, [TBAT] = (99.1 ± 0.2) mM, which is in excellent agreement with the theoretical 101 mM based

on mass of the solute (98% of the theoretical value). The analysis of [FTPS] against time was slightly more ambiguous. The plots (Figure S5.16) show that likely FTPS decomposed in at least some of the solutions. In the solution which contained 22.6 mM FTPS based on the mass of the solute, a decrease from [FTPS] = 22.6 mM to [FTPS] = 17.5 mM (23%) was observed over 340 h, based on quantitation against NAPH. The decomposition profile appears to be relatively linear (R^2 = 0.86), with an intercept of 22.3 mM (98% of the theoretical value) and a slope of –0.0142 mM h^{-1}. The linear decomposition model of Equation S5.65 was therefore used to determine [FTPS] at each time *t* for this solution. The other four solutions showed more ambiguity in whether FTPS decomposes. Whilst the 101 mM and 204 mM solutions show scatter, it may be argued that a slight decrease in [FTPS] was observed for the 62.9 mM and 144 mM solutions (5.0% and 3.2%, respectively, based on the linear trend lines). Nonetheless, the difference between the highest and lowest [FTPS] determined for each of these solutions was between 3.5% and 7.4% of the corresponding concentrations by mass of the solute. Any potential decrease in [FTPS] in these four solutions was therefore negligible compared to the differences in [FTPS] between them. As a result, the concentration of FTPS was assumed to be independent of time for the four solutions which contained 62.9–204 mM FTPS by mass of the solute. An average of all 10 values from quantitation of FTPS against NAPH for each solution was taken as the "true" concentration in that solution, [FTPS] (mM) {(60.8 ± 0.9), (100 ± 1), (143 ± 1), (202 ± 1)}; all of these values are in excellent agreement with the theoretical concentrations based on mass of the solute (97–99% of the corresponding theoretical values).

S5.65

$$\frac{[\mathrm{FTPS}]}{\mathrm{mM}} = -0.0142t + 22.3$$

The average T_1^{obs} (s) {(1.46 ± 0.01), (1.59 ± 0.03), (1.70 ± 0.05), (1.87 ± 0.05), (2.10 ± 0.05)} from all ten measurements for each solution, presented in the order of increasing [FTPS], are also in excellent agreement with the expected values based on the weighted averages of T_1^{TBAT} and T_1^{FTPS} as isolated spins (4–16% lower than the corresponding expected values). The plots of T_1^{obs} against *t* show a slight decrease of T_1^{obs} over the 340 hours of monitoring. Despite the observed decrease, T_1^{obs} determined from the non-selective inversion-recovery experiments were directly used in the fitting of the magnetisation transfer model to the corresponding measurements (*i.e.* in the same set of experiments). Firstly, T_1^{obs} may be dependent on even slight changes in conditions and hence the noise in the T_1^{obs} *vs t* plots may not represent experimental errors but rather true T_1^{obs} at the time of the measurement. Secondly, a (significant) error in T_1^{obs} used in the magnetisation transfer model would show in the magnetisation transfer plot and can be easily corrected by refitting the model with T_1^{obs} as the fourth unknown parameter.

Table S5.8: Quantitation of the spins and longitudinal relaxation fitting parameters under the stabilising conditions.

t (h)	[TBAT] (mM)	[FTPS] (mM)	I_{eq}	x	T_1^{obs} (s)	R
2.36	99.3	22.6	1634	1.60	1.50	8
26.7	100	20.5	1475	1.75	1.48	20
75.8	98.8	21.7	1799	1.54	1.47	18
100	98.3	21.9	1805	1.56	1.46	31
123	99.1	20.5	1763	1.62	1.43	38
148	99.4	19.7	1791	1.56	1.48	18
168	99.4	19.8	1843	1.57	1.45	14
232	98.4	18.9	1493	1.76	1.45	15
284	98.1	18.0	1609	1.74	1.47	9
340	98.3	17.5	1676	1.75	1.45	10
1.69	99.8	62.3	1644	1.45	1.63	35
25.8	99.6	60.8	1483	1.56	1.66	27
75.1	99.8	59.7	1821	1.39	1.62	25
97.8	98.9	61.6	1842	1.41	1.63	47
122	99.7	62.9	1815	1.45	1.56	32
147	99.7	61.2	1783	1.49	1.52	66
167	101	61.5	1840	1.44	1.54	35
224	99.2	60.9	1601	1.58	1.57	40
284	99.5	58.8	1490	1.59	1.57	36
339	100	58.3	1687	1.60	1.56	35
0.741	99.6	100	1589	1.32	1.82	24
24.7	99.1	101	1449	1.42	1.82	28
73.7	98.8	100	1738	1.28	1.76	42
96.7	98.5	101	1615	1.34	1.73	55
121	98.1	102	1676	1.39	1.58	72
145	97.7	99.0	1758	1.37	1.66	77
165	98.7	102	1798	1.36	1.70	58
223	98.8	96.9	1520	1.48	1.66	54
283	98.1	98.9	1531	1.49	1.63	55
339	97.1	98.6	1617	1.48	1.66	49
3.16	98.7	146	1610	1.19	2.00	15
27.6	99.5	142	1462	1.32	1.93	38
76.6	100	146	1802	1.18	1.93	38
101	98.8	145	1809	1.19	1.93	39
124	99.3	144	1769	1.24	1.86	63
149	98.0	144	1793	1.23	1.87	52
144	98.6	142	1807	1.24	1.82	60
233	99.7	141	1472	1.36	1.78	56
285	98.4	141	1428	1.37	1.80	55
341	99.5	141	1635	1.36	1.80	58
1.56	99.1	203	1611	1.10	2.17	22
25.6	99.5	199	1470	1.16	2.28	12
75.0	99.7	204	1537	1.11	2.03	51
97.6	98.5	204	1594	1.09	2.15	28
122	98.6	206	1753	1.13	2.08	57
146	100	201	1787	1.10	2.07	49
167	98.3	202	1836	1.12	2.07	39
224	99.6	200	1556	1.22	2.06	55
284	99.4	203	1597	1.22	2.04	53
339	99.0	199	1643	1.22	2.03	59

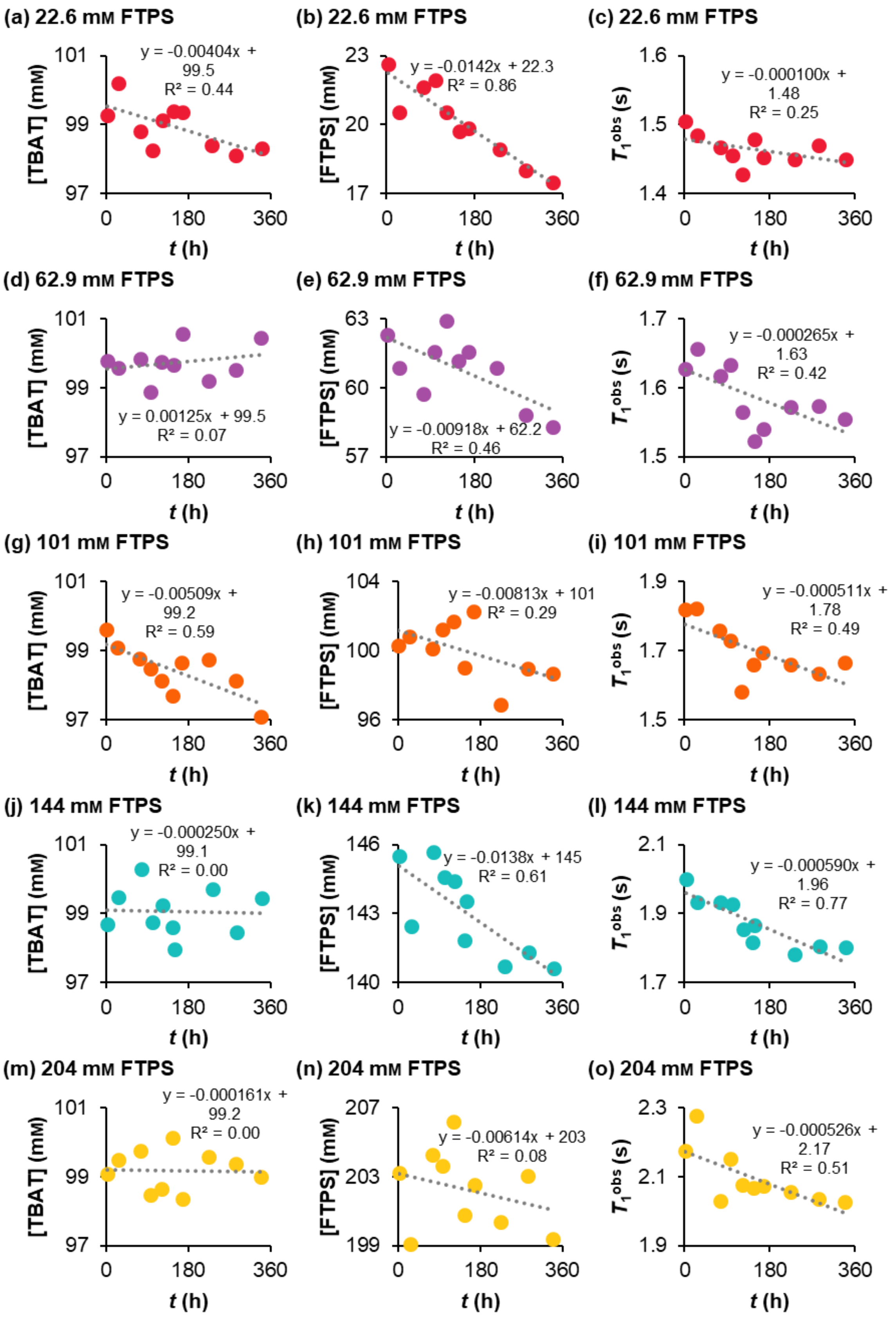

Figure S5.16: Temporal evolution of [TBAT], [FTPS], and the averaged longitudinal relaxation time of the spins, T_1^{obs}, under the stabilising conditions. (a–c) 22.6 mM FTPS; (d–f) 62.9 mM FTPS; (g–i) 101 mM FTPS; (j–l) 144 mM FTPS; (m–o) 204 mM FTPS.

The ^{19}F exchange fitting parameters (I_{eq}, x and α_{obs}) and root mean square errors (R) obtained for (the initially non-inverted) TBAT are presented in Table S5.1. A selection of the magnetisation transfer plots is presented in Figure S5.17.

Table S5.9: Evolution of magnetisation transfer rate under stabilising conditions.

t (h)	[TBAT] (mM)	[FTPS] (mM)	I_{eq}	x	α_{obs} (s^{-1})	R
2.36	99.3	22.6	2400	480	2.05	31
26.7	100	20.5	2942	598	1.54	32
75.8	98.8	21.7	2293	451	1.08	13
100	98.3	21.9	2272	409	1.23	18
123	99.1	20.5	2783	506	1.17	18
148	99.4	19.7	2840	474	1.24	26
168	99.4	19.8	2936	509	1.37	17
232	98.4	18.9	2983	528	1.23	6
284	98.1	18.0	2927	510	1.28	4
340	98.3	17.5	3350	556	1.31	4
1.69	99.8	62.3	2388	1097	3.89	36
25.8	99.6	60.8	2948	1413	3.32	31
75.1	99.8	59.7	2297	1058	2.46	19
97.8	98.9	61.6	2322	1007	3.01	26
122	99.7	62.9	2875	1283	2.29	32
147	99.7	61.2	2831	1267	2.23	23
167	101	61.5	2927	1282	2.40	19
224	99.2	60.9	2900	1359	2.22	19
284	99.5	58.8	2967	1397	2.39	15
339	100	58.3	3360	1579	2.52	16
0.741	99.6	100	2369	1496	5.76	12
24.7	99.1	101	2693	1823	4.75	41
73.7	98.8	100	2195	1414	3.60	24
96.7	98.5	101	2372	1475	4.10	49
121	98.1	102	2724	1763	3.29	24
145	97.7	99.0	2799	1777	3.53	17
165	98.7	102	2841	1831	3.44	30
223	98.8	96.9	2771	1876	3.28	18
283	98.1	98.9	2808	1903	3.26	21
339	97.1	98.6	3228	2159	3.51	22
3.16	98.7	146	2380	1818	9.58	27
27.6	99.5	142	2864	2338	9.65	25
76.6	100	146	2277	1806	5.64	21
101	98.8	145	2307	1713	5.29	23
124	99.3	144	2809	2185	4.83	42
149	98.0	144	2837	2170	4.84	26
144	98.6	142	2875	2232	4.64	27
233	99.7	141	2917	2434	4.35	38
285	98.4	141	2835	2384	4.45	28
341	99.5	141	3275	2748	4.60	48
1.56	99.1	203	2390	2223	10.9	20
25.6	99.5	199	2899	2796	15.5	34
75.0	99.7	204	3429	3283	8.08	50
97.6	98.5	204	2307	2087	9.21	28
122	98.6	206	2735	2529	7.46	49
146	100	201	2841	2587	7.87	40
167	98.3	202	2864	2646	7.38	35
224	99.6	200	2806	2784	6.78	34
284	99.4	203	2850	2836	6.66	39
339	99.0	199	3238	3220	6.74	58

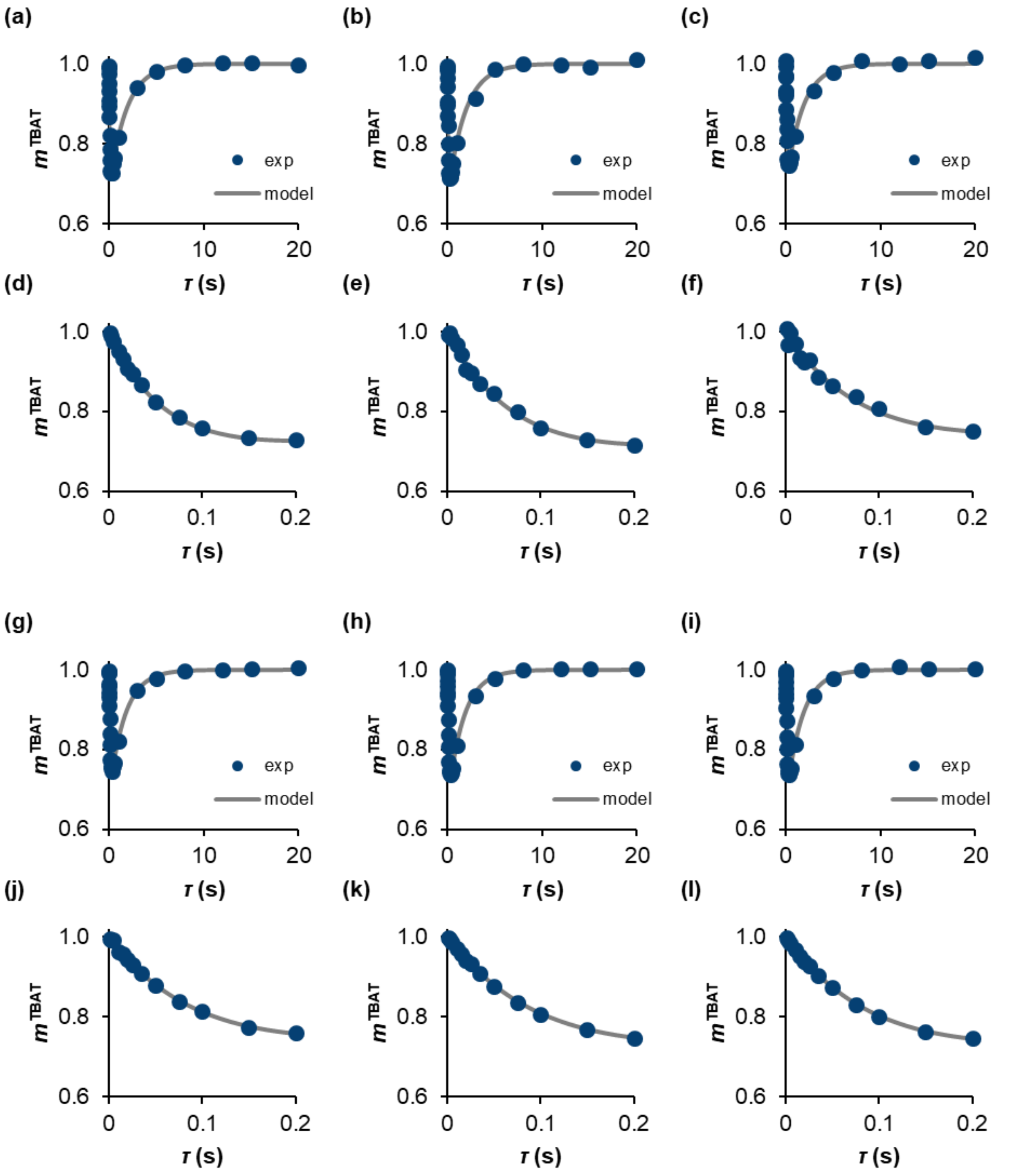

Figure S5.17: **^{19}F magnetisation transfer profiles of the initially non-inverted TBAT, and expansions of the plots to early variable delays, over time; 101 mM FTPS (Entry 3 in Table S5.7): (a and d) 0.741 h, (b and e) 24.7 h, (c and f) 96.7 h, (g and j) 145 h, (h and k) 223 h, (i and l) 339 h.**

All five solutions eventually reached temporal stability of α_{obs} (Figure S5.18), the dissociative *vs* direct transfer pathways in these now stabilised systems could be interrogated. The exponential decay model from Equation S5.63 was fitted to the experimental data (dotted lines in plots in Figure S5.18). The fitting parameters and root mean square errors (*R*) are presented in Table S5.10.

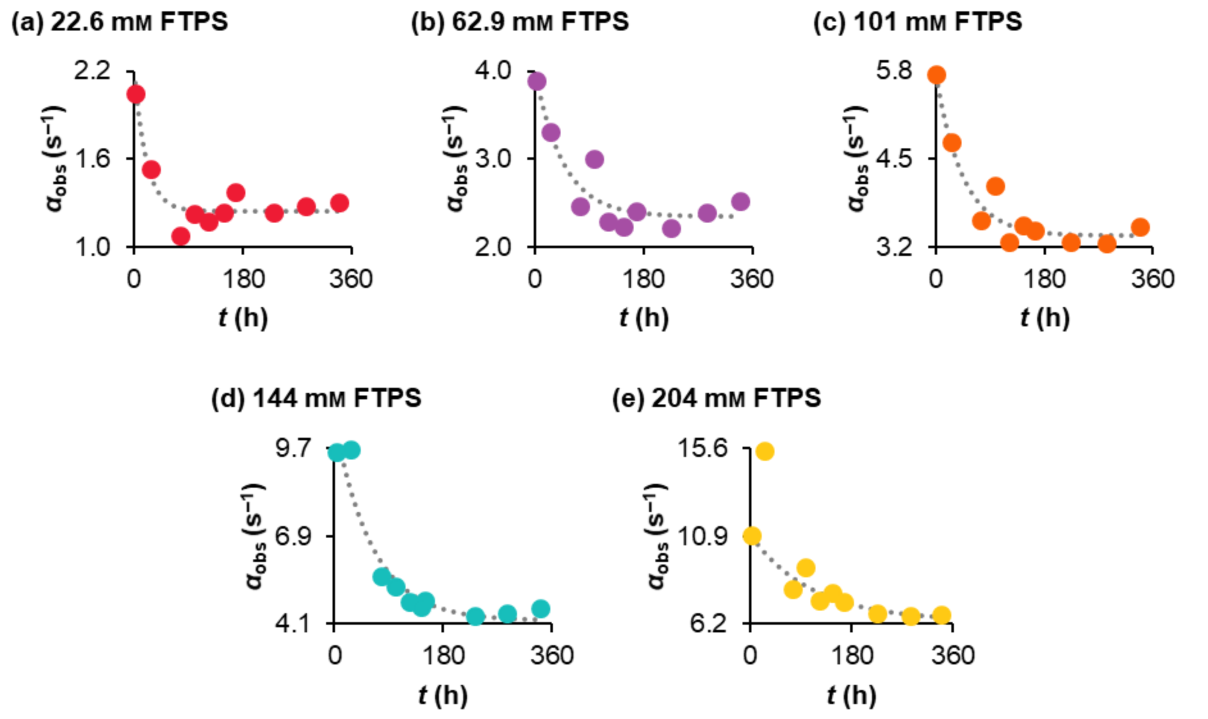

Figure S5.18: Heading towards temporal stability of the stabilised systems.

Table S5.10: Determined fitting parameters and root mean square errors in the exponential decay of the observed exchange rate constant in the stabilised system.

[FTPS] (mM)	α_0 (s^{-1})	α_S (s^{-1})	k_S (h^{-1})	R (s^{-1})
18.1	2.16	1.24	0.0498	0.08
60.8	3.96	2.34	0.0211	0.19
100	5.79	3.37	0.0224	0.19
143	10.6	4.22	0.0158	0.53
202	11.0	6.39	0.00907	0.42

Two issues are noted regarding these models. Firstly, the second data point in α_{obs} *vs* t for the 204 mM FTPS solution was treated as an outlier and discarded from the fitting, as discarding it gave a better fit for the last five data points. Secondly, although it was established that [FTPS] decreased by 23% overall in the 22.6 mM FTPS solution, the last three data points in the α_{obs} *vs* t plot correspond to 18.9 mM, 18.0 mM and 17.5 mM FTPS. The experimental values of α_{obs} for these three concentrations are in good agreement with one another and the model α_S determined for the solution (average of 1.27 s^{-1} compared to model α_S = 1.24 s^{-1}). It was therefore assumed that for this solution, once "stabilised", α_S = 1.24 s^{-1} and [FTPS] = 18.0 mM (the average of the last three concentrations).

Finally, α_S was found to be a linear function of [FTPS] with slope = 27.3 M^{-1} s^{-1}, intercept = 0.656 s^{-1} and R^2 = 0.989. This plot is presented in Figure S5.19a. According to Equation S5.66 (similar to Equation S5.35, except the subscript "s" was given to α), the slope of the plot corresponds to half the rate constant of the direct transfer pathway, $k_2/2$, and the intercept –

to half the rate constant of the dissociative pathway, $k_1/2$; thus k_2 = 55 M^{-1} s^{-1}, k_1 = 1.3 s^{-1}. Notably, this result shows that at least in the temporally-stabilised system (*via* addition of 3Å-MS and DTBP), the dissociative and direct transfer pathways proceed in parallel in the transfer of fluorine between TBAT and FTPS, and the contribution of each pathway depends on [FTPS], Equation S5.67 (where $c_{FTPS}°$ = 1 M is the standard concentration). For example, at 1 M FTPS the transfer is near-exclusively direct (>97%), while at 1 mM FTPS it is near-exclusively dissociative (>95%).

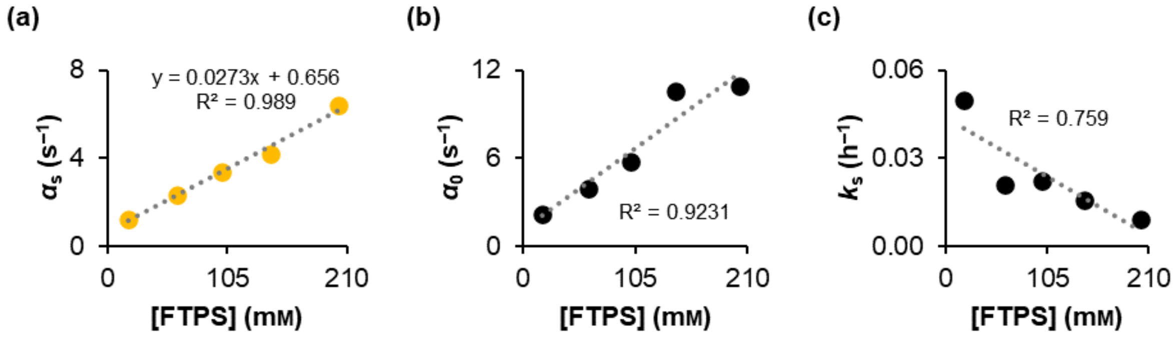

Figure S5.19: (a) The interrogation of the dissociative and direct fluoride transfer pathways in the stabilised TBAT/FTPS systems. (b and c) In comparison, irreproducibility is observed prior to the systems reaching stability.

S5.66

$$\alpha_s = \frac{k_1 + k_2[\text{FTPS}]}{2}$$

S5.67

$$\frac{k_2 c_{\text{FTPS}}°}{k_1} = 42$$

The effect of the added DTBP and 3Å-MS on the stability and reproducibility of the systems is especially pronounced in comparison with corresponding plots of α_0 against [FTPS] and k_s against [FTPS] (Figure S5.19b–c), which do not show linear correlations.

S5.2.6. Proof of dissociative pathway in the non-stabilised system in THF

A solution was prepared in the glovebox by weighing TBAT (222 mM) and NAPH (269 mM) into a 1 mL volumetric flask and dissolving them in THF up to the 1 mL mark. The solution was transferred to a J Young® valve capped NMR tube. Seven solid aliquots of FTPS were added to the solution. After each aliquot addition, the NMR tube was shaken thoroughly, and ^{19}F pulse-acquire and ^{19}F selective inversion-recovery experiments were run at 300 K. The variable delays used in the experiments were, τ (s) {0.001, 0.002, 0.005, 0.01, 0.015, 0.02, 0.025, 0.035, 0.05, 0.075, 0.1, 0.15, 0.2, 0.25, 0.3, 0.4, 0.5, 0.75, 1, 2, 3, 4, 5.5, 7}. Last acquisition ended ~1.5 h after the solution was prepared.

The concentration of TBAT, based on NAPH, was [TBAT] = 218 mM. The concentrations of FTPS are presented in Table S5.11. The ^{19}F selective inversion-recovery spectral sets were processed as described above, except with T_1^{obs} as a fourth fitting parameter, and $\alpha(r + 1)$ as a fitting parameter instead of α. The fitting parameters and root mean square error (R) obtained for (the initially non-inverted) TBAT are presented in Table S5.11. The experimental and model magnetisation transfer profiles for each [FTPS] are presented in Figure S5.20 (expansion of the plot for $\tau \leq 0.05$ is presented on the right). The plot of $\alpha(r + 1)$ *vs* [FTPS] is presented in Figure S5.21. It shows that $\alpha(r + 1)$ initially decreased with increased [FTPS] (first-to-second and second-to-third measurements). Then, after reaching a minimum around the third measurement, $\alpha(r + 1)$ started increasing. Evidently, fluoride transfer from TBAT to FTPS is at least partially dissociative in character in the non-stabilised system.

Table S5.11: The fitting parameters and root mean square errors (*R*) for the magnetisation transfer between TBAT and FTPS.

[FTPS] (mM)	I_{eq}	x	$\alpha(r + 1)$ (s^{-1})	T_1^{obs} (s)	R
3.31	7681	191	234	1.31	15
11.9	7754	464	151	1.24	14
23.1	7782	756	142	1.27	16
32.1	7640	952	144	1.16	20
45.7	7487	1254	145	1.25	19
50.9	7496	1406	154	1.26	24
67.0	7451	1715	160	1.31	17

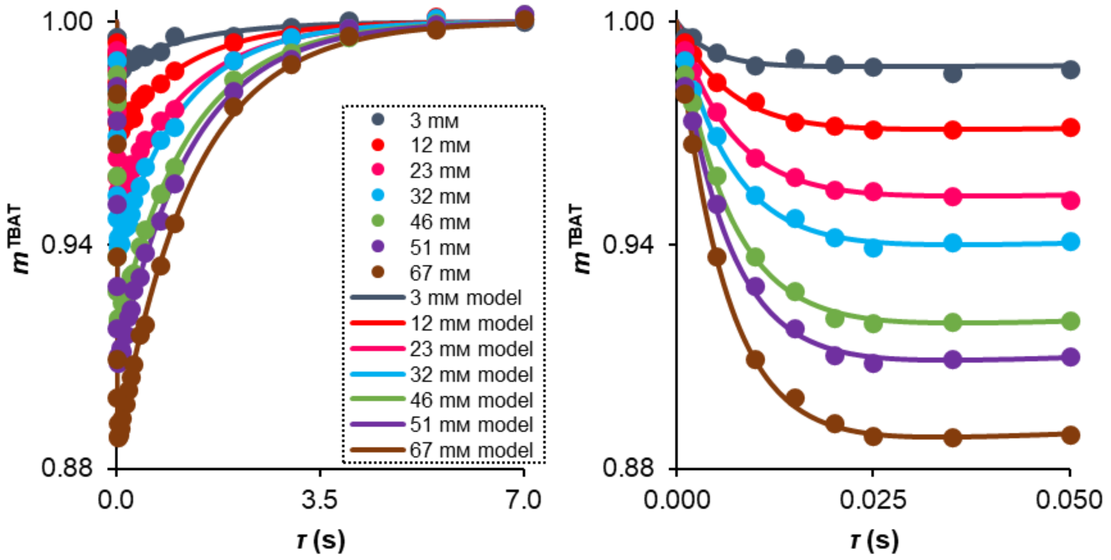

Figure S5.20: Magnetisation transfer profiles in titration of TBAT with FTPS.

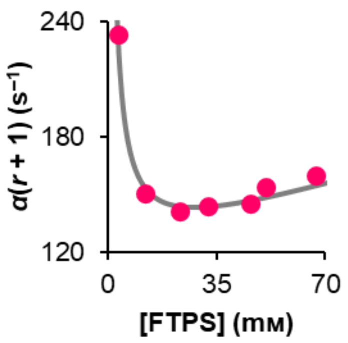

Figure S5.21: Proof of at least partially dissociative character of TBAT/FTPS fluoride transfer in the non-stabilised system in THF. (The grey line is not a fitted model but merely an aid to the eye).

S5.2.7. Exchange in MeCN

A solution was prepared in the glovebox by weighing TBAT (48.8 mM), FTPS (45.8 mM) and NAPH (322 mM) into a 1 mL volumetric flasks and dissolving them in MeCN up to the 1 mL mark. The solution was transferred to two J Young® valve capped NMR tubes (~0.5 mL, each), one of them preloaded with six 3 Å molecular sieve beads (3Å-MS, ~40 mg). DTBP (5.00 μL, 23.1 mM) was added to the NMR tube containing the molecular sieves, and the tube was shaken thoroughly. ^{19}F inversion-recovery and ^{19}F selective inversion-recovery experiments were performed at 300 K for each solution; 2 sets for the solution in the absence of base and molecular sieves and 6 sets for the solution in the presence of base and sieves. The variable delays used in the inversion-recovery experiment were, τ (s) {0.1, 0.3, 0.5, 1, 3, 5, 7.5, 10, 15, 20, 35, 50}. The variable delays used in the selective inversion-recovery experiment were, τ (s) {0.001, 0.002, 0.005, 0.01, 0.015, 0.02, 0.025, 0.035, 0.05, 0.075, 0.1, 0.15, 0.2, 0.25, 0.3, 0.4, 0.5, 1, 5, 10, 15, 20, 35, 50}. The date and time of acquisition of each set (more specifically, of the ^{19}F selective inversion-recovery experiment in a given set) were converted to the time interval between preparation of the sample and the acquisition, *t*.

The ^{19}F longitudinal relaxation fitting parameters (I_{eq}, x and T_1^{obs}) and root mean square errors (R) are presented in Table S5.12. ^{19}F longitudinal relaxation time constant of FTPS in MeCN (at 300 K), T_1^{FTPS} = 16.6 s, was found to be independent of its concentration and significantly larger than the one of TBAT (7.14 ≥ T_1^{TBAT} ≥ 4.46 in the 14.1-229 mM concentration range studied herein). Similarly to solutions of TBAT and FTPS in THF, an averaged longitudinal relaxation rate was observed for the spins in the mixture, T_1^{obs} = (8.37 ± 0.24) s (*cf*. the expected 8.07 s), due to very rapid exchange.

Table S5.12: Longitudinal relaxation fitting parameters in TBAT/FTPS system in MeCN.

Solution	*t* (h)	I_{eq}	*x*	T_1^{obs} (s)	*R*
Original	1.23	766	1.15	7.88	8
Original	3.23	660	1.11	7.84	13
Base + MS	2.25	712	1.20	8.73	3
Base + MS	4.27	703	1.20	8.28	4
Base + MS	6.28	717	1.20	8.48	3
Base + MS	8.33	642	1.24	8.56	4
Base + MS	10.4	639	1.23	8.64	5
Base + MS	12.4	617	1.22	8.58	6

The ^{19}F exchange fitting parameters, I_{eq}, *x*, *x*' and $\alpha(r + 1)$, and root mean square errors (*R*) obtained for (the initially non-inverted) TBAT are presented in Table S5.13 (except for the second measurement for the original solution).

Table S5.13: Evolution of magnetisation transfer rate in TBAT/FTPS system in MeCN.

Solution	*t* (h)	I_{eq}	*x*	*x*'	$\alpha(r + 1)$ (s^{-1})	*R*
Original	1.23	1102	396	588	604	23
Original	3.23	N/A	N/A	N/A	N/A	N/A
Base + MS	2.25	1155	702	753	149	10
Base + MS	4.27	1143	727	751	97.6	9
Base + MS	6.28	1161	719	759	91.9	11
Base + MS	8.33	1136	728	756	105	13
Base + MS	10.4	1129	694	754	133	10
Base + MS	12.4	1100	711	736	180	15

Indeed, very rapid exchange was observed between the spins in the magnetisation transfer experiment (original solution, t = 1.23 h), with a magnetisation transfer rate constant $\alpha(r + 1)$ = 604 s^{-1}. The exchange was found to be significantly more rapid than in THF ($\alpha^{MeCN}/\alpha^{THF} \approx$ 16 for the solutions described herein and in Section S5.2.2), and extremely rapid on the longitudinal relaxation timescale, as illustrated by Figure S5.22b (expansion of the plot from Figure S5.22a showing the first eight data points, τ < 40 ms). The initial decay of longitudinal magnetisation of TBAT reached an apparent plateau corresponding to a statistical distribution of the inverted ^{19}F spins between TBAT and FTPS, prior to pseudo-exponential relaxation of TBAT (and FTPS) with T_1^{obs} = 7.88 s. This statistical distribution was practically achieved before the third data point, τ = 5 ms (m^{TBAT} = 0.742 *cf*. the minimum m^{TBAT} = 0.734 for τ = 13.4 ms). The model fractional magnetisation of TBAT at τ = 0 was less than 1, m^{TBAT} = 0.913, due to non-negligible exchange between TBAT and FTPS during the selective inversion (the soft inversion pulse was ~1.3 ms long). At τ = 1 ms, m^{TBAT} decreased further to 0.831. Such rapid exchange between the spins rendered magnetisation transfer impractical for TBAT/FTPS systems in MeCN at 300 K. Although shorter variable delays could be used in the experiments, very significant broadening of both TBAT and FTPS signals was observed (as presented in Figure S5.22c), which resulted in noisier data (Figure S5.22a–b, compared to magnetisation transfer in THF). Repeating the magnetisation transfer measurement of the sample two hours

after the first one gave a magnetisation transfer profile presented in Figure S5.22d, whereby the statistical distribution of magnetisation was reached prior to the first measurement, where τ = 1 ms.

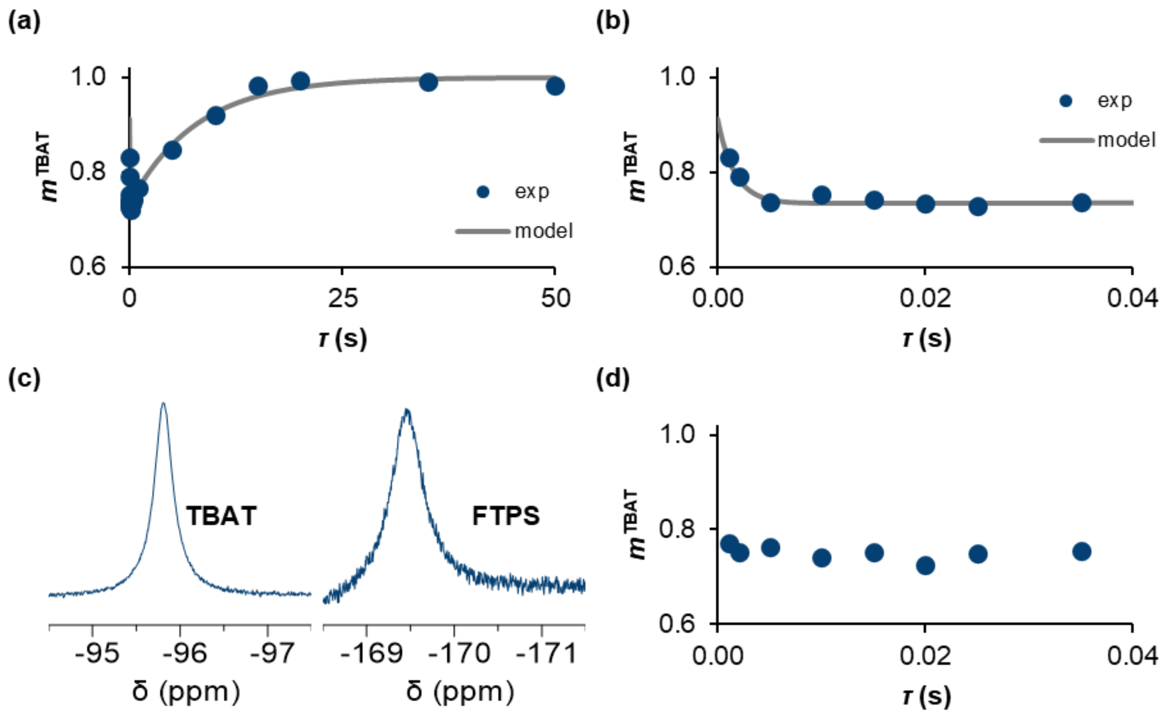

Figure S5.22: ^{19}F magnetisation transfer data from a solution of TBAT (48.8 mM) and FTPS (45.8 mM) in MeCN at 300 K. (a) A plot of experimental and model fractional magnetisation of TBAT, m^{TBAT}, against variable delay, τ, resulting from the selective inversion of FTPS. The chemical exchange between the spins is extremely rapid compared to their longitudinal relaxation, $\alpha T_1^{obs} \approx 1500$. (b) An expansion of the plot from (a) showing the first eight experimental data points (τ < 40 ms). (c) Both TBAT and FTPS exhibited a very significant line broadening in the ^{19}F NMR spectrum of the solution (the spectrum was phase- but not baseline-corrected). (d) After two hours, the transfer rate in the sample increased to an extent that a statistical distribution of the magnetisation was reached before the τ = 1 ms time point (the last data point, τ = 50 s was used as the equilibrium integral for conversion into m^{TBAT}, and the τ < 40 ms part of the plot is presented).

The magnetisation transfer plots for the solution in the presence of base and sieves are presented in Figure S5.23.

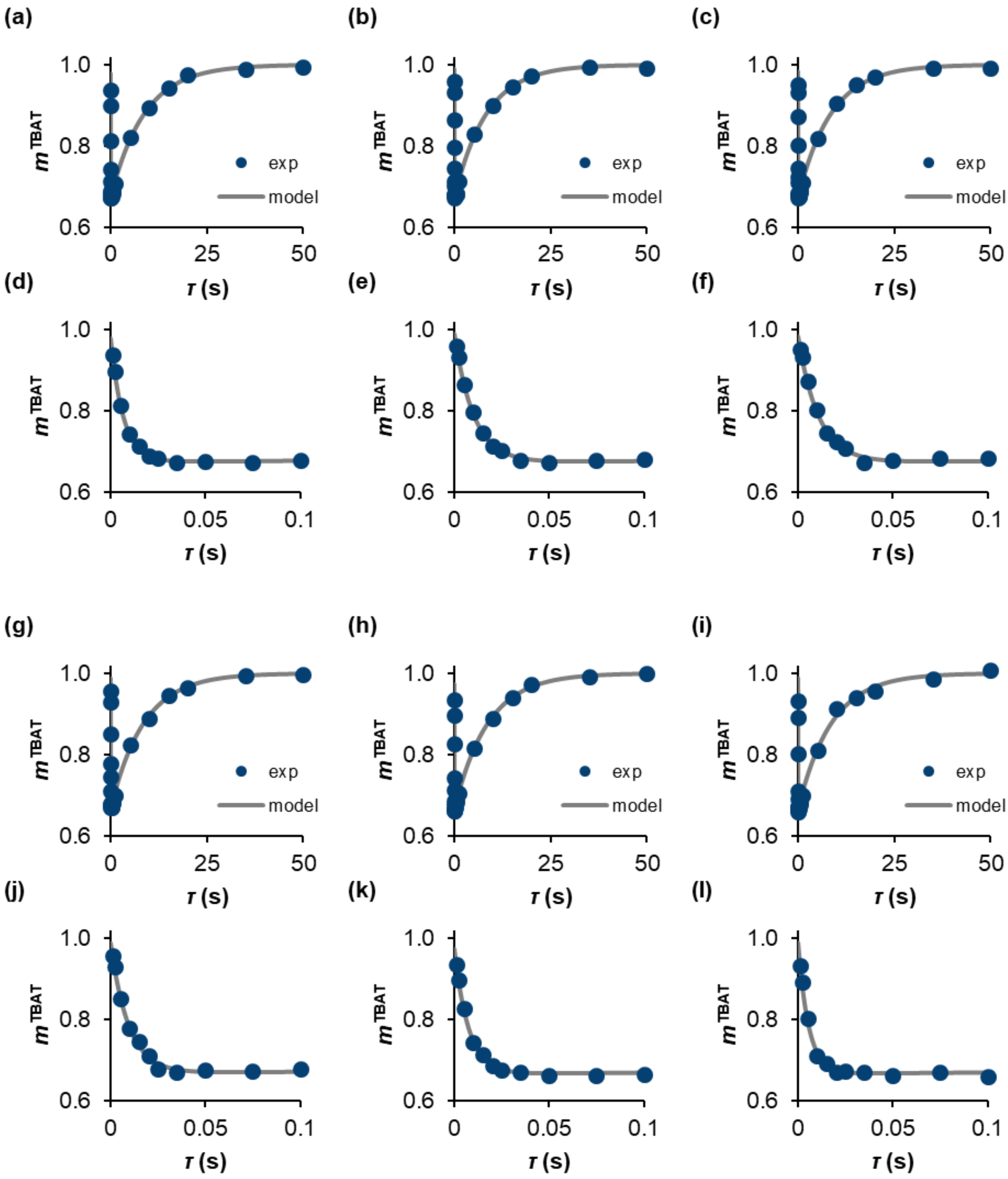

Figure S5.23: ^{19}F magnetisation transfer profiles of the initially non-inverted TBAT, and expansions of the plots to early variable delays, over time, for the solution in the presence of base and molecular sieves: (a and d) 2.25 h, (b and e) 4.27 h, (c and f) 6.28 h, (g and j) 8.33 h, (h and k) 10.4 h, (i and l) 12.4 h.

Based on the observations discussed in Section S5.2.5, we envisaged that in the presence of DTBP and 3Å-MS, the magnetisation transfer rate in the system would decrease over time, eventually reaching a plateau, analogous to systems in THF. Indeed, the first measurement showed a significant, four-fold decrease in the magnetisation transfer rate, $\alpha(r + 1) = 149\ s^{-1}$. This decrease was confirmed by the appearance of the signals, Figure S5.24a, which

exhibited less broadening (^{29}Si satellites were observed for both signals, *cf*. Figure S5.22c). An initial decrease of $\alpha(r + 1)$ was observed as expected. However, as opposed to plateauing, the exchange rate reached a minimum for $t \approx 7$ h prior to increasing with time again, Figure S5.24b.

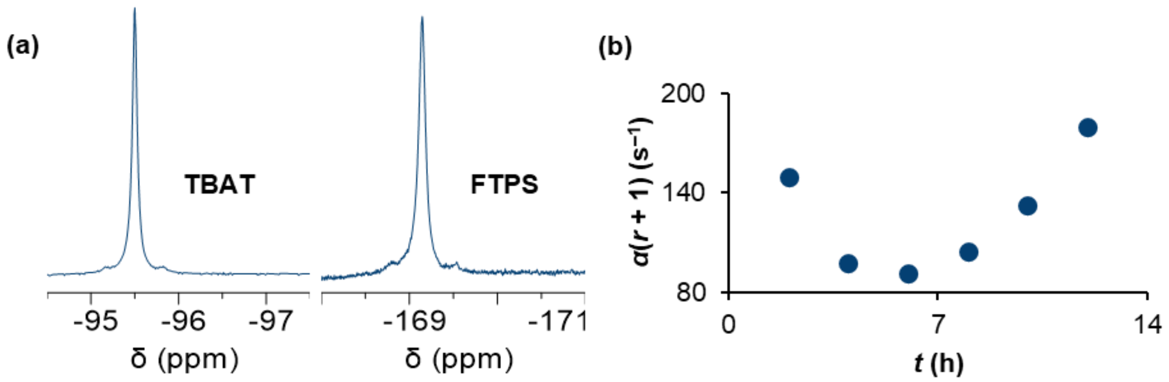

Figure S5.24: (a) Both TBAT and FTPS exhibited far less line broadening in the ^{19}F NMR spectrum of the solution in the presence of base and molecular sieves. (b) Temporal evolution of the magnetisation transfer between the spins. An initial decrease in the exchange rate was followed by a minimum and subsequent increase, as opposed to the expected plateau.

S5.3. TBAT/ARSF

S5.3.1. Kinetic model

Zheng *et al*. reported that aryl fluorosulfates undergo rapid fluorine exchange in the presence of several fluoride sources, including TBAT.[S14] They proposed that the rapid equilibrium exchange is associative with respect to the sulfur centre and that the equilibrium position of the fluoride addition is shifted towards the neutral ester. Therefore, the exchange can be approximated to concerted substitution. Hence, a commercially available aryl fluorosulfate, 2-naphthalenyl fluorosulfate (ARSF), was used as a second exchanging partner to TBAT in this study. The kinetic model for the TBAT/ARSF spin system, where ARSF is the electrophilic acceptor of the fluoride (Scheme S5.5a), is derived in an analogous way to the TBAT/FTPS one. We note that equilibrium dissociation of TBAT, produces low-concentration FTPS and TBAF. Hence, Equations S5.26–S5.28 are the starting point in the derivation of the model herein.

S5.26, recalled

$$\frac{dm^{\mathrm{TBAT}}}{d\tau} = \frac{1}{T_1^{\mathrm{TBAT}}}(1 - m^{\mathrm{TBAT}}) + \frac{k_{-1}[\mathrm{FTPS}][\mathrm{TBAF}]}{2[\mathrm{TBAT}]}(m^{\mathrm{FTPS}} + m^{\mathrm{TBAF}}) - k_1 m^{\mathrm{TBAT}} + \frac{k_2}{2}[\mathrm{FTPS}](m^{\mathrm{FTPS}} - m^{\mathrm{TBAT}})$$

S5.27, recalled

$$\frac{dm^{\mathrm{FTPS}}}{d\tau} = \frac{1}{T_1^{\mathrm{FTPS}}}(1 - m^{\mathrm{FTPS}}) + \frac{k_1[\mathrm{TBAT}]}{[\mathrm{FTPS}]} m^{\mathrm{TBAT}} - k_{-1}[\mathrm{TBAF}] m^{\mathrm{FTPS}} + k_2[\mathrm{TBAT}](m^{\mathrm{TBAT}} - m^{\mathrm{FTPS}})$$

S5.28, recalled

$$\frac{dm^{\mathrm{TBAF}}}{d\tau} = \frac{1}{T_1^{\mathrm{TBAF}}}(1 - m^{\mathrm{TBAF}}) + \frac{k_1[\mathrm{TBAT}]}{[\mathrm{TBAF}]} m^{\mathrm{TBAT}} - k_{-1}[\mathrm{FTPS}] m^{\mathrm{TBAF}}$$

ARSF is assumed to exchange the fluoride in an associative process, where an anionic ARSF' intermediate containing two equivalent fluorine atoms on the sulfur centre is formed with Δ*G*° >> 0 (Scheme S5.5a). The addition of ARSF to the solution of TBAT, therefore, results in two more exchanging species present in the system, ARSF and ARSF', the latter being a low-concentration intermediate. Since ARSF' is short-lived (and thus in low concentration), the exchange of the S–F fluoride may be approximated to a concerted process with respect to ARSF (Scheme S5.5b).

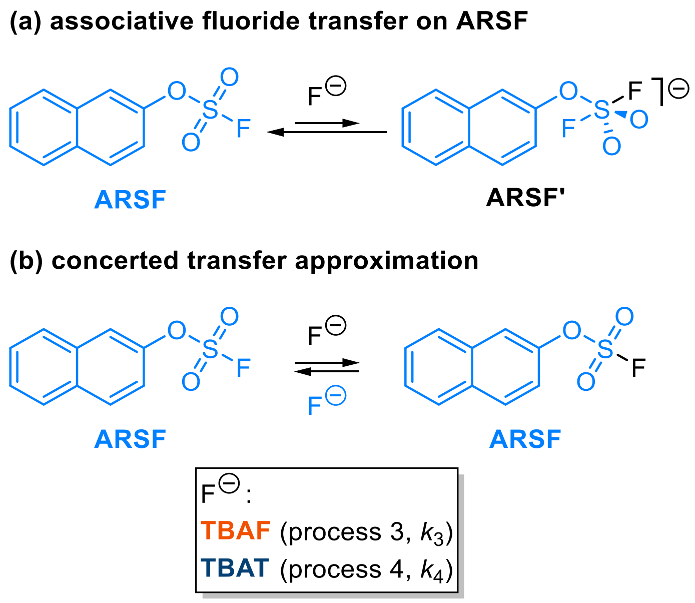

Scheme S5.5: (a) ARSF exchanges fluoride in an associative process *via* a pentacoordinate difluoro intermediate ARSF' (Δ*G*° >> 0). (b) Due to the intermediate being short-lived, it can be assumed in the derivation of the model that the fluoride exchange is concerted with respect to ARSF. In both cases, "F⁻" denotes a nucleophilic fluoride source, which may be TBAF and/or TBAT in the TBAT/ARSF system.

The fluoride that exchanges with ARSF in the system may be the low-concentration TBAF formed from dissociation of TBAT (Scheme S5.1a) and/or TBAT directly (Scheme S5.1b). These processes, described by the rate constants k_3 and k_4 (or k_3 and $k_4/2$ "per fluoride"), respectively, correspond to the dissociative and direct transfer pathways with respect to TBAT. These two pathways, accounting for the magnetisation states of TBAT, TBAF and ARSF, are presented in Scheme S5.6.

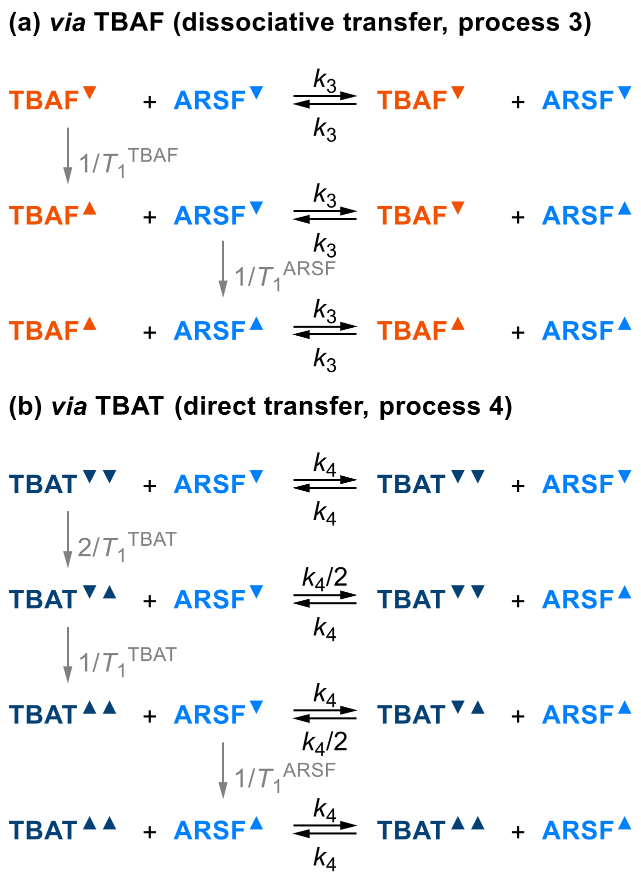

Scheme S5.6: The breakdown of the (a) dissociative and (b) direct transfer exchange pathways between TBAT and ARSF, accounting for their longitudinal magnetisation states. Note the rate constants of the chemically degenerate equilibrium transfer processes relative to the overall rate constant k_4 depending on the magnetisation states of the spins.

The rate laws arising from the exchange pathways in Scheme S5.6, and the relaxation of ARSF, are presented in Equations S5.69–S5.79 (the expression for fractional magnetisation of ARSF is analogous to those of FTPS and TBAF, and is given by Equation S5.68).

S5.68

$$m^{\mathrm{ARSF}} = \frac{[\mathrm{ARSF}^{\blacktriangle}]}{[\mathrm{ARSF}]} = \frac{[\mathrm{ARSF}^{\blacktriangle}]}{[\mathrm{ARSF}^{\blacktriangledown}] + [\mathrm{ARSF}^{\blacktriangle}]} = \frac{M_z^{\mathrm{ARSF}} + M_{z,\mathrm{eq}}^{\mathrm{ARSF}}}{2M_{z,\mathrm{eq}}^{\mathrm{ARSF}}}$$

Relaxation of ARSF:

S5.69

$$\left(\frac{d[\mathrm{ARSF}^{\blacktriangle}]}{d\tau}\right)_{T_1} = \frac{1}{T_1^{\mathrm{ARSF}}}[\mathrm{ARSF}^{\blacktriangledown}]$$

S5.70

$$\left(\frac{dm^{\mathrm{ARSF}}}{d\tau}\right)_{T_1} = \frac{1}{T_1^{\mathrm{ARSF}}}(1 - m^{\mathrm{ARSF}})$$

Dissociative transfer (process 3, "P3"):

S5.71

$$\left(\frac{d[\mathrm{TBAF}^{\blacktriangle}]}{d\tau}\right)_{\mathrm{P3}} = k_3[\mathrm{TBAF}^{\blacktriangledown}][\mathrm{ARSF}^{\blacktriangle}] - k_3[\mathrm{TBAF}^{\blacktriangle}][\mathrm{ARSF}^{\blacktriangledown}]$$

S5.72

$$\left(\frac{dm^{\mathrm{TBAF}}}{d\tau}\right)_{\mathrm{P3}} = k_3[\mathrm{ARSF}](m^{\mathrm{ARSF}} - m^{\mathrm{TBAF}})$$

S5.73

$$\left(\frac{d[\mathrm{ARSF}^{\blacktriangle}]}{d\tau}\right)_{\mathrm{P3}} = k_3[\mathrm{TBAF}^{\blacktriangle}][\mathrm{ARSF}^{\blacktriangledown}] - k_3[\mathrm{TBAF}^{\blacktriangledown}][\mathrm{ARSF}^{\blacktriangle}]$$

S5.74

$$\left(\frac{dm^{\mathrm{ARSF}}}{d\tau}\right)_{\mathrm{P3}} = k_3[\mathrm{TBAF}](m^{\mathrm{TBAF}} - m^{\mathrm{ARSF}})$$

Direct transfer (process 4, "P4"):

S5.75

$$\left(\frac{d[\mathrm{TBAT}^{\blacktriangledown\blacktriangle}]}{d\tau}\right)_{\mathrm{P4}} = k_4[\mathrm{TBAT}^{\blacktriangledown\blacktriangledown}][\mathrm{ARSF}^{\blacktriangle}] + k_4[\mathrm{TBAT}^{\blacktriangle\blacktriangle}][\mathrm{ARSF}^{\blacktriangledown}] - \frac{k_4}{2}[\mathrm{TBAT}^{\blacktriangledown\blacktriangle}][\mathrm{ARSF}^{\blacktriangledown}] - \frac{k_4}{2}[\mathrm{TBAT}^{\blacktriangledown\blacktriangle}][\mathrm{ARSF}^{\blacktriangle}]$$

S5.76

$$\left(\frac{d[\mathrm{TBAT}^{\blacktriangle\blacktriangle}]}{d\tau}\right)_{\mathrm{P4}} = \frac{k_4}{2}[\mathrm{TBAT}^{\blacktriangledown\blacktriangle}][\mathrm{ARSF}^{\blacktriangle}] - k_4[\mathrm{TBAT}^{\blacktriangle\blacktriangle}][\mathrm{ARSF}^{\blacktriangledown}]$$

S5.77

$$\left(\frac{dm^{\mathrm{TBAT}}}{d\tau}\right)_{\mathrm{P4}} = \frac{k_4}{2}[\mathrm{ARSF}](m^{\mathrm{ARSF}} - m^{\mathrm{TBAT}})$$

S5.78

$$\left(\frac{d[\mathrm{ARSF}^{\blacktriangle}]}{d\tau}\right)_{\mathrm{P4}} = \frac{k_4}{2}[\mathrm{ARSF}^{\blacktriangledown}]([\mathrm{TBAT}^{\blacktriangledown\blacktriangle}] + 2[\mathrm{TBAT}^{\blacktriangle\blacktriangle}]) - \frac{k_4}{2}[\mathrm{ARSF}^{\blacktriangle}](2[\mathrm{TBAT}^{\blacktriangledown\blacktriangledown}] + [\mathrm{TBAT}^{\blacktriangledown\blacktriangle}])$$

S5.79

$$\left(\frac{dm^{\mathrm{ARSF}}}{d\tau}\right)_{\mathrm{P4}} = k_4[\mathrm{TBAT}](m^{\mathrm{TBAT}} - m^{\mathrm{ARSF}})$$

Summing Equations S5.26 and S5.77 gives the rate law of the fractional magnetisation of TBAT; Equations S5.28 and S5.72 – of TBAF; and Equations S5.70, S5.74 and S5.79 – of ARSF. These three rate laws are presented in Equations S5.80–S5.82. The rate law of the fractional magnetisation of FTPS remains as in Equation S5.27.

S5.80

$$\frac{dm^{\mathrm{TBAT}}}{d\tau} = \frac{1}{T_1^{\mathrm{TBAT}}}(1 - m^{\mathrm{TBAT}}) + \frac{k_2}{2}[\mathrm{FTPS}](m^{\mathrm{FTPS}} - m^{\mathrm{TBAT}}) + \frac{k_1}{2}(m^{\mathrm{FTPS}} + m^{\mathrm{TBAF}} - 2m^{\mathrm{TBAT}}) + \frac{k_4}{2}[\mathrm{ARSF}](m^{\mathrm{ARSF}} - m^{\mathrm{TBAT}})$$

S5.27, recalled

$$\frac{dm^{\mathrm{FTPS}}}{d\tau} = \frac{1}{T_1^{\mathrm{FTPS}}}(1 - m^{\mathrm{FTPS}}) + \frac{k_1[\mathrm{TBAT}]}{[\mathrm{FTPS}]}m^{\mathrm{TBAT}} - k_{-1}[\mathrm{TBAF}]m^{\mathrm{FTPS}} + k_2[\mathrm{TBAT}](m^{\mathrm{TBAT}} - m^{\mathrm{FTPS}})$$

S5.81

$$\frac{dm^{\mathrm{TBAF}}}{d\tau} = \frac{1}{T_1^{\mathrm{TBAF}}}(1 - m^{\mathrm{TBAF}}) + \frac{k_1[\mathrm{TBAT}]}{[\mathrm{TBAF}]}m^{\mathrm{TBAT}} - k_{-1}[\mathrm{FTPS}]m^{\mathrm{TBAF}} + k_3[\mathrm{ARSF}](m^{\mathrm{ARSF}} - m^{\mathrm{TBAF}})$$

S5.82

$$\frac{dm^{\mathrm{ARSF}}}{d\tau} = \frac{1}{T_1^{\mathrm{ARSF}}}(1 - m^{\mathrm{ARSF}}) + k_3[\mathrm{TBAF}](m^{\mathrm{TBAF}} - m^{\mathrm{ARSF}}) + k_4[\mathrm{TBAT}](m^{\mathrm{TBAT}} - m^{\mathrm{ARSF}})$$

Here, both FTPS and TBAF are low-concentration intermediates and hence the steady state approximation of both can be applied (ignoring the relaxation of FTPS and TBAF, and noting the expression for the equilibrium constant of process 1, Equation S5.31).

FTPS:

S5.83

$$\frac{dm^{\mathrm{FTPS}}}{d\tau} = \frac{1}{T_1^{\mathrm{FTPS}}}(1 - m^{\mathrm{FTPS}}) + \frac{k_1[\mathrm{TBAT}]}{[\mathrm{FTPS}]}m^{\mathrm{TBAT}} - k_{-1}[\mathrm{TBAF}]m^{\mathrm{FTPS}} + k_2[\mathrm{TBAT}](m^{\mathrm{TBAT}} - m^{\mathrm{FTPS}}) \approx 0$$

S5.84

$$m^{\mathrm{FTPS}} \approx m^{\mathrm{TBAT}}$$

TBAF:

S5.85

$$\frac{dm^{\mathrm{TBAF}}}{d\tau} = \frac{1}{T_1^{\mathrm{TBAF}}}(1 - m^{\mathrm{TBAF}}) + \frac{k_1[\mathrm{TBAT}]}{[\mathrm{TBAF}]}m^{\mathrm{TBAT}} - k_{-1}[\mathrm{FTPS}]m^{\mathrm{TBAF}} + k_3[\mathrm{ARSF}](m^{\mathrm{ARSF}} - m^{\mathrm{TBAF}}) \approx 0$$

S5.86

$$m^{\mathrm{TBAF}} \approx \frac{k_{-1}[\mathrm{FTPS}]m^{\mathrm{TBAT}} + k_3[\mathrm{ARSF}]m^{\mathrm{ARSF}}}{k_{-1}[\mathrm{FTPS}] + k_3[\mathrm{ARSF}]}$$

Inserting Equation S5.84 into Equation S5.80 “cancels” the effect of process 2 (direct transfer between TBAT and FTPS) on the magnetisation transfer in the TBAT/ARSF system:

S5.87

$$\frac{dm^{\mathrm{TBAT}}}{d\tau} = \frac{1}{T_1^{\mathrm{TBAT}}}(1 - m^{\mathrm{TBAT}}) + \frac{k_1}{2}(m^{\mathrm{TBAF}} - m^{\mathrm{TBAT}}) + \frac{k_4}{2}[\mathrm{ARSF}](m^{\mathrm{ARSF}} - m^{\mathrm{TBAT}})$$

Inserting Equation S5.86 into Equations S5.87 and S5.82, and rearranging:

S5.88

$$\frac{dm^{\mathrm{TBAT}}}{d\tau} = \frac{1}{T_1^{\mathrm{TBAT}}}(1 - m^{\mathrm{TBAT}}) + \left(\frac{k_1 k_3[\mathrm{ARSF}]}{2k_{-1}[\mathrm{FTPS}] + 2k_3[\mathrm{ARSF}]} + \frac{k_4}{2}[\mathrm{ARSF}]\right)(m^{\mathrm{ARSF}} - m^{\mathrm{TBAT}})$$

S5.89

$$\frac{dm^{\mathrm{ARSF}}}{d\tau} = \frac{1}{T_1^{\mathrm{ARSF}}}(1 - m^{\mathrm{ARSF}}) + \left(\frac{k_{-1} k_3[\mathrm{FTPS}][\mathrm{TBAF}]}{k_{-1}[\mathrm{FTPS}] + k_3[\mathrm{ARSF}]} + k_4[\mathrm{TBAT}]\right)(m^{\mathrm{TBAT}} - m^{\mathrm{ARSF}})$$

Assuming that k_{-1}[FTPS] >> k_3[ARSF] and noting the expression for the equilibrium constant of process 1 (Equation S5.31):

S5.90

$$\frac{dm^{\mathrm{TBAT}}}{d\tau} = \frac{1}{T_1^{\mathrm{TBAT}}}(1 - m^{\mathrm{TBAT}}) + [\mathrm{ARSF}]\left(\frac{K_1 k_3}{2[\mathrm{FTPS}]} + \frac{k_4}{2}\right)(m^{\mathrm{ARSF}} - m^{\mathrm{TBAT}})$$
$$= \frac{1}{T_1^{\mathrm{TBAT}}}(1 - m^{\mathrm{TBAT}}) + \alpha(m^{\mathrm{ARSF}} - m^{\mathrm{TBAT}})$$

S5.91

$$\frac{dm^{\mathrm{ARSF}}}{d\tau} = \frac{1}{T_1^{\mathrm{ARSF}}}(1 - m^{\mathrm{ARSF}}) + [\mathrm{TBAT}]\left(\frac{K_1 k_3}{[\mathrm{FTPS}]} + k_4\right)(m^{\mathrm{TBAT}} - m^{\mathrm{ARSF}})$$
$$= \frac{1}{T_1^{\mathrm{ARSF}}}(1 - m^{\mathrm{ARSF}}) + r\alpha(m^{\mathrm{TBAT}} - m^{\mathrm{ARSF}})$$

Where:

S5.92

$$\alpha = [\mathrm{ARSF}]\left(\frac{K_1 k_3}{2[\mathrm{FTPS}]} + \frac{k_4}{2}\right)$$

S5.93

$$r = \frac{M_{z,\mathrm{eq}}^{\mathrm{TBAT}}}{M_{z,\mathrm{eq}}^{\mathrm{ARSF}}} = \frac{2[\mathrm{TBAT}]}{[\mathrm{ARSF}]}$$

The expression for α describes the rate of magnetisation transfer between TBAT and ARSF as a function of the appropriate equilibrium and rate constants, as well as concentrations of ARSF and FTPS (in the regime of steady-state magnetisation of FTPS). In the absence of added FTPS, the sole source of FTPS is dissociation of TBAT.

Equations S5.90 and S5.91 are Equivalent to Equations S5.37 and S5.38, and hence can be integrated analogously. The overall integrated rate laws of the fractional magnetisation of TBAT and ARSF are therefore given by Equations S5.43, S5.92, S5.93 and S5.94–S5.100.

S5.43, recalled

$$m^{\mathrm{TBAT}} = c_1 e^{(\beta+\gamma)\tau} + c_2 e^{(\beta-\gamma)\tau} + 1$$

S5.94

$$m^{\mathrm{ARSF}} = c_3 e^{(\beta+\gamma)\tau} + c_4 e^{(\beta-\gamma)\tau} + 1$$

Where:

S5.95

$$\beta = -\frac{\alpha(r+1) + \frac{1}{T_1^{\mathrm{TBAT}}} + \frac{1}{T_1^{\mathrm{ARSF}}}}{2}$$

S5.96

$$\gamma = \frac{\sqrt{\left(\alpha + \frac{1}{T_1^{\mathrm{TBAT}}} - r\alpha - \frac{1}{T_1^{\mathrm{ARSF}}}\right)^2 + 4r\alpha^2}}{2}$$

S5.97

$$c_1 = \frac{\alpha\left(m_0^{\mathrm{ARSF}} - m_0^{\mathrm{TBAT}}\right) + \left(\beta - \gamma + \frac{1}{T_1^{\mathrm{TBAT}}}\right)\left(1 - m_0^{\mathrm{TBAT}}\right)}{2\gamma}$$

S5.98

$$c_2 = -\frac{\alpha\left(m_0^{\mathrm{ARSF}} - m_0^{\mathrm{TBAT}}\right) + \left(\beta + \gamma + \frac{1}{T_1^{\mathrm{TBAT}}}\right)\left(1 - m_0^{\mathrm{TBAT}}\right)}{2\gamma}$$

S5.99

$$c_3 = \frac{r\alpha\left(m_0^{\mathrm{TBAT}} - m_0^{\mathrm{ARSF}}\right) + \left(\beta - \gamma + \frac{1}{T_1^{\mathrm{ARSF}}}\right)\left(1 - m_0^{\mathrm{ARSF}}\right)}{2\gamma}$$

S5.100

$$c_4 = -\frac{r\alpha\left(m_0^{\mathrm{TBAT}} - m_0^{\mathrm{ARSF}}\right) + \left(\beta + \gamma + \frac{1}{T_1^{\mathrm{ARSF}}}\right)\left(1 - m_0^{\mathrm{ARSF}}\right)}{2\gamma}$$

S5.92, recalled

$$\alpha = [\mathrm{ARSF}]\left(\frac{K_1 k_3}{2[\mathrm{FTPS}]} + \frac{k_4}{2}\right)$$

S5.93, recalled

$$r = \frac{M_{z,\mathrm{eq}}^{\mathrm{TBAT}}}{M_{z,\mathrm{eq}}^{\mathrm{ARSF}}} = \frac{2[\mathrm{TBAT}]}{[\mathrm{ARSF}]}$$

Magnetisation transfer between TBAT and ARSF is slow with respect to the longitudinal relaxation times of these spins (*cf*. the TBAT/FTPS exchange); as shown in Sections S5.3.2–S5.3.4. The magnitude of exchange and relaxation rates in TBAT/ARSF systems enables the approximation, whereby the extent of both these processes over the duration of the soft pulse are negligible, and hence the fractional magnetisation of the (initially) non-inverted spin at $\tau = 0$ is 1. Accounting for any imperfections in the selective π pulse, the fractional magnetisation

of the inverted spin at $\tau = 0$ may be >0. For selective inversion of TBAT and detection of ARSF, the constants c_3 and c_4 then take the forms:

S5.101

$$c_3 \approx -\frac{r\alpha(1 - m_0^{\mathrm{TBAT}})}{2\gamma}$$

S5.102

$$c_4 \approx \frac{r\alpha(1 - m_0^{\mathrm{TBAT}})}{2\gamma}$$

With this approximation, Equation S5.103 describes the system where TBAT is selectively inverted and (the initially non-inverted) ARSF is observed. The auxiliary terms α, β and γ are given by Equations S5.92, S5.95 and S5.96.

S5.103

$$m^{\mathrm{ARSF}} \approx \frac{r\alpha(1 - m_0^{\mathrm{TBAT}})}{2\gamma}\left[e^{(\beta-\gamma)\tau} - e^{(\beta+\gamma)\tau}\right] + 1$$

Equation S5.103 can be expressed as $M_z^{\mathrm{ARSF}}(\tau)$ according to Equation S5.3:

S5.104

$$M_z^{\mathrm{ARSF}} \approx \frac{r\alpha M_{\mathrm{eq}}^{\mathrm{ARSF}}(1 - m_0^{\mathrm{TBAT}})}{\gamma}\left[e^{(\beta-\gamma)\tau} - e^{(\beta+\gamma)\tau}\right] + M_{z,\mathrm{eq}}^{\mathrm{ARSF}}$$

Therefore, in TBAT/ARSF system, to the experimental integrals of ARSF at each variable delay was fitted a three-parameter ($M_{\mathrm{eq}}^{\mathrm{ARSF}}$, m_0^{TBAT}, α) model of Equation S5.104. The parameters r, T_1^{TBAT} and T_1^{ARSF} were determined from ^{19}F pulse-acquire and ^{19}F inversion-recovery experiments. The experimental and model integrals were then converted into fractional magnetisations (Equation S5.3).

S5.3.2. ^{19}F selective inversion-recovery in THF

A solution was prepared in the glovebox by weighing TBAT (198 mM), ARSF (58.6 mM) and NAPH (227 mM) into a 1 mL volumetric flask and dissolving them in THF up to the 1 mL mark. The solution (~0.4 mL) was transferred to a J Young® valve capped NMR tube. ^{19}F pulse-acquire, ^{19}F inversion-recovery and ^{19}F selective inversion-recovery experiments were run at 300 K, 310 K and 320 K. The NMR tube was open to air for ~30 s, closed, shaken thoroughly and the same set of experiments was run at 320 K. Then, two aliquots of a solution of water in THF were added to the sample under air (~0.2 mL of water was dissolved in THF up to 2 mL total volume under air; 2 µL and 5 µL of the resulting solution were added to the NMR tube *via* a microsyringe, corresponding to ~30 and 100 mM total added water, respectively). After each aliquot addition, the tube was shaken thoroughly, and the same set of experiments was run at 320 K. The variable delays used in the inversion-recovery experiments were, τ (s) {0.5,

1, 1.5, 2, 3, 4, 5, 7.5, 10, 12.5, 15, 20}. The variable delays used in the selective inversion-recovery experiments were, τ (s) {0.01, 0.02, 0.05, 0.1, 0.2, 0.3, 0.5, 0.75, 1, 1.5, 2, 3, 5, 10, 15, 20}.

The concentrations of TBAT and ARSF based on the internal standard, as well as the determined ^{19}F longitudinal relaxation fitting parameters (I_{eq}, x and T_1) and root mean square errors (R) for TBAT and ARSF are presented in Table S5.14a and Table S5.14b (for TBAT and ARSF, respectively).

Table S5.14: Concentrations (based on the internal standard, NAPH), longitudinal relaxation fitting parameters and root mean square errors (from ^{19}F inversion-recovery experiments) for (a) TBAT and (b) ARSF in the solution of TBAT and ARSF in THF.

(a)

Conditions	[TBAT] (mM)	I_{eq}^{TBAT}	x^{TBAT}	T_1^{TBAT} (s)	R^{TBAT}
300 K	197	11493	0.557	1.22	19
310 K	196	10157	0.549	1.42	11
320 K	195	9523	0.559	1.60	7
320 K, aired	192	6483	0.548	1.56	19
320 K, 30 mM water	174	5939	0.605	1.42	67
320 K, 100 mM water	152	4712	0.564	1.50	115

(b)

Conditions	[ARSF] (mM)	I_{eq}^{ARSF}	x^{ARSF}	T_1^{ARSF} (s)	R^{ARSF}
300 K	55.1	1616	0.574	1.74	5
310 K	53.8	1393	0.554	1.85	4
320 K	53.3	1307	0.531	2.01	5
320 K, aired	52.4	897	0.513	2.05	2
320 K, 30 mM water	54.2	1038	0.569	1.94	2
320 K, 100 mM water	52.8	971	0.562	1.93	2

The plots of experimental fractional magnetisations of ARSF, m^{ARSF}, against variable delay, τ, in ^{19}F selective inversion-recovery experiments are presented in Figure S5.25, as well as in Figure S5.26 (individual plots for each experiment for $\tau \leq 0.5$ s; all experimental data for $\tau \leq 0.5$ s are within the m^{ARSF} range shown in the plots). The equilibrium integrals, I_{eq}^{ARSF}, were determined by taking an average of the experimental integrals for all 16 variable delays in a given magnetisation transfer experiment in each plot in Figure S5.26. As evident from these plots, no exchange was detected between the spins at 300 K, 310 K and 320 K. Neither opening the system to air, nor the addition of two aliquots of water (~30 mM and ~100 mM total [H_2O], respectively), resulted in detection of any magnetisation transfer in the TBAT/ARSF system in THF (both these operations were found to catalyse TBAT/FTPS exchange in THF).

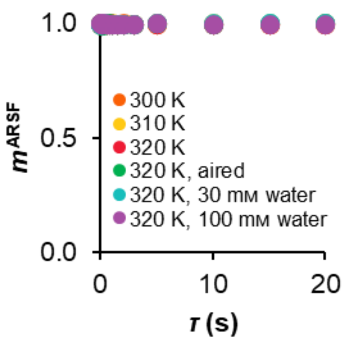

Figure S5.25: Magnetisation transfer profiles in TBAT/ARSF system in THF. The individual plots expanded to $\tau \leq 0.5$ s are presented in Figure S5.26.

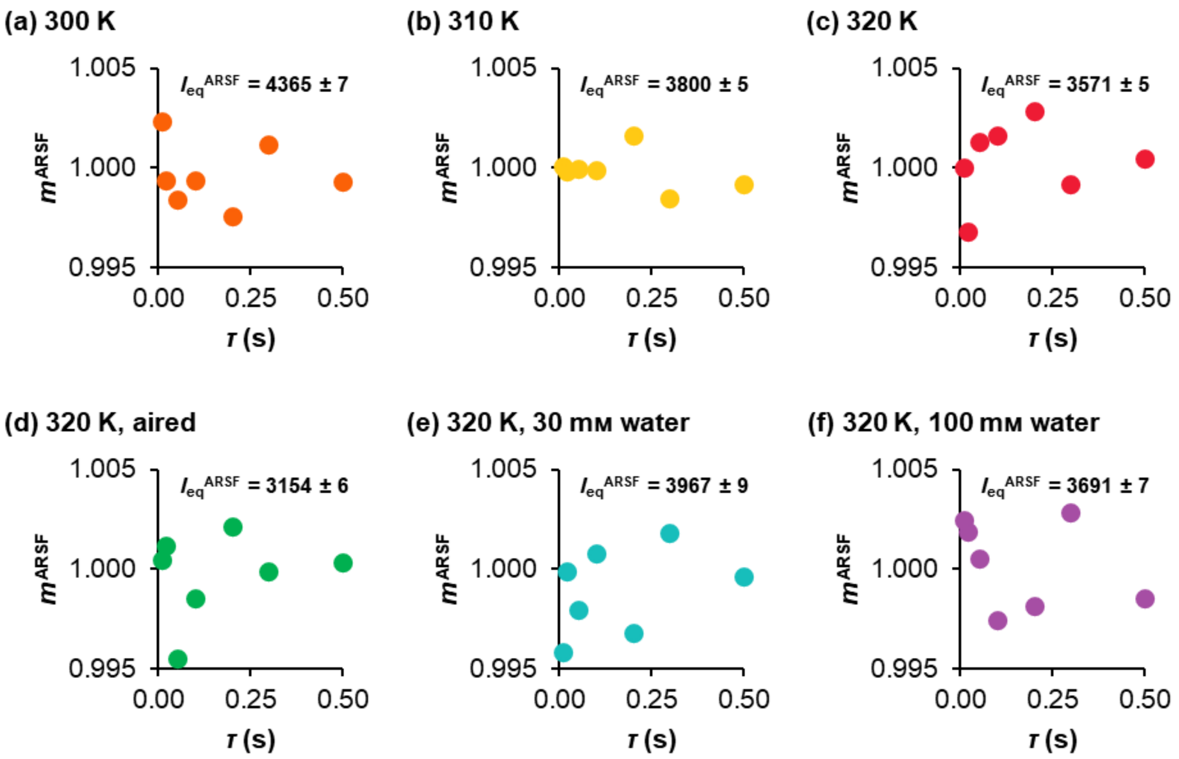

Figure S5.26: Plots of experimental fractional magnetisations of ARSF, m^{ARSF} *vs* τ (for $\tau \leq 0.5$ s) in ^{19}F selective inversion-recovery experiments between TBAT and ARSF in THF. I_{eq}^{ARSF}, used to convert experimental integrals in each experiment to the corresponding fractional magnetisations, are their averages (and given in the plots).

S5.3.3. Selective inversion-recovery in MeCN and inhibition by exogenous FTPS

A solution was prepared in the glovebox by weighing TBAT (191 mм), ARSF (56.9 mм) and NAPH (184 mм) into a 1 mL volumetric flask and dissolving them in MeCN up to the 1 mL mark. The solution (~0.4 mL) was transferred to a J Young® valve capped NMR tube. Four sets of ^{19}F pulse-acquire, ^{19}F inversion-recovery and ^{19}F selective inversion-recovery experiments were run at 300 K and within ~3 hours from sample preparation (except ^{19}F inversion-recovery, which was run only in the first set of experiments). A small solid aliquot of

FTPS was then added to the solution (~0.5 mg, corresponding to ~4 mM FTPS), and the NMR tube shaken thoroughly. The same four sets of NMR experiments were repeated (except for ^{19}F inversion-recovery), again over ~3 hours. The variable delays used in the inversion-recovery experiments were, τ (s) {1, 1.5, 2, 3, 4, 5, 7.5, 10, 12.5, 15, 20, 30}. The variable delays used in the selective inversion-recovery experiments were, τ (s) {0.001, 0.005, 0.01, 0.02, 0.05, 0.1, 0.2, 0.5, 1, 2, 3, 5, 10, 15, 20, 30}.

The concentrations of TBAT and ARSF based on NAPH are presented in Table S5.15. Based on this data, [ARSF] = (52.3 ± 0.1) mM for each experimental dataset (92% of the theoretical value based on the mass of the solute). The concentration of TBAT, however, seems vastly different before and after the addition of FTPS, [TBAT] = (188 ± 1) mM and (173 ± 2) mM, respectively (99% and 91% of the theoretical value). However, as presented in Figure S5.27a, the presence of even such small amount of FTPS (~4 mM, *cf*. ~190 mM TBAT) caused significant line broadening in TBAT (whilst the ARSF spin remained apparently unaffected by addition of FTPS). The FTPS signal was broadened to below the detection limit (Figure S5.27b). Therefore, the average of four concentrations of TBAT from before the spiking, [TBAT] = (188 ± 1) mM was used for all experimental datasets.

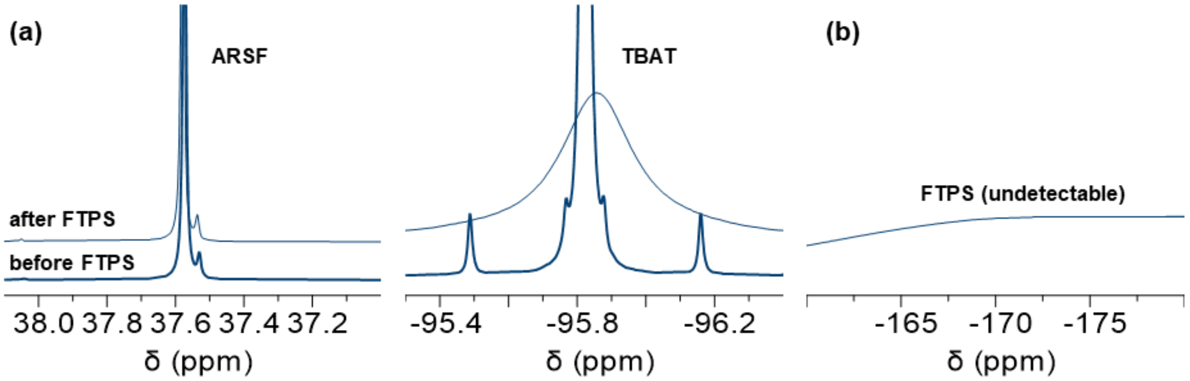

Figure S5.27: TBAT/ARSF system in MeCN prior to addition of exogenous FTPS (bottom spectra) and after addition of exogenous FTPS (top spectra). (a) The ^{19}F NMR signal of ARSF did not exhibit line broadening upon addition of FTPS unlike that of TBAT. (b) The FTPS signal was too broad to detect.

The determined ^{19}F longitudinal relaxation fitting parameters and root mean square errors (I_{eq}, x, T_1, R) for TBAT and ARSF were (13633, 0.625, 4.73 s, 5) and (1896, 0.567, 2.33 s, 5), respectively.

The magnetisation transfer model was fitted to the four dataset from prior to the addition of FTPS, as described above, except that two fitting parameters, I_{eq} and α, were independent for each dataset, whereas the third parameter, m_0^{TBAT}, was assumed to be equal in all of them (*i.e.* the sum of 64 squares was minimised by changing 9 variable parameters, all in one fitting). The fitting parameters I_{eq} and α obtained for (the initially non-inverted) ARSF, are presented

in Table S5.15. The "mutual" fitting parameter, m_0^{TBAT} = 0.0768, and the overall root mean square error, R = 5 (individual ones are given in the table). Then, fitting was performed analogously for the four datasets obtained after spiking the sample with FTPS, except m_0^{TBAT} was fixed as 0.0768 (the value obtained in the fitting of the four datasets above). This modification to the fitting procedure (as opposed to fitting each dataset individually) was applied due to the magnetisation transfer being much slower than relaxation. Because of this discrepancy in the rates of both processes, the absolute values of α and m_0^{TBAT} fitting parameters were prone to significant errors, but the relative changes in α between datasets were relatively independent of m_0^{TBAT} used/returned in the fitting. For example, the starting values in the fitting used herein were: the last measured integral of ARSF (τ = 30 s) as I_{eq} in the given dataset, m_0^{TBAT} = 0.07 (approximately the value of m_0^{FTPS} in Section S5.2.2), α (s^{-1}) {0.002, 0.0015, 0.0015, 0.0015} (as these values gave good visual fits). In this case, the returned m_0^{TBAT} was very close to the starting value, and so were α for each dataset. The determined α decreased by 24.4% between the first and second run, and remained constant throughout runs 2–4. When an arbitrary m_0^{TBAT} = 0.5 was used as a starting value (*i.e.* saturation of TBAT as opposed to inversion), with all the remaining starting parameters unchanged, the returned m_0^{TBAT} = 0.253 and the newly determined $\alpha = 2.37\cdot10^{-3}$ s^{-1} for the first run was 24.1% larger than previously, with nearly unchanged visual fit and R = 5. However, the relative decrease between the first and second run was nearly identical as previously, at 24.7%.

The plots of experimental and model (for the runs in the absence of FTPS only) fractional magnetisations of ARSF, m^{ARSF}, against variable delay, τ, are presented in Figure S5.28.

Table S5.15: Concentrations of TBAT and ARSF (based on the internal standard, NAPH), magnetisation transfer fitting parameters and root mean square errors (from ^{19}F selective inversion-recovery experiments) for the solution of TBAT and ARSF in MeCN.

Conditions	[TBAT] (mM)	[ARSF] (mM)	I_{eq}	α (s^{-1})	R
Run 1	190	52.2	3183	$1.91\cdot10^{-3}$	4
Run 2	188	52.3	3214	$1.44\cdot10^{-3}$	7
Run 3	188	52.4	3217	$1.44\cdot10^{-3}$	5
Run 4	187	52.4	3216	$1.46\cdot10^{-3}$	5
FTPS, run 1	176	52.3	3170	0 (exactly)	5
FTPS, run 2	172	52.2	3172	0 (exactly)	10
FTPS, run 3	173	52.4	3183	$1.01\cdot10^{-4}$	9
FTPS, run 4	170	52.4	3185	$2.23\cdot10^{-4}$	8

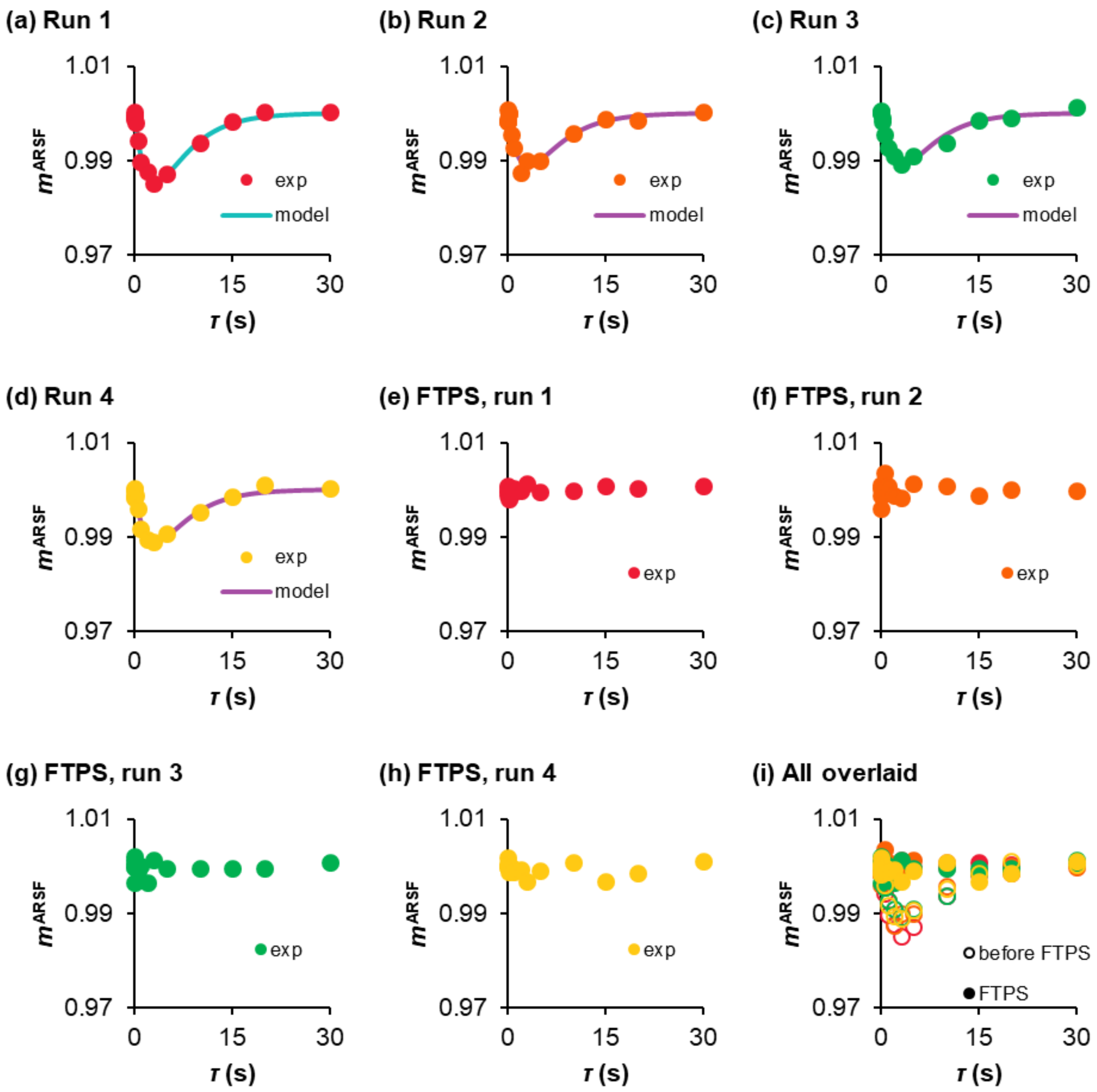

Figure S5.28: Plots of experimental (and model, where applicable) fractional magnetisations of ARSF, m^{ARSF} *vs* τ in ^{19}F selective inversion-recovery experiments between TBAT and ARSF in MeCN in the absence (a–d) and presence (e–h) of added FTPS.

The detected magnetisation transfer between the spins (TBAT/ARSF in MeCN, 300 K) is significantly slower than the exchange between TBAT and FTPS in THF or in MeCN. Across the four experiments in the absence of exogenous FTPS, the fractional magnetisation of ARSF only decreased to 97–98% of its equilibrium magnetisation, prior to subsequent recovery. The longitudinal relaxation time constants determined for TBAT and ARSF in the solution *via* ^{19}F non-selective inversion-recovery measurements (4.73 s and 2.33 s, respectively) were unequal and in excellent agreement with the corresponding constants for isolated spins under these conditions (4.77 s and 2.49 s, respectively). Both these observations confirmed that the exchange between TBAT and ARSF is very slow on the timescale of longitudinal relaxation of

these spins at 300 K in MeCN. The rate of exchange decreased slightly between the first and second measurement (a 24% decrease of α from $1.91 \cdot 10^{-3}$ s^{-1} to $1.44 \cdot 10^{-3}$ s^{-1}), and then remained constant across runs 2–4 (average $\alpha = 1.45 \cdot 10^{-3}$ s^{-1}).

As derived above, α in the TBAT/ARSF system should be a decreasing function of [FTPS] for the dissociative pathway, and independent of [FTPS] for the direct transfer pathway. The magnetisation transfer between TBAT and ARSF was inhibited by the addition of FTPS to below the detection limit. The extent of the inhibition, and thus the contributions of the dissociative and direct transfer pathways to the overall fluoride exchange between TBAT and ARSF in the solution, were estimated. Firstly, the average (stabilised) $\alpha = 1.4 \cdot 10^{-3}$ s^{-1} from runs 2–4 prior to the inhibition was used as the observed exchange rate constant in the system without added FTPS. Secondly, although qualitatively no exchange was observed in the FTPS-inhibited solution, the model was fitted to the four datasets. For the first two runs, the fittings gave $\alpha = 0$ (exactly), and the third and fourth run gave $\alpha = 1.01 \cdot 10^{-4}$ s^{-1} and $\alpha = 2.23 \cdot 10^{-4}$ s^{-1}, respectively; the average of these four values is $\alpha = 8.1 \cdot 10^{-5}$ s^{-1}. Thus, an estimated standard state partitioning for ARSF, $k_4(c_{\mathrm{TBAT}}°)^{0.5}/(K_1)^{0.5}k_3 < 6.1 \cdot 10^{-2}$.

S5.3.4. Temporal profile

A solution was prepared in the glovebox by weighing TBAT (204 mM), ARSF (73.3 mM) and NAPH (370 mM) into a 1 mL volumetric flask and dissolving them in MeCN up to the 1 mL mark. The solution was transferred to a J Young® valve capped NMR tube. The date and time of preparation of the solution was also noted. The solution was immediately inserted into the NMR probe preheated to 335 K and remained there for the duration of the experiment. Eleven sets of ^{19}F pulse-acquire, ^{19}F inversion-recovery and ^{19}F selective inversion-recovery experiments were performed at 335 K. The variable delays used in the inversion-recovery experiments were, τ (s) {1, 1.5, 2, 3, 4, 5, 7.5, 10, 12.5, 15, 20, 30}. The variable delays used in the selective inversion-recovery experiments were, τ (s) {0.01, 0.02, 0.05, 0.1, 0.2, 0.3, 0.5, 0.75, 1, 1.5, 2, 3, 5, 10, 20, 30}.

The date and time of each acquisition was converted to the time interval between preparation of the sample and the acquisition, t.

The concentrations of TBAT and ARSF based on NAPH are presented in Table S5.16. Both [ARSF] and [TBAT] decreased over time. The plot of [ARSF] against t is given in Figure S5.29. A first-order decomposition model, $[\mathrm{ARSF}] = [\mathrm{ARSF}]_\infty + ([\mathrm{ARSF}]_0 - [\mathrm{ARSF}]_\infty)e^{-kt}$ gave an excellent fit with the following fitting parameters and root mean square error (R): $[\mathrm{ARSF}]_\infty$ = 58.5 mM, $[\mathrm{ARSF}]_0$ = 67.9 mM, k = 0.275 h^{-1}, R = 0.3 mM. This models was used to calculate the concentration of ARSF during each magnetisation transfer experiment. The plot of [TBAT]

against t is given in Figure S5.29. The decomposition exhibited apparent first-order kinetics for all solutions as well, $[TBAT] = [TBAT]_\infty + ([TBAT]_0 – [TBAT]_\infty)e^{–kt}$, albeit with slightly more noise as gradual broadening of the signal was observed. The fitting parameters and root mean square error (R) were: $[TBAT]_\infty$ = 184 mM, $[TBAT]_0$ = 201 mM, k = 0.154 h$^{–1}$, R = 1 mM. The data for TBAT appears to be relatively noisy towards the later reaction times, possibly due to gradual broadening of the signal. Nevertheless, the concentrations of both species decreased by ~10 mM over the course of the experiment (it appears from the models that slightly more TBAT decomposes than ARSF, although it may be due to experimental error.

Table S5.16: Evolution of concentrations of TBAT and ARSF (based on NAPH).

t (h), TBAT	[TBAT] (mM)	*t* (h), ARSF	[ARSF] (mM)
0.268	201	0.316	67.3
1.38	197	1.40	64.8
2.37	196	2.40	62.8
3.38	195	3.40	62.3
4.38	193	4.41	61.5
5.39	190	5.41	60.7
6.39	190	6.41	60.2
7.38	192	7.40	59.5
8.38	189	8.40	60.0
9.39	189	9.41	58.8
10.4	186	10.4	58.9

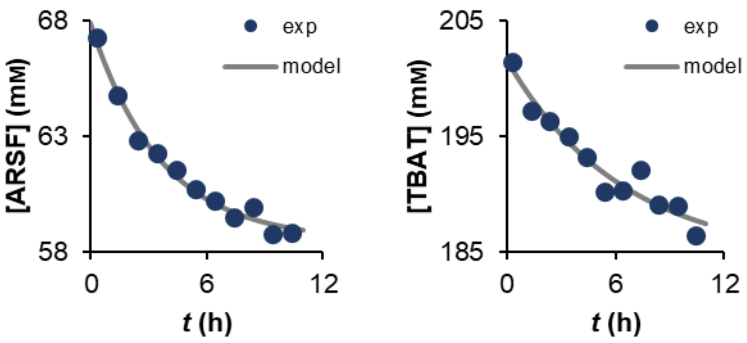

Figure S5.29: Apparent first-order decomposition of ARSF and TBAT in MeCN in a solution of the two species in MeCN, 335 K.

A rapid decomposition of phenyl fluorosilicate (an analogue of ARSF) in the presence of "anhydrous" TBAF was previously reported by Zheng *et al.*[S14] They proposed a defluorosulfation pathway, presented in Scheme S5.7. Similarly, we observed a gradual formation of two small signals resonating around the frequency of ARSF (Figure S5.30a; although their integrals did not make up for the decomposed ARSF and TBAT as presented in Figure S5.30b). We identified them as $^{–}OSO_2F$, by spiking the sample with $KOSO_2F$ (Figure S5.30c); and SO_2F_2, by analogy to the reported chemical shift. The by-product of TBAT decomposition, FTPS, was not observed in the spectra at any point, plausibly due to the line

broadening of the signal. According to Sanford, fluorosulfates with electron-poor aryl moieties may be susceptible to nucleophilic aromatic substitution by fluoride.[S15] Although we used 2-naphthalenyl fluorosulfate (ARSF) as the exchanging partner to TBAT, and 1-fluoronaphthalene (NAPH) as the internal standard, we tested the possibility of formation of NAPH in TBAT/ARSF systems in MeCN by preparing a sample according to the usual procedure, except in the absence of exogenous NAPH. A ^{19}F NMR spectrum of the solution was acquired immediately and after approximately ten hours of keeping the sample at 335 K (Figure S5.30d). The previously observed decomposition products were observed, and no NAPH was detected in either spectrum.

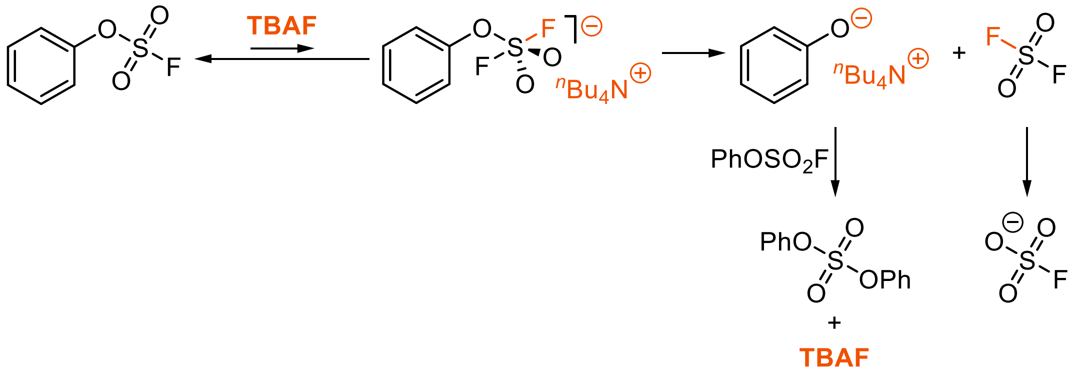

Scheme S5.7: Defluorosulfation of $PhOSO_2F$ (1.52) in the presence of "anhydrous" TBAF, reported by Zheng *et al*.[S14]

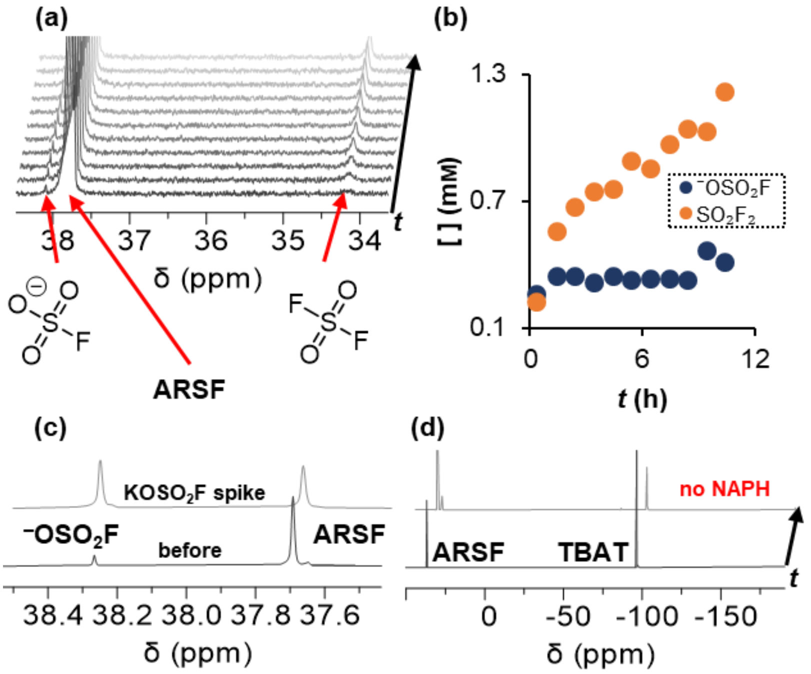

Figure S5.30: (a) ^{19}F NMR spectra of the TBAT/ARSF system in MeCN at 335 K, showing gradual decomposition of the species. Two new ^{19}F spins were observed, identified as $^{-}OSO_2F$ and SO_2F_2. (b) The temporal evolution of the decomposition products. (c) Spiking of the solution with $KOSO_2F$. (d) Probing the formation of NAPH as a decomposition product.

The determined ^{19}F longitudinal relaxation fitting parameters and root mean square errors (I_{eq}, x, T_1, R) for TBAT and ARSF are presented in Table S5.17. An average of both constants were taken, T_1^{TBAT} = (5.70 ± 0.10) s and T_1^{ARSF} = (3.42 ± 0.07) s, and used for fitting the magnetisation transfer model to data.

Table S5.17: Fitting parameters and root mean square errors for non-selective inversion-recovery measurements.

***t* (h)**	**TBAT**				**ARSF**			
	I_{eq}	x	T_1 (s)	R	I_{eq}	x	T_1 (s)	R
0.594	12312	0.672	5.74	68	2020	0.522	3.65	12
1.61	12196	0.676	5.71	42	1968	0.524	3.54	13
2.61	12215	0.684	5.68	79	1930	0.527	3.43	13
3.61	12048	0.686	5.40	69	1919	0.523	3.55	11
4.61	12094	0.672	5.84	63	1892	0.531	3.38	12
5.62	11929	0.676	5.54	66	1882	0.527	3.39	12
6.62	11908	0.678	5.53	72	1862	0.523	3.41	10
7.61	11926	0.678	5.76	80	1851	0.529	3.36	11
8.61	11993	0.669	5.91	83	2020	0.522	3.33	12
9.62	11925	0.673	5.93	99	1968	0.524	3.29	11
10.6	11776	0.682	5.62	79	1930	0.527	3.33	13

The ^{19}F exchange fitting parameters (I_{eq}, m_0^{TBAT} and α_{obs}), and root mean square errors (R) obtained for (the initially non-inverted) ARSF, are presented in Table S5.18.

Table S5.18: Evolution of magnetisation transfer between TBAT and ARSF (based on NAPH).

***t* (h)**	I_{eq}	m_0^{TBAT}	α_{obs} (s^{-1})	R
0.387	3392	0.263	0.0512	11
1.41	3286	0.286	0.0464	11
2.41	3282	0.324	0.0452	11
3.42	3240	0.329	0.0427	14
4.42	3202	0.319	0.0399	15
5.42	3171	0.320	0.0380	12
6.43	3143	0.332	0.0380	14
7.42	3129	0.332	0.0366	15
8.42	3100	0.347	0.0362	15
9.42	3092	0.348	0.0359	17
10.4	3071	0.363	0.0357	12

Magnetisation transfer measurements on the system showed a decrease in the extent of magnetisation transfer in the solution over time (T_1^{TBAT} and T_1^{ARSF} were constant throughout the experiment). In order to interrogate the exchange between the spins, α_{obs} was first determined for each magnetisation transfer measurement, and the plot of α_{obs} *vs* t is presented in Figure S5.31a. According to Equation S5.92, α is a linear function of [ARSF] and hence it is unsurprising that α_{obs} decreased over time. However, the plot of α_{obs}/[ARSF] *vs* t in Figure S5.31b shows that α_{obs} decreased more rapidly than [ARSF]. From Equation S5.92, α/[ARSF] is independent of [TBAT] and [ARSF] for the direct exchange pathway, provided that the rate constant, k_4, is independent of the changing conditions. For the dissociative pathway (again assuming that constants K_1 and k_3 are constant), α/[ARSF] depends on [TBAT] indirectly, as changing [TBAT] shifts the position of the dissociation equilibrium. Moreover, FTPS is a by-

product of decomposition of TBAT, which also affects the equilibrium. Both these effects are governed by the presence of [FTPS] in the denominator of the dissociative pathway term in Equation S5.92. Therefore, here the plot of α_{obs}/[ARSF] *vs* *t* is suggestive of at least a partially dissociative pathway, inhibited over time due to formation of FTPS from decomposing TBAT. The contributions of the dissociative and direct pathways and their temporal evolution were estimated from a plot of α_{obs}/[ARSF] *vs* 1/([TBAT]$_0$ – [TBAT]), Equation S5.105 and Figure S5.31c; K_1k_3 = 2.0·10^{-3} s^{-1}, k_4 = 1.1 M^{-1} s^{-1}, R = 0.974 (the first two measurements were discarded from the fitting due to non-negligible contribution of dissociation of TBAT to the total concentration of FTPS). The value of K_1 ≈ 0.042 mM was estimated based on the discrepancy between the concentration of TBAT weighed into the volumetric flask, [TBAT]$_{\text{theoretical}}$ = 204 mM, and [TBAT]$_0$ = 201 mM determined from the decomposition model. Thus, k_3/k_4 = 43, indicating that TBAF is a significantly more potent direct fluoride donor than TBAT.

S5.105

$$\frac{\alpha_{\text{obs}}}{[\text{ARSF}]} = \frac{K_1k_3}{2[\text{FTPS}]} + \frac{k_4}{2} \approx \frac{K_1k_3}{2([\text{TBAT}]_0 - [\text{TBAT}])} + \frac{k_4}{2}$$

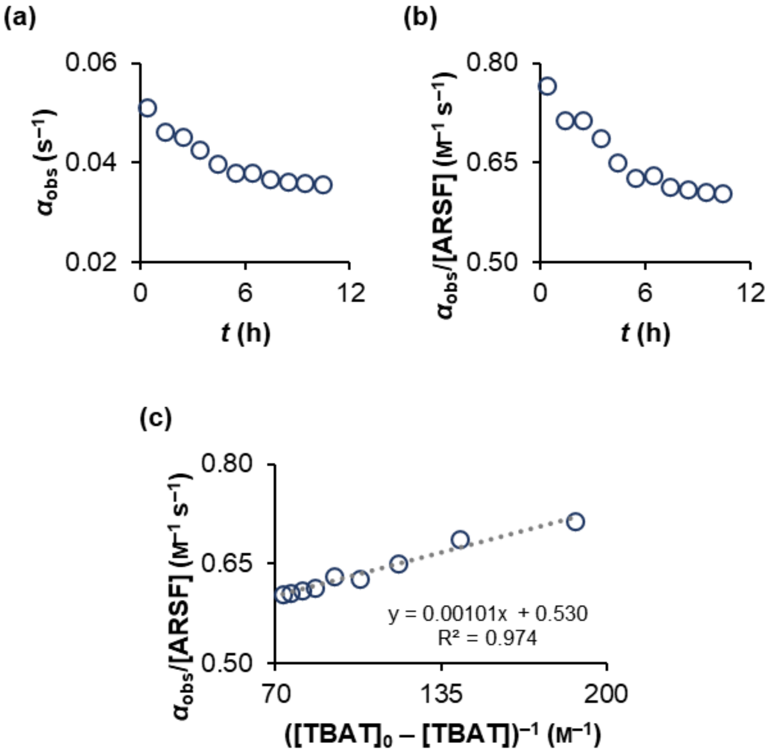

Figure S5.31: Plots of (a) α_{obs} *vs* *t*, (b) α_{obs}/[ARSF] *vs* *t* and (c) α_{obs}/[ARSF] *vs* ([TBAT]$_0$ – [TBAT])$^{-1}$; for the TBAT/ARSF system in MeCN, 335 K.

In order to account for the observed mass imbalance in the decomposing solution of TBAT and ARSF in MeCN, a solution was prepared in the glovebox by weighing TBAT (122 mM), ARSF (131 mM) and NAPH (295 mM) into a 1 mL volumetric flask and dissolving them in

MeCN up to the 1 mL mark. The solution was transferred to a J Young® valve capped NMR tube. The sample was then inserted into an NMR probe precooled to 233 K.[a] Three ^{19}F pulse-acquire NMR spectra were acquired at 233 K; for quantification of TBAT, FTPS and ARSF, against the internal standard (NAPH). The sample was then incubated at 335 K for 15 h, and the ^{19}F NMR spectra were acquired again.

The concentrations of TBAT and ARSF were 122 mM and 130 mM, respectively, prior to the incubation at 335 K. These values are in excellent agreement with the corresponding concentrations based on the masses of the solutes. After keeping the sample at 335 K for 15 h, these concentrations decreased to 113 mM and 116 mM, respectively. Both $^-OSO_2F$ and SO_2F_2 were also observed in these spectra, [$^-OSO_2F$] = 475 μM and [SO_2F_2] = 4.65 mM. FTPS was not observed in the NMR spectra acquired prior to and after the incubation; Figure S5.32a. According to Scheme S5.7, the mass balances with respect to Si and F are presented in Equations S5.106 and S5.107, respectively. The concentration of FTPS expected from the mass balance with respect to F is 150% larger than for the mass balance with respect to Si. This imbalance could plausibly again be explained by progressive line broadening of the signals – even at 233 K – thus confirming that accurate monitoring of the decomposition of TBAT and ARSF in MeCN is very challenging even at low temperatures; Figure S5.32b.

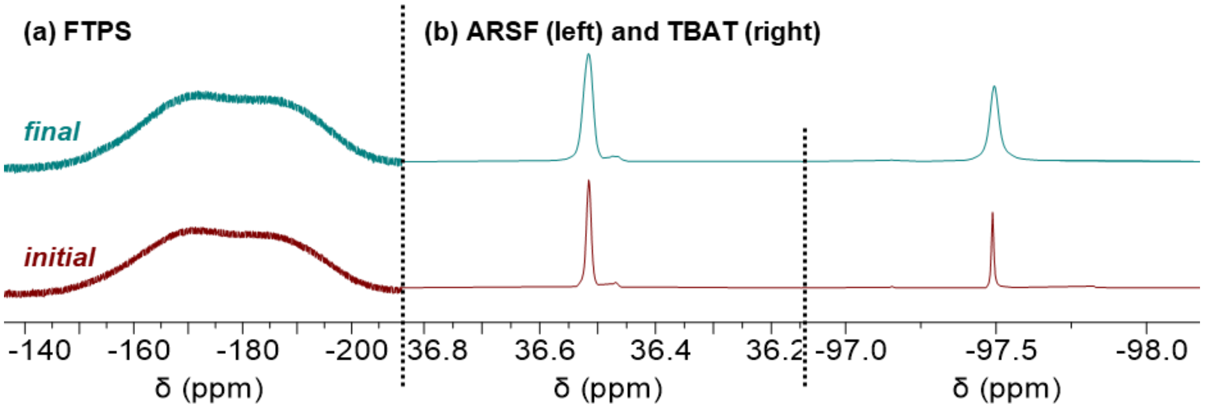

Figure S5.32: ^{19}F NMR spectra at 233 K of the solution of TBAT and ARSF in MeCN prior to (bottom) and after (top) incubating the sample at 335 K for 15 h. (a) FTPS was not observed in either spectrum. (b) Both ARSF and TBAT signals exhibited line broadening upon the heating of the sample at 335 K.

S5.106

$$[\mathrm{FTPS}]_t = [\mathrm{TBAT}]_0 - [\mathrm{TBAT}]_t = 8.67\ \mathrm{mM}$$

S5.107

$$[\mathrm{FTPS}]_t = 2([\mathrm{TBAT}]_0 - [\mathrm{TBAT}]_t) + [\mathrm{ARSF}]_0 - [\mathrm{ARSF}]_t - 2[\mathrm{SO_2F_2}]_t - [\,^{-}\mathrm{OSO_2F}]_t = 21.7\ \mathrm{mM}$$

[a] Bruker UltraShield 400 MHz NMR spectrometer equipped with a BBFO+/TBO broad band probe (^{19}F 377 MHz).

S6. Fluorination of benzyl bromide

Mąkosza and Bujok reported that benzyl bromide (BzBr) undergoes fluorination by TBAT in MeCN at elevated temperature (Scheme S6.1a), a reaction that can be monitored by standard *in situ* techniques.[S16] We envisaged that, if dissociative, the reaction should be inhibited both progressively and by the addition of exogenous FTPS, and if direct, it should be unaffected by FTPS. We therefore studied the initial rates of this fluorination in the absence and presence of exogenous FTPS (Scheme S6.1b), and compared the initial rates of this reaction.

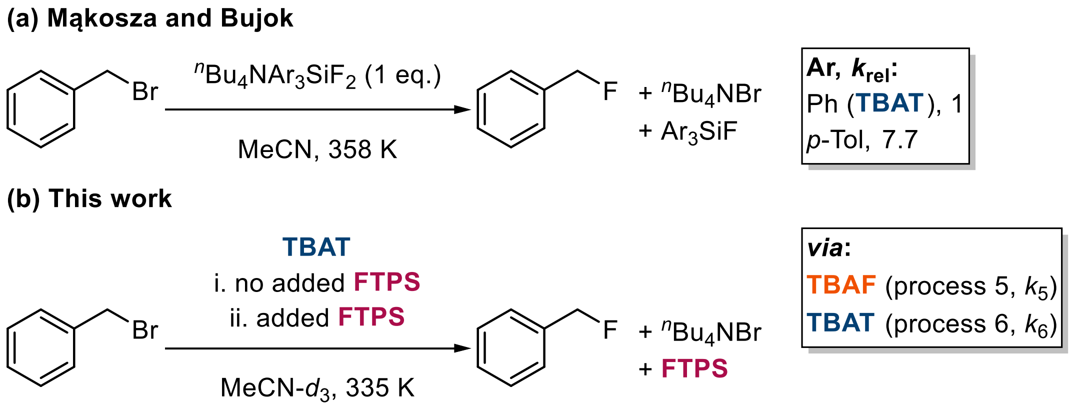

Scheme S6.1: Fluorination of benzyl bromide (BzBr). (a) Relative rate constants of fluorination of BzBr using $^nBu_4NAr_3SiF_2$, where Ar = Ph (TBAT) or *p*-Tol, reported by Mąkosza and Bujok.[S16] (b) In this work, we focused on the fluorination of BzBr using TBAT in the absence and presence of exogenous FTPS, in order to determine the character of the reaction (direct transfer *via* TBAT, k_6, and/or dissociative transfer *via* TBAF, k_5).

S6.1. Fluorination of BzBr in the absence of exogenous FTPS

A solution was prepared in the glovebox by weighing BzBr (131 mM), TBAT (129 mM), TMB (120 mM) and NAPH (136 mM) into a 1 mL volumetric flask and dissolving them in MeCN-d_3 up to the 1 mL mark. The solution was transferred to a J Young® valve capped NMR tube. The date and time of preparation of the solution was noted. The sample was immediately removed from the glovebox, inserted into the NMR probe preheated to 335 K, and kept there for the duration of the experiment.

Thirteen sets of ^{1}H and ^{19}F (8 scans for both ^{1}H and ^{19}F) pulse-acquire experiments were performed over time at 335 K. The ^{1}H NMR spectra were used to quantify BzBr (CH_2 signal; δ_H = 4.60 ppm, T_1 = 5.89 s) against TMB (CH_3 signal; δ_H = 3.76 ppm, T_1 = 4.05 s); and the ^{19}F NMR spectra – benzyl fluoride (BzF; δ_F = –205.49 ppm) against NAPH (δ_F = –123.95 ppm, T_1 = 6.04 s). (The longitudinal relaxation times of BzBr, TMB and NAPH, quoted above, were determined from inversion-recovery experiments of these species in MeCN-d_3 at 335 K).

The concentrations of BzBr and BzF based on the internal standards are presented in Table S6.1. The initial concentration of BzBr, $[BzBr]_0$ = 129 mM, was determined by minimising the

sum of squares for experimental [BzBr] and [BzF], and is in agreement with the concentration of BzBr based on the mass of the reagent used to prepare the solution (131 mM). The plots of (i) concentration of BzBr and (ii) the difference between the initial concentration of BzBr and the temporal concentration of BzF, $[BzBr]_0$ – [BzF], against time, are presented in Figure S6.1a. The plot of 1/[BzBr] against time is presented in Figure S6.1b, and the expansion of the plot to the first 3.10 h of the reaction – in Figure S6.1c. The intercepts of the linear trend lines in the reciprocal plots were set to $1/[BzBr]_0$ = 7.77 M^{-1}.

Table S6.1: Concentrations of BzBr and BzF in the reaction of BzBr with TBAT (MeCN-d_3, 335 K).

$10^{-3}t$ (s), ^{1}H	[BzBr] (mM)	$10^{-3}t$ (s), ^{19}F	[BzF] (mM)
2.28	125	2.64	3.34
4.02	122	4.32	6.24
5.70	120	6.00	8.54
7.50	118	7.80	10.6
9.36	116	9.66	12.3
11.2	115	11.5	13.8
14.8	112	15.1	17.0
18.4	109	18.7	19.6
22.0	107	22.3	21.9
25.6	105	25.9	24.1
32.9	101	33.2	28.0
40.0	97.7	40.3	31.5
47.2	94.8	47.5	34.4

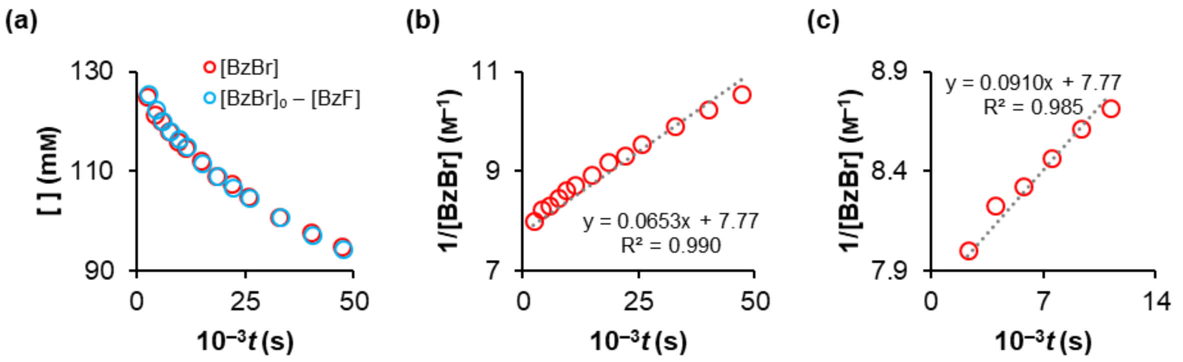

Figure S6.1: Fluorination of BzBr with TBAT in MeCN-d_3 at 335 K. (a) [BzBr] and $[BzBr]_0$ – [BzF] against time, (b) 1/[BzBr] against time, and (c) the expansion of the reciprocal plot to the first 3.10 h of the reaction.

As shown in Figure S6.1a, the formation of BzF in the solution mirrored the decay of BzBr, with the sum of their concentrations constant throughout the experiment. After 13.1 h, 26.4% of BzBr was converted to BzF. The pseudo second-order rate constant of the reaction, based on the reciprocal plot in Figure S6.1b, was determined as $7{\cdot}10^{-5}$ M^{-1} s^{-1}, R^2 = 0.990. The curvature in the plot is consistent with progressive inhibition of the reaction. Indeed, the first 3.10 h of the reaction, Figure S6.1c, show the *initial* pseudo second-order rate constant of $9{\cdot}10^{-5}$ M^{-1} s^{-1}, R^2 = 0.985.

S6.2. Fluorination of BzBr in the presence of exogenous FTPS

A solution was prepared in the glovebox by weighing BzBr (111 mM), TBAT (130 mM), TMB (170 mM), NAPH (151 mM) and FTPS (140 mM) into a 1 mL volumetric flask and dissolving them in MeCN-d_3 up to the 1 mL mark. The solution was transferred to a J Young® valve capped NMR tube. The date and time of preparation of the solution was noted. The sample was immediately removed from the glovebox, inserted into the NMR probe preheated to 335 K, and kept there for the duration of the experiment.

Ten sets of ^{1}H and ^{19}F (8 scans for both ^{1}H and ^{19}F) pulse-acquire experiments were performed over time at 335 K. The ^{1}H NMR spectra were used to quantify BzBr (CH_2 signal; δ_H = 4.60 ppm, T_1 = 5.89 s) against TMB (CH_3 signal; δ_H = 3.76 ppm, T_1 = 4.05 s); and the ^{19}F NMR spectra – benzyl fluoride (BzF; δ_F = –205.49 ppm) against NAPH (δ_F = –123.95 ppm, T_1 = 6.04 s). (The longitudinal relaxation times of BzBr, TMB and NAPH, quoted above, were determined from inversion-recovery experiments of these species in MeCN-d_3 at 335 K).

The ^{19}F signal of TBAT was very broad in the NMR spectra, and no FTPS was observed in the ^{19}F NMR spectra over the duration of the experiment. The concentrations of BzBr and BzF based on the internal standards are presented in Table S6.2. The initial concentration of BzBr, $[BzBr]_0$ = 110 mM, was determined by minimising the sum of squares for experimental [BzBr] and [BzF], and is in agreement with the concentration of BzBr based on the mass of the reagent used to prepare the solution (111 mM). The plots of (i) concentration of BzBr and (ii) the difference between the initial concentration of BzBr and the temporal concentration of BzF, $[BzBr]_0$ – [BzF], against time, are presented in Figure S6.2a. The plot of 1/[BzBr] against time is presented in Figure S6.2b, and the expansion of the plot to the first 2.98 h of the reaction – in Figure S6.2c. The intercepts of the linear trend lines in the reciprocal plots were set to $1/[BzBr]_0$ = 9.06 M^{-1}.

Table S6.2: Concentrations of BzBr and BzF in the reaction of BzBr with TBAT in the presence of exogenous FTPS (MeCN-d_3, 335 K).

$10^{-3}t$ (s), ^{1}H	[BzBr] (mM)	$10^{-3}t$ (s), ^{19}F	[BzF] (mM)
3.60	108	3.90	1.55
5.34	108	5.70	2.24
7.20	107	7.50	3.02
9.00	106	9.30	3.76
10.7	106	11.1	4.69
14.4	104	14.7	6.32
18.1	103	18.4	7.87
21.6	101	21.9	9.32
25.1	100	25.5	10.7
32.4	97.5	32.7	14.0

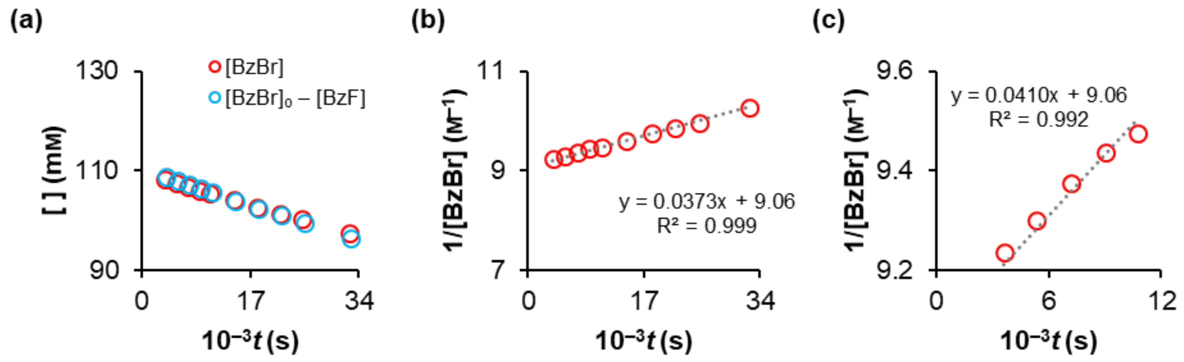

Figure S6.2: Fluorination of BzBr with TBAT in the presence of exogenous FTPS in MeCN-*d*$_3$ at 335 K. (a) [BzBr] and [BzBr]$_0$ – [BzF] against time, (b) 1/[BzBr] against time, and (c) the expansion of the reciprocal plot to the first 2.98 h of the reaction.

As shown in Figure S6.2a, the formation of BzF in the solution mirrored the decay of BzBr, with the sum of their concentrations constant throughout the experiment. After 9.00 h, 11.6% of BzBr was converted to BzF (compared to 21.6% over 9.13 h in the absence of exogenous FTPS). The pseudo second-order rate constant of the reaction, based on the reciprocal plot in Figure S6.2b, was determined as $4{\cdot}10^{-5}$ M^{-1} s^{-1}, R^2 = 0.999. Notably, the reciprocal plot in Figure S6.2b does not exhibit the curvature observed for the reaction in the absence of exogenous FTPS, indicating little progressive inhibition of the reaction. The first 2.98 h of the reaction, Figure S6.2c, show a nearly identical *initial* pseudo second-order rate constant of $4{\cdot}10^{-5}$ M^{-1} s^{-1}, R^2 = 0.992; nearly identical to that in Figure S6.2b. This result is expected even if the reaction is (at least partially) dissociative, as in the presence of >1 equivalent (140 mM) of exogenous FTPS, any FTPS formed as a by-product of the reaction (~13 mM, overall) is negligible to the overall [FTPS], and hence does not affect the rate of the reaction significantly.

Furthermore, the determined pseudo second-order rate constant in the presence of exogenous FTPS is 43% smaller than that in the absence of it (55% for the initial ~3 h in both reactions). These values indicate inhibition of the reaction by the exogenous FTPS, which in turn indicates partially dissociative character to the exchange (proceeding in parallel with the direct transfer pathway). However, compared to the reaction between TBAT and ARSF in MeCN at 335 K, where an 8.5% conversion of (initially) 201 mM TBAT into FTPS decreased the measured α by 30%. The comparison of these results indicates a significantly lesser contribution of the dissociative pathway to the overall fluoride transfer in the fluorination of benzyl bromide *cf*. the TBAT/ARSF exchange in MeCN(-*d*$_3$) at 335 K. Indeed, the *initial* standard state partitioning in this case is estimated to be $k_6(c_{\mathrm{TBAT}}°)^{0.5}/(K_1)^{0.5}k_5 \approx 1$.

S7. Appendices

S7.1. Principles of magnetisation transfer experiments

Magnetisation transfer methods detect the transfer of longitudinal magnetisation between exchanging spins of different resonance frequencies. The first such method was introduced in 1963 by Forsén and Hoffman,[S17] who used double-resonance continuous-wave ^{1}H NMR spectroscopy to study acetic acid-catalysed proton exchange between salicylaldehyde and 2'-hydroxyacetophenone. One year later, they generalised the method to multi-site exchange networks.[S18]

All magnetisation transfer methods follow the same principle: selective disturbance of the longitudinal magnetisation of one (or more) spins is detected in all spins which exchange with the initially perturbed one(s), provided sufficiently rapid exchange rate relative to longitudinal relaxation under the applied conditions. Two commonly used magnetisation transfer methods (in pulsed NMR spectroscopy) are exemplified herein on a two-site A ⇌ B exchange system, where A and B each contain an NMR-observable spin-half nucleus. For simplicity, we assume no cross-relaxation and no scalar couplings in the system.[S19,S20]

S7.1.1. Chemical exchange saturation transfer (CEST)

Chemical exchange saturation transfer (CEST) is a magnetisation transfer method, which enables one to observe low-concentration species present in solution, provided that they exchange with an observable spin to a sufficient extent compared to longitudinal relaxation.[S21,S22] In a typical CEST experiment (Figure S7.1a), a series of narrow frequency ranges are in turn saturated for a certain time, called the saturation time, t_{sat}. The saturation of each frequency range is followed by a pulse-acquire sequence, which after Fourier transformation yields a series of NMR spectra. The magnetisation of the observable spin is then plotted against saturation frequency and if a low-concentration spin is present in the scanned range and the rate of exchange considerable, a decrease in the magnetisation of the observable spin is observed for that frequency. Consider a system where A and B are at chemical equilibrium, and the degree of conversion of A to B is small, K = [B]/[A] << 1 (Figure S7.1b). In an NMR spectrum of the equilibrium mixture, only A is observed at its resonance frequency, δ_A, and B (resonating at δ_B) is below detection. When a frequency range is selectively saturated where neither A nor B resonate, the spectrum shows equilibrium longitudinal magnetisation for A (and no signal for B). When a frequency range containing δ_A is saturated, the resulting spectrum shows the magnetisation of A suppressed to (in theory) 0. Saturation of the frequency range containing B, δ_B, results in the decrease of magnetisation

of A, to the extent which depends on the saturation time, exchange rate and longitudinal relaxation times of A and B (Figure S7.1c–d).

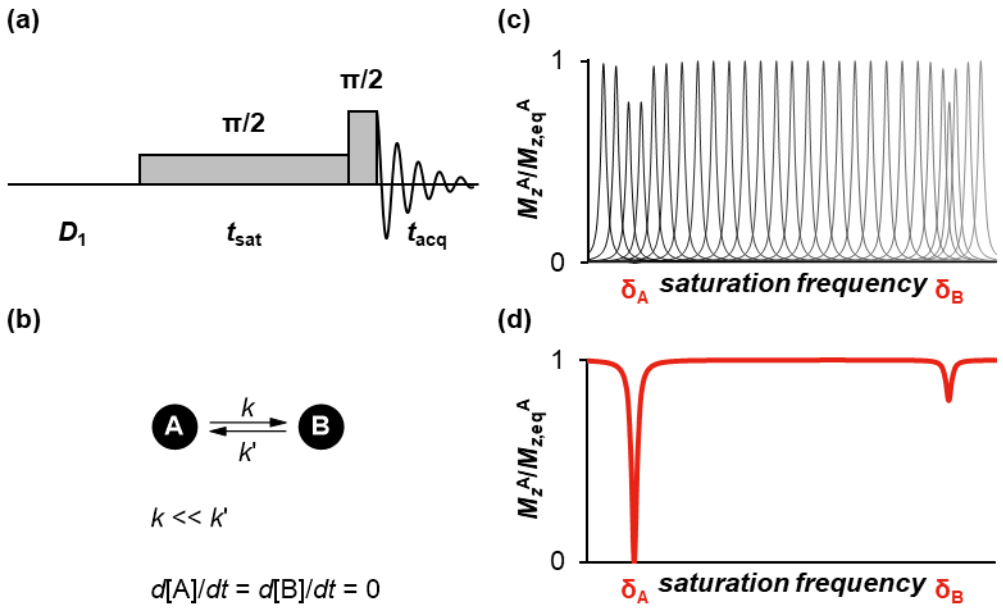

Figure S7.1: (a) The pulse sequence of a CEST experiment. Initially (provided a sufficiently long relaxation delay, D_1, is used), all spins are at magnetic equilibrium along the +*z*-axis. Selective saturation of a narrow frequency range using a soft π/2 pulse is followed by immediate detection of the longitudinal magnetisation of an observable spin (with acquisition time t_{acq}). The sequence is repeated for several frequency ranges. (b) An A ⇌ B general system is used herein to describe the principles of a CEST experiment. A and B exchange *via* a unimolecular elementary step in each direction, with unequal rate constants. The system is at dynamic equilibrium and B in low concentration (below the detection limit in pulse-acquire NMR spectra). (c) Simulated peaks of A detected for a series of saturation frequencies immediately after saturation, and (d) the corresponding simulated plot of normalised magnetisation of A against the saturation frequency (*i.e.* the centre frequency of the range). δ_A and δ_B are the chemical shifts of A and B, respectively.

S7.1.2. Magnetisation transfer *via* selective inversion

A magnetisation transfer experiment (Figure S7.2a), developed by Dahlquist, Longmuir and Vernet for an equally-populated two-spin system,[S12] and subsequently generalised by Alger and Prestegard,[S23] and Brown and Ogawa,[S24] uses a soft (frequency-selective) π pulse to invert one of the two spins to the –*z*-axis, whereas the second spin remains at magnetic equilibrium. The soft pulse is followed by a variable delay, during which the magnetisation vectors of the spins evolve *via* longitudinal relaxation and, if present, exchange, prior to the signal detection. This pulse sequence is repeated for incremented variable delays. Consider a scenario where A and B are of equal populations and exchange *via* an elementary unimolecular reaction with the rate constant *k* (in both directions). The longitudinal relaxation time constants of the spins are equal, and such that $kT_1 = 1$ (Figure S7.2b). Initially, both spins are at magnetic equilibrium along the +*z*-axis (Figure S7.2c). This equilibrium corresponds to the “standard” NMR spectrum of the mixture of A and B. The soft (frequency-selective) π pulse

inverts one signal, here – A, to the $-z$-axis, whilst leaving the magnetisation of the other spin (B) intact. If the resulting longitudinal magnetisations are detected immediately (variable delay, $\tau = 0$), and assuming perfect inversion by the soft pulse and no relaxation nor exchange during the irradiation by the pulses, the acquired NMR spectrum shows negative equilibrium longitudinal magnetisation, $-M_{z,eq}$, for A, and equilibrium longitudinal magnetisation, $M_{z,eq}$, for B. As the variable delay, τ, increases, two processes occur for both spins: (i) the redistribution of magnetisation between A and B heading towards equal (fractional) magnetisations resulting from chemical exchange, and (ii) longitudinal relaxation of both spins. Both these processes increase the magnetisation of the inverted spin, whereas for the non-inverted spin they act in the opposite fashion (exchange decreases and relaxation increases its magnetisation). The result is, for the inverted spin, a more rapid recovery, and for the non-inverted spin – an initial decrease in magnetisation, and then recovery. Finally, as the variable delay approaches infinity, both spins recover fully to their magnetic equilibrium. In practice, for the A $\rightleftharpoons$ B system herein, $M_z^A = M_z^B = 0.993M_{z,eq}$ is achieved for both spins at $\tau = 5T_1$.

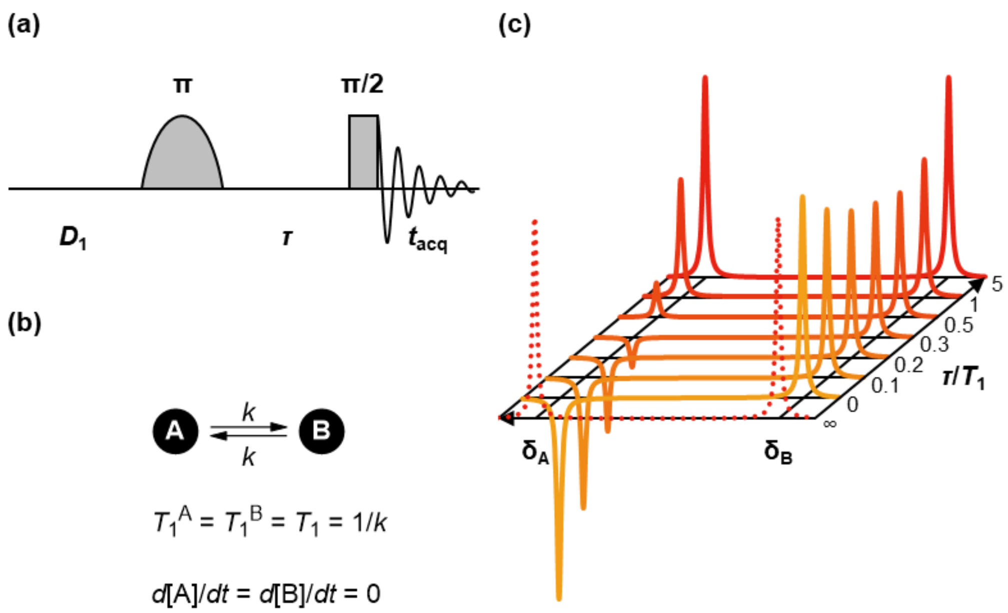

Figure S7.2: (a) The pulse sequence of a magnetisation transfer experiment. Initially (provided a sufficiently long relaxation delay, D_1, is used), all spins are at magnetic equilibrium along the +*z*-axis. Selective inversion of one or more spins using a soft π pulse is followed by a variable delay, τ, during which the spins exchange and relax. The resulting longitudinal magnetisations are then detected using a hard π/2 pulse to rotate the magnetisation onto the XY-plane (with acquisition time t_{acq}). The sequence is repeated for incremented variable delays. (b) An A ⇌ B general system is used here to describe the principles of a magnetisation transfer experiment. A and B undergo unimolecular exchange with rate constant *k* in each direction and $kT_1 = 1$, where T_1 is the longitudinal relaxation time constant, equal for both spins, for simplicity. (c) A simulation of the spectra at different stages of a magnetisation transfer experiment for the system described in (b), where δ_A and δ_B are the chemical shifts of A and B, respectively. Initially (dotted spectrum), the spins are at magnetic equilibrium. Selective inversion of A and immediate acquisition results in the second spectrum in the series (from the bottom). Increasing variable delay results in more magnetisation transfer between the spins, as well as their recovery *via* longitudinal relaxation. Finally, at sufficiently long variable delays, τ, the spins return to magnetic equilibrium.

Figure S7.3a–b shows a plot of the normalised longitudinal magnetisations of the two spins ($M_z/M_{z,eq}$) against the ratio of variable delay to longitudinal relaxation time, τ/T_1, for the system described above ($kT_1 = 1$), as well as for $kT_1 = 0, 2, 5, 50$. The dotted lines represent A (the inverted spin) and the continuous lines of the same colour – B (the initially non-inverted spin). If there is no exchange between the spins ($kT_1 = 0$), the recovery of the inverted signal (A) follows first-order longitudinal relaxation kinetics with the rate constant $1/T_1$, and the non-inverted signal (B) is unaffected by the soft pulse and remains at magnetic equilibrium regardless of variable delay. If exchange is present ($kT_1 = 1, 2, 5, 50$ in the plot), the recovery of A becomes faster and no longer first-order, whereas the magnetisation of B *vs* τ gives a characteristic "V-shaped" plot. The plot is asymmetrically-stretched, and the minimum of the function (*i.e.*, the point where $dM_z^B/d\tau = 0$) heads towards lower τ and M_z^B as kT_1 increases (Figure S7.3c–d).

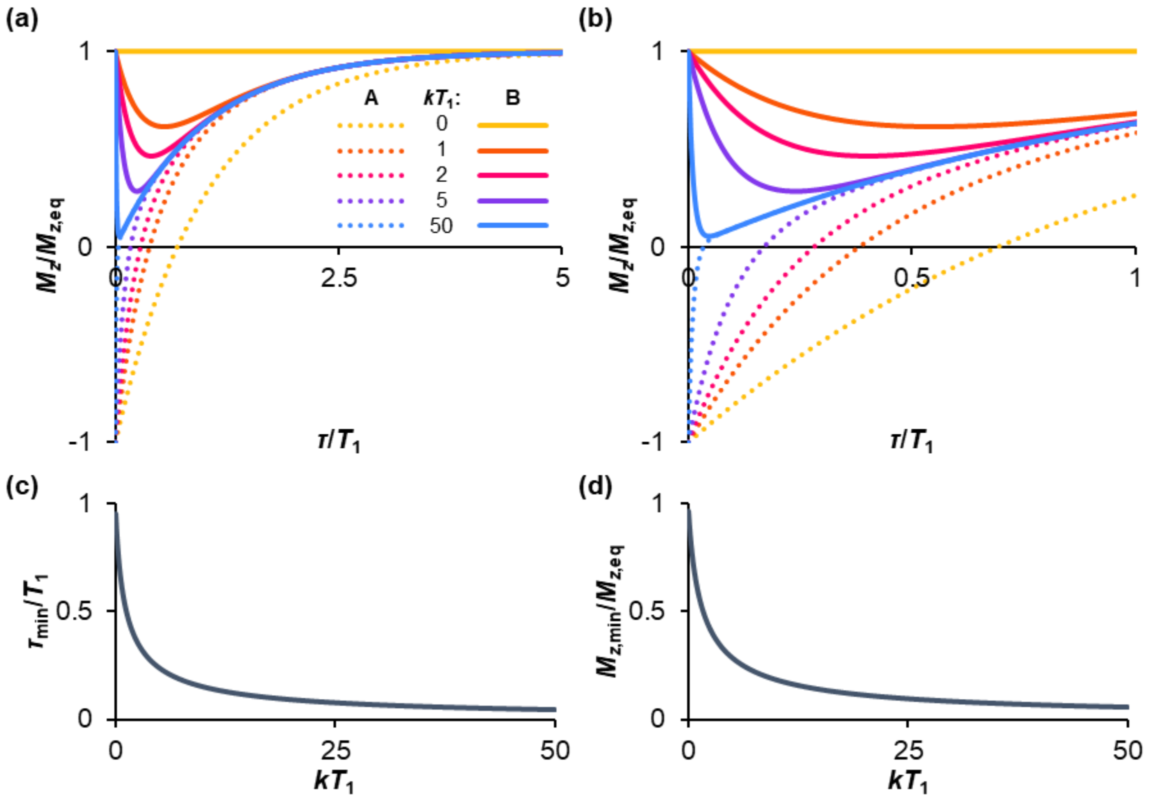

Figure S7.3: (a) The evolution of longitudinal magnetisation of exchanging spins depending on the exchange rate constant relative to relaxation. Lines with the same colour represent the inverted spin (A, dotted lines) and the initially non-inverted one (B, continuous lines). (b) An expansion of the plot from (a) for $\tau \leq T_1$. Magnetisation of the initially non-inverted signal reaches the minimum (c) more rapidly and (d) with larger relative decrease in magnetisation – with increased kT_1.

Quantitative description of the evolution of longitudinal magnetisation in magnetisation transfer experiments is derived from Bloch equations modified for exchange.[S25–S27] For an (equally- or unequally-populated) n-site exchanging system ($n \geq 2$), a system of n coupled differential equations which describe the rate of change of longitudinal magnetisation at each site is presented in Equation S7.1,[S18] where M_z^i is the longitudinal magnetisation of site i at variable delay τ, $M_{z,\mathrm{eq}}^i$ is the corresponding equilibrium magnetisation (*i.e.*, for the fully magnetised spin), k_{ij} is the first-order rate constant of exchange from i to j, and T_1^i is the longitudinal relaxation time constant of i.

S7.1

$$\frac{dM_z^i}{d\tau} = -\sum_{\substack{j=1 \\ (i\neq j)}}^{n} k_{ij} M_z^i + \sum_{\substack{j=1 \\ (i\neq j)}}^{n} k_{ji} M_z^j + \frac{M_{z,\mathrm{eq}}^i - M_z^i}{T_1^i}$$

For magnetisation transfer experiments, and when $n = 2$, this model leads to longitudinal magnetisations of both sites (A and B, M_z^A and M_z^B) as biexponential functions of the variable delay, τ; Equations S7.2–S7.5, where A is inverted.[S28,S29] Fractional inversion of A, $f =$ $= -M_{z,0}^A/M_{z,\mathrm{eq}}^A$, where $M_{z,0}^A$ is the magnetisation of A immediately after inversion, was

introduced to account for imperfect inversion by the soft π pulse. For a perfect inversion scenario, $f = 1$.

S7.2

$$\frac{M_z^{\mathrm{A}}}{M_{z,\mathrm{eq}}^{\mathrm{A}}} = \frac{f+1}{2\gamma}\left[\left(\beta - \gamma + k_{\mathrm{AB}} + \frac{1}{T_1^{\mathrm{A}}}\right)e^{(\beta+\gamma)\tau} - \left(\beta + \gamma + k_{\mathrm{AB}} + \frac{1}{T_1^{\mathrm{A}}}\right)e^{(\beta-\gamma)\tau}\right] + 1$$

S7.3

$$\frac{M_z^{\mathrm{B}}}{M_{z,\mathrm{eq}}^{\mathrm{B}}} = \frac{k_{\mathrm{BA}}(f+1)}{2\gamma}\left[e^{(\beta-\gamma)\tau} - e^{(\beta+\gamma)\tau}\right] + 1$$

Where:

S7.4

$$\beta = -\frac{k_{\mathrm{AB}} + k_{\mathrm{BA}} + \frac{1}{T_1^{\mathrm{A}}} + \frac{1}{T_1^{\mathrm{B}}}}{2}$$

S7.5

$$\gamma = \frac{\sqrt{\left(k_{\mathrm{AB}} + \frac{1}{T_1^{\mathrm{A}}} - k_{\mathrm{BA}} - \frac{1}{T_1^{\mathrm{B}}}\right)^2 + 4k_{\mathrm{AB}}k_{\mathrm{BA}}}}{2}$$

As *n* increases, data analysis becomes more complex. For $n = 3$, the general solution is a triexponential function,[S30] which can be simplified to a biexponential one in specific exchange scenarios.[S18] For $n \geq 4$, numerical solutions can be obtained with an aid of appropriate software.[S31,S32] Alternatively, *n*-site exchanging systems can be simplified to two-site ones by continuous saturation of all spins but the two of interest, concurrently to the magnetisation transfer.[S33]

Another approach to deriving these models is the formalism whereby discrete inverted and non-inverted spins are treated as separate, although chemically degenerate, species.[S12] This formalism was used in the first magnetisation transfer experiment, as well as in this study, and leads to identical results to these described above, based on bulk magnetisations.

S7.1.3. Selective pulses

A variety of soft (frequency-selective) pulses can be used in magnetisation transfer experiments,[S34] and the selection of a suitable pulse depends primarily on the method (magnetisation transfer *via* selective inversion or saturation, CEST *etc.*), separation of the signals and exchange rate. The simplest soft pulse is a long rectangular pulse with low power. It is typically avoided in magnetisation transfer experiments, as information about exchange and relaxation processes occurring during the long pulse duration is lost. Poor selectivity, due to far-extending side-lobes, is also characteristic to the pulse. More commonly, it is used in

CEST (as presented in Figure S7.1a), although in this method, series of back-to-back shaped pulses (“pulse trains”) are also used (*e.g.*, in this study; see Section S4.3).[S35]

S7.2. Mathematics

S7.2.1. Relaxation rate constants of TBAT$^{\blacktriangledown\blacktriangledown}$ and TBAT$^{\blacktriangledown\blacktriangle}$, relative to T_1^{TBAT}

The relaxation of TBAT (Scheme S5.3) is a sequence of two consecutive first order processes. Assuming that the relaxation of each of the equivalent ^{19}F nuclei in TBAT is independent of the magnetisation state of the other, the first step is twice as likely as the second one, 2*k* and *k*, respectively (Scheme S7.1). The integrated rate law for this reaction sequence is shown in Equations S7.6–S7.8 (where x^{AA}, x^{AB} and x^{BB} are the molar fractions of AA, AB and BB, and assuming initially only AA is present, $x_0^{\mathrm{AA}} = 1$).

$$\mathbf{AA} \xrightarrow{2k} \mathbf{AB} \xrightarrow{k} \mathbf{BB}$$

Scheme S7.1: A sequence of two consecutive unimolecular reactions where equivalent and independent reactive sites A react to form B.

S7.6

$$x^{\mathrm{AA}} = e^{-2kt}$$

S7.7

$$x^{\mathrm{AB}} = -2(e^{-2kt} - e^{-kt})$$

S7.8

$$x^{\mathrm{BB}} = 1 + e^{-2kt} - 2e^{-kt}$$

The fractional conversion (in Scheme S7.1, that of reactive sites A into B), which corresponds to the fractional magnetisation of TBAT (for AA ≡ TBAT$^{\blacktriangledown\blacktriangledown}$, AB ≡ TBAT$^{\blacktriangledown\blacktriangle}$ and BB ≡ TBAT$^{\blacktriangle\blacktriangle}$), is therefore given by $x^{\mathrm{AB}}/2 + x^{\mathrm{BB}}$. From Equations S7.7 and S7.8, this gives:

S7.9

$$\frac{x^{\mathrm{AB}}}{2} + x^{\mathrm{BB}} = 1 - e^{-kt}$$

According to the standard T_1 inversion-recovery equation:

S7.10

$$\frac{M_z^{\mathrm{TBAT}}}{M_{z,\mathrm{eq}}^{\mathrm{TBAT}}} = 1 - 2e^{-\frac{\tau}{T_1^{\mathrm{TBAT}}}}$$

Equation S7.10 can be expressed as fractional magnetisation, m^{TBAT}, *vs* variable delay (see Equation S5.6):

S7.11

$$m^{\mathrm{TBAT}} = 1 - e^{-\frac{\tau}{T_1^{\mathrm{TBAT}}}}$$

The fractional magnetisation in Equation S7.11 corresponds to the fractional conversion in Equation S7.9 and therefore:

S7.12

$$e^{-\frac{\tau}{T_1^{\mathrm{TBAT}}}} = e^{-kt}$$

Where t and τ both correspond to reaction time (from *time* = 0) and hence represent the same value.

Therefore, $1/T_1^{\mathrm{TBAT}}$ is equal to the rate constant of the second relaxation step, k, (*i.e.* half the rate constant of the first step), as shown in Scheme S5.3.

S7.2.2. Derivation of constants c_1–c_4

The constants c_1–c_4 can be determined as follows (where m_0^{TBAT} and m_0^{FTPS} are the fractional magnetisations of TBAT and FTPS at τ = 0).

Firstly, Equations S5.43 and S5.44 at τ = 0 give:

S7.13

$$m_0^{\mathrm{TBAT}} = c_1 + c_2 + 1$$

S7.14

$$m_0^{\mathrm{FTPS}} = c_3 + c_4 + 1$$

The first derivatives of Equations S5.43 and S5.44 at τ = 0 are:

S7.15

$$\left(\frac{dm^{\mathrm{TBAT}}}{d\tau}\right)_0 = \beta(c_1 + c_2) + \gamma(c_1 - c_2)$$

S7.16

$$\left(\frac{dm^{\mathrm{FTPS}}}{d\tau}\right)_0 = \beta(c_3 + c_4) + \gamma(c_3 - c_4)$$

Also, Equations S5.37 and S5.38 at τ = 0 are:

S7.17

$$\left(\frac{dm^{\mathrm{TBAT}}}{d\tau}\right)_0 = \frac{1}{T_1^{\mathrm{TBAT}}}\left(1 - m_0^{\mathrm{TBAT}}\right) + \alpha\left(m_0^{\mathrm{FTPS}} - m_0^{\mathrm{TBAT}}\right)$$

S7.18

$$\left(\frac{dm^{\mathrm{FTPS}}}{d\tau}\right)_0 = \frac{1}{T_1^{\mathrm{FTPS}}}\left(1 - m_0^{\mathrm{FTPS}}\right) + r\alpha\left(m_0^{\mathrm{TBAT}} - m_0^{\mathrm{FTPS}}\right)$$

Solving the system of Equations S7.13–S7.18 gives the constants c_1–c_4.

S5.47, recalled

$$c_1 = \frac{\alpha\left(m_0^{\mathrm{FTPS}} - m_0^{\mathrm{TBAT}}\right) + \left(\beta - \gamma + \frac{1}{T_1^{\mathrm{TBAT}}}\right)\left(1 - m_0^{\mathrm{TBAT}}\right)}{2\gamma}$$

S5.48, recalled

$$c_2 = -\frac{\alpha\left(m_0^{\mathrm{FTPS}} - m_0^{\mathrm{TBAT}}\right) + \left(\beta + \gamma + \frac{1}{T_1^{\mathrm{TBAT}}}\right)\left(1 - m_0^{\mathrm{TBAT}}\right)}{2\gamma}$$

S5.49, recalled

$$c_3 = \frac{r\alpha\left(m_0^{\mathrm{TBAT}} - m_0^{\mathrm{FTPS}}\right) + \left(\beta - \gamma + \frac{1}{T_1^{\mathrm{FTPS}}}\right)\left(1 - m_0^{\mathrm{FTPS}}\right)}{2\gamma}$$

S5.50, recalled

$$c_4 = -\frac{r\alpha\left(m_0^{\mathrm{TBAT}} - m_0^{\mathrm{FTPS}}\right) + \left(\beta + \gamma + \frac{1}{T_1^{\mathrm{FTPS}}}\right)\left(1 - m_0^{\mathrm{FTPS}}\right)}{2\gamma}$$

S7.2.3. Dissociative pathway in the non-stabilised TBAT/FTPS system

We start from Equation S5.59, which describes the fractional magnetisation of TBAT in TBAT/FTPS systems upon selective inversion of FTPS.

S5.59, recalled

$$m^{\mathrm{TBAT}} = \frac{\Delta m_0}{r+1}\left[e^{-\alpha(r+1)\tau} - e^{-\frac{\tau}{T_1^{\mathrm{obs}}}}\right] + 1$$

The (concentration-dependent) observed rate constant of magnetisation transfer between the spins is $\alpha(r + 1)$, where:

S5.35, recalled

$$\alpha = \frac{k_1 + k_2[\mathrm{FTPS}]}{2}$$

S5.36, recalled

$$r = \frac{M_{z,\mathrm{eq}}^{\mathrm{TBAT}}}{M_{z,\mathrm{eq}}^{\mathrm{FTPS}}} = \frac{2[\mathrm{TBAT}]}{[\mathrm{FTPS}]}$$

We start with a solution of TBAT ([TBAT]) and FTPS ([FTPS]) and measure $\alpha(r + 1)$ using a selective inversion-recovery experiment. We then titrate the solution with solid FTPS and measure $\alpha(r + 1)$ after each aliquot addition. To simplify mathematical operations, we assume the following:

- Each aliquot increases the concentration of FTPS by [FTPS] (*i.e.* its concentration in the original solution).

- The selective inversion-recovery experiments are acquired in equal time intervals (*t*) and themselves are of negligible duration. The time between preparation of the original solution and the first acquisition is also *t*.
- k_1 is a linear function of time with slope = 2*a* and intercept = 2*b*, which are independent of the concentration of FTPS.
- k_2 = 0, independently of time.

Then:

S7.19

$$\alpha(r+1) = (b + at + ait)\left(\frac{2[\mathrm{TBAT}]}{[\mathrm{FTPS}] + i[\mathrm{FTPS}]} + 1\right) = (b + at + ait)\left(\frac{r}{i+1} + 1\right)$$

Where *i* is the aliquot number (*i* = 0 is the original solution, *i* = 1 is the solution after first aliquot *etc*.).

Taking a derivative of this equation with respect to *i*:

S7.20

$$\frac{d[\alpha(r+1)]}{di} = \frac{at[\mathrm{FTPS}](i+1) - 2b[\mathrm{TBAT}]}{[\mathrm{FTPS}](i+1)^2} = \frac{at(i+1) - br}{(i+1)^2}$$

This function is decreasing when:

S7.21

$$(i+1) < \frac{br}{at}$$

Of course, *i* is fixed as {0, 1, 2…}. Assuming that the coefficients *a* and *b* are also fixed (*i.e.* the inherent temporal instability of the system cannot be changed), there are two ways to maximise the chance of observing a decrease in exchange rate between TBAT and FTPS in a titration of the former with the latter: (i) maximising *r* and (ii) minimising *t*. In other words, preparing a very concentrated solution of TBAT and titrating it with very small amounts of FTPS (although large enough for sufficient signal-to-noise ratio) may show a decrease in the exchange rate, followed by an increase towards higher *i*. Decreasing time, *t*, between the runs will also have an effect. Similarly, minimising [FTPS] will decrease the relative contribution of direct transfer pathway to the net exchange rate.

Assuming all of the above, except this time k_2 > 0 (and independent of time).

S7.22

$$\alpha(r+1) = \left(b + at + ait + \frac{k_2[\mathrm{FTPS}] + k_2 i[\mathrm{FTPS}]}{2}\right)\left(\frac{r}{i+1} + 1\right)$$

S7.23

$$\frac{d[\alpha(r+1)]}{di} = \frac{\left(at + \frac{k_2[\mathrm{FTPS}]}{2}\right)(i+1)^2 - br}{(i+1)^2}$$

S7.24

$$(i+1) < \sqrt{\frac{2br}{2at + k_2[\mathrm{FTPS}]}}$$

S7.2.4. Stoichiometric reaction between substrate 'S' and TBAT

Consider a substrate, 'S', undergoing stoichiometric reaction with TBAT via direct (k_d) or dissociative (k_F) pathways, to generate product, 'P', plus FTPS; Scheme S7.2.

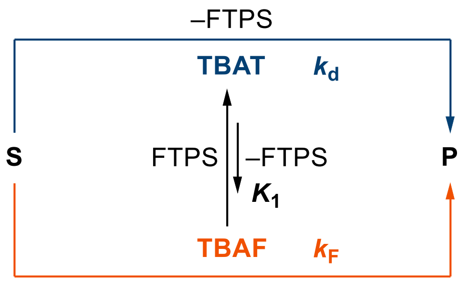

Scheme S7.2: A general scheme for substrate 'S' undergoing stoichiometric reaction with TBAT via direct (k_d) or dissociative (k_F) pathways.

TBAT, FTPS and S are mixed in such amounts that their concentrations at t = 0 are $[\mathrm{TBAT}]_0$, $[\mathrm{FTPS}]_{\mathrm{exog}}$ and $[\mathrm{S}]_0$, respectively. Assuming that the fluorination of S is much slower than the equilibrium dissociation of TBAT into FTPS + TBAF (instantaneous dissociation of TBAT), and no other reactions, the equilibrium concentrations of the three species at t = 0 are: $[\mathrm{TBAT}]_0 - x$, $[\mathrm{FTPS}]_{\mathrm{exog}} + x$, and $[\mathrm{S}]_0$. The equilibrium concentration of TBAF at t = 0 is x. As the reaction progresses, TBAT and S are consumed, and FTPS and P are formed. The (equilibrium) concentrations at time t are:

- $[\mathrm{TBAT}]_t = [\mathrm{TBAT}]_0 - x - [\mathrm{P}]_t$
- $[\mathrm{FTPS}]_t = [\mathrm{FTPS}]_{\mathrm{exog}} + x + [\mathrm{P}]_t$
- $[\mathrm{TBAF}]_t = x$
- $[\mathrm{S}]_t = [\mathrm{S}]_0 - [\mathrm{P}]_t$
- $[\mathrm{P}]_t$

The rates of formation of P *via* the direct and dissociative pathways are given by Equations S7.25 and S7.26, respectively.

S7.25

$$\left(\frac{d[\mathrm{P}]_t}{dt}\right)_{\mathrm{d}} = k_{\mathrm{d}}[\mathrm{TBAT}]_t[\mathrm{S}]_t = k_{\mathrm{d}}([\mathrm{TBAT}]_0 - x - [\mathrm{P}]_t)([\mathrm{S}]_0 - [\mathrm{P}]_t)$$

S7.26

$$\left(\frac{d[\mathrm{P}]_t}{dt}\right)_{\mathrm{F}} = k_{\mathrm{F}}[\mathrm{TBAF}]_t[\mathrm{S}]_t = k_{\mathrm{F}}x([\mathrm{S}]_0 - [\mathrm{P}]_t)$$

Under the regime of instantaneous equilibrium dissociation of TBAT, Equation S7.27 describes the equilibrium between TBAT, FTPS and TBAF at any time *t*. Since $K_1 << 1$, $x <<$ $[\mathrm{TBAT}]_0$; we also note that soon after the start of the reaction, $x << [\mathrm{P}]_t$. Solving Equation S7.27 for *x* gives Equation S7.28, which can be inserted into Equations S7.25 and S7.26 to give Equations S7.29 and S7.30.

S7.27

$$K_1 = \frac{[\mathrm{FTPS}]_t[\mathrm{TBAF}]_t}{[\mathrm{TBAT}]_t} = \frac{\left([\mathrm{FTPS}]_{\mathrm{exog}} + x + [\mathrm{P}]_t\right)x}{[\mathrm{TBAT}]_0 - x - [\mathrm{P}]_t} \approx \frac{\left([\mathrm{FTPS}]_{\mathrm{exog}} + [\mathrm{P}]_t\right)x}{[\mathrm{TBAT}]_0 - [\mathrm{P}]_t}$$

S7.28

$$x \approx \frac{K_1([\mathrm{TBAT}]_0 - [\mathrm{P}]_t)}{[\mathrm{FTPS}]_{\mathrm{exog}} + [\mathrm{P}]_t}$$

S7.29

$$\left(\frac{d[\mathrm{P}]_t}{dt}\right)_{\mathrm{d}} \approx k_{\mathrm{d}}([\mathrm{TBAT}]_0 - [\mathrm{P}]_t)([\mathrm{S}]_0 - [\mathrm{P}]_t)$$

S7.30

$$\left(\frac{d[\mathrm{P}]_t}{dt}\right)_{\mathrm{F}} \approx \frac{K_1 k_{\mathrm{F}}}{[\mathrm{FTPS}]_{\mathrm{exog}} + [\mathrm{P}]_t}([\mathrm{TBAT}]_0 - [\mathrm{P}]_t)([\mathrm{S}]_0 - [\mathrm{P}]_t)$$

The overall rate of formation of P is given by Equation S7.31.

S7.31

$$\frac{d[\mathrm{P}]_t}{dt} \approx \left(\frac{K_1 k_{\mathrm{F}}}{[\mathrm{FTPS}]_{\mathrm{exog}} + [\mathrm{P}]_t} + k_{\mathrm{d}}\right)([\mathrm{TBAT}]_0 - [\mathrm{P}]_t)([\mathrm{S}]_0 - [\mathrm{P}]_t)$$

The fractionation of the direct pathway, f_{d}, is therefore given by Equation S7.32; and that of the dissociative pathway, $f_{\mathrm{F}} = 1 – f_{\mathrm{d}}$, by Equation S7.33.

S7.32

$$f_{\mathrm{d}} = \frac{(d[\mathrm{P}]_t)_{\mathrm{d}}}{d[\mathrm{P}]_t} \approx \frac{1}{1 + \frac{K_1 k_{\mathrm{F}}}{k_{\mathrm{d}}\left([\mathrm{FTPS}]_{\mathrm{exog}} + [\mathrm{P}]_t\right)}}$$

S7.33

$$f_{\mathrm{F}} \approx \frac{1}{1 + \frac{k_{\mathrm{d}}\left([\mathrm{FTPS}]_{\mathrm{exog}} + [\mathrm{P}]_t\right)}{K_1 k_{\mathrm{F}}}}$$

S7.3. Water content

S7.3.1. FTPS

In the glovebox, MeCN-d_3 (0.300 mL, accurately) was transferred to a J Young® valve capped NMR tube *via* a microsyringe, and a ^{1}H NMR spectrum (8 scans) of the sample was acquired. The solution was then titrated with five aliquots of solid FTPS (also in the glovebox). After each aliquot addition, the NMR tube was shaken thoroughly, and a ^{1}H NMR spectrum of the sample was acquired.

The concentration of the residual solvent peak was determined as [CHD_2CN] = 115 mM, based on integration of the signal against FTPS (Table S7.1); which corresponds to the isotopic purity of 99.8% (the minimum purity assured by the supplier).

Table S7.1: Integration of residual CHD_2CN against FTPS.

Aliquot	Mass of FTPS in aliquot (mg)	Total concentration of FTPS (mM)	Integral ratio (FTPS/solvent)
1	12.2	146	18.7
2	14.2	315	41.0
3	11.8	457	59.8
4	14.0	624	81.7
5	17.8	837	109

Water was quantified against ^{13}C satellites of the solvent peak (each of the two satellites corresponded to 636 μM). The calculated concentrations of water were plotted against the total concentration of FTPS, [FTPS], and are presented in Figure S7.4a. The water content in the final sample (837 mM FTPS) was then re-measured over ~2 h, and the calculated concentrations of water were plotted against time (*t*, from addition of the final aliquot of FTPS); Figure S7.4b. The plots show that water content in the sample was unaffected by addition of FTPS (*i.e.* the water came solely from the solvent and/or the glassware) and constant over time (*i.e.* no hydrolytic decomposition of FTPS). The average concentration of water, from all ten measurement, was [H_2O] = (206 ± 11) μM.

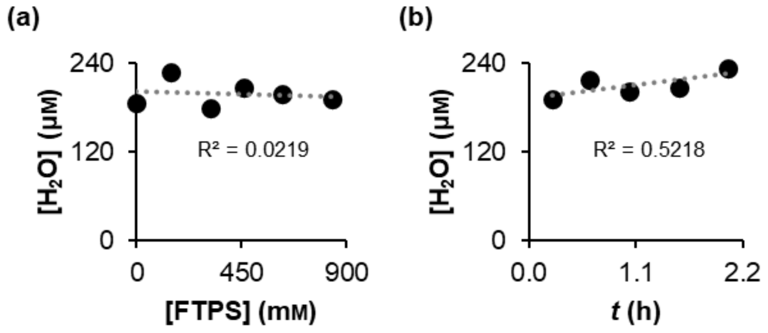

Figure S7.4: (a) Water content in the sample *vs* total concentration of FTPS in a titration of MeCN-d_3 with solid FTPS. (b) Water content in the sample obtained from the final addition of FTPS, measured over time.

S7.3.2. THF and MeCN

Water contents in dry (non-deuteriated) THF and MeCN were estimated from a Karl-Fischer titration (Metrohm 831 Coulometer). Solvent samples were transported from the glovebox to the titrator (outside the glovebox) in standard 1 mL syringes, with the tips of the needles inserted into a septum cap to seal the solvents from air. Upon removal of a needle from the septum cap, the sample was promptly injected into the titrator. The measurements were performed in quadruplicate (each time from a new syringe). The readings in ppm are presented in Table S7.2, along with the conversions to water concentrations (1 ppm = $10^6 \cdot [H_2O] \cdot M_w / d_{solv}$; M_w is the molar mass of water and d_{solv} is the solvent density).

Table S7.2: Estimating water contents in THF and MeCN from a Karl-Fischer titration.

Sample	Water content (ppm)	$[H_2O]$[a] (μM)
THF, 1	0.9	40
THF, 2	1.4	70
THF, 3	1.2	60
THF, 4	1.2	60
MeCN, 1	0.7	30
MeCN, 2	0.9	40
MeCN, 3	1.3	60
MeCN, 4	0.9	40

Firstly, significant discrepancies were found between the four readings for each solvent, which could be attributed to non-negligible amounts of adventitious water in the samples – from the syringes, needles and/or leaked air. Secondly, whilst the water content determined for MeCN (an average of ~1.0 ppm) was reasonable,[S2] THF gave significantly lower readings than expected based on the the value reported in literature (an average of ~1.2 ppm herein *cf.* a reported 4.1 ppm) for THF dried using a similar method,[S2] *i.e.* storage of the solvent over 3 Å molecular sieves (3Å-MS); see Section S1.1. However, THF used in this study was dispensed from a solvent purification system fitted inside the glovebox (directly into the container preloaded with 3Å-MS), and hence already significantly drier than the "wet" solvent (108 ppm water) used in the cited publication as a starting point. Moreover, the titrations described in this section were performed after several weeks of storing THF over 3Å-MS, as opposed to 72 h in the publication. It is unclear whether 4.1 ppm represented an equilibrium water content in the solvent, or whether it decreased further after the measurement at 72 h. A different loading of 3Å-MS could play an additional role in the discrepancy between the measured and "expected" water content.

Therefore, the concentration of water in THF and MeCN used in this study was estimated to be between 30 μM and 70 μM in both solvents, albeit with caution.

[a] To the nearest 10 μM.

S8. References

S1. L. Merakeb, S. Bennaamane, J. De Freitas, E. Clot, N. Mézailles and M. Robert, *Angew. Chemie Int. Ed.*, 2022, **61**, e202209899.

S2. D. B. G. Williams and M. Lawton, *J. Org. Chem.*, 2010, **75**, 8351–8354.

S3. C. J. Handy, Y. F. Lam and P. DeShong, *J. Org. Chem.*, 2000, **65**, 3542–3543.

S4. D. H. Wu, A. Chen and C. S. Johnson, *J. Magn. Reson. Ser. A*, 1995, **115**, 260–264.

S5. H. Kessler, H. Oschkinat, C. Griesinger and W. Bermel, *J. Magn. Reson.*, 1986, **70**, 106–133.

S6. J. Stonehouse, P. Adell, J. Keeler and A. J. Shaka, *J. Am. Chem. Soc.*, 1994, **116**, 6037–6038.

S7. K. Stott, J. Keeler, T. L. Hwang, A. J. Shaka and J. Stonehouse, *J. Am. Chem. Soc.*, 1995, **117**, 4199–4200.

S8. J. Zhou and P. C. M. va. Zijl, *Prog. Nucl. Magn. Reson. Spectrosc.*, 2006, **48**, 109–136.

S9. P. C. M. Van Zijl and N. N. Yadav, *Magn. Reson. Med.*, 2011, **65**, 927–948.

S10. H. Sun and S. G. DiMagno, *J. Am. Chem. Soc.*, 2005, **127**, 2050–2051.

S11. R. K. Sharma and J. L. Fry, *J. Org. Chem.*, 1983, **48**, 2112–2114.

S12. F. W. Dahlquist, K. J. Longmuir and R. B. D. Vernet, *J. Magn. Reson.*, 1975, **17**, 406–410.

S13. J. B. Lambert and J. W. Keepers, *J. Magn. Reson.*, 1980, **38**, 233–244.

S14. Q. Zheng, H. Xu, H. Wang, W. G. H. Du, N. Wang, H. Xiong, Y. Gu, L. Noodleman, K. B. Sharpless, G. Yang and P. Wu, *J. Am. Chem. Soc.*, 2021, **143**, 3753–3763.

S15. S. D. Schimler, R. D. J. Froese, D. C. Bland and M. S. Sanford, *J. Org. Chem.*, 2018, **83**, 11178–11190.

S16. M. Mąkosza and R. Bujok, *J. Fluor. Chem.*, 2005, **126**, 209–216.

S17. S. Forsén and R. A. Hoffman, *J. Chem. Phys.*, 1963, **39**, 2892–2901.

S18. S. Forsén and R. A. Hoffman, *J. Chem. Phys*., 1964, **40**, 1189–1196.

S19. R. E. D. Mcclung and G. H. M. Aarts, *J. Magn. Reson. Ser. A*, 1995, **115**, 145–154.

S20. A. D. Bain and D. A. Fletcher, *Mol. Phys*., 1998, **95**, 1091–1098.

S21. P. Vallurupalli, A. Sekhar, T. Yuwen and L. E. Kay, *J. Biomol. NMR*, 2017, **67**, 243–271.

S22. D. Columbus, V. Arunachalam, F. Glang, L. Avram, S. Haber, A. Zohar, M. Zaiss and M. Leskes, *J. Am. Chem. Soc*., 2022, **144**, 9836–9844.

S23. J. R. Alger and J. H. Prestegard, *J. Magn. Reson*., 1977, **27**, 137–141.

S24. T. R. Brown and S. Ogawa, *Proc. Nat. Acad. Sci*., 1977, **74**, 3627–3631.

S25. F. Bloch, *Phys. Rev*., 1946, **70**, 460–474.

S26. H. S. Gutowsky and A. Saika, *J. Chem. Phys*., 1953, **21**, 1688–1694.

S27. H. M. McConnell, *J. Chem. Phys*., 1958, **28**, 430–431.

S28. A. Allerhand and H. S. Gutowsky, *J. Chem. Phys*., 1964, **41**, 2115–2126.

S29. C. R. Malloy, A. D. Sherry and R. L. Nunnally, *J. Magn. Reson*., 1985, **64**, 243–254.

S30. C. L. Perrin and E. R. Johnston, *J. Magn. Reson*., 1979, **33**, 619–626.

S31. M. Grassi, B. E. Mann, B. T. Pickup and C. M. Spencer, *J. Magn. Reson*., 1986, **69**, 92–99.

S32. A. D. Bain and J. A. Cramer, *J. Magn. Reson. - Ser. A*, 1996, **118**, 21–27.

S33. K. Uğurbil, *J. Magn. Reson*., 1985, **64**, 207–219.

S34. R. Freeman, *Prog. Nucl. Magn. Reson. Spectrosc*., 1998, **32**, 59–106.

S35. G. Liu, X. Song, K. W. Y. Chan and M. T. Mcmahon, *NMR Biomed*., 2013, **26**, 810–828.
